# Meta-analysis of the clinical performance of commercial SARS-CoV-2 nucleic acid and antibody tests up to 22 August 2020

**DOI:** 10.2807/1560-7917.ES.2021.26.45.2001675

**Published:** 2021-11-11

**Authors:** Ivo Van Walle, Katrin Leitmeyer, Eeva K Broberg, the European COVID-19 microbiological laboratories group, European COVID-19 microbiological laboratories group, Marjan Van Esbroeck, Kurt Beuselinck, Pieter Vermeersch, Christos Karagiannis, Andreas Mentis, Stavroula Lampropoulou, Iris Erlund, Merit Melin, Nina Ekström, Terhi Vihervaara, Alexandre Gaymard, Emilie Frobert, Vanessa Escuret, Ivan-Christian Kurolt, Guillaume Fournier, Tamir Abdelrahman, Trung Nguyen, Adrian Klak, Anne E Bos, Anne Russcher, Annemarie van ’t Veen, Annette M Stemerding, Annette van Corteveen-Splinter, Babette C van Hees, Bas B Wintermans, Bjorn L Herpers, Chantal BEM Reusken, Christel FM van der Donk, Claudy Oliveira dos Santos, Corine H GeurtsvanKessel, Cornelis P Timmerman, David SY Ong, Deborah J Kaersenhout, Ellen van Lochem, Felix Geeraedts, Ger T Rijkers, Hannke Berkhout, Hans GM Koeleman, Inge HM van Loo, Janette Rahamat-Langendoen, Jean-Luc Murk, Jeroen HT Tjhie, Johan Kissing, Johan Reimerink, Jos J Kerremans, Jutte JC de Vries, Karen A Heemstra, Khoa TD Thai, Kin Ki Jim, Leontine Mulder, Maaike JC van den Beld, Manou R Batstra, Maria M Konstantinovski, Marjolijn CA Wegdam-Blans, Martine Hoogewerf, Melanie J de Graaf, Menno D. de Jong, Michiel Heron, Michiel van Rijn, Moniek Heusinkveld, Nathalie Van Burgel, Paul HM Savelkoul, Paul Martijn den Reijer, Peter C Wever, Peter Croughs, Rens Zonneveld, Sim van Gyseghem, Steven FT Thijsen, Susanne P Stoof, Suzanne Jurriaans, Sylvia B Debast, Theo Mank, Vishal Hira, Aleksander Michalski, Anna Siewierska-Puchlerska, Ewa Gajda, Jarosław Paciorek, Marta Pakieła, Agnieszka Kołakowska-Kulesza, Katarzyna Pancer, Magdalena Nowakowska, Inês Costa, Líbia Zé-Zé, Raquel Guiomar, Berit Hammas, Johan Brynedal Öckinger, Katarina Prosenc, Nataša Berginc

**Affiliations:** 1 European Centre for Disease Prevention and Control, Stockholm, Sweden; 2 Centre for Infectious Disease Control, National Institute for Public Health and the Environment, The Netherlands; 3 The members of the European COVID-19 microbiological laboratories group are listed under Investigators

**Keywords:** COVID-19, SARS-CoV-2, diagnostic, accuracy, sensitivity, specificity, meta-analysis

## Abstract

**Background:**

Reliable testing for SARS-CoV-2 is key for the management of the COVID-19 pandemic.

**Aim:**

We estimate diagnostic accuracy for nucleic acid and antibody tests 5 months into the COVID-19 pandemic, and compare with manufacturer-reported accuracy.

**Methods:**

We reviewed the clinical performance of SARS-CoV-2 nucleic acid and antibody tests based on 93,757 test results from 151 published studies and 20,205 new test results from 12 countries in the European Union and European Economic Area (EU/EEA).

**Results:**

Pooling the results and considering only results with 95% confidence interval width ≤ 5%, we found four nucleic acid tests, including one point-of-care test and three antibody tests, with a clinical sensitivity ≥ 95% for at least one target population (hospitalised, mild or asymptomatic, or unknown). Nine nucleic acid tests and 25 antibody tests, 12 of them point-of-care tests, had a clinical specificity of ≥ 98%. Three antibody tests achieved both thresholds. Evidence for nucleic acid point-of-care tests remains scarce at present, and sensitivity varied substantially. Study heterogeneity was low for eight of 14 sensitivity and 68 of 84 specificity results with confidence interval width ≤ 5%, and lower for nucleic acid tests than antibody tests. Manufacturer-reported clinical performance was significantly higher than independently assessed in 11 of 32 and four of 34 cases, respectively, for sensitivity and specificity, indicating a need for improvement in this area.

**Conclusion:**

Continuous monitoring of clinical performance within more clearly defined target populations is needed.

## Introduction

Testing is one of the central pillars of public health actions in epidemic and pandemic situations to allow timely identification, contact tracing and isolation of infectious cases to reduce the spread of infectious diseases. In addition, it allows estimating disease incidence, disease prevalence, and prevalence and duration of humoral immunity. Reliable testing for severe acute respiratory syndrome coronavirus 2 (SARS-CoV-2) and timely reporting of the data to public health authorities is therefore key for the management of the coronavirus disease (COVID-19) pandemic. This requires appropriate and sufficiently accurate diagnostic tests to identify individuals who are currently infected with SARS-CoV-2 as well as those who have been infected in the past. Timely access to testing, sufficient supply of testing materials, availability of tests and related reagents and consumables as well as high-throughput testing are pivotal in this context.

By August 2020, a large number of commercial tests for SARS-CoV-2 RNA detection (nucleic acid tests) were available, as well as serological tests for SARS-CoV-2-specific antibodies. The various types of tests can be used for different purposes and many of these tests have the CE certificate for in vitro diagnostics (CE-IVD) that indicates compliance with the European IVD directive (98/79/EC) and can thus be marketed in the countries in the European Union and European Economic Area (EU/EEA). In addition, the United States (US) Food and Drug Administration has granted emergency use authorisations for many commercial tests in the US, and the World Health Organization (WHO) maintains an emergency use listing of commercial tests [1,2]. It is, however, important to note that CE certification is based on a self-declaration of the test manufacturer, including the claims on performance of the test. Independent information on the clinical performance of these tests in terms of sensitivity and specificity is still limited, and yet this is critical for proper interpretation of results.

For this reason, the European Centre for Disease Prevention and Control (ECDC) launched a continuous call to EU/EEA countries and the United Kingdom (UK) on 1 April 2020 to provide any such clinical performance data for sharing with other countries. These data, provided by 12 countries, are presented in this article. In addition, we included publicly available data. Finally, minimal performance criteria for different intended uses were gathered from public sources and aided by a survey conducted among EU/EEA countries and the UK from 20 May to 1 June 2020.

## Methods

### Search strategy and selection criteria

Studies containing potentially usable data on the clinical performance of SARS-CoV-2 nucleic acid and antibody tests were first extracted from systematic reviews on this topic. We identified these reviews through an initial PubMed (Medline) search for systematic reviews and meta-analyses for ‘COVID-19’ and ‘SARS-CoV-2’, followed by snowballing using the ‘find similar articles’ feature. We extended the selection with the studies listed in the Foundation for Innovative Diagnostics database (FIND, www.finddx.org/covid-19/tests) and the European Commission COVID-19 In Vitro Diagnostic Devices and Test Methods Database (EC, https://covid-19-diagnostics.jrc.ec.europa.eu). Both databases attempt to exhaustively identify peer-reviewed as well as grey literature on clinical performance of COVID-19 tests and are continuously updated [3,4]. Results from the latter were further filtered for articles with a description indicating that they contain clinical performance results. We also included results produced by the US Food and Drug Administration (FDA) [5]. Finally, we searched PubMed according to the query shown in Supplement 1.

The resulting studies were subsequently assessed for eligibility. By August 2020 there were no clinical performance studies that can be judged as having low risk of bias and low applicability concerns. Systematic reviews up to that point have not used risk of bias or applicability concerns as exclusion criteria [6-9]. This was not done in this work either. Instead, we excluded studies if they did not contain data on commercial tests, or if one or more of the authors were employed by the developer or manufacturer of the index test, to avoid possible conflicts of interest. Subsequently, we also excluded studies with an ineligible design, such as blinded tests, analytical validation only, use of another threshold for positivity than in the instructions for use, comparisons between different specimen types or use of an antibody rather than nucleic acid test as reference test for any type of index test.

Further exclusions were made at sample level based on the reference test employed. Samples classified as actual negatives, i.e. used for determining specificity, had to be taken (i) before the COVID-19 outbreak, in practice before 2020, (ii) from an individual without COVID-19-compatible symptoms, or (iii) from an individual with COVID-19-compatible symptoms but who was confirmed with another respiratory illness. Samples classified as actual negatives that were taken during the outbreak and were negative according to a nucleic acid test were therefore excluded. We did this to maximally reduce misclassification as actual negatives because of known issues with sensitivity of nucleic acid tests. Such misclassified samples would artificially lower index test specificity, in particular when the index test is more sensitive than the reference test [10-16]. For the same reason, the reported sensitivity of nucleic acid index tests, based on a nucleic acid reference test, was considered to be a positive agreement instead, calculated as part of a head-to-head comparison between the two tests. For antibody index tests on the other hand, we considered a nucleic acid test to be a valid reference test to determine actual positive samples and sensitivity, in accordance with WHO interim guidelines [17].

Manufacturer-reported clinical sensitivity and specificity data were extracted from instructions for use where available, or otherwise from the manufacturer’s website. Sensitivity results derived from contrived samples spiked with purified viral RNA were excluded.

### Original clinical performance data

Primary clinical performance data generated by the COVID-19 microbiological laboratories author group were assessed by the ECDC according to the same criteria as those of the literature review.

### Statistical analysis

Meta-analysis of the included clinical sensitivity and specificity results was performed per test and per target, i.e. the genomic region for nucleic acid tests and the antibody isotype for antibody tests. Antibody test sensitivity results below the threshold number of days after onset were excluded. Sensitivity and positive agreement results were further stratified by case population as hospitalised cases, mild or asymptomatic cases, or unknown. We calculated pooled sensitivity and specificity values using fixed effects analysis, i.e. separately summing and dividing the number of correct predictions by the total number of samples in the group. Wilson score 95% confidence intervals (CI) were calculated for pooled results. Study heterogeneity was assessed through the I^2^ statistic, calculated through random effects analysis using R version 4.0.2 and the metafor package [18]. We considered I^2^ values < 50.0% as low heterogeneity, 50.0–74.9% as moderate and ≥ 75% as high heterogeneity.

## Results

### Minimum performance criteria

By 1 June 2020, minimum performance criteria for tests were publicly available from Belgium, France, the Netherlands and the UK (Supplementary Table S1). All were applicable solely to antibody tests. The intended uses included diagnosis of COVID-19, determination of exposure to SARS-CoV-2 and determination of the immune status against SARS-CoV-2. Minimum clinical sensitivity for all of the specified intended uses ranged from 85% to 98%, with a median of 95%. These thresholds applied to samples collected at least 15 days post onset of symptoms (dpo), taking into account the time to seroconversion. Minimum clinical specificity for all of the specified intended uses was 98% in three countries and 98.5% in one. For nucleic acid confirmatory tests, the draft WHO Target Product Profiles for priority diagnostics to support response to the COVID-19 pandemic state > 95% to > 98% sensitivity (acceptable/desired) and > 99% specificity [19].

We used general thresholds of > 95% sensitivity and > 98% specificity to determine if a test met the minimum performance criteria, together with a maximum 95% CI width ≤ 5%. For results on IgM antibodies only, an upper limit of ≤ 28 dpo, or the highest dpo category with an upper limit ≤ 28 dpo, was added since IgM antibodies decrease fairly rapidly and such tests are not intended to be used long after exposure [20]. These sensitivity and specificity thresholds can be converted to false positives (FP) and negatives (FN), and positive and negative predictive value (PPV, NPV) if the prevalence of the condition, i.e. SARS-CoV-2 nucleic acid or antibody positivity, is known. These metrics better express the real impact of the accuracy. For a hypothetical low prevalence of 1% in a population of 100,000 people, the PPV would be > 32.4% (FP < 1,980) and NPV > 99.9% (FN < 50). For a high prevalence of 5%, these values would be > 71.4% (FP < 1,900) and > 99.7% (FN < 250). Finally, for a high prevalence of 30%, PPV would be > 95.3% (FP < 1,400) and NPV > 97.9% (FN < 1,500).

### Primary clinical performance data

We identified eight systematic reviews, including one by health technology assessment bodies not listed as a peer-reviewed study, and included the primary studies they were based on [6-9,21-24]. The full list of studies in the FIND and EC databases was retrieved on 22 August 2020. PubMed was searched on the same date. From the EC database, 268 of 385 studies were screened out because their description did not indicate that they contained clinical performance data on commercial tests. Of the remaining 117 studies, 81 were not present in the FIND database and 82 were not present in the EC database. From the PubMed results, 1,520 of 1,738 studies were screened out. From the combined list of 364 unique studies, 105 had no clinical performance data on commercial nucleic acid or antibody tests, 34 were excluded because of a potential conflict of interest and 74 were excluded because of ineligible design, leaving a total of 151 included studies. Of those, 53 were exclusively found through the Pubmed search and 15 in the FIND database. The remaining studies were listed by at least two sources.

**Figure 1:**
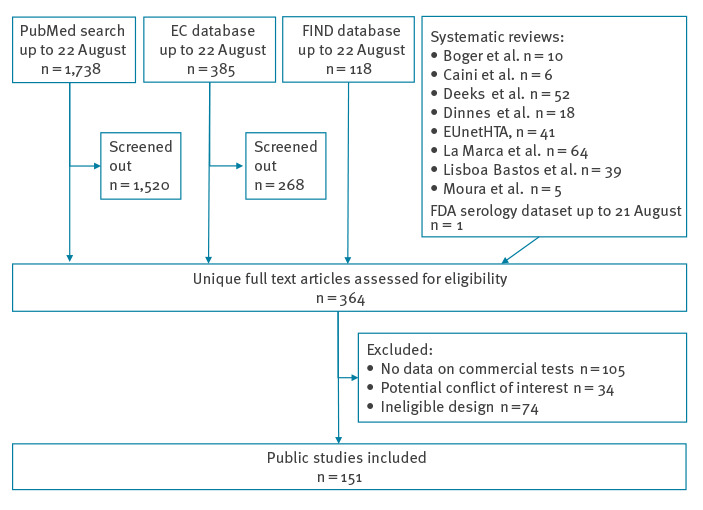
Selection of public studies on clinical performance of SARS-CoV-2 nucleic acid and antibody tests, up to 22 August 2020 (n = 151)

EC: European Commission COVID-19 In Vitro Diagnostic Devices and Test Methods database; FDA: United States Food and Drug Administration; FIND: Foundation for Innovative Diagnostics database; SARS-CoV-2: severe acute respiratory syndrome coronavirus 2.

A complete overview of the study selection is given in Figure 1. After exclusion of antibody test sensitivity results ≤ 14 dpo and ineligible specificity results, a total of 37,435 and 56,322 index test results remained for calculation of sensitivity and specificity, respectively. After addition of original, previously unpublished results provided by the authors of this study, this increased to 47,543 and 66,419 index test results, respectively, for 198 tests. A descriptive overview of the number of studies and results per country in given in Table 1. A complete overview of the studies is given in Supplementary Tables S2-S4.

**Table 1:** Descriptive statistics on the number of published studies on clinical performance of SARS-CoV-2 nucleic acid and antibody tests, whether we included additional original data, and number of samples included in the meta-analysis, up to 22 August 2020 (n = 151 studies)

Country	Studies	Original data	PCR sens/spec	CLIA sens/spec	ELISA sens/spec	LFIA sens/spec	Other^a^ sens/spec	Total sens/spec
Australia	3	No	125/59	0/0	209/0	1,511/1,012	0/0	1,845/1,071
Austria	5	No	115/75	195/2,308	421/0	220/0	0/0	951/2,383
Belgium	6	Yes	22/6	1,192/1,031	957/922	3,934/2,985	287/254	6,392/5,198
Brazil	1	No	0/0	0/0	0/0	0/100	0/0	0/100
Canada	1	No	0/0	84/150	185/150	499/450	0/0	768/750
China	17	No	364/0	3,659/1,572	1,494/726	1,038/557	0/0	6,555/2,855
Croatia	0	Yes	168/271	0/0	0/0	0/0	0/0	168/271
Cyprus	0	Yes	6/466	0/0	0/0	0/0	0/0	6/466
Denmark	2	No	0/0	1,495/4,421	195/1,403	126/62	0/0	1,816/5,886
Ecuador	1	No	33/21	0/0	0/0	0/0	0/0	33/21
Finland	3	Yes	121/75	0/82	64/238	0/242	0/0	185/637
France	13	Yes	567/324	173/165	515/154	1,160/486	154/625	2,569/1,754
Germany	9	No	85/200	643/1,597	508/568	32/13	0/0	1,268/2,378
Greece	0	Yes	0/0	0/0	139/20	0/0	0/0	139/20
Hong Kong SAR	1	No	72/114	0/0	0/0	0/0	0/0	72/114
Italy	10	No	0/0	139/37	531/203	60/97	0/0	730/337
Japan	5	No	340/435	0/0	0/0	735/245	98/111	1,173/791
Luxembourg	0	Yes	0/0	0/0	235/218	0/0	0/0	235/218
The Netherlands	4	Yes	253/210	415/1,177	2,107/3,449	2,336/1,642	0/0	5,111/6,478
Norway	1	No	0/0	0/0	0/0	207/0	0/0	207/0
Poland	0	Yes	390/662	0/0	0/0	0/0	0/0	390/662
Portugal	0	Yes	0/0	0/0	0/0	22/28	0/0	22/28
Singapore	2	No	0/0	202/878	0/0	0/0	0/0	202/878
Slovenia	1	Yes	168/641	0/0	0/0	0/0	0/0	168/641
South Korea	1	No	0/0	0/0	0/0	140/158	0/0	140/158
Spain	4	No	0/0	0/0	0/124	806/566	0/0	806/690
Sweden	2	Yes	39/4	58/113	0/0	78/248	0/0	175/365
Switzerland	6	No	1,920/3,816	0/0	312/50	129/50	100/200	2,461/4,116
Taiwan	1	No	0/0	0/0	0/0	129/0	0/0	129/0
United Kingdom	17	No	15/1710	1,975/5,247	65/0	412/200	0/0	2,467/7,157
United States	35	No	2,273/2,628	1,260/4,164	794/769	5,446/11,140	587/1,295	10,360/19,996
**Total**	**151 **	**NA**	**7,076/11,717**	**11,490/22,942**	**8,731/8,994**	**19,020/20,281**	**1,226/2,485**	**47,543/66,419**

CLIA: chemiluminescence assay; ELISA: enzyme-linked immunosorbent assay; LFIA: lateral flow immunoassay; sens/spec: number of samples that are reference test positive/negative; NA: not applicable; SARS-CoV-2: severe acute respiratory syndrome coronavirus 2.

^a^ Includes loop-mediated isothermal amplification, microarray, transcription-mediated amplification, and enzyme-linked fluorescent assay.

### Meta-analysis

Pooled estimates for clinical sensitivity and specificity per test, target and, for sensitivity, case population were made. For antibody tests, we restricted the results to those estimates that had a 95% CI width ≤ 5% and were derived from at least two studies, to be able to assess study heterogeneity. Based on the minimum performance criteria analysis, results ≥ 95% sensitivity and/or ≥ 98% specificity for a particular population are highlighted in Table 2. Among these results, there were two CLIA, one ELISA and no LFIA/POC that had ≥ 95% sensitivity and nine CLIA, four ELISAs and 12 LFIA/POC that had ≥ 98% specificity, including the three with ≥ 95% sensitivity. Study heterogeneity was low for four of 10 sensitivity and 53 of 69 specificity results with CI width ≤ 5%. There were few sensitivity results for IgG for mild or asymptomatic cases, for IgA and for total antibody, none of which had a CI width ≤ 5%. In four cases where the same test was used for hospitalised cases, a reduction in sensitivity was observed of 7.4%, 11.0%, 13.1% and 19.2% for IgG (Table 2). For IgA and total antibody, data were available for only one test each. A reduction of 28.8% was observed for IgA and an increase of 6.0% for total antibody. The latter increase was probably due to the small number of samples for both populations.

**Table 2:** Pooled sensitivity and specificity results for SARS-CoV-2 antibody tests with confidence interval width ≤ 5% for either or both and based on at least two studies, up to 22 August 2020

Category	Test	Target	Case population	Sensitivity^a^	Specificity^a^
CLIA	Abbott, SARS-CoV-2 IgG assay on Architect	IgG	Hospitalised	**95.9 (93.4–97.5)** n = 368 BE, CA, NL, UK, US(3)	**99.5 (99.3–99.6)** n = 8,243 AT, BE(2), CA, DE(2), DK, FI, FR(3), IT, NL, SE, SG, UK(3), US(8)
CLIA	Abbott, SARS-CoV-2 IgG assay on Architect	IgG	Mild/asymptomatic	88.5 (84.6–91.5) ^b^ n = 331 NL, UK(2), US	Same as above
CLIA	Abbott, SARS-CoV-2 IgG assay on Architect	IgG	Unk	92.0 (90.4–93.3) n = 1,332 AT, BE, DE, DK, FI, FR(2), SE, SG, UK(2), US(4)	Same as above
LFIA, POC	Anhui Deep Blue Medical Technology, COVID-19 (SARS-CoV-2) IgG/IgM Antibody Test Kit	IgG	Na	Nd	**99.4 (96.5–99.9)** n = 158 CA, US
ELISA	Beijing Wantai Biological Pharmacy Enterprise, Wantai SARS-CoV-2 IgM ELISA	IgM	Hospitalised	92.8 (88.3–95.7) ^b,c^ n = 195 CN(2), NL	**98.7 (98.0–99.1)** n = 1,505 CN(2), DK, NL(2)
ELISA	Beijing Wantai Biological Pharmacy Enterprise, Wantai SARS-CoV-2 total Ab ELISA	Total Ab	Hospitalised	**97.5 (95.9–98.5)** ^c^ n = 603 CN(2), DE, DK, NL	**99.5 (99.2–99.7)** n = 3,097 CN(2), DE, DK(2), FR(2), NL(3)
ELISA	Beijing Wantai Biological Pharmacy Enterprise, Wantai SARS-CoV-2 total Ab ELISA	Total Ab	Unk	**97.5 (94.9–98.8)** n = 279 AT, DK, FR	Same as above
ELISA	Bio-Rad, Platelia SARS-CoV-2 Total Ab	Total Ab	Na	Nd	96.4 (93.3–98.1) n = 250 BE, FR, LU, NL
LFIA, POC	CTK Biotech, OnSite COVID-19 IgG/IgM Rapid Test	IgG	Na	Nd	**98.6 (95.2–99.6)** n = 148 AU, NL
CLIA	DiaSorin, Liaison XL S1/S2 IgG chemiluminescence immunoassay	IgG	Hospitalised	92.9 (89.6–95.2) ^b,c^ n = 324 CA, DE, NL	97.7 (97.3–98.0) ^c^ n = 5,994 AT, BE(2), CA, DE(3), DK, FI, FR, NL(2), SE, UK, US(2)
CLIA	DiaSorin, Liaison XL S1/S2 IgG chemiluminescence immunoassay	IgG	Mild/asymptomatic	81.9 (76.3–86.3) ^b^ n = 226 NL, UK	Same as above
CLIA	DiaSorin, Liaison XL S1/S2 IgG chemiluminescence immunoassay	IgG	Unk	90.9 (88.9–92.6) ^d^ n = 967 AT(2), BE(2), DK, SE, UK, US	Same as above
CLIA	Diazyme Laboratories, DZ-Lite SARS-CoV-2 IgM and IgG CLIA	IgG	Unk	95.3 (84.5–98.7) ^b^ n = 43 US(2)	**99.0 (97.5–99.6)** n = 414 US(2)
CLIA	Diazyme Laboratories, DZ-Lite SARS-CoV-2 IgM and IgG CLIA	IgG or IgM	Unk	100.0 (91.8–100.0) ^b^ n = 43 US(2)	**98.6 (96.9–99.3)** n = 414 US(2)
CLIA	Diazyme Laboratories, DZ-Lite SARS-CoV-2 IgM and IgG CLIA	IgM	Unk	90.7 (78.4–96.3) ^b^ n = 43 US(2)	**99.5 (98.3–99.9)** n = 414 US(2)
LFIA, POC	Dynamiker Biotechnology Tianjin, 2019 nCoV IgG/IgM Rapid test	IgG or IgM	Hospitalised	100.0 (89.0–100.0) ^b^ n = 31 BE, DK	97.6 (94.8–98.9) n = 248 BE, DK, SE
LFIA, POC	Dynamiker Biotechnology Tianjin, 2019 nCoV IgG/IgM Rapid test	IgG or IgM	Unk	89.0 (79.8–94.3) ^b,d^ n = 73 SE, TW	Same as above
ELISA	Epitope Diagnostics, EPI-KT-1032 Coronavirus COVID-19 IgG ELISA Kit	IgG	Hospitalised	94.0 (86.7–97.4) ^b,c^ n = 83 CA, NL, US	97.6 (96.7–98.3) ^c^ n = 1,451 AT, CA, DE(2), NL, UK, US(3)
ELISA	Epitope Diagnostics, EPI-KT-1032 Coronavirus COVID-19 IgG ELISA Kit	IgG	Mild/asymptomatic	74.8 (65.8–82.0)^b,d^ n = 107 NL, US	Same as above
ELISA	Epitope Diagnostics, EPI-KT-1032 Coronavirus COVID-19 IgG ELISA Kit	IgG	Unk	96.0 (90.1–98.4) ^b,c^ n = 99 AT, DE, US	Same as above
ELISA	Epitope Diagnostics, EPI-KT-1033 Coronavirus COVID-19 IgM ELISA Kit	IgM	Hospitalised	95.5 (78.2–99.2) ^b,c^ n = 22 CA, NL	**98.1 (97.0–98.9)** n = 810 AT, CA, NL, US
ELISA	Epitope Diagnostics, EPI-KT-1033 Coronavirus COVID-19 IgM ELISA Kit	IgM	Unk	83.3 (70.4–91.3) ^b,c^ n = 48 AT, US	Same as above
ELISA	Euroimmun Medizinische Labordiagnostika, Anti-SARS-CoV-2 IgA S1 ELISA	IgA	Hospitalised	96.0 (92.5–97.9) ^b^ n = 224 BE(2), CA, DK, FI, FR, GR, NL	86.7 (84.9–88.3) ^d^ n = 1,459 AU, BE(2), CA, DK, ES, FI(2), FR(2), GR, LU, NL(2), US
ELISA	Euroimmun Medizinische Labordiagnostika, Anti-SARS-CoV-2 IgA S1 ELISA	IgA	Mild/asymptomatic	67.2 (55.0–77.4) ^b^ n = 64 FI, NL	Same as above
ELISA	Euroimmun Medizinische Labordiagnostika, Anti-SARS-CoV-2 IgA S1 ELISA	IgA	Unk	94.8 (90.9–97.1) ^b^ n = 212 AU, BE, FR, US	Same as above
ELISA	Euroimmun Medizinische Labordiagnostika, Anti-SARS-CoV-2 IgG S1 ELISA	IgG	Hospitalised	92.6 (89.7–94.7) n = 431 BE(3), CA, CH(2), DE, DK, FI, FR, GR, NL, US	97.9 (97.4–98.3) n = 3,954 AU, BE(3), CA, CH(2), DE(6), DK(2), ES, FI(2), FR(3), GR, LU, NL(2), US(5)
ELISA	Euroimmun Medizinische Labordiagnostika, Anti-SARS-CoV-2 IgG S1 ELISA	IgG	Mild/asymptomatic	79.5 (71.9–85.5) ^b,d^ n = 132 CH, FI, NL, US	Same as above
ELISA	Euroimmun Medizinische Labordiagnostika, Anti-SARS-CoV-2 IgG S1 ELISA	IgG	Unk	89.0 (86.7–91.0) ^c^ n = 785 AT, AU, BE, DE(2), DK, FR, UK, US(2)	Same as above
LFIA, POC	Getein Biotech, One Step Test for Novel Coronavirus (2019-nCoV) IgM/IgG Antibody (Colloidal Gold)	IgG	Na	Nd	**100.0 (96.9–100.0)** n = 120 CA, US
LFIA, POC	Getein Biotech, One Step Test for Novel Coronavirus (2019-nCoV) IgM/IgG Antibody (Colloidal Gold)	IgG or IgM	Na	Nd	**99.2 (95.4–99.9)** n = 120 CA, US
LFIA, POC	Getein Biotech, One Step Test for Novel Coronavirus (2019-nCoV) IgM/IgG Antibody (Colloidal Gold)	IgM	Na	Nd	**99.2 (95.4–99.9)** n = 120 CA, US
LFIA, POC	Guangzhou Wondfo Biotech, Wondfo SARS-CoV-2 Antibody Test	IgG or IgM	Unk	88.0 (82.6–92.0) ^b,d^ n = 184 AU, ES, TW, US	**99.3 (98.3–99.7)** n = 605 AU, BR, ES, US(2)
LFIA, POC	Hangzhou Alltest Biotech, 2019-nCoV IgG/IgM Rapid Test Cassette	IgG	Unk	88.7 (81.6–93.3) ^b^ n = 115 AU, ES	**100.0 (98.5–100.0)** n = 254 AU, ES(2)
LFIA, POC	Hangzhou Alltest Biotech, 2019-nCoV IgG/IgM Rapid Test Cassette	IgG or IgM	Unk	92.3 (87.2–95.4) ^b^ n = 168 AU, ES, TW	96.7 (93.8–98.2) n = 269 AU, DK, ES(2)
LFIA, POC	Hangzhou Alltest Biotech, 2019-nCoV IgG/IgM Rapid Test Cassette	IgM	Unk	21.7 (15.2–30.1) ^b,d^ n = 115 AU, ES	97.2 (94.4–98.7) n = 254 AU, ES(2)
LFIA, POC	Innovita Biological Technology, 2019-nCoV Ab Test (Colloidal Gold)	IgG	Hospitalised	86.9 (76.2–93.2) ^b^ n = 61 CA, JP	**100.0 (98.5–100.0)** n = 258 CA, JP, US
LFIA, POC	Innovita Biological Technology, 2019-nCoV Ab Test (Colloidal Gold)	IgM	Hospitalised	75.4 (63.3–84.5) ^b,d^ n = 61 CA, JP	**98.4 (96.1–99.4)** n = 258 CA, JP, US
ELISA	Mikrogen Diagnostik, recomWell SARS-CoV-2 IgG	IgG	Na	Nd	96.4 (94.2–97.8) n = 445 BE, DE, NL
ELISA	NovaTec Immundiagnostica, NovaLisa SARS-CoV-2 IgA ELISA	IgA	Hospitalised	88.7 (78.5–94.4) ^b^ n = 62 BE(2)	95.2 (92.1–97.1) ^c^ n = 293 BE(2), IT, NL
ELISA	NovaTec Immundiagnostica, NovaLisa SARS-CoV-2 IgG ELISA	IgG	Hospitalised	91.9 (82.5–96.5) ^b^ n = 62 BE(2)	97.3 (94.7–98.6) n = 293 BE(2), IT, NL
ELISA	NovaTec Immundiagnostica, NovaLisa SARS-CoV-2 IgM ELISA	IgM	Hospitalised	43.5 (31.9–55.9) ^b,d^ n = 62 BE(2)	**99.0 (97.0–99.7)** n = 293 BE(2), IT, NL
CLIA	Ortho Clinical Diagnostics, VITROS Immunodiagnostic Products Anti-SARS-CoV-2 IgG	IgG	Unk	93.4 (89.4–96.0) ^b^ n = 227 DK, UK	**99.7 (99.3–99.9)** n = 1,420 DK, UK, US
CLIA	Ortho Clinical Diagnostics, VITROS Immunodiagnostic Products Anti-SARS-CoV-2 Total Ab	Total Ab	Na	Nd	**100.0 (99.5–100.0)** n = 732 DK, US
CLIA	Roche, Elecsys Anti-SARS-CoV-2	Total Ab	Hospitalised	85.7 (75.7–92.1) ^b^ n = 70 CA, DE, NL	**99.8 (99.7–99.9)** n = 7,833 AT, BE(3), CA, DE(5), DK, LU, NL, SE, SG, UK(2), US(5)
CLIA	Roche, Elecsys Anti-SARS-CoV-2	Total Ab	Mild/asymptomatic	91.7 (84.4–95.7) ^b,c^ n = 96 NL, UK	Same as above
CLIA	Roche, Elecsys Anti-SARS-CoV-2	Total Ab	Unk	94.7 (93.3–95.7) ^c^ n = 1,351 AT(2), BE(3), DE(2), DK, SE, SG, UK(2), US(2)	Same as above
LFIA, POC	SD BioSensor, Standard Q COVID-19 IgM/IgG Duo	IgG	Na	Nd	**99.8 (99.3–99.9)** ^c^ n = 1,254 US(2)
LFIA, POC	SD BioSensor, Standard Q COVID-19 IgM/IgG Duo	IgM	Na	Nd	**98.8 (98.0–99.3)** n = 1,256 US(2)
CLIA	Shenzhen New Industries Biomedical Engineering (SNIBE), Maglumi 2019-nCoV (SARS-CoV-2) IgG/IgM kit	IgG	Hospitalised	93.4 (85.5–97.2) ^b,c^ n = 76 BE(2)	97.6 (96.8–98.3) ^d^ n = 1,744 BE(2), CN(2), DK
CLIA	Shenzhen New Industries Biomedical Engineering (SNIBE), Maglumi 2019-nCoV (SARS-CoV-2) IgG/IgM kit	IgG	Unk	91.1 (89.2–92.6) ^d^ n = 1084 CN, DK	Same as above
CLIA	Shenzhen New Industries Biomedical Engineering (SNIBE), Maglumi 2019-nCoV (SARS-CoV-2) IgG/IgM kit	IgG or IgM	Hospitalised	96.1 (89.0–98.6) ^b^ n = 76 BE(2)	**98.6 (96.4–99.5)** n = 285 BE(3)
CLIA	Shenzhen New Industries Biomedical Engineering (SNIBE), Maglumi 2019-nCoV (SARS-CoV-2) IgG/IgM kit	IgM	Hospitalised	93.4 (85.5–97.2) ^b,c^ n = 76 BE(2)	**99.2 (98.7–99.5)** ^d^ n = 1,756 BE(2), CN(2), DK
CLIA	Shenzhen New Industries Biomedical Engineering (SNIBE), Maglumi 2019-nCoV (SARS-CoV-2) IgG/IgM kit	IgM	Unk	67.8 (65.0–70.5) ^b,d^ n = 1084 CN, DK	Same as above
CLIA	Shenzhen Yahuilong (YHLO) Biotech, SARS-CoV-2 IgG/IgM antibody detection kit	IgG	Na	Nd	**99.0 (98.3–99.4)** n = 1,313 CN(2), DK, IT
CLIA	Shenzhen Yahuilong (YHLO) Biotech, SARS-CoV-2 IgG/IgM antibody detection kit	IgM	Na	Nd	**98.7 (97.9–99.2)** ^d^ n = 1314 CN(2), DK, IT
CLIA	Siemens, Healthineers SARS-CoV-2 Total Assay on Atellica/ADVIA Centaur	Total Ab	Unk	**96.7 (95.2–97.8)** ^d^ n = 757 DE, DK, UK	**99.8 (99.5–99.9)** n = 2,108 DE(2), DK, UK
LFIA, POC	SureScreen Diagnostic, Covid-19 IgG/IgM Rapid Test Cassette	IgG	Na	Nd	**99.0 (96.4–99.7)** n = 198 BE, NL
LFIA, POC	VivaChek Biotech, VivaDiag COVID-19 IgM/IgG Rapid Test	IgG	Unk	78.9 (69.7–85.9) ^b^ n = 95 AU, US	**98.2 (96.1–99.2)** n = 334 AU, BE, IT, NL, US
LFIA, POC	VivaChek Biotech, VivaDiag COVID-19 IgM/IgG Rapid Test	IgG or IgM	Hospitalised	100.0 (89.0–100.0) ^b^ n = 31 BE, NL	97.5 (95.2–98.7) n = 324 AU, BE, IT, US
LFIA, POC	VivaChek Biotech, VivaDiag COVID-19 IgM/IgG Rapid Test	IgG or IgM	Unk	80.0 (70.9–86.8) ^b^ n = 95 AU, US	Same as above
LFIA, POC	VivaChek Biotech, VivaDiag COVID-19 IgM/IgG Rapid Test	IgM	Unk	80.0 (70.9–86.8) ^b^ n = 95 AU, US	97.8 (95.6–98.9) n = 324 AU, BE, IT, US
LFIA, POC	Xiamen Biotime Biotechnology, SARS-CoV-2 IgG/IgM Rapid Qualitative Test Kit	IgG	Na	Nd	**98.0 (94.3–99.3)** n = 150 FI, US
CLIA	Xiamen Innodx Biotech, Antibody test kit for 2019-nCoV	IgG or IgM	Na	Nd	**99.3 (98.0–99.8)** n = 430 CN(2)
LFIA, POC	Zhejiang Orient Gene Biotech, COVID-19 IgG/IgM Rapid Test Cassette	IgG	Hospitalised	96.7 (91.7–98.7) ^b^ n = 120 BE, CH, NL	97.7 (96.1–98.7) n = 568 BE, CH, FR, NL, SE
LFIA, POC	Zhejiang Orient Gene Biotech, COVID-19 IgG/IgM Rapid Test Cassette	IgG	Unk	92.4 (85.1–96.3) ^b^ n = 92 FR, SE	Same as above
LFIA, POC	Zhejiang Orient Gene Biotech, COVID-19 IgG/IgM Rapid Test Cassette	IgM	Hospitalised	86.0 (77.5–91.6) ^b^ n = 93 BE, NL	**98.4 (96.3–99.3)** n = 308 BE, FR, SE
LFIA, POC	Zhejiang Orient Gene Biotech, COVID-19 IgG/IgM Rapid Test Cassette	IgM	Unk	82.6 (73.6–89.0) ^b,c^ n = 92 FR, SE	Same as above
LFIA, POC	Zhuhai Livzon Pharmaceutical Group, Diagnostic Kit for IgM / IgG Antibody to Coronavirus (SARS-CoV-2) (Lateral Flow)	IgG	Hospitalised	86.4 (80.3–90.9) ^b^ n = 162 CN(2), FR	**98.0 (94.3–99.3)** n = 150 CN, FR, US
LFIA, POC	Zhuhai Livzon Pharmaceutical Group, Diagnostic Kit for IgM / IgG Antibody to Coronavirus (SARS-CoV-2) (Lateral Flow)	IgM	Hospitalised	75.9 (68.8–81.9) ^b^ n = 162 CN(2), FR	**99.3 (96.3–99.9)** n = 150 CN, FR, US

Ab: antibody; AT: Austria; AU: Australia; BE: Belgium; BR: Brazil; CA: Canada; CH: Switzerland; CLIA: chemiluminescence assay; CN: China; COVID-19: coronavirus disease; DE: Germany; DK: Denmark; ELISA: enzyme-linked immunosorbent assay; ES: Spain; FI: Finland; FR: France; GR: Greece; IT: Italy; JP: Japan; LFIA: lateral flow immunoassay; LU: Luxembourg; Na: not applicable; Nd: not determined, either due to no data or due to data from only one country or study; NL: The Netherlands; POC: point-of-care test; SARS-CoV-2: severe acute respiratory syndrome coronavirus 2; SE: Sweden; SG: Singapore; TW: Taiwan; UK: United Kingdom; Unk: unknown or unclearly defined; US: United States.

^a^ Sensitivity and specificity values given as value (confidence interval), number of samples (n = X), list of countries (number of studies per country if > 1). Value in bold if both confidence interval width ≤ 5% and value ≥ 95% (for sensitivity) or ≥ 98% (for specificity).

^b^ Confidence interval width > 5%.

^c^ Moderate study heterogeneity (50.0 ≤ I^2^ < 75.0%).

^d^ High study heterogeneity (I^2^ ≥ 75.0%).

Only samples taken > 14 days post onset of symptoms are included, and ≤ 28 days post onset for IgM only as target. Rows are sorted alphabetically by test, target and case population.

For nucleic acid tests, results were restricted as for antibody tests (Table 3). Four tests, including one POC, had ≥ 95% positive agreement with a CI width ≤ 5%, and nine had ≥ 98% specificity. Study heterogeneity was low for all five sensitivity and all 15 specificity results with CI width ≤ 5%.

**Table 3:** Pooled positive agreement and specificity results for SARS-CoV-2 nucleic acid tests with confidence interval width ≤ 5% for either or both and based on at least two studies, up to 22 August 2020

Category	Test	Target	Case population	Positive agreement^a^	Specificity^a^
PCR	Altona Diagnostics, RealStar SARS-CoV-2 RT-PCR Kit 1.0	E	Unk	88.1 (80.4–93.1) ^b^ n = 101 CH, FR, NL, US	**100.0 (96.7–100.0)** n = 112 CH, NL
PCR	Altona Diagnostics, RealStar SARS-CoV-2 RT-PCR Kit 1.0	S	Unk	87.1 (79.2–92.3) ^b^ n = 101 CH, FR, NL, US	**100.0 (96.7–100.0)** n = 112 CH, NL
PCR	Altona Diagnostics, RealStar SARS-CoV-2 RT-PCR Kit 1.0	S or E	Unk	81.6 (75.8–86.3) ^b,c^ n = 207 FR(3), NL	**100.0 (98.4–100.0)** n = 237 FR, NL, UK
PCR	AusDiagnostics, Coronavirus Typing Assay	ORF1ab	Na	Nd	**100.0 (98.5–100.0)** n = 254 AU, UK
PCR	BGI, Real-time fluorescent RT-PCR kit for detecting 2019 nCoV	ORF1ab	Unk	93.8 (88.7–96.7) ^b^ n = 146 CH, JP, NL, PL	**99.1 (95.1–99.8)** n = 112 CH, NL
PCR, POC	Cepheid, GeneXpert Xpert Xpress SARS-CoV-2	E or N	Unk	**98.8 (97.3–99.5)** n = 427 BE, CH, CY, DE, FI, FR, NL, SE, US(5)	100.0 (82.4–100.0) ^b^ n = 18 BE, CH, SE
PCR	CerTest Biotec, VIASURE SARS-CoV-2 Real Time PCR Detection Kit	N	Unk	96.8 (89.1–99.1) ^b,c^ n = 63 CH, NL	**100.0 (96.7–100.0)** n = 112 CH, NL
PCR	CerTest Biotec, VIASURE SARS-CoV-2 Real Time PCR Detection Kit	ORF1ab	Unk	93.7 (84.8–97.5) ^b,d^ n = 63 CH, NL	**100.0 (96.7–100.0)** n = 112 CH, NL
PCR	CerTest Biotec, VIASURE SARS-CoV-2 Real Time PCR Detection Kit	ORF1ab or N	Na	Nd	**100.0 (98.2–100.0)** n = 207 NL, UK
PCR	DiaSorin, Simplexa COVID-19 Direct RT-PCR Kit	ORF1ab or S	Unk	**97.8 (94.4–99.1)** n = 180 US(3)	Nd
PCR	Hologic, SARS-CoV-2 Assay (Panther Fusion System)	ORF1ab	Unk	**98.3 (96.8–99.1)** n = 525 FR, US(6)	Nd
PCR	KH Medical, RADI COVID-19 Detection Kit and RADI COVID-19 Triple Detection Kit	RdRP	Unk	96.8 (89.1–99.1) ^b,c^ n = 63 CH, NL	**100.0 (96.7–100.0)** n = 112 CH, NL
PCR	KH Medical, RADI COVID-19 Detection Kit and RADI COVID-19 Triple Detection Kit	S	Unk	98.4 (91.5–99.7) ^b^ n = 63 CH, NL	**100.0 (96.7–100.0)** n = 112 CH, NL
PCR	Primerdesign, genesig Real-Time PCR CoVID-19 kit	RdRP	Unk	95.3 (89.4–98.0) ^b,c^ n = 106 CH, NL, PL	**100.0 (98.8–100.0)** n = 307 CH, NL, UK
PCR	R-Biopharm, Ridagene SARS-CoV2	E	Unk	100.0 (94.3–100.0) ^b^ n = 63 CH, NL	**100.0 (96.7–100.0)** n = 112 CH, NL
PCR	Roche, COBAS SARS-CoV-2 test	ORF1ab or E	Unk	**98.8 (97.9–99.3)** n = 1,125 AT, CH, DE, FR, SI, US(5)	100.0 (90.8–100.0) ^b^ n = 38 CH, FR
PCR	Seegene, Allplex 2019-nCoV assay	E	Unk	85.0 (75.6–91.2) ^b,d^ n = 80 CH, FR, NL	**100.0 (96.7–100.0)** n = 112 CH, NL
PCR	Seegene, Allplex 2019-nCoV assay	RdRP	Unk	91.3 (83.0–95.7) ^b,c^ n = 80 CH, FR, NL	**100.0 (96.7–100.0)** n = 112 CH, NL
PCR	Tibmolbiol, SARS-CoV (COVID19) E-gene	E	Unk	100.0 (94.4–100.0) ^b^ n = 65 CH, UK	**100.0 (98.5–100.0)** n = 250 CH, UK

AT: Austria; AU: Australia; BE: Belgium; CH: Switzerland; COVID-19: coronavirus disease; CY: Cyprus; DE: Denmark; E: envelope gene; FI: Finland; FR: France; JP: Japan; N: nucleoprotein gene; Na: not applicable; Nd: not determined, either because there were no data or because there were data from only one country or study; NL: The Netherlands; PL: Poland; S: spike gene; SARS-CoV-2: severe acute respiratory syndrome coronavirus 2; SE: Sweden; SI: Slovenia; UK: United Kingdom; Unk: unknown or unclearly defined; US: United States.

^a^ Positive agreement and specificity values given as value (confidence interval), number of samples (n = X), list of countries (number of studies per country if > 1). Value in bold if both confidence interval width ≤ 5% and value ≥ 95% (for positive agreement) or ≥ 98% (for specificity).

^b^ Confidence interval width > 5%.

^c^ Moderate study heterogeneity (50.0 ≤ I^2^ < 75.0%).

^d^ High study heterogeneity (I^2^ ≥ 75.0%).

Rows are sorted alphabetically by test, target and case population.

**Figure 2:**
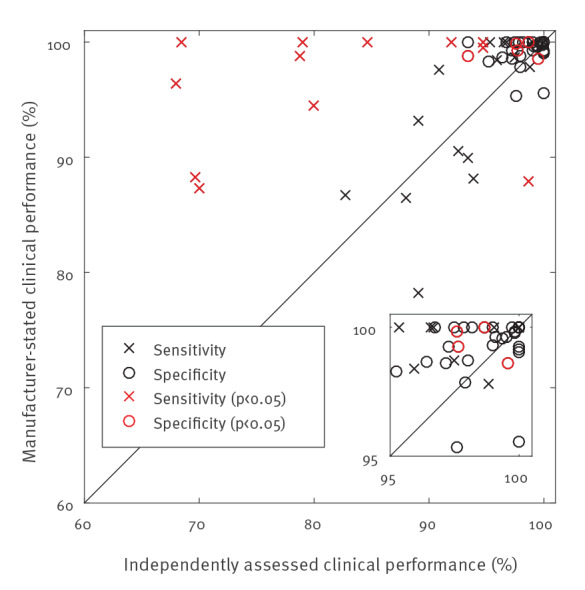
Independently assessed vs manufacturer-reported clinical sensitivity and specificity per SARS-CoV-2 test, up to 22 August 2020 (n = 55)

The correlation between independently assessed clinical performance results and manufacturer-reported results is shown in Figure 2. The manufacturer-reported documents are listed in Supplementary Table S2. Only independently assessed results with CI width ≤ 5% are included. A total of 11 of 32 sensitivity and four of 33 specificity results reported by the manufacturer were significantly larger (p < 0.05).

SARS-CoV-2: severe acute respiratory syndrome coronavirus 2.

Significantly different (p < 0.05) results are highlighted. Independently assessed results limited to those with 95% confidence interval width ≤ 5%. The inset expands the 95–100% region.

## Discussion

This review presents a comprehensive independent overview of clinical performance of commercially available nucleic acid and antibody tests 5 months into the COVID-19 pandemic. A substantial amount of previously unpublished data from European countries are included as well. By August 2020, there are numerous commercial tests for which sufficient performance data are available to allow calculation of clinical sensitivity or positive agreement, and specificity with narrow confidence interval ranges. It is reassuring that the clinical performance of several nucleic acid and antibody tests exceeded the minimum performance criteria. As time progresses, the list of tests with sufficient available performance data is expected to grow.

At the same time, the available evidence for point-of-care nucleic acid and antigen tests remains scarce, even though these tests can have substantial practical advantages for e.g. screening. We therefore recommend more emphasis on the validation of these tests, including as part of a testing algorithm, whereby the sensitivity and specificity of taking two tests with a number of days in between is assessed, and which can for example be useful to reduce the duration of a quarantine period.

The comparison between the independently assessed clinical performance data and manufacturer-reported clinical performance revealed that in particular sensitivity is frequently (34.4% of the cases in this study) significantly overestimated by the manufacturer. At a minimum, this emphasises that such independent assessments are clearly necessary. In the longer term, an explicit and proactive regulatory mechanism in Europe to compare available independently generated evidence on these tests against the manufacturer-reported values, coupled with appropriate regulatory action, would be useful. This could also be rewarding towards those manufacturers that do provide robust estimates of their product’s performance. The new in vitro diagnostic medical devices Regulation (EU) 2017/746 (IVDR), which will enter into force in May 2022, will impose more stringent requirements on clinical performance studies done by manufacturers. In addition, the IVDR will also regulate the use of lab-developed tests such as the in-house PCR tests developed for COVID-19 [25]. Because of the COVID-19 pandemic, the European Commission has recently proposed to modify the roll-out [26].

Limitations of our article include that most of the included studies had a substantial risk of bias in the sample selection, especially for the sensitivity panel, as established also in the assessments performed in the systematic reviews that we used as a source. Results were mainly based on hospitalised cases or poorly defined populations, whereas the population of interest often consists of symptomatic cases in general, or even asymptomatic cases, and differences in performance may exist depending on disease severity. Performance also varies depending on the type of specimen used, and our study design allowed for the inclusion of multiple specimen types in accordance with the instructions for use. This reflected to some extent clinical practice, but is also a contributing factor to study heterogeneity that we did not address here. Similarly, the pre-analytical steps such as RNA extraction can have a substantial effect on performance. These are often not specified in detail or several processes may be allowed according to the instructions for use, which can have contributed to study heterogeneity. While this review addresses a pressing need for actionable clinical performance data, ideally, the clinical performance should be assessed through prospective studies or clinical trials with a guaranteed unbiased sample selection for a clearly defined target population and intended use of the test. Given the difficulty of assessing and extracting the data from individual studies in a coherent way, we recommend that the Standard for Reporting of Diagnostic Accuracy Studies (STARD) should also be followed when publishing the results [27].

In this context, the selection of the reference test is particularly important with respect to reference negative samples. As described in some of the assessed studies, it should be avoided that index test results are considered as false positives while the samples are from actual cases; for this reason we excluded nucleic acid-negative samples from suspected COVID-19 patients altogether. We therefore expect little bias in the specificity results, except potentially from under- or overrepresentation of confounders. This is especially relevant for seroprevalence studies where, in a low-prevalence situation, in particular the specificity of the test needs to be well defined and high. On the other hand, sensitivity results using a nucleic acid test as reference should be interpreted with caution because the positive samples may exclude some actual cases.

Possibilities to improve the reference test can include testing - potentially only the false positives - with a second reference nucleic acid test preferably targeting different genes, testing more than one sample from the same patient including for antibodies at a later time point, testing samples from both upper and lower respiratory tracts, and sequencing the sample. The handling of intermediate index test results is an issue that needs to be described in studies and in general, these should be considered as positive results rather than as negatives or excluding them from the validation, since in clinical practice they would normally require further follow-up to confirm the positivity of the sample. Finally, the quality of the execution of the tests is also an important factor. For non-point-of-care tests, external quality assessment exercises using well validated standard reference materials remain a critical tool to detect and address such issues.

## Conclusion

Given the study limitations, the authors and organisations contributing to this study in no way recommend the use of the listed commercial tests over other not listed commercial or in-house tests. The clinical performance of tests may also change over time as the virus population evolves. We recommend, however, continuous monitoring of clinical performance both in Europe and globally, which is key for reliable monitoring of the pandemic and which will also support vaccine and antiviral development. These results should be shared publicly in a timely manner. 

## European COVID-19 microbiological laboratories group

Marjan Van Esbroeck: Institute of Tropical Medicine, Antwerpen, Belgium

Pieter Vermeersch: Clinical Department of Laboratory Medicine and National Reference Center for Respiratory Pathogens, University Hospitals Leuven, Leuven, Belgium

Kurt Beuselinck: Clinical Department of Laboratory Medicine and National Reference Center for Respiratory Pathogens, University Hospitals Leuven, Leuven, Belgium

Christos Karagiannis: Nicosia General Hospital, Cyprus

Merit Melin: Department of Health Protection, Expert Microbiology Unit, Finnish Institute for Health and Welfare (THL), Helsinki, Finland

Nina Ekström: Department of Health Protection, Expert Microbiology Unit, Finnish Institute for Health and Welfare (THL), Helsinki, Finland

Iris Erlund: Department of Government Services, Finnish Institute for Health and Welfare (THL), Helsinki, Finland

Terhi Vihervaara: Department of Government Services, Finnish Institute for Health and Welfare (THL), Helsinki, Finland

Vanessa Escuret: Laboratoire de Virologie des HCL, Institut des Agents Infectieux, CNR des virus à transmission respiratoire (dont la grippe), Groupement Hospitalier Nord, Lyon, France

Emilie Frobert: Laboratoire de Virologie des HCL, Institut des Agents Infectieux, CNR des virus à transmission respiratoire (dont la grippe), Groupement Hospitalier Nord, Lyon, France

Alexandre Gaymard: Laboratoire de Virologie des HCL, Institut des Agents Infectieux, CNR des virus à transmission respiratoire (dont la grippe), Groupement Hospitalier Nord, Lyon, France

Andreas Mentis: Hellenic Pasteur Institute, Athens, Greece

Stavroula Lampropoulou: Hellenic Pasteur Institute, Athens, Greece

Ivan-Christian Kurolt: Research unit, University Hospital for Infectious Diseases “Dr. Fran Mihaljević”, Zagreb, Croatia

Tamir Abdelrahman: Department of Microbiology, Laboratoire national de santé, Luxembourg

Trung Nguyen: Department of Microbiology, Laboratoire national de santé, Luxembourg

Guillaume Fournier: Department of Microbiology, Laboratoire national de santé, Luxembourg

Chantal B.E.M. Reusken: Centre for Infectious Disease Control, National Institute for Public Health and the Environment, The Netherlands

Maaike J.C. van den Beld: Centre for Infectious Disease Control, National Institute for Public Health and the Environment, The Netherlands

Janette Rahamat-Langendoen MD PhD: Department of Medical Microbiology, Radboud University Medical Center, Nijmegen

Marjolijn C.A. Wegdam-Blans: Department of Medical Microbiology, PAMM, Veldhoven, The Netherlands

Jeroen H. T. Tjhie: Department of Medical Microbiology, PAMM, Veldhoven, The Netherlands

Peter Croughs: Department of Medical Microbiology and Infectious Diseases, Erasmus Medical Center, Rotterdam, The Netherlands

Corine H. GeurtsvanKessel: Department of Virology, Erasmus Medical Center, Rotterdam, The Netherlands

Johan Reimerink: Centre for Infectious Diseases Research, Diagnostics and Laboratory Surveillance, Centre for Infectious Disease Control, National Institute for Public Health and the Environment, The Netherlands

David S.Y. Ong: Department of Medical Microbiology and Infection Control, Franciscus Gasthuis & Vlietland, Rotterdam, The Netherlands, Department of Epidemiology, Julius Center for Health Sciences and Primary Care, University Medical Center Utrecht, Utrecht, The Netherlands

Hans G.M. Koeleman: Department of Medical Microbitherlands

Hannke Berkhout: Canisius-Wilhelmina hospital, Nijmegen, The Netherlands

Christel F.M. van der Donk: Canisius-Wilhelmina hospital, Nijmegen, The Netherlands

Menno D. de Jong MD PhD: Department of Medical Microbiology & Infection prevention, Amsterdam University Medical Centers, The Netherlands

Rens Zonneveld MD PhD: Department of Medical Microbiology, Amsterdam University Medical Center, Amsterdam, The Netherlands

Suzanne Jurriaans PhD: Department of Medical Microbiology, Amsterdam University Medical Center, Amsterdam, The Netherlands

Nathalie Van Burgel: Hagaziekenhuis, The Hague, The Netherlands

Bas B. Wintermans MD: Department of Medical Microbiology and Immunology, Admiraal de Ruyter Hospital, Vlissingen, The Netherlands

Ger T. Rijkers: Department of Medical Microbiology and Immunology, Admiraal de Ruyter Hospital, Goes, The Netherlands, Elisabeth-Tweesteden Hospital, Tilburg, The Netherlands

Jean-Luc Murk MD PhD: Elisabeth-Tweesteden Hospital, Tilburg, The Netherlands

Khoa T.D. Thai MD PhD: Unit of Medical Microbiology, Star-shl Medical Diagnostic Center, Rotterdam, The Netherlands, Department of Medical Microbiology and Infectious Diseases, Erasmus Medical Center, Rotterdam, The Netherlands

Melanie J de Graaf: Department of Medical Microbiology, University Medical Centre, Utrecht, The Netherlands, Saltro Diagnostic Centre, Utrecht, The Netherlands

Annemarie van ’t Veen: Department of Medical Microbiology, University Medical Centre, Utrecht, The Netherlands, Saltro Diagnostic Centre, Utrecht, The Netherlands

Cornelis P. Timmerman: Central Bacteriology and Serology Laboratory, Tergooi Hospital, Hilversum, The Netherlands

Annette van Corteveen-Splinter: Central Bacteriology and Serology Laboratory, Tergooi Hospital, Hilversum, The Netherlands

Felix Geeraedts: Laboratory for Medical Microbiology and Public Health, Hengelo, The Netherlands

Adrian Klak: Laboratory for Medical Microbiology and Public Health, Hengelo, The Netherlands

Maria M. Konstantinovski MD: Reinier Haga Medical Diagnostic Centre, Delft, The Nederlands

Manou R. Batstra: Reinier Haga Medical Diagnostic Centre, Delft, The Nederlands

K. A. Heemstra MD PhD: Alrijne Zorggroep, Leiderdorp, The Netherlands

Jos J. Kerremans MD PhD: Alrijne Zorggroep, Leiderdorp, The Netherlands

Inge H. M. van Loo: Department of Medical Microbiology, Maastricht University Medical Center, The Netherlands, Care and Public Health Research Institute, Maastricht University

Paul H. M. Savelkoul: Department of Medical Microbiology, Maastricht University Medical Center, The Netherlands, Care and Public Health Research Institute, Maastricht University

Johan Kissing: Department of Medical Microbiology and Infection prevention, Gelre Hospitals, Apeldoorn, The Netherlands

Paul Martijn den Reijer: Department of Medical Microbiology and Infection prevention, Gelre Hospitals, Apeldoorn, The Netherlands

Anne Russcher: Department of Medical Microbiology, Medical Meander Center, Amersfoort, The Netherlands

Moniek Heusinkveld PhD: Department of Medical Microbiology, Hospital Gelderse Vallei, Ede, The Netherlands

Ellen van Lochem: Department of Medical Microbiology and Immunology, Hospital Rijnstate, The Netherlands

Steven F. T. Thijsen: Medical Microbiology and Immunology, Diakonessen Hospital, Utrecht, The Netherlands

Michiel Heron: Medical Microbiology and Immunology, Diakonessen Hospital, Utrecht, The Netherlands

Susanne P. Stoof MD PhD: Department of Medical Microbiology, Comicro, Hoorn, The Netherlands

Sim van Gyseghem BSc: Department of Medical Microbiology, Comicro, Hoorn, The Netherlands

Sylvia B. Debast MD PhD: Laboratory of Clinical Microbiology and Infectious Diseases, Isala Hospital, Zwolle, The Netherlands

Claudy Oliveira dos Santos MD: Laboratory of Clinical Microbiology and Infectious Diseases, Isala Hospital, Zwolle, The Netherlands

Bjorn L. Herpers MD PhD: Regional Public Health Laboratory Kennemerland, The Netherlands

Theo Mank PhD: Regional Public Health Laboratory Kennemerland, The Netherlands

Kin Ki Jim: Department of Medical Microbiology and Infection Control, Jeroen Bosch Hospital, ’s-Hertogenbosch, The Netherlands, Department of Medical Microbiology and Infection Prevention, Amsterdam University Medical Centers, Amsterdam institute for Infection and Immunity, Amsterdam, The Netherlands

Peter C. Wever: Department of Medical Microbiology and Infection Control, Jeroen Bosch Hospital, ’s-Hertogenbosch, The Netherlands

Jutte J.C. de Vries: Department of Medical Microbiology, Leiden University Medical Center, Leiden, The Netherlands

Martine Hoogewerf: Department of Medical Microbiology, Northwest Hospital Group, Alkmaar, The Netherlands

Deborah J. Kaersenhout MD MSc: Atalmedial Medical Microbiology Laboratory, Amsterdam, the Netherlands

Annette M. Stemerding: Deventer Ziekenhuis, Deventer, the Netherlands

Babette C. van Hees: Deventer Ziekenhuis, Deventer, the Netherlands

Vishal Hira: Department of Medical Microbiology and Infection Prevention, Groene Hart Ziekenhuis, Gouda, the Netherlands

Anne E. Bos: Department of Medical Microbiology and Infection Prevention, Groene Hart Ziekenhuis, Gouda, the Netherlands

Leontine Mulder: Clinical Laboratory, Medlon B.V., Enschede, The Netherlands

Michiel van Rijn MD: Medical Laboratory, Ikazia Hospital, Rotterdam, The Netherlands

Aleksander Michalski: 1st Clinical Military Hospital with Outpatient Clinic, Lublin, Poland

Marta Pakieła: Voivodeship Sanitary Epidemiological Station, Warsaw, Poland

Anna Siewierska-Puchlerska: Voivodeship Sanitary Epidemiological Station, Warsaw, Poland

Jarosław Paciorek: Voivodeship Sanitary Epidemiological Station, Warsaw, Poland

Ewa Gajda: Epidemiological Response Centre of The Polish Armed Forces, Warsaw, Poland

Katarzyna Pancer PhD: Department of Virology, BSL3 Laboratory, COVID-19 NIPH-NIH-NRI team, National Institute of Public Health-National Institute of Hygiene – National Research Institute, Warsaw, Poland

Agnieszka Kołakowska-Kulesza: Department of Virology, COVID-19 NIPH-NIH-NRI team, National Institute of Public Health-National Institute of Hygiene – National Research Institute, Warsaw, Poland

Magdalena Nowakowska: Department of Bacteriology and Biocontamination Control, COVID-19 NIPH-NIH-NRI team, National Institute of Public Health-National Institute of Hygiene – National Research Institute, Warsaw, Poland

Raquel Guiomar: Instituto Nacional de Saúde Dr. Ricardo Jorge, I.P., Portugal.

Líbia Zé-Zé: Instituto Nacional de Saúde Dr. Ricardo Jorge, I.P., Portugal.

Inês Costa: Instituto Nacional de Saúde Dr. Ricardo Jorge, I.P., Portugal.

Johan Brynedal Öckinger: Department of Virology, Clinical Microbiology, Karolinska University Laboratory, Karolinska University Hospital, Stockholm, Sweden

Berit Hammas: Department of Virology, Clinical Microbiology, Karolinska University Laboratory, Karolinska University Hospital, Stockholm, Sweden

Katarina Prosenc: National Laboratory for Health, Environment and Food Slovenia, Laboratory for Public Health Virology

Nataša Berginc: National Laboratory for Health, Environment and Food Slovenia, Laboratory for Public Health Virology

## Supplementary Data

159000 Supplement1

## Supplementary Data

3430000 SupplementaryTableS4
