# Supplementary material for: Meta-analysis of the clinical performance of commercial SARS-CoV-2 nucleic acid and antibody tests up to 22 August 2020
Source: Euro Surveill. 2021 Nov 11;26(45):2001675. doi: 10.2807/1560-7917.ES.2021.26.45.2001675 (PMC8646979; doi:10.2807/1560-7917.ES.2021.26.45.2001675)
Supplement: SupplementaryTableS4 [file 20-01675_vanWALLE_SupplementaryTableS4.pdf]

Disclaimer

This supplementary material is hosted by Eurosurveillance as supporting information alongside the article "*Meta-analysis of the clinical performance of commercial SARS-CoV-2 nucleic acid and antibody tests up to 22 August 2020*" on behalf of the authors who remain responsible for the accuracy and appropriateness of the content. The same standards for ethics, copyright, attributions and permissions as for the article apply. Supplements are not edited by Eurosurveillance and the journal is not responsible for the maintenance of any links or email addresses provided therein.

Table S4: pooled diagnostic accuracy

Category	Test	Target	Metric	CasePopulation	Count	Proportion	I2	Studies_Countries	ForestPlot
Ab (LFIA, POC)	Abacus Pharma International, SARS-CoV-2 IgM/IgG AB Antibody Rapid Test (Immunochromatography)	IgG	SENS	unk	19/27	70.4 (51.5-84.1)		5; US	na
Ab (LFIA, POC)	Abacus Pharma International, SARS-CoV-2 IgM/IgG AB Antibody Rapid Test (Immunochromatography)	IgG	SPEC	na	69/70	98.6 (92.3-99.7)		5; US	na
Ab (LFIA, POC)	Abacus Pharma International, SARS-CoV-2 IgM/IgG AB Antibody Rapid Test (Immunochromatography)	IgG or IgM	SENS	unk	20/27	74.1 (55.3-86.8)		5; US	na
Ab (LFIA, POC)	Abacus Pharma International, SARS-CoV-2 IgM/IgG AB Antibody Rapid Test (Immunochromatography)	IgG or IgM	SPEC	na	69/70	98.6 (92.3-99.7)		5; US	na
Ab (LFIA, POC)	Abacus Pharma International, SARS-CoV-2 IgM/IgG AB Antibody Rapid Test (Immunochromatography)	IgM	SENS	unk	13/27	48.1 (30.7-66.0)		5; US	na
Ab (LFIA, POC)	Abacus Pharma International, SARS-CoV-2 IgM/IgG AB Antibody Rapid Test (Immunochromatography)	IgM	SPEC	na	70/70	100.0 (94.8-100.0)		5; US	na
Ab (LFIA, POC)	Abbexa, COVID-19 IgG/IgM Rapid Test Kit	IgG	SENS	unk	28/29	96.6 (82.8-99.4)		5; US	na
Ab (LFIA, POC)	Abbexa, COVID-19 IgG/IgM Rapid Test Kit	IgG	SPEC	na	67/70	95.7 (88.1-98.5)		5; US	na
Ab (LFIA, POC)	Abbexa, COVID-19 IgG/IgM Rapid Test Kit	IgG or IgM	SENS	unk	28/29	96.6 (82.8-99.4)		5; US	na
Ab (LFIA, POC)	Abbexa, COVID-19 IgG/IgM Rapid Test Kit	IgG or IgM	SPEC	na	64/70	91.4 (82.5-96.0)		5; US	na
Ab (LFIA, POC)	Abbexa, COVID-19 IgG/IgM Rapid Test Kit	IgM	SENS	unk	15/29	51.7 (34.4-68.6)		5; US	na
Ab (LFIA, POC)	Abbexa, COVID-19 IgG/IgM Rapid Test Kit	IgM	SPEC	na	67/70	95.7 (88.1-98.5)		5; US	na
Ab (CLIA)	Abbott, SARS-CoV-2 IgG assay on Alinity	IgG	SENS	unk	133/150	88.7 (82.6-92.8)		83; DK	na
Ab (CLIA)	Abbott, SARS-CoV-2 IgG assay on Alinity	IgG	SPEC	na	669/673	99.4 (98.5-99.8)		83; DK	na
Ab (CLIA)	Abbott, SARS-CoV-2 IgG assay on Architect	IgG	SENS	mild/asymptomatic	293/331	88.5 (84.6-91.5)	40.6	3, 80, 180, 277; NL, UK(2), US	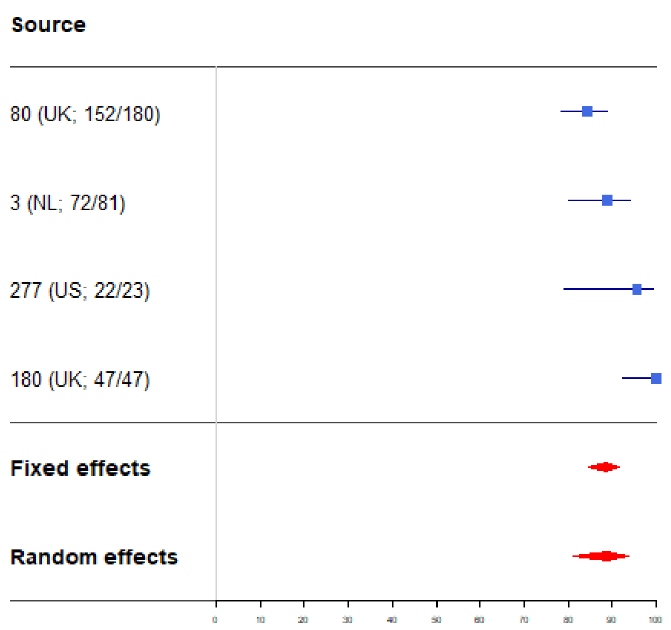
Ab (CLIA)	Abbott, SARS-CoV-2 IgG assay on Architect	IgG	SENS	hospitalised	353/368	95.9 (93.4-97.5)	0.0	3, 35, 44, 80, 217, 242, 277; BE, CA, NL, UK, US(3)	

									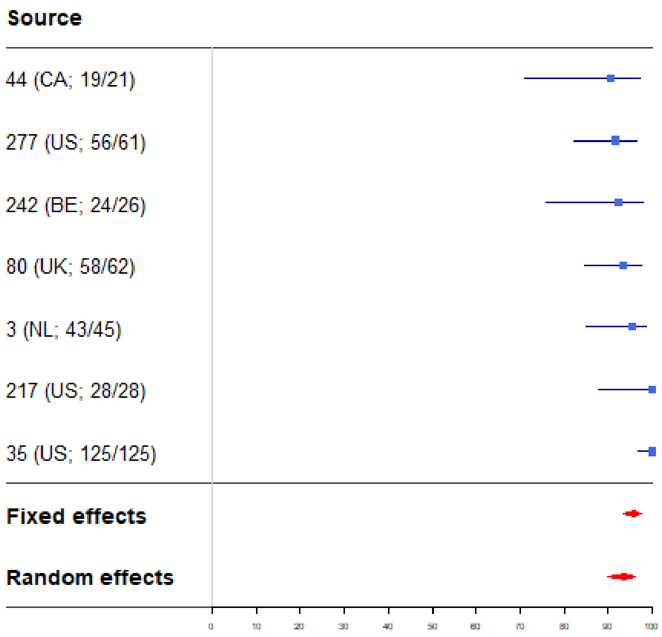
Ab (CLIA)	Abbott, SARS-CoV-2 IgG assay on Architect	IgG	SENS	unk	1225/1332	92.0 (90.4-93.3)	33.0	1, 5, 6, 16, 46, 57, 65, 81, 83, 208, 224, 244, 255, 271, 285; AT, BE, DE, DK, FI, FR(2), SE, SG, UK(2), US(4)	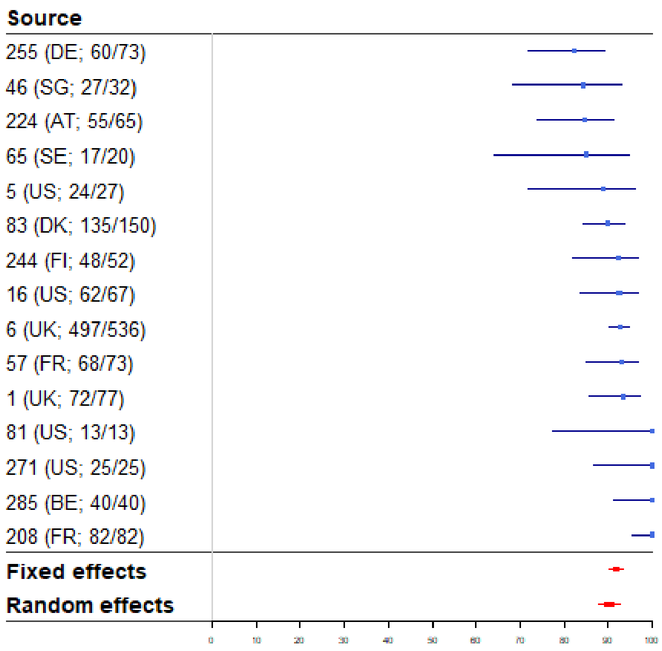
Ab (CLIA)	Abbott, SARS-CoV-2 IgG assay on Architect	IgG	SPEC	na	8203/8243	99.5 (99.3-99.6)	31.0	1, 3, 5, 6, 16, 30, 35, 44, 46, 57, 65, 80, 81, 83, 141, 145, 173, 208, 217, 224, 242, 269, 271, 274, 277, 285; AT, BE(2), CA, DE(2), DK, FI, FR(3), IT, NL, SE, SG, UK(3), US(8)	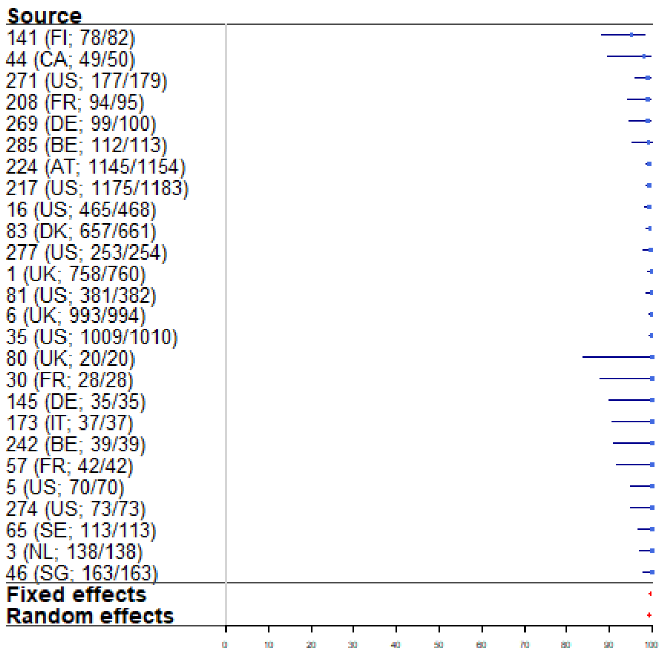
Ab (LFIA, POC)	Access Bio, CareStart COVID-19 IgM/IgG Rapid Diagnostic Test for the Detection of SARS-CoV-2 IgM/IgG Ab	IgG	SENS	unk	29/29	100.0 (88.3-100.0)		5; US	na
Ab (LFIA, POC)	Access Bio, CareStart COVID-19 IgM/IgG Rapid Diagnostic Test for the Detection of SARS-CoV-2 IgM/IgG Ab	IgG	SPEC	na	69/70	98.6 (92.3-99.7)		5; US	na
Ab (LFIA, POC)	Access Bio, CareStart COVID-19 IgM/IgG Rapid Diagnostic Test for the Detection of SARS-CoV-2 IgM/IgG Ab	IgG or IgM	SENS	unk	29/29	100.0 (88.3-100.0)		5; US	na
Ab (LFIA, POC)	Access Bio, CareStart COVID-19 IgM/IgG Rapid Diagnostic Test for the Detection of SARS-CoV-2 IgM/IgG Ab	IgG or IgM	SPEC	na	68/70	97.1 (90.2-99.2)		5; US	na
Ab (LFIA, POC)	Access Bio, CareStart COVID-19 IgM/IgG Rapid Diagnostic Test for the Detection of SARS-CoV-2 IgM/IgG Ab	IgM	SENS	unk	26/29	89.7 (73.6-96.4)		5; US	na
Ab (LFIA, POC)	Access Bio, CareStart	IgM	SPEC	na	69/70	98.6 (92.3-		5; US	na

	COVID-19 IgM/IgG Rapid Diagnostic Test for the Detection of SARS-CoV-2 IgM/IgG Ab					99.7)			
Ab (LFIA, POC)	AccuBioTech, Accu-Tell COVID-19 IgG/IgM Rapid Test Cassette	IgG	SENS	hospitalised	11/13	84.6 (57.8-95.7)		284; FR	na
Ab (LFIA, POC)	AccuBioTech, Accu-Tell COVID-19 IgG/IgM Rapid Test Cassette	IgG	SPEC	na	20/20	100.0 (83.9-100.0)		284; FR	na
Ab (LFIA, POC)	AccuBioTech, Accu-Tell COVID-19 IgG/IgM Rapid Test Cassette	IgM	SENS	hospitalised	11/12	91.7 (64.6-98.5)		284; FR	na
Ab (LFIA, POC)	AccuBioTech, Accu-Tell COVID-19 IgG/IgM Rapid Test Cassette	IgM	SPEC	na	20/20	100.0 (83.9-100.0)		284; FR	na
Ab (LFIA, POC)	Accudiagnostics, Covid-19 IgM/IgG Test Kit	IgG	SENS	unk	27/27	100.0 (87.5-100.0)		5; US	na
Ab (LFIA, POC)	Accudiagnostics, Covid-19 IgM/IgG Test Kit	IgG	SPEC	na	65/70	92.9 (84.3-96.9)		5; US	na
Ab (LFIA, POC)	Accudiagnostics, Covid-19 IgM/IgG Test Kit	IgG or IgM	SENS	unk	27/27	100.0 (87.5-100.0)		5; US	na
Ab (LFIA, POC)	Accudiagnostics, Covid-19 IgM/IgG Test Kit	IgG or IgM	SPEC	na	59/70	84.3 (74.0-91.0)		5; US	na
Ab (LFIA, POC)	Accudiagnostics, Covid-19 IgM/IgG Test Kit	IgM	SENS	unk	27/27	100.0 (87.5-100.0)		5; US	na
Ab (LFIA, POC)	Accudiagnostics, Covid-19 IgM/IgG Test Kit	IgM	SPEC	na	62/70	88.6 (79.0-94.1)		5; US	na
Ab (LFIA, POC)	Acro Biotech, COVID-19 2019-nCoV IgG/IgM Rapid Test Cassette	IgG	SENS	mild/asymptomatic	27/35	77.1 (61.0-87.9)	70.2	4, 278; NL, NO	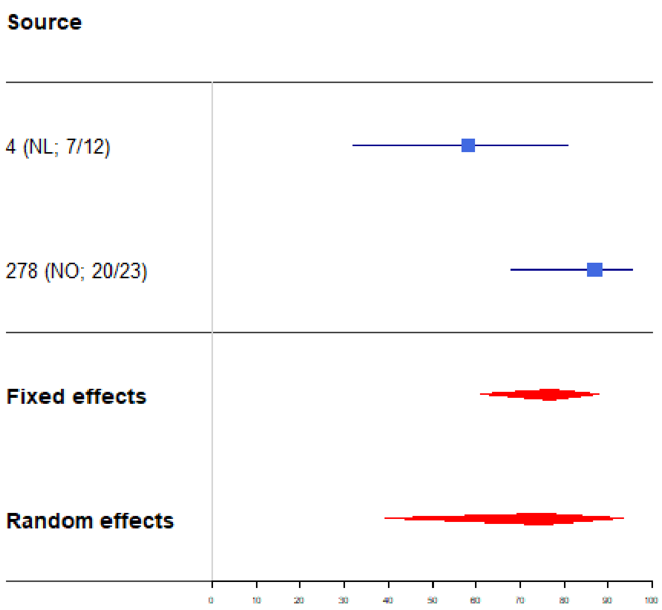
Ab (LFIA, POC)	Acro Biotech, COVID-19 2019-nCoV IgG/IgM Rapid Test Cassette	IgG	SENS	hospitalised	42/47	89.4 (77.4-95.4)	0.0	4, 284; FR, NL	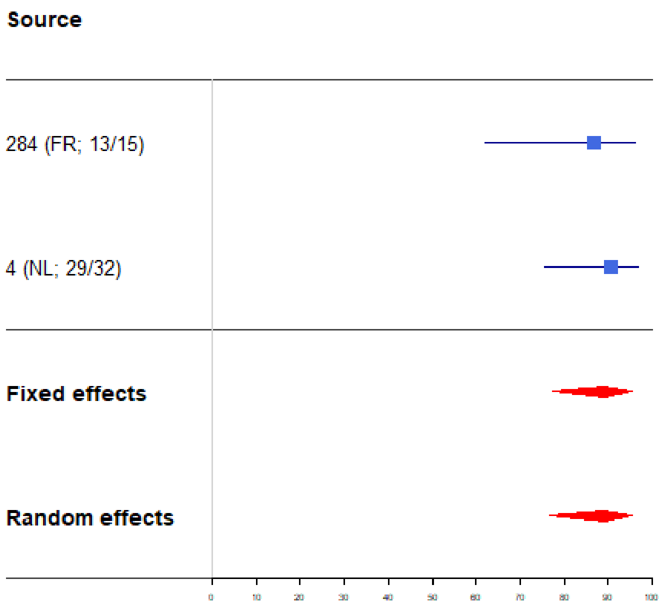
Ab (LFIA, POC)	Acro Biotech, COVID-19 2019-nCoV IgG/IgM Rapid Test Cassette	IgG	SPEC	na	130/152	85.5 (79.1-90.2)	76.6	4, 141, 284; FI, FR, NL	

									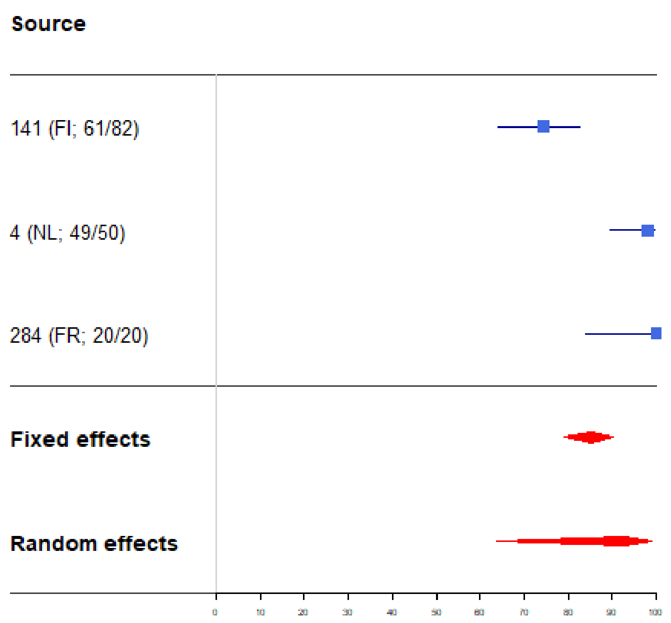
Ab (LFIA, POC)	Acro Biotech, COVID-19 2019-nCoV IgG/IgM Rapid Test Cassette	IgG or IgM	SENS	hospitalised	NaN/NaN	NaN (NaN-NaN)		150;	na
Ab (LFIA, POC)	Acro Biotech, COVID-19 2019-nCoV IgG/IgM Rapid Test Cassette	IgG or IgM	SPEC	na	12/15	80.0 (54.8-93.0)		150; DK	na
Ab (LFIA, POC)	Acro Biotech, COVID-19 2019-nCoV IgG/IgM Rapid Test Cassette	IgM	SENS	mild/asymptomatic	19/35	54.3 (38.2-69.5)	0.0	4, 278; NL, NO	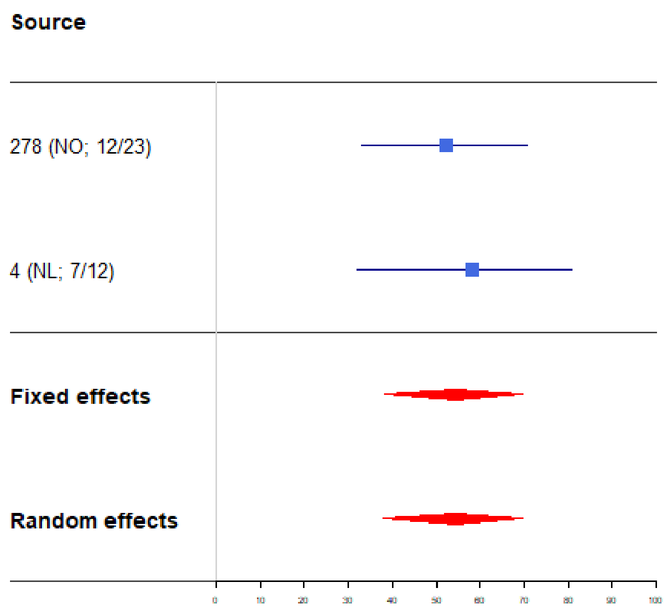
Ab (LFIA, POC)	Acro Biotech, COVID-19 2019-nCoV IgG/IgM Rapid Test Cassette	IgM	SENS	hospitalised	20/45	44.4 (30.9-58.8)	85.4	4, 284; FR, NL	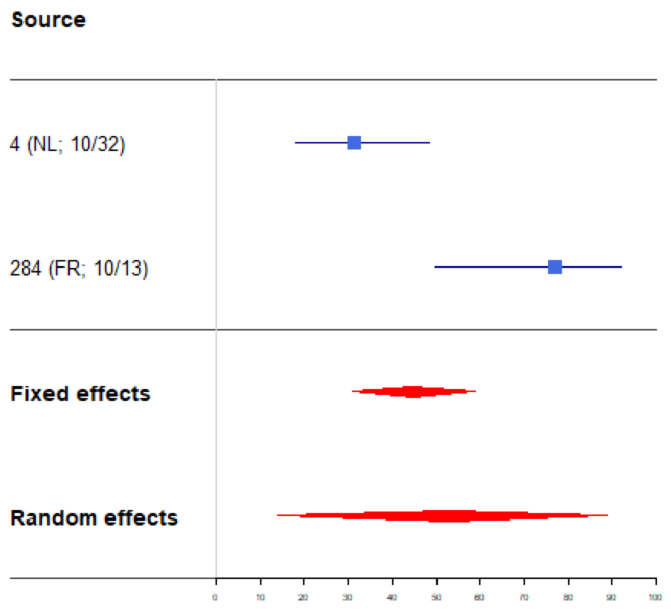
Ab (LFIA, POC)	Acro Biotech, COVID-19 2019-nCoV IgG/IgM Rapid Test Cassette	IgM	SPEC	na	77/102	75.5 (66.3-82.8)	74.8	141, 284; FI, FR	

									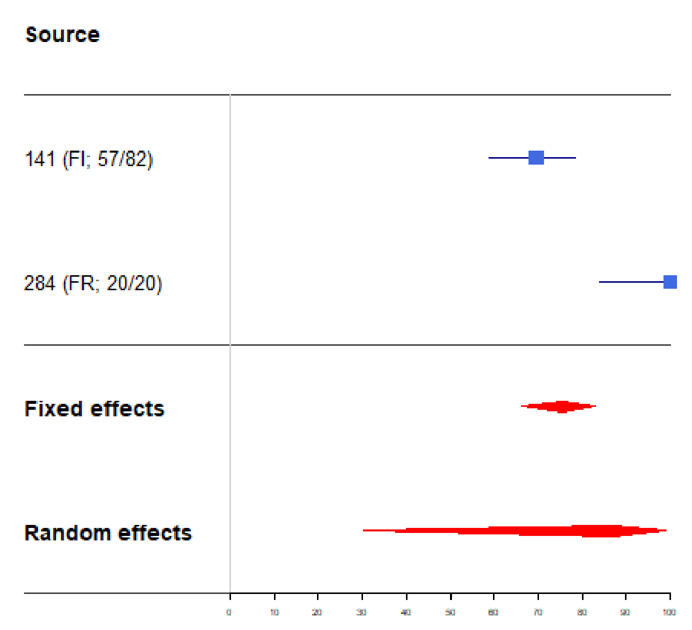
Ab (LFIA, POC)	Alfa Scientific Designs, Covid-19 IgG/IgM Antibody Test	IgG	SENS	unk	22/27	81.5 (63.3-91.8)		5; US	na
Ab (LFIA, POC)	Alfa Scientific Designs, Covid-19 IgG/IgM Antibody Test	IgG	SPEC	na	70/70	100.0 (94.8-100.0)		5; US	na
Ab (LFIA, POC)	Alfa Scientific Designs, Covid-19 IgG/IgM Antibody Test	IgG or IgM	SENS	unk	27/27	100.0 (87.5-100.0)		5; US	na
Ab (LFIA, POC)	Alfa Scientific Designs, Covid-19 IgG/IgM Antibody Test	IgG or IgM	SPEC	na	66/70	94.3 (86.2-97.8)		5; US	na
Ab (LFIA, POC)	Alfa Scientific Designs, Covid-19 IgG/IgM Antibody Test	IgM	SENS	unk	27/27	100.0 (87.5-100.0)		5; US	na
Ab (LFIA, POC)	Alfa Scientific Designs, Covid-19 IgG/IgM Antibody Test	IgM	SPEC	na	66/70	94.3 (86.2-97.8)		5; US	na
Ab (LFIA, POC)	Anhui Deep Blue Medical Technology, COVID-19 (SARS-CoV-2) IgG/IgM Antibody Test Kit	IgG	SENS	hospitalised	16/20	80.0 (58.4-91.9)		44; CA	na
Ab (LFIA, POC)	Anhui Deep Blue Medical Technology, COVID-19 (SARS-CoV-2) IgG/IgM Antibody Test Kit	IgG	SENS	unk	24/30	80.0 (62.7-90.5)		297; US	na
Ab (LFIA, POC)	Anhui Deep Blue Medical Technology, COVID-19 (SARS-CoV-2) IgG/IgM Antibody Test Kit	IgG	SPEC	na	157/158	99.4 (96.5-99.9)	0.0	44, 297; CA, US	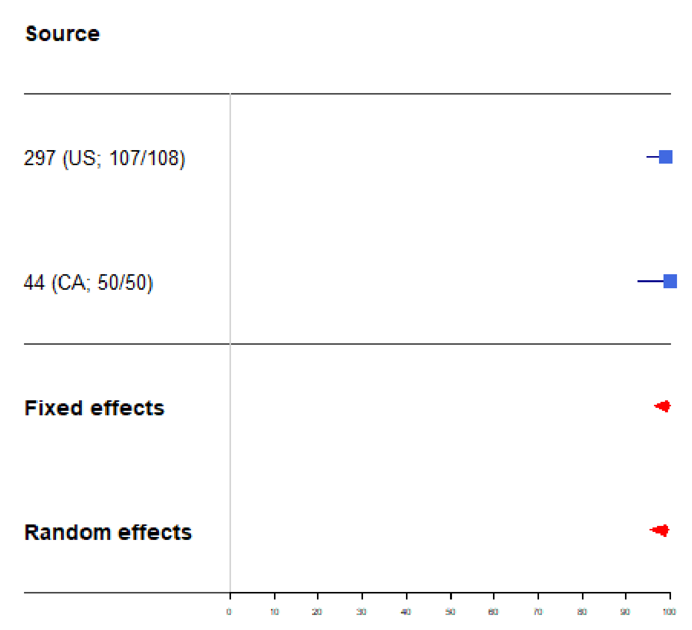
Ab (LFIA, POC)	Anhui Deep Blue Medical Technology, COVID-19 (SARS-CoV-2) IgG/IgM Antibody Test Kit	IgG or IgM	SENS	hospitalised	18/20	90.0 (69.9-97.2)		44; CA	na
Ab (LFIA, POC)	Anhui Deep Blue Medical Technology, COVID-19 (SARS-CoV-2) IgG/IgM Antibody Test Kit	IgG or IgM	SENS	unk	27/30	90.0 (74.4-96.5)		297; US	na
Ab (LFIA, POC)	Anhui Deep Blue Medical Technology, COVID-19 (SARS-CoV-2) IgG/IgM Antibody Test Kit	IgG or IgM	SPEC	na	141/158	89.2 (83.4-93.2)	75.8	44, 297; CA, US	

									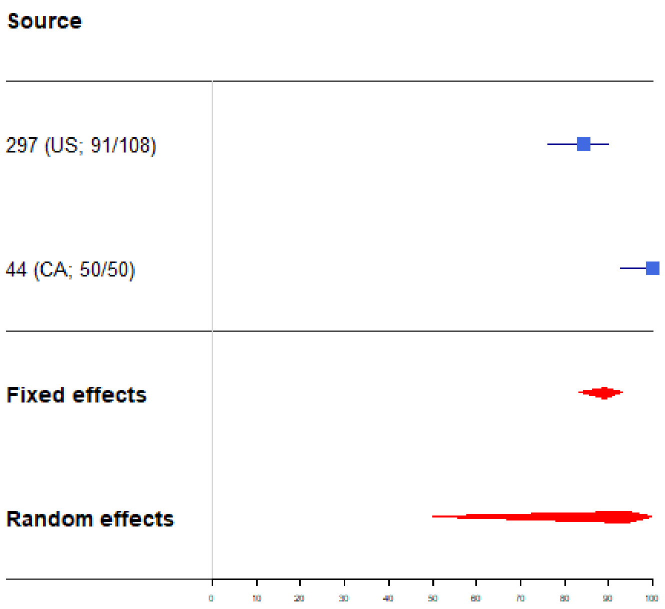
Ab (LFIA, POC)	Anhui Deep Blue Medical Technology, COVID-19 (SARS-CoV-2) IgG/IgM Antibody Test Kit	IgM	SENS	hospitalised	17/20	85.0 (64.0-94.8)		44; CA	na
Ab (LFIA, POC)	Anhui Deep Blue Medical Technology, COVID-19 (SARS-CoV-2) IgG/IgM Antibody Test Kit	IgM	SENS	unk	26/30	86.7 (70.3-94.7)		297; US	na
Ab (LFIA, POC)	Anhui Deep Blue Medical Technology, COVID-19 (SARS-CoV-2) IgG/IgM Antibody Test Kit	IgM	SPEC	na	140/158	88.6 (82.7-92.7)	77.8	44, 297; CA, US	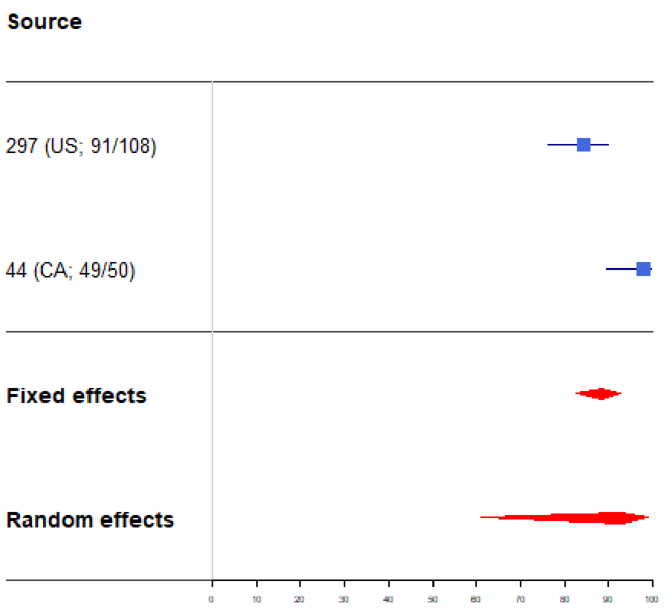
Ab (ELISA, POC)	Arbor Vita Corporation, CoVisa IgG Test	IgG	SENS	unk	22/29	75.9 (57.9-87.8)		5; US	na
Ab (ELISA, POC)	Arbor Vita Corporation, CoVisa IgG Test	IgG	SPEC	na	70/70	100.0 (94.8-100.0)		5; US	na
Ab (LFIA, POC)	Artron Laboratories, One Step Novel Coronavirus 2019 (COVID-19) IgM/IgG Antibody Test Kit	IgG	SENS	hospitalised	15/24	62.5 (42.7-78.8)		137; JP	na
Ab (LFIA, POC)	Artron Laboratories, One Step Novel Coronavirus 2019 (COVID-19) IgM/IgG Antibody Test Kit	IgG	SPEC	na	47/48	97.9 (89.1-99.6)		137; JP	na
Ab (LFIA, POC)	Artron Laboratories, One Step Novel Coronavirus 2019 (COVID-19) IgM/IgG Antibody Test Kit	IgG or IgM	SENS	hospitalised	31/32	96.9 (84.3-99.4)	0.0	137, 150; DK, JP	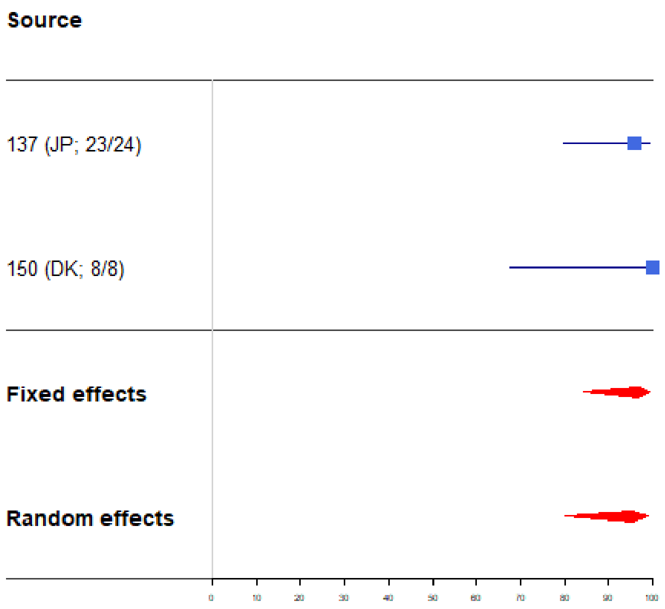
Ab (LFIA, POC)	Artron Laboratories, One Step Novel Coronavirus 2019 (COVID-19)	IgG or IgM	SPEC	na	66/66	100.0 (94.5-100.0)	0.0	137, 150; DK, JP	

	IgM/IgG Antibody Test Kit								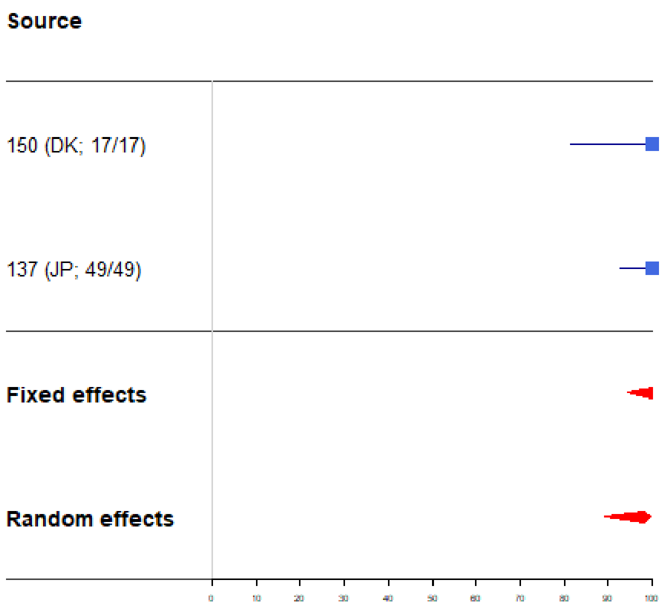
Ab (LFIA, POC)	Artron Laboratories, One Step Novel Coronavirus 2019 (COVID-19) IgM/IgG Antibody Test Kit	IgM	SENS	hospitalised	23/24	95.8 (79.8-99.3)		137; JP	na
Ab (LFIA, POC)	Artron Laboratories, One Step Novel Coronavirus 2019 (COVID-19) IgM/IgG Antibody Test Kit	IgM	SPEC	na	47/48	97.9 (89.1-99.6)		137; JP	na
Ab (LFIA, POC)	Assure Tech, COVID-19 IgG/IgM Rapid Test Device	IgG	SENS	unk	26/29	89.7 (73.6-96.4)		5; US	na
Ab (LFIA, POC)	Assure Tech, COVID-19 IgG/IgM Rapid Test Device	IgG	SPEC	na	70/70	100.0 (94.8-100.0)		5; US	na
Ab (LFIA, POC)	Assure Tech, COVID-19 IgG/IgM Rapid Test Device	IgG or IgM	SENS	hospitalised	NaN/NaN	NaN (NaN-NaN)		146;	na
Ab (LFIA, POC)	Assure Tech, COVID-19 IgG/IgM Rapid Test Device	IgG or IgM	SENS	unk	29/29	100.0 (88.3-100.0)		5; US	na
Ab (LFIA, POC)	Assure Tech, COVID-19 IgG/IgM Rapid Test Device	IgG or IgM	SPEC	na	82/83	98.8 (93.5-99.8)	0.0	5, 146; DE, US	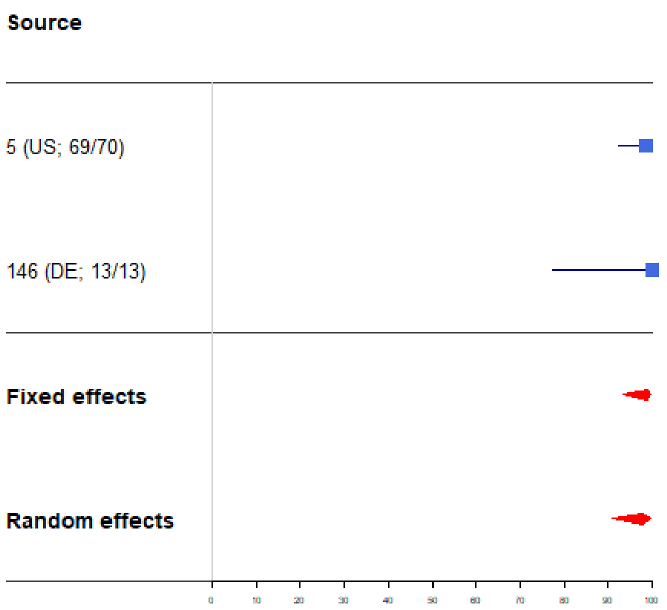
Ab (LFIA, POC)	Assure Tech, COVID-19 IgG/IgM Rapid Test Device	IgM	SENS	unk	29/29	100.0 (88.3-100.0)		5; US	na
Ab (LFIA, POC)	Assure Tech, COVID-19 IgG/IgM Rapid Test Device	IgM	SPEC	na	69/70	98.6 (92.3-99.7)		5; US	na
Ab (LFIA, POC)	Aurora Biomed, COVID-19 IgG/IgM Rapid Test	IgG	SENS	unk	20/27	74.1 (55.3-86.8)		5; US	na
Ab (LFIA, POC)	Aurora Biomed, COVID-19 IgG/IgM Rapid Test	IgG	SPEC	na	70/70	100.0 (94.8-100.0)		5; US	na
Ab (LFIA, POC)	Aurora Biomed, COVID-19 IgG/IgM Rapid Test	IgG or IgM	SENS	unk	20/27	74.1 (55.3-86.8)		5; US	na
Ab (LFIA, POC)	Aurora Biomed, COVID-19 IgG/IgM Rapid Test	IgG or IgM	SPEC	na	70/70	100.0 (94.8-100.0)		5; US	na
Ab (LFIA, POC)	Aurora Biomed, COVID-19 IgG/IgM Rapid Test	IgM	SENS	unk	17/27	63.0 (44.2-78.5)		5; US	na
Ab (LFIA, POC)	Aurora Biomed, COVID-19 IgG/IgM Rapid Test	IgM	SPEC	na	70/70	100.0 (94.8-100.0)		5; US	na
Ab (LFIA, POC)	AutoBio Diagnostics, Anti-SARS-CoV-2 Rapid Test	IgG	SENS	mild/asymptomatic	3/3	100.0 (43.9-100.0)		77; ES	na
Ab (LFIA, POC)	AutoBio Diagnostics, Anti-SARS-CoV-2 Rapid Test	IgG	SENS	hospitalised	32/32	100.0 (89.3-100.0)		77; ES	na
Ab (LFIA, POC)	AutoBio Diagnostics,	IgG	SENS	unk	27/29	93.1 (78.0-		5; US	na

	Anti-SARS-CoV-2 Rapid Test					98.1)			
Ab (LFIA, POC)	AutoBio Diagnostics, Anti-SARS-CoV-2 Rapid Test	IgG	SPEC	na	69/70	98.6 (92.3-99.7)		5; US	na
Ab (LFIA, POC)	AutoBio Diagnostics, Anti-SARS-CoV-2 Rapid Test	IgG or IgM	SENS	mild/asymptomatic	3/3	100.0 (43.9-100.0)		77; ES	na
Ab (LFIA, POC)	AutoBio Diagnostics, Anti-SARS-CoV-2 Rapid Test	IgG or IgM	SENS	hospitalised	40/40	100.0 (91.2-100.0)	0.0	77, 150; DK, ES	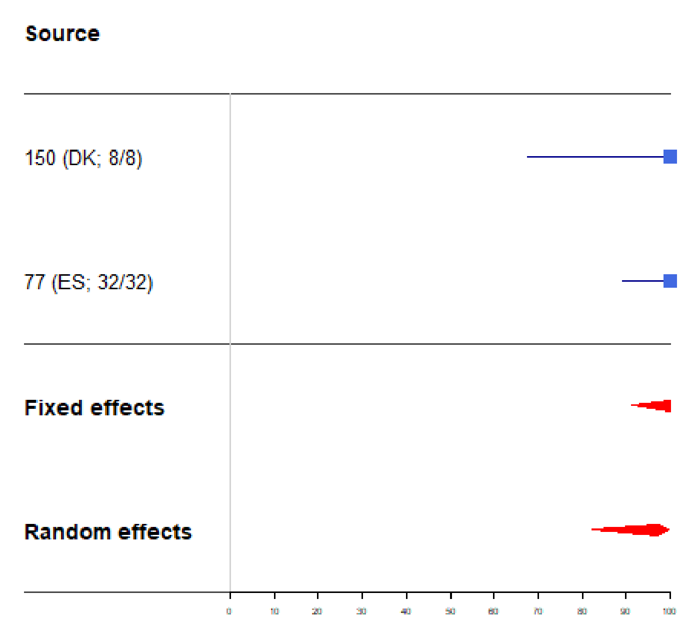
Ab (LFIA, POC)	AutoBio Diagnostics, Anti-SARS-CoV-2 Rapid Test	IgG or IgM	SENS	unk	27/29	93.1 (78.0-98.1)		5; US	na
Ab (LFIA, POC)	AutoBio Diagnostics, Anti-SARS-CoV-2 Rapid Test	IgG or IgM	SPEC	na	101/102	99.0 (94.7-99.8)	0.0	5, 150; DK, US	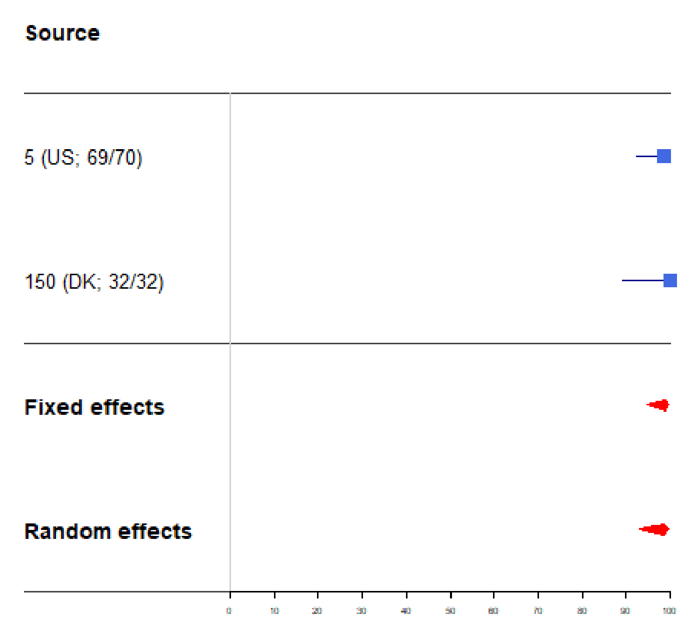
Ab (LFIA, POC)	AutoBio Diagnostics, Anti-SARS-CoV-2 Rapid Test	IgM	SENS	mild/asymptomatic	1/3	33.3 (6.1-79.2)		77; ES	na
Ab (LFIA, POC)	AutoBio Diagnostics, Anti-SARS-CoV-2 Rapid Test	IgM	SENS	hospitalised	25/32	78.1 (61.2-89.0)		77; ES	na
Ab (LFIA, POC)	AutoBio Diagnostics, Anti-SARS-CoV-2 Rapid Test	IgM	SENS	unk	14/29	48.3 (31.4-65.6)		5; US	na
Ab (LFIA, POC)	AutoBio Diagnostics, Anti-SARS-CoV-2 Rapid Test	IgM	SPEC	na	70/70	100.0 (94.8-100.0)		5; US	na
Ab (LFIA, POC)	Avioq Bio-Tech, Novel Coronavirus (2019-nCov)Antibody IgG/IgM Assay Kit (Colloidal Gold)	IgG	SPEC	na	69/72	95.8 (88.5-98.6)		177; BE	na
Ab (LFIA, POC)	Avioq Bio-Tech, Novel Coronavirus (2019-nCov)Antibody IgG/IgM Assay Kit (Colloidal Gold)	IgG or IgM	SENS	hospitalised	31/33	93.9 (80.4-98.3)		177; BE	na
Ab (LFIA, POC)	Avioq Bio-Tech, Novel Coronavirus (2019-nCov)Antibody IgG/IgM Assay Kit (Colloidal Gold)	IgG or IgM	SPEC	na	69/72	95.8 (88.5-98.6)		177; BE	na
Ab (LFIA, POC)	Avioq Bio-Tech, Novel Coronavirus (2019-nCov)Antibody IgG/IgM Assay Kit (Colloidal Gold)	IgM	SPEC	na	69/72	95.8 (88.5-98.6)		177; BE	na
Ab (LFIA, POC)	BTNX, Rapid Response COVID-19 IgG/IgM Test Cassette	IgG	SENS	hospitalised	16/20	80.0 (58.4-91.9)		44; CA	na
Ab (LFIA, POC)	BTNX, Rapid Response COVID-19 IgG/IgM Test Cassette	IgG	SPEC	na	50/50	100.0 (92.9-100.0)		44; CA	na
Ab (LFIA, POC)	BTNX, Rapid Response	IgG or	SENS	hospitalised	17/20	85.0 (64.0-		44; CA	na

	COVID-19 IgG/IgM Test Cassette	IgM				94.8)			
Ab (LFIA, POC)	BTNX, Rapid Response COVID-19 IgG/IgM Test Cassette	IgG or IgM	SPEC	na	50/50	100.0 (92.9-100.0)		44; CA	na
Ab (LFIA, POC)	BTNX, Rapid Response COVID-19 IgG/IgM Test Cassette	IgM	SENS	hospitalised	17/20	85.0 (64.0-94.8)		44; CA	na
Ab (LFIA, POC)	BTNX, Rapid Response COVID-19 IgG/IgM Test Cassette	IgM	SPEC	na	50/50	100.0 (92.9-100.0)		44; CA	na
Ab (LFIA, POC)	Beier Bioengineering Company, 2019-New Coronavirus IgG/IgM Rapid Test	IgG	SENS	hospitalised	11/14	78.6 (52.4-92.4)		73; CN	na
Ab (LFIA, POC)	Beier Bioengineering Company, 2019-New Coronavirus IgG/IgM Rapid Test	IgG	SENS	unk	NaN/NaN	NaN (NaN-NaN)		323;	na
Ab (LFIA, POC)	Beier Bioengineering Company, 2019-New Coronavirus IgG/IgM Rapid Test	IgM	SENS	hospitalised	9/14	64.3 (38.8-83.7)		73; CN	na
Ab (LFIA, POC)	Beier Bioengineering Company, 2019-New Coronavirus IgG/IgM Rapid Test	IgM	SENS	unk	NaN/NaN	NaN (NaN-NaN)		323;	na
Ab (CLIA, POC)	Beier Bioengineering Company, 2019-New Coronavirus IgM/IgG Test for MCLIA	IgG	SENS	unk	NaN/NaN	NaN (NaN-NaN)		323;	na
Ab (CLIA, POC)	Beier Bioengineering Company, 2019-New Coronavirus IgM/IgG Test for MCLIA	IgM	SENS	unk	NaN/NaN	NaN (NaN-NaN)		323;	na
Ab (LFIA, POC)	Beijing Atlas-Link, Nova COVID-19 IgG/IgM Antibody Rapid Test	IgG	SENS	unk	24/27	88.9 (71.9-96.1)		5; US	na
Ab (LFIA, POC)	Beijing Atlas-Link, Nova COVID-19 IgG/IgM Antibody Rapid Test	IgG	SPEC	na	62/70	88.6 (79.0-94.1)		5; US	na
Ab (LFIA, POC)	Beijing Atlas-Link, Nova COVID-19 IgG/IgM Antibody Rapid Test	IgG or IgM	SENS	unk	24/27	88.9 (71.9-96.1)		5; US	na
Ab (LFIA, POC)	Beijing Atlas-Link, Nova COVID-19 IgG/IgM Antibody Rapid Test	IgG or IgM	SPEC	na	62/70	88.6 (79.0-94.1)		5; US	na
Ab (LFIA, POC)	Beijing Atlas-Link, Nova COVID-19 IgG/IgM Antibody Rapid Test	IgM	SENS	unk	24/27	88.9 (71.9-96.1)		5; US	na
Ab (LFIA, POC)	Beijing Atlas-Link, Nova COVID-19 IgG/IgM Antibody Rapid Test	IgM	SPEC	na	62/70	88.6 (79.0-94.1)		5; US	na
Ab (LFIA, POC)	Beijing Diagreat Biotechnologies, 2019-nCoV IgG/IgM Antibody Rapid Test Kit	IgG	SENS	hospitalised	NaN/NaN	NaN (NaN-NaN)		264;	na
Ab (LFIA, POC)	Beijing Diagreat Biotechnologies, 2019-nCoV IgG/IgM Antibody Rapid Test Kit	IgG	SPEC	na	7/7	100.0 (64.6-100.0)		264; IT	na
Ab (ELISA)	Beijing Hotgen Biotech, Coronavirus disease (COVID-19) Antibody Test (ELISA)	IgG	SENS	hospitalised	90/100	90.0 (82.6-94.5)		158; CN	na
Ab (ELISA)	Beijing Hotgen Biotech, Coronavirus disease (COVID-19) Antibody Test (ELISA)	IgG or IgM	SENS	hospitalised	94/100	94.0 (87.5-97.2)		158; CN	na
Ab (ELISA)	Beijing Hotgen Biotech, Coronavirus disease (COVID-19) Antibody Test (ELISA)	IgM	SENS	hospitalised	93/100	93.0 (86.3-96.6)		158; CN	na
Ab (LFIA, POC)	Beijing Wantai Biological Pharmacy Enterprise, Wantai SARS-CoV-2 Ab Rapid Test Kit	IgG	SENS	hospitalised	NaN/NaN	NaN (NaN-NaN)		164;	na
Ab (LFIA, POC)	Beijing Wantai Biological Pharmacy Enterprise, Wantai SARS-CoV-2 Ab Rapid Test Kit	IgG	SPEC	na	208/209	99.5 (97.3-99.9)		164; CN	na
Ab (LFIA, POC)	Beijing Wantai Biological Pharmacy Enterprise, Wantai SARS-CoV-2 Ab Rapid Test Kit	IgG or IgM	SENS	hospitalised	NaN/NaN	NaN (NaN-NaN)		164;	na
Ab (LFIA, POC)	Beijing Wantai Biological Pharmacy Enterprise, Wantai SARS-CoV-2 Ab Rapid Test Kit	IgG or IgM	SENS	unk	87/99	87.9 (80.0-92.9)		296; AT	na
Ab (LFIA, POC)	Beijing Wantai Biological Pharmacy Enterprise, Wantai SARS-CoV-2 Ab Rapid Test Kit	IgG or IgM	SPEC	na	199/209	95.2 (91.4-97.4)		164; CN	na
Ab (LFIA, POC)	Beijing Wantai Biological Pharmacy Enterprise, Wantai SARS-CoV-2 Ab Rapid Test Kit	IgM	SENS	hospitalised	NaN/NaN	NaN (NaN-NaN)		164;	na
Ab (LFIA, POC)	Beijing Wantai Biological Pharmacy Enterprise,	IgM	SPEC	na	205/209	98.1 (95.2-99.3)		164; CN	na

	Wantai SARS-CoV-2 Ab Rapid Test Kit							
Ab (LFIA, POC)	Beijing Wantai Biological Pharmacy Enterprise, Wantai SARS-CoV-2 Ab Rapid Test Kit	total Ab	SENS	mild/asymptomatic	12/23	52.2 (33.0-70.8)		278; NO	na
Ab (LFIA, POC)	Beijing Wantai Biological Pharmacy Enterprise, Wantai SARS-CoV-2 Ab Rapid Test Kit	total Ab	SENS	hospitalised	NaN/NaN	NaN (NaN-NaN)		4;	na
Ab (LFIA, POC)	Beijing Wantai Biological Pharmacy Enterprise, Wantai SARS-CoV-2 Ab Rapid Test Kit	total Ab	SENS	unk	27/29	93.1 (78.0-98.1)		11; FR	na
Ab (LFIA, POC)	Beijing Wantai Biological Pharmacy Enterprise, Wantai SARS-CoV-2 Ab Rapid Test Kit	total Ab	SPEC	na	238/248	96.0 (92.7-97.8)	0.0	4, 11, 164; CN, FR, NL	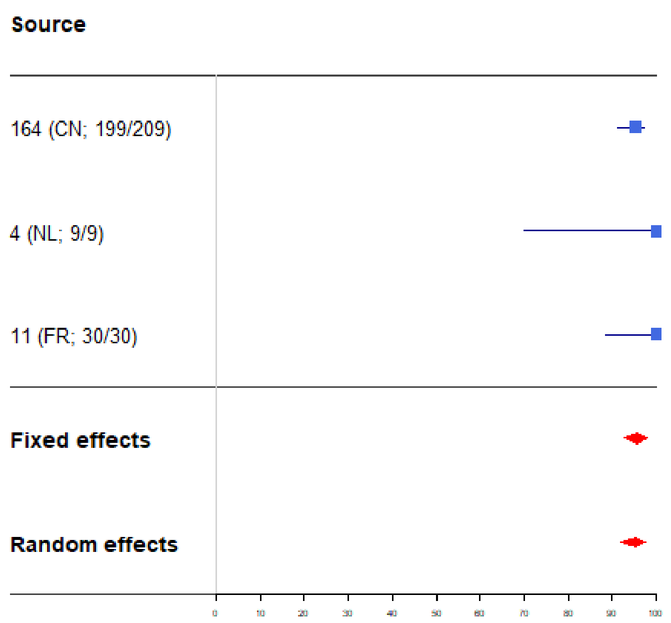
Ab (ELISA)	Beijing Wantai Biological Pharmacy Enterprise, Wantai SARS-CoV-2 IgM ELISA	IgM	SENS	mild/asymptomatic	76/84	90.5 (82.3-95.1)		3; NL	na
Ab (ELISA)	Beijing Wantai Biological Pharmacy Enterprise, Wantai SARS-CoV-2 IgM ELISA	IgM	SENS	hospitalised	181/195	92.8 (88.3-95.7)	61.0	3, 164, 326; CN(2), NL	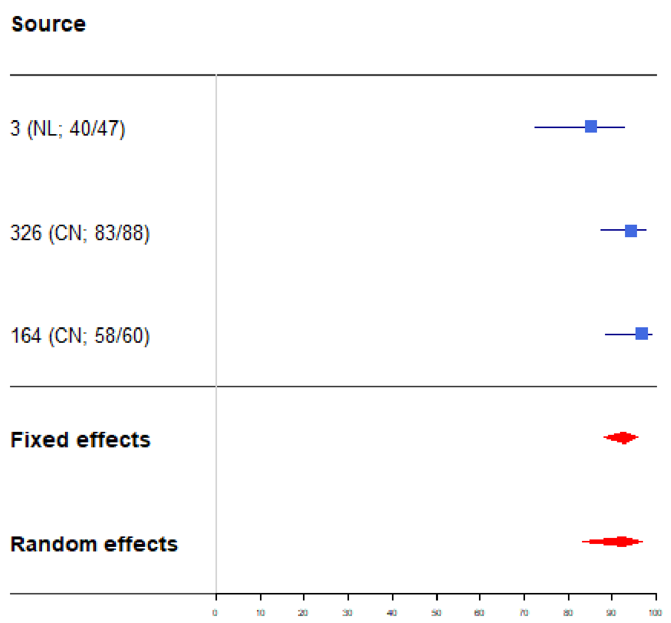
Ab (ELISA)	Beijing Wantai Biological Pharmacy Enterprise, Wantai SARS-CoV-2 IgM ELISA	IgM	SENS	unk	124/150	82.7 (75.8-87.9)		83; DK	na
Ab (ELISA)	Beijing Wantai Biological Pharmacy Enterprise, Wantai SARS-CoV-2 IgM ELISA	IgM	SPEC	na	1485/1505	98.7 (98.0-99.1)	48.0	3, 75, 83, 164, 326; CN(2), DK, NL(2)	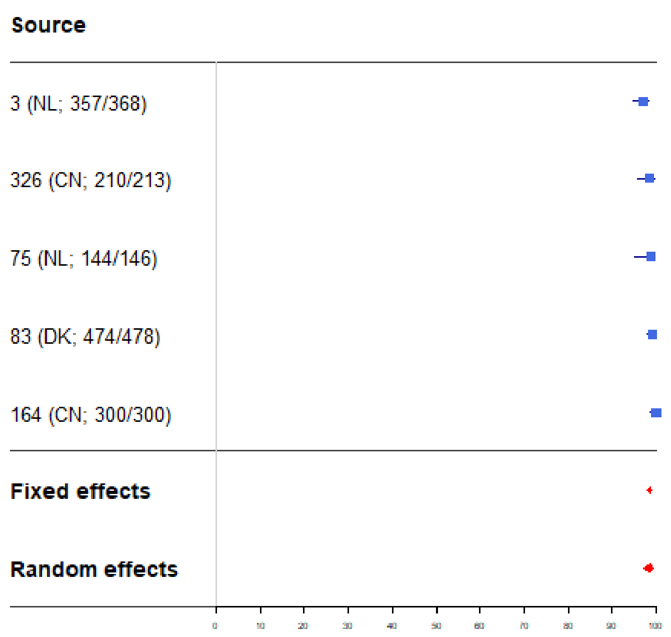
Ab (ELISA)	Beijing Wantai Biological Pharmacy Enterprise, Wantai SARS-CoV-2 total Ab ELISA	total Ab	SENS	mild/asymptomatic	237/249	95.2 (91.8-97.2)		3; NL	na

Ab (ELISA)	Beijing Wantai Biological Pharmacy Enterprise, Wantai SARS-CoV-2 total Ab ELISA	total Ab	SENS	hospitalised	588/603	97.5 (95.9-98.5)	71.2	3, 150, 164, 226, 326; CN(2), DE, DK, NL	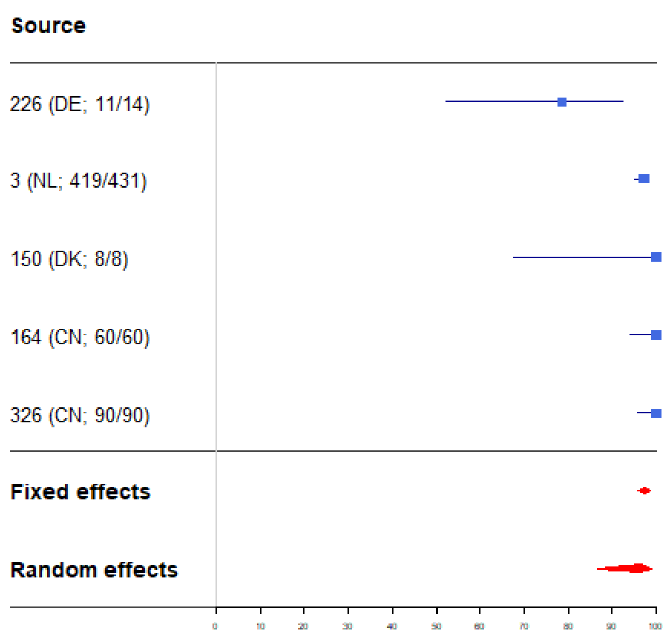
Ab (ELISA)	Beijing Wantai Biological Pharmacy Enterprise, Wantai SARS-CoV-2 total Ab ELISA	total Ab	SENS	unk	272/279	97.5 (94.9-98.8)	0.0	11, 83, 296; AT, DK, FR	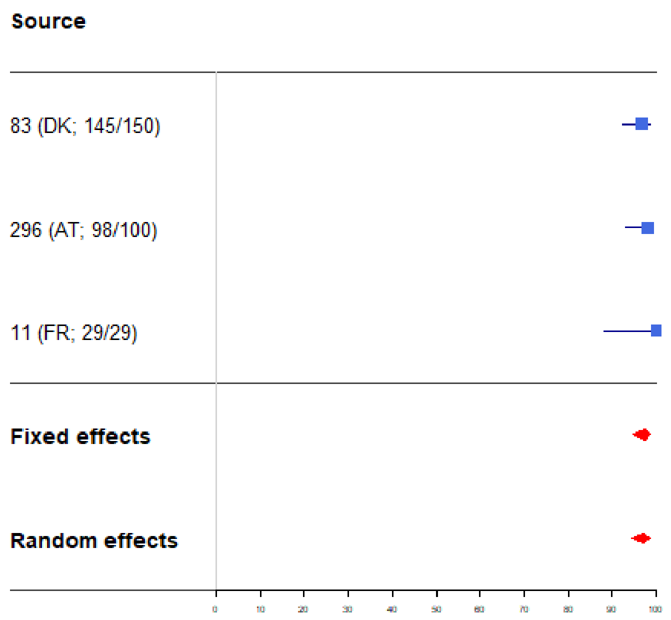
Ab (ELISA)	Beijing Wantai Biological Pharmacy Enterprise, Wantai SARS-CoV-2 total Ab ELISA	total Ab	SPEC	na	3083/3097	99.5 (99.2-99.7)	0.0	3, 11, 30, 75, 83, 150, 164, 212, 226, 326; CN(2), DE, DK(2), FR(2), NL(3)	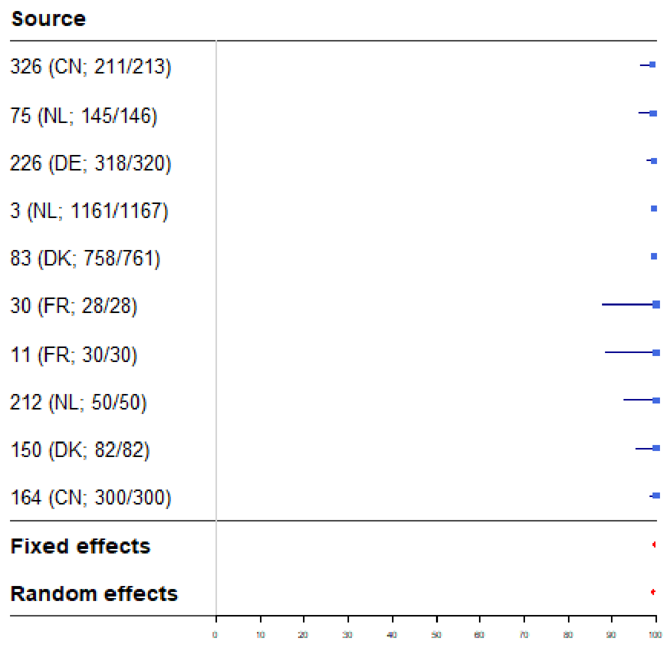
Ab (ELISA)	Bio-Rad, Platelia SARS-CoV-2 Total Ab	total Ab	SENS	mild/asymptomatic	74/83	89.2 (80.7-94.2)		3; NL	na
Ab (ELISA)	Bio-Rad, Platelia SARS-CoV-2 Total Ab	total Ab	SENS	hospitalised	36/39	92.3 (79.7-97.3)		282; BE	na
Ab (ELISA)	Bio-Rad, Platelia SARS-CoV-2 Total Ab	total Ab	SENS	unk	NaN/NaN	NaN (NaN-NaN)		245;	na
Ab (ELISA)	Bio-Rad, Platelia SARS-CoV-2 Total Ab	total Ab	SPEC	na	241/250	96.4 (93.3-98.1)	5.4	3, 30, 245, 282; BE, FR, LU, NL	

									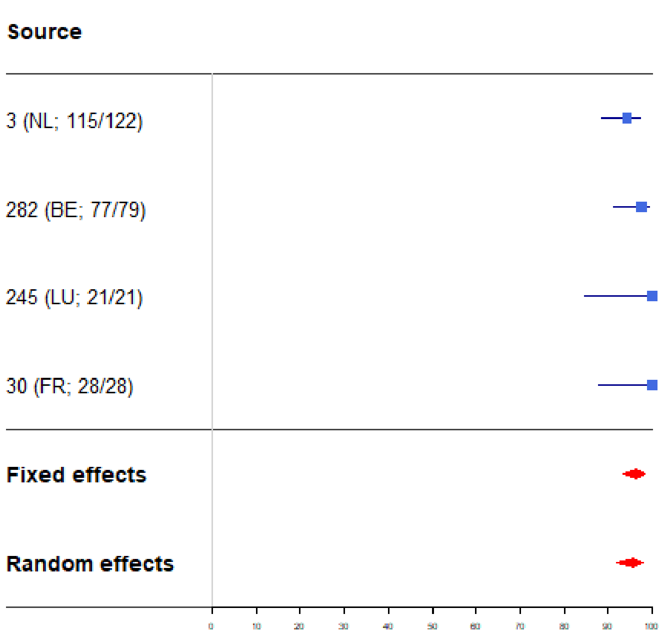
Ab (LFIA, POC)	BioMedomics, COVID-19 IgM-IgG Dual Antibody Rapid Test	IgG	SENS	unk	51/59	86.4 (75.5-93.0)	73.4	5, 297; US(2)	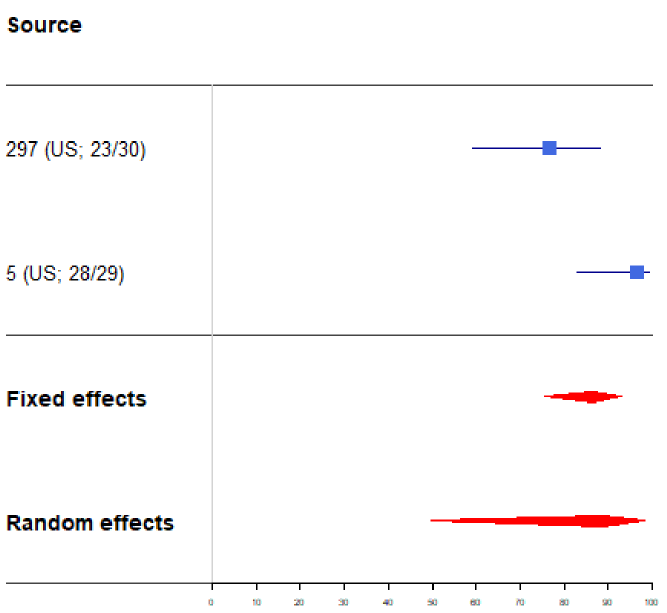
Ab (LFIA, POC)	BioMedomics, COVID-19 IgM-IgG Dual Antibody Rapid Test	IgG	SPEC	na	170/177	96.0 (92.1-98.1)	0.0	5, 297; US(2)	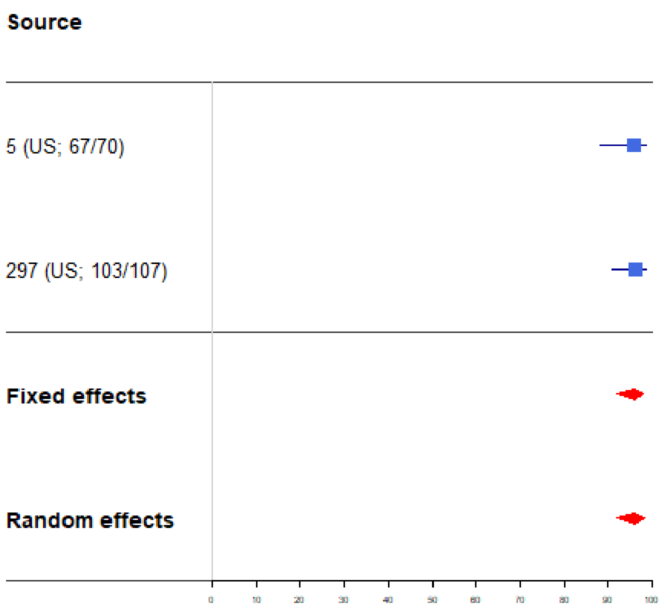
Ab (LFIA, POC)	BioMedomics, COVID-19 IgM-IgG Dual Antibody Rapid Test	IgG or IgM	SENS	unk	55/59	93.2 (83.8-97.3)	52.3	5, 297; US(2)	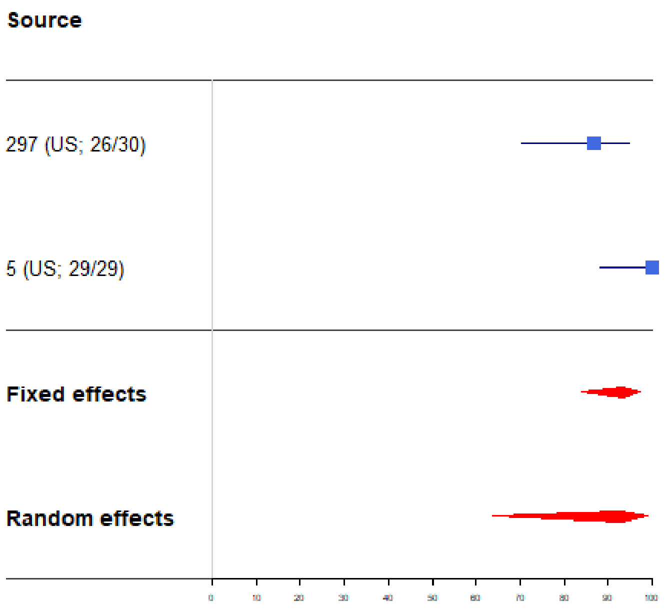
Ab (LFIA, POC)	BioMedomics, COVID-	IgG or	SPEC	na	159/177	89.8 (84.5-	58.0	5, 297; US(2)	

	19 IgM-IgG Dual Antibody Rapid Test	IgM				93.5)			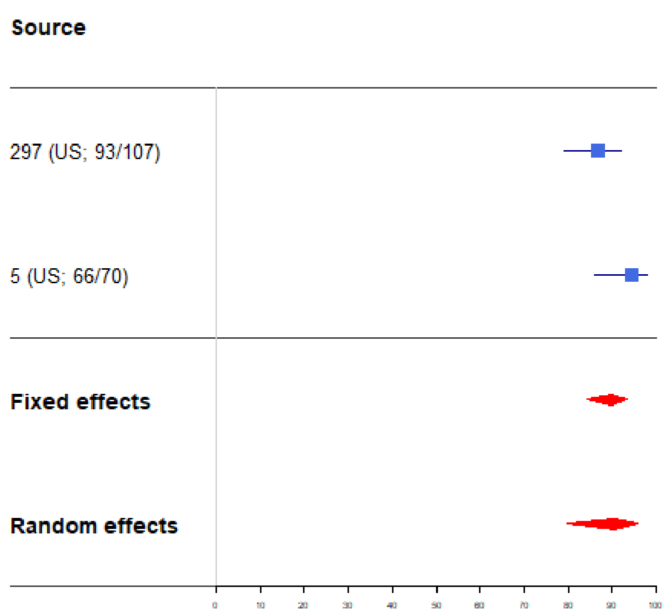
Ab (LFIA, POC)	BioMedomics, COVID-19 IgM-IgG Dual Antibody Rapid Test	IgM	SENS	unk	50/59	84.7 (73.5-91.8)	0.0	5, 297; US(2)	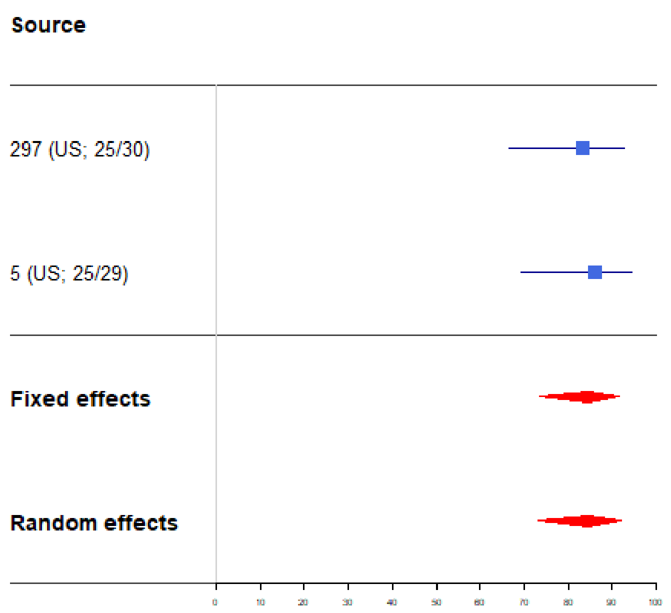
Ab (LFIA, POC)	BioMedomics, COVID-19 IgM-IgG Dual Antibody Rapid Test	IgM	SPEC	na	162/177	91.5 (86.5-94.8)	74.9	5, 297; US(2)	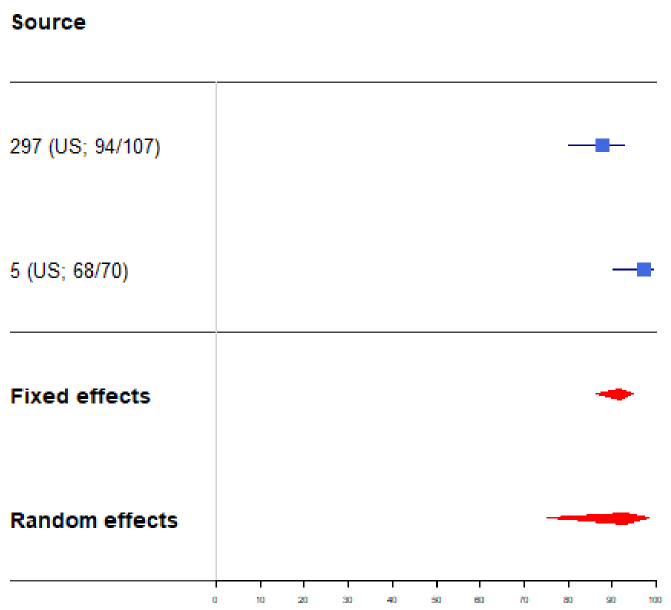
Ab (ELFA)	BioMerieux, VIDAS anti-SARS-CoV-2 IgG ELFA	IgG	SENS	unk	39/45	86.7 (73.8-93.7)		299; BE	na
Ab (ELFA)	BioMerieux, VIDAS anti-SARS-CoV-2 IgG ELFA	IgG	SPEC	na	96/96	100.0 (96.2-100.0)		299; BE	na
Ab (ELFA)	BioMerieux, VIDAS anti-SARS-CoV-2 IgM ELFA	IgM	SENS	unk	33/45	73.3 (59.0-84.0)		299; BE	na
Ab (ELFA)	BioMerieux, VIDAS anti-SARS-CoV-2 IgM ELFA	IgM	SPEC	na	96/96	100.0 (96.2-100.0)		299; BE	na
Ab (LFIA, POC)	Biolidics, 2019-nCoV IgG/IgM Antibody Detection Kit	IgG	SENS	hospitalised	18/20	90.0 (69.9-97.2)		44; CA	na
Ab (LFIA, POC)	Biolidics, 2019-nCoV IgG/IgM Antibody Detection Kit	IgG	SENS	unk	28/29	96.6 (82.8-99.4)		5; US	na
Ab (LFIA, POC)	Biolidics, 2019-nCoV IgG/IgM Antibody Detection Kit	IgG	SPEC	na	116/120	96.7 (91.7-98.7)	30.4	5, 44; CA, US	

									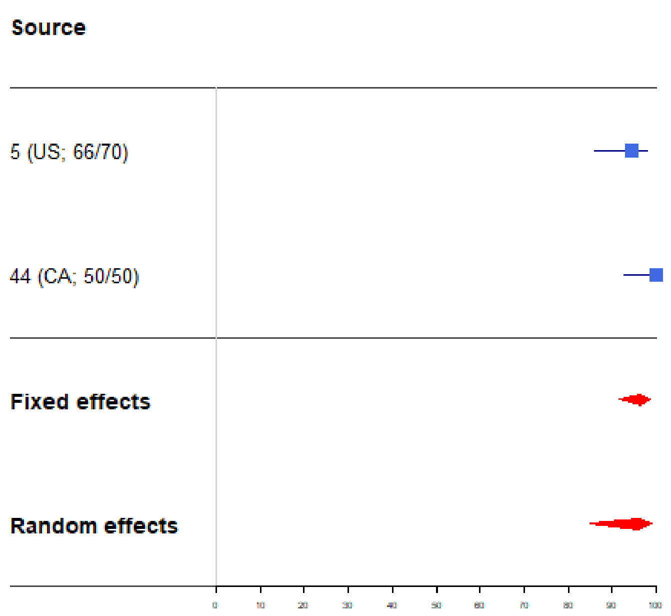
Ab (LFIA, POC)	Biolidics, 2019-nCoV IgG/IgM Antibody Detection Kit	IgG or IgM	SENS	hospitalised	18/20	90.0 (69.9-97.2)		44; CA	na
Ab (LFIA, POC)	Biolidics, 2019-nCoV IgG/IgM Antibody Detection Kit	IgG or IgM	SENS	unk	28/29	96.6 (82.8-99.4)		5; US	na
Ab (LFIA, POC)	Biolidics, 2019-nCoV IgG/IgM Antibody Detection Kit	IgG or IgM	SPEC	na	116/120	96.7 (91.7-98.7)	30.4	5, 44; CA, US	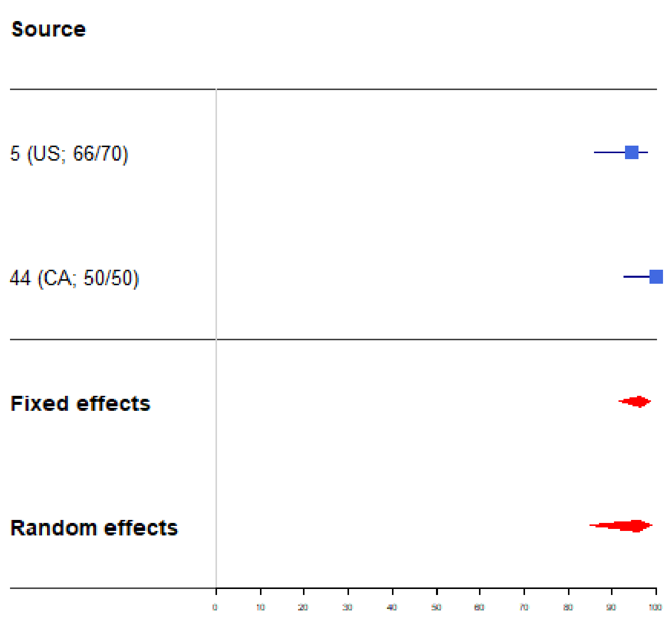
Ab (LFIA, POC)	Biolidics, 2019-nCoV IgG/IgM Antibody Detection Kit	IgM	SENS	hospitalised	6/20	30.0 (14.5-51.9)		44; CA	na
Ab (LFIA, POC)	Biolidics, 2019-nCoV IgG/IgM Antibody Detection Kit	IgM	SENS	unk	9/29	31.0 (17.3-49.2)		5; US	na
Ab (LFIA, POC)	Biolidics, 2019-nCoV IgG/IgM Antibody Detection Kit	IgM	SPEC	na	115/120	95.8 (90.6-98.2)	0.0	5, 44; CA, US	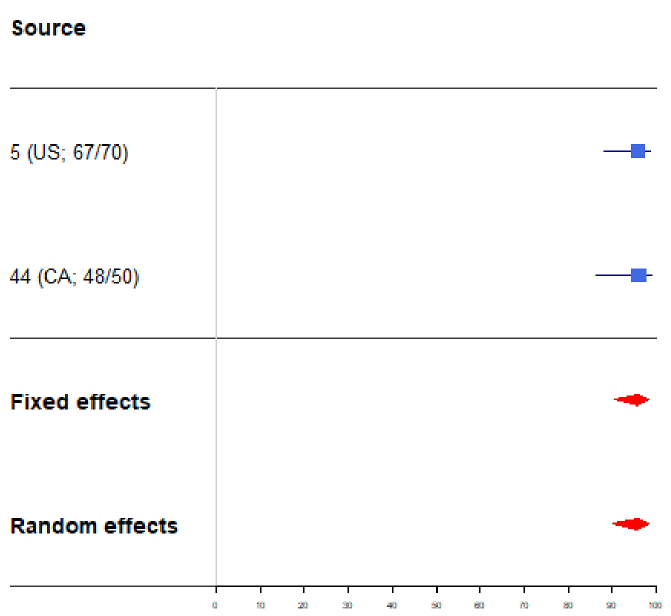
Ab (LFIA, POC)	Biomedomics, COVID-19 IgM-IgG Rapid Test kit	IgG	SENS	unk	20/27	74.1 (55.3-86.8)		5; US	na
Ab (LFIA, POC)	Biomedomics, COVID-19 IgM-IgG Rapid Test kit	IgG	SPEC	na	70/70	100.0 (94.8-100.0)		5; US	na
Ab (LFIA, POC)	Biomedomics, COVID-19 IgM-IgG Rapid Test kit	IgG or IgM	SENS	unk	26/27	96.3 (81.7-99.3)		5; US	na
Ab (LFIA, POC)	Biomedomics, COVID-19 IgM-IgG Rapid Test kit	IgG or IgM	SPEC	na	68/70	97.1 (90.2-99.2)		5; US	na
Ab (LFIA, POC)	Biomedomics, COVID-19 IgM-IgG Rapid Test kit	IgM	SENS	unk	24/27	88.9 (71.9-96.1)		5; US	na
Ab (LFIA, POC)	Biomedomics, COVID-19 IgM-IgG Rapid Test kit	IgM	SPEC	na	68/70	97.1 (90.2-99.2)		5; US	na

Ab (LFIA, POC)	Biomerica, COVID-19 IgG/IgM Rapid test	IgG	SENS	mild/asymptomatic	6/10	60.0 (31.3-83.2)		4; NL	na
Ab (LFIA, POC)	Biomerica, COVID-19 IgG/IgM Rapid test	IgG	SENS	hospitalised	NaN/NaN	NaN (NaN-NaN)		4;	na
Ab (LFIA, POC)	Biomerica, COVID-19 IgG/IgM Rapid test	IgG	SPEC	na	25/25	100.0 (86.7-100.0)		4; NL	na
Ab (LFIA, POC)	Biomerica, COVID-19 IgG/IgM Rapid test	IgM	SENS	mild/asymptomatic	0/10	0.0 (0.0-27.8)		4; NL	na
Ab (LFIA, POC)	Biomerica, COVID-19 IgG/IgM Rapid test	IgM	SENS	hospitalised	NaN/NaN	NaN (NaN-NaN)		4;	na
Ab (CLIA)	Bioscience (Chongqing) Diagnostic Technology, IgG Antibody test kit for novel coronavirus 2019-nCoV	IgG	SENS	unk	NaN/NaN	NaN (NaN-NaN)		291;	na
Ab (CLIA)	Bioscience (Chongqing) Diagnostic Technology, IgG Antibody test kit for novel coronavirus 2019-nCoV	IgG	SPEC	na	126/130	96.9 (92.4-98.8)		291; CN	na
Ab (CLIA)	Bioscience (Chongqing) Diagnostic Technology, IgM Antibody test kit for novel coronavirus 2019-nCoV	IgM	SENS	unk	NaN/NaN	NaN (NaN-NaN)		291;	na
Ab (CLIA)	Bioscience (Chongqing) Diagnostic Technology, IgM Antibody test kit for novel coronavirus 2019-nCoV	IgM	SPEC	na	122/130	93.8 (88.3-96.8)		291; CN	na
Ab (LFIA, POC)	Biosynex, COVID-19 BSS	IgG	SENS	mild/asymptomatic	100/131	76.3 (68.4-82.8)		67; FR	na
Ab (LFIA, POC)	Biosynex, COVID-19 BSS	IgG	SENS	hospitalised	66/73	90.4 (81.5-95.3)		4; NL	na
Ab (LFIA, POC)	Biosynex, COVID-19 BSS	IgG	SPEC	na	53/53	100.0 (93.2-100.0)		4; NL	na
Ab (LFIA, POC)	Biosynex, COVID-19 BSS	IgG or IgM	SENS	mild/asymptomatic	126/131	96.2 (91.4-98.4)		67; FR	na
Ab (LFIA, POC)	Biosynex, COVID-19 BSS	IgM	SENS	mild/asymptomatic	115/131	87.8 (81.1-92.3)		67; FR	na
Ab (LFIA, POC)	Biosynex, COVID-19 BSS	IgM	SENS	hospitalised	66/73	90.4 (81.5-95.3)		4; NL	na
Ab (LFIA, POC)	Biozek, COVID-19 IgG/IgM Rapid Test	IgG	SENS	mild/asymptomatic	18/21	85.7 (65.4-95.0)		4; NL	na
Ab (LFIA, POC)	Biozek, COVID-19 IgG/IgM Rapid Test	IgG	SENS	hospitalised	102/107	95.3 (89.5-98.0)		4; NL	na
Ab (LFIA, POC)	Biozek, COVID-19 IgG/IgM Rapid Test	IgG	SPEC	na	480/489	98.2 (96.5-99.0)		4; NL	na
Ab (LFIA, POC)	Biozek, COVID-19 IgG/IgM Rapid Test	IgM	SENS	mild/asymptomatic	6/21	28.6 (13.8-50.0)		4; NL	na
Ab (LFIA, POC)	Biozek, COVID-19 IgG/IgM Rapid Test	IgM	SENS	hospitalised	56/107	52.3 (43.0-61.6)		4; NL	na
Ab (LFIA, POC)	CTK Biotech, OnSite COVID-19 IgG/IgM Rapid Test	IgG	SENS	mild/asymptomatic	19/23	82.6 (62.9-93.0)		278; NO	na
Ab (LFIA, POC)	CTK Biotech, OnSite COVID-19 IgG/IgM Rapid Test	IgG	SENS	unk	52/66	78.8 (67.5-86.9)		7; AU	na
Ab (LFIA, POC)	CTK Biotech, OnSite COVID-19 IgG/IgM Rapid Test	IgG	SPEC	na	146/148	98.6 (95.2-99.6)	0.0	4, 7; AU, NL	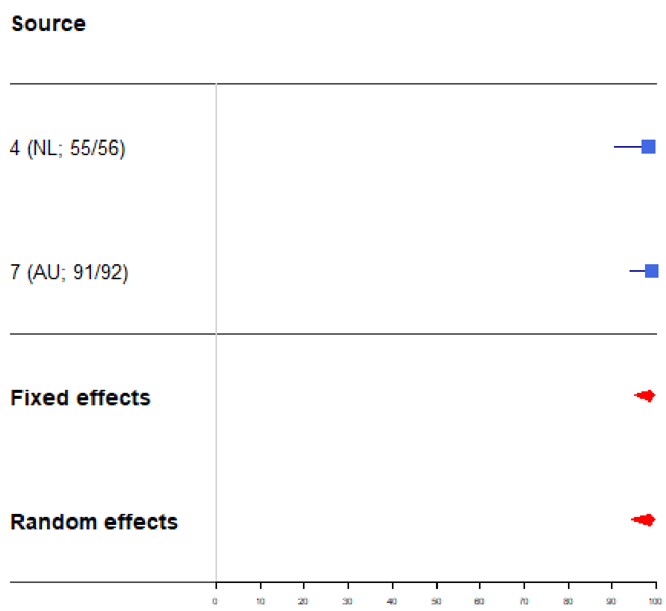
Ab (LFIA, POC)	CTK Biotech, OnSite COVID-19 IgG/IgM Rapid Test	IgG or IgM	SENS	mild/asymptomatic	18/37	48.6 (33.4-64.1)		4; NL	na
Ab (LFIA, POC)	CTK Biotech, OnSite COVID-19 IgG/IgM Rapid Test	IgG or IgM	SENS	hospitalised	8/8	100.0 (67.6-100.0)		150; DK	na
Ab (LFIA, POC)	CTK Biotech, OnSite COVID-19 IgG/IgM Rapid Test	IgG or IgM	SENS	unk	56/66	84.8 (74.3-91.6)		7; AU	na
Ab (LFIA, POC)	CTK Biotech, OnSite COVID-19 IgG/IgM Rapid Test	IgG or IgM	SPEC	na	214/224	95.5 (92.0-97.6)	0.0	7, 150, 218; AU, DK, UK	

									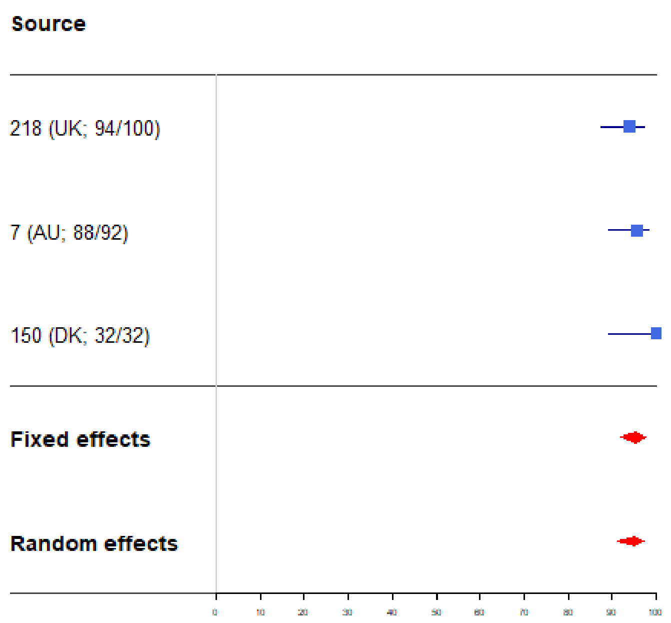
Ab (LFIA, POC)	CTK Biotech, OnSite COVID-19 IgG/IgM Rapid Test	IgM	SENS	mild/asymptomatic	20/23	87.0 (67.9-95.5)		278; NO	na
Ab (LFIA, POC)	CTK Biotech, OnSite COVID-19 IgG/IgM Rapid Test	IgM	SENS	unk	46/66	69.7 (57.8-79.4)		7; AU	na
Ab (LFIA, POC)	CTK Biotech, OnSite COVID-19 IgG/IgM Rapid Test	IgM	SPEC	na	89/92	96.7 (90.8-98.9)		7; AU	na
Ab (LFIA, POC)	Cellex, qSARS-CoV-2 IgG/IgM cassette Rapid test	IgG	SENS	mild/asymptomatic	50/65	76.9 (65.4-85.5)		4; NL	na
Ab (LFIA, POC)	Cellex, qSARS-CoV-2 IgG/IgM cassette Rapid test	IgG	SENS	hospitalised	31/31	100.0 (89.0-100.0)		4; NL	na
Ab (LFIA, POC)	Cellex, qSARS-CoV-2 IgG/IgM cassette Rapid test	IgG	SPEC	na	110/112	98.2 (93.7-99.5)		4; NL	na
Ab (LFIA, POC)	Cellex, qSARS-CoV-2 IgG/IgM cassette Rapid test	IgG or IgM	SPEC	na	97/98	99.0 (94.4-99.8)		75; NL	na
Ab (LFIA, POC)	Cellex, qSARS-CoV-2 IgG/IgM cassette Rapid test	IgM	SENS	mild/asymptomatic	15/65	23.1 (14.5-34.6)		4; NL	na
Ab (LFIA, POC)	Cellex, qSARS-CoV-2 IgG/IgM cassette Rapid test	IgM	SENS	hospitalised	12/31	38.7 (23.7-56.2)		4; NL	na
Ab (LFIA, POC)	Chembio, DPP COVID-19 IgM/IgG System	IgG	SENS	unk	19/27	70.4 (51.5-84.1)		5; US	na
Ab (LFIA, POC)	Chembio, DPP COVID-19 IgM/IgG System	IgG	SPEC	na	65/70	92.9 (84.3-96.9)		5; US	na
Ab (LFIA, POC)	Chembio, DPP COVID-19 IgM/IgG System	IgG or IgM	SENS	unk	20/27	74.1 (55.3-86.8)		5; US	na
Ab (LFIA, POC)	Chembio, DPP COVID-19 IgM/IgG System	IgG or IgM	SPEC	na	59/70	84.3 (74.0-91.0)		5; US	na
Ab (LFIA, POC)	Chembio, DPP COVID-19 IgM/IgG System	IgM	SENS	unk	13/27	48.1 (30.7-66.0)		5; US	na
Ab (LFIA, POC)	Chembio, DPP COVID-19 IgM/IgG System	IgM	SPEC	na	63/70	90.0 (80.8-95.1)		5; US	na
Ab (LFIA, POC)	Chemtron Biotech, Rapid COVID-19 IgM/IgG Antibody Screen Test	IgG	SENS	unk	16/27	59.3 (40.7-75.5)		5; US	na
Ab (LFIA, POC)	Chemtron Biotech, Rapid COVID-19 IgM/IgG Antibody Screen Test	IgG	SPEC	na	69/70	98.6 (92.3-99.7)		5; US	na
Ab (LFIA, POC)	Chemtron Biotech, Rapid COVID-19 IgM/IgG Antibody Screen Test	IgG or IgM	SENS	unk	17/27	63.0 (44.2-78.5)		5; US	na
Ab (LFIA, POC)	Chemtron Biotech, Rapid COVID-19 IgM/IgG Antibody Screen Test	IgG or IgM	SPEC	na	69/70	98.6 (92.3-99.7)		5; US	na
Ab (LFIA, POC)	Chemtron Biotech, Rapid COVID-19 IgM/IgG Antibody Screen Test	IgM	SENS	unk	5/27	18.5 (8.2-36.7)		5; US	na
Ab (LFIA, POC)	Chemtron Biotech, Rapid COVID-19 IgM/IgG Antibody Screen Test	IgM	SPEC	na	70/70	100.0 (94.8-100.0)		5; US	na
Ab (LFIA, POC)	Chongqing iSIA BIO-Technology, 2019-nCoV IgM/IgG Diagnostic Test Kit	IgG	SENS	hospitalised	12/15	80.0 (54.8-93.0)		284; FR	na
Ab (LFIA, POC)	Chongqing iSIA BIO-Technology, 2019-nCoV IgM/IgG Diagnostic Test Kit	IgG	SPEC	na	20/20	100.0 (83.9-100.0)		284; FR	na
Ab (LFIA, POC)	Chongqing iSIA BIO-Technology, 2019-nCoV IgM/IgG Diagnostic Test Kit	IgM	SENS	hospitalised	14/15	93.3 (70.2-98.8)		284; FR	na
Ab (LFIA, POC)	Chongqing iSIA BIO-Technology, 2019-nCoV IgM/IgG Diagnostic Test Kit	IgM	SPEC	na	20/20	100.0 (83.9-100.0)		284; FR	na
Ab (ELISA)	Creative Diagnostics,	IgG	SENS	mild/asymptomatic	24/46	52.2 (38.1-		3; NL	na

	SARS-CoV-2 IgG ELISA Kit					65.9)			
Ab (ELISA)	Creative Diagnostics, SARS-CoV-2 IgG ELISA Kit	IgG	SENS	hospitalised	NaN/NaN	NaN (NaN-NaN)		3;	na
Ab (ELISA)	Creative Diagnostics, SARS-CoV-2 IgG ELISA Kit	IgG	SPEC	na	77/78	98.7 (93.1-99.8)		3; NL	na
Ab (ELISA)	Creative Diagnostics, SARS-CoV-2 IgG ELISA Kit	IgM	SENS	mild/asymptomatic	NaN/NaN	NaN (NaN-NaN)		3;	na
Ab (ELISA)	Creative Diagnostics, SARS-CoV-2 IgG ELISA Kit	IgM	SENS	hospitalised	NaN/NaN	NaN (NaN-NaN)		3;	na
Ab (ELISA)	Creative Diagnostics, SARS-CoV-2 IgG ELISA Kit	IgM	SPEC	na	76/78	97.4 (91.1-99.3)		3; NL	na
Ab (ELISA)	DRG, Coronavirus COVID-19 IgG ELISA	IgG	SENS	hospitalised	20/21	95.2 (77.3-99.2)		44; CA	na
Ab (ELISA)	DRG, Coronavirus COVID-19 IgG ELISA	IgG	SPEC	na	50/50	100.0 (92.9-100.0)		44; CA	na
Ab (ELISA)	DRG, Coronavirus COVID-19 IgM ELISA	IgM	SENS	hospitalised	12/21	57.1 (36.5-75.5)		44; CA	na
Ab (ELISA)	DRG, Coronavirus COVID-19 IgM ELISA	IgM	SPEC	na	49/50	98.0 (89.5-99.6)		44; CA	na
Ab (LFIA, POC)	Decombio, Noval Coronavirus (SARS-COV-2) IgM/IgG Combo Rapid Test Kit	IgG	SENS	unk	24/29	82.8 (65.5-92.4)		297; US	na
Ab (LFIA, POC)	Decombio, Noval Coronavirus (SARS-COV-2) IgM/IgG Combo Rapid Test Kit	IgG	SPEC	na	98/107	91.6 (84.8-95.5)		297; US	na
Ab (LFIA, POC)	Decombio, Noval Coronavirus (SARS-COV-2) IgM/IgG Combo Rapid Test Kit	IgG or IgM	SENS	unk	24/29	82.8 (65.5-92.4)		297; US	na
Ab (LFIA, POC)	Decombio, Noval Coronavirus (SARS-COV-2) IgM/IgG Combo Rapid Test Kit	IgG or IgM	SPEC	na	96/107	89.7 (82.5-94.2)		297; US	na
Ab (LFIA, POC)	Decombio, Noval Coronavirus (SARS-COV-2) IgM/IgG Combo Rapid Test Kit	IgM	SENS	unk	24/29	82.8 (65.5-92.4)		297; US	na
Ab (LFIA, POC)	Decombio, Noval Coronavirus (SARS-COV-2) IgM/IgG Combo Rapid Test Kit	IgM	SPEC	na	97/107	90.7 (83.6-94.8)		297; US	na
Ab (CLIA)	DiaSorin, Liaison XL S1/S2 IgG chemiluminescence immunoassay	IgG	SENS	mild/asymptomatic	185/226	81.9 (76.3-86.3)	0.0	3, 180; NL, UK	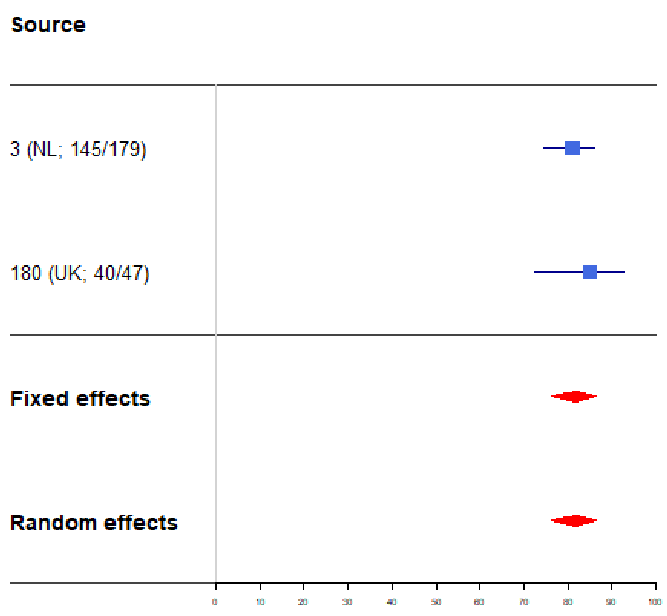
Ab (CLIA)	DiaSorin, Liaison XL S1/S2 IgG chemiluminescence immunoassay	IgG	SENS	hospitalised	301/324	92.9 (89.6-95.2)	73.6	3, 44, 226; CA, DE, NL	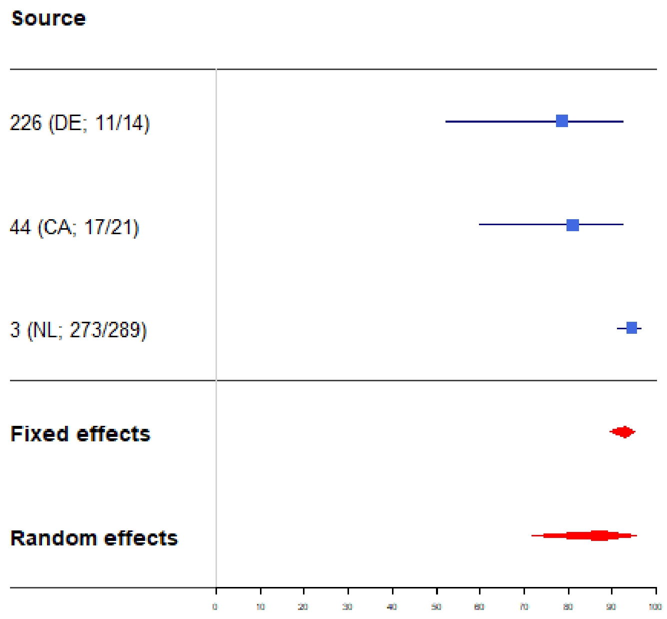

Ab (CLIA)	DiaSorin, Liaison XL S1/S2 IgG chemiluminescence immunoassay	IgG	SENS	unk	879/967	90.9 (88.9-92.6)	80.5	6, 65, 81, 83, 224, 285, 296, 299; AT(2), BE(2), DK, SE, UK, US	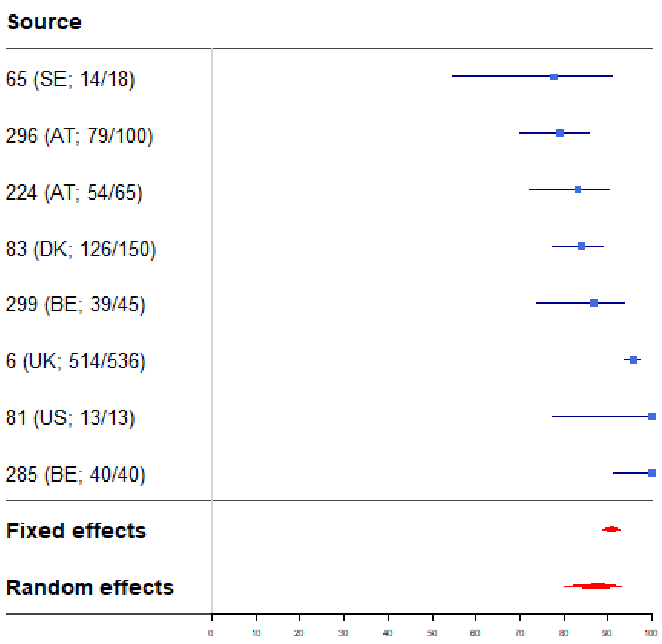
Ab (CLIA)	DiaSorin, Liaison XL S1/S2 IgG chemiluminescence immunoassay	IgG	SPEC	na	5854/5994	97.7 (97.3-98.0)	67.6	3, 6, 30, 44, 65, 75, 81, 83, 141, 145, 171, 224, 226, 269, 285, 299; AT, BE(2), CA, DE(3), DK, FI, FR, NL(2), SE, UK, US(2)	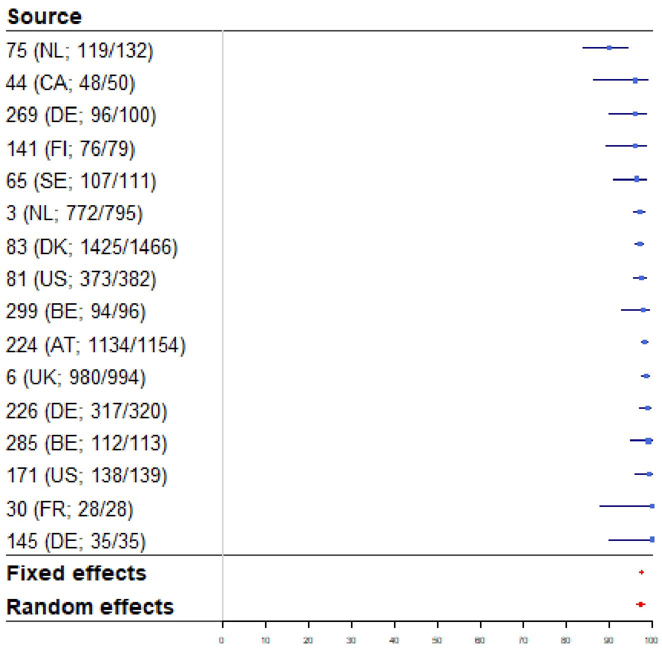
Ab (CLIA)	Diazyme Laboratories, DZ-Lite SARS-CoV-2 IgM and IgG CLIA	IgG	SENS	unk	41/43	95.3 (84.5-98.7)	0.0	270, 271; US(2)	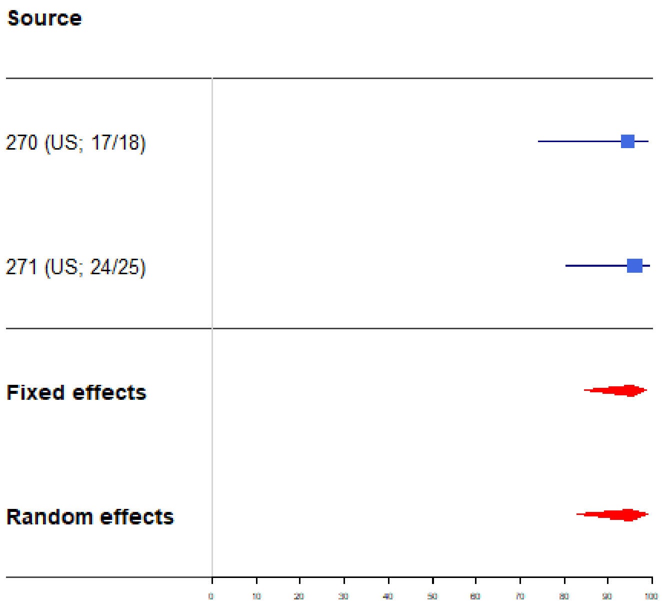
Ab (CLIA)	Diazyme Laboratories, DZ-Lite SARS-CoV-2 IgM and IgG CLIA	IgG	SPEC	na	410/414	99.0 (97.5-99.6)	0.0	270, 271; US(2)	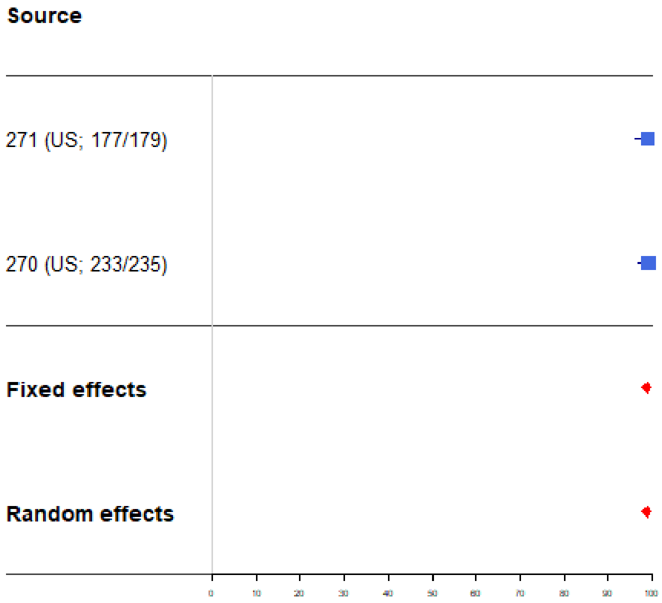
Ab (CLIA)	Diazyme Laboratories,	IgG or	SENS	unk	43/43	100.0	0.0	270, 271; US(2)	

	DZ-Lite SARS-CoV-2 IgM and IgG CLIA	IgM				(91.8-100.0)			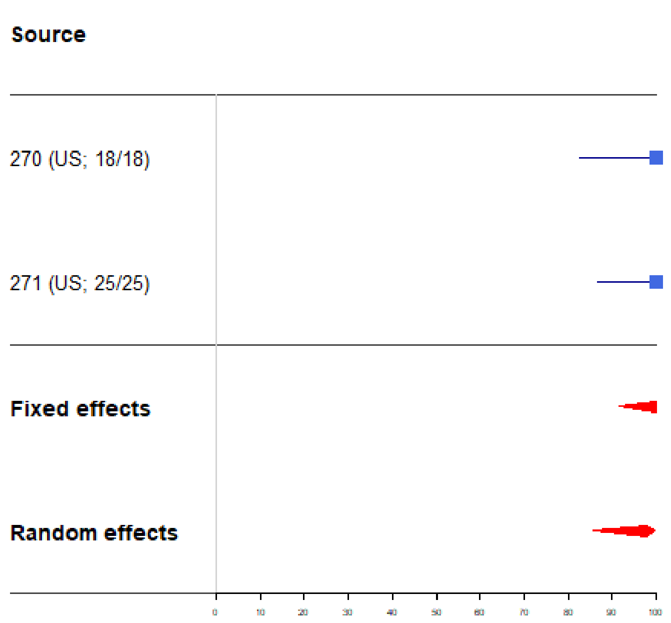
Ab (CLIA)	Diazyme Laboratories, DZ-Lite SARS-CoV-2 IgM and IgG CLIA	IgG or IgM	SPEC	na	408/414	98.6 (96.9-99.3)	0.0	270, 271; US(2)	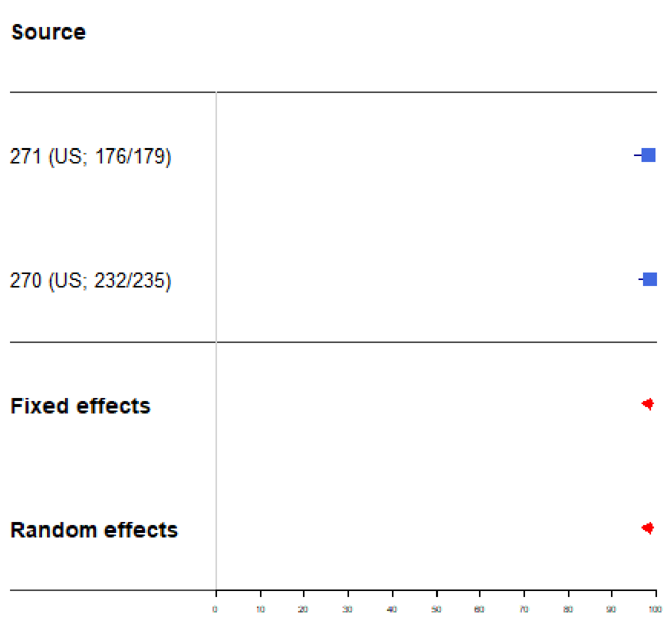
Ab (CLIA)	Diazyme Laboratories, DZ-Lite SARS-CoV-2 IgM and IgG CLIA	IgM	SENS	unk	39/43	90.7 (78.4-96.3)	0.0	270, 271; US(2)	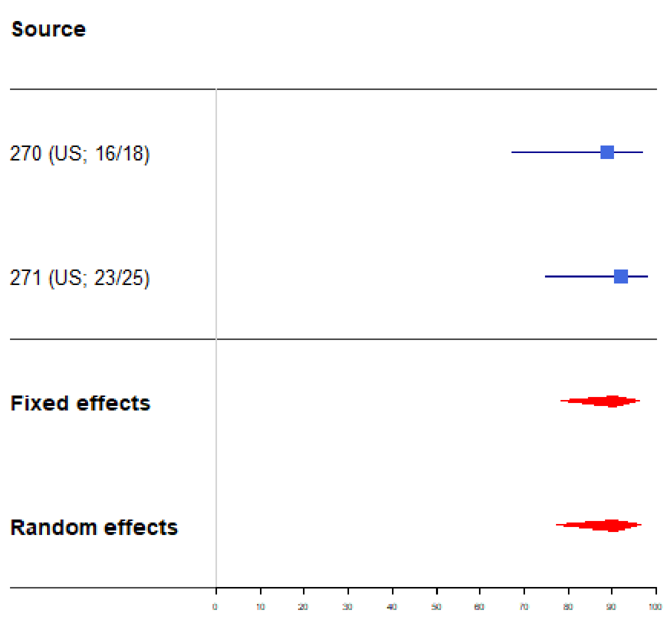
Ab (CLIA)	Diazyme Laboratories, DZ-Lite SARS-CoV-2 IgM and IgG CLIA	IgM	SPEC	na	412/414	99.5 (98.3-99.9)	0.0	270, 271; US(2)	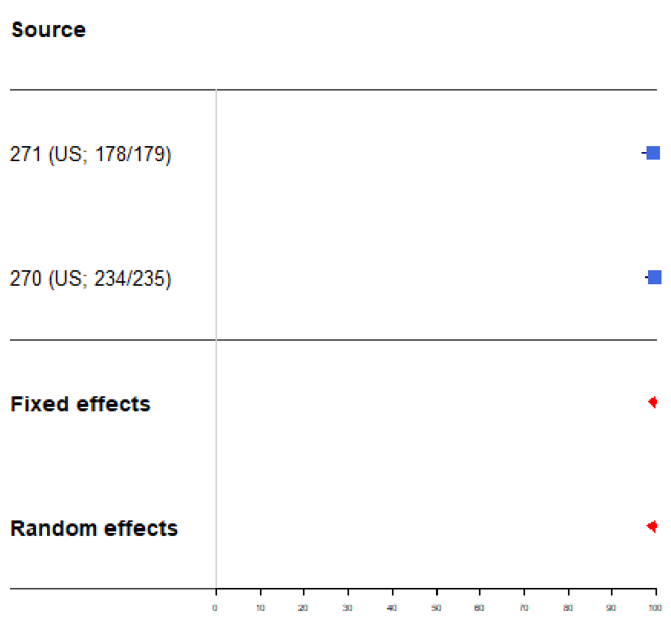
Ab (ELISA)	Diesse, ENZY-WELL	IgA	SENS	unk	NaN/NaN	NaN (NaN-		215;	na

	SARS-CoV-2 IgA					NaN)			
Ab (ELISA)	Diesse, ENZY-WELL SARS-CoV-2 IgA	IgA	SPEC	na	80/81	98.8 (93.3-99.8)		215; IT	na
Ab (ELISA)	Diesse, ENZY-WELL SARS-CoV-2 IgG	IgG	SENS	unk	NaN/NaN	NaN (NaN-NaN)		215;	na
Ab (ELISA)	Diesse, ENZY-WELL SARS-CoV-2 IgG	IgM	SPEC	na	80/81	98.8 (93.3-99.8)		215; IT	na
Ab (ELISA)	Diesse, ENZY-WELL SARS-CoV-2 IgM	IgG	SPEC	na	81/81	100.0 (95.5-100.0)		215; IT	na
Ab (ELISA)	Diesse, ENZY-WELL SARS-CoV-2 IgM	IgM	SENS	unk	NaN/NaN	NaN (NaN-NaN)		215;	na
Ab (LFIA, POC)	Dynamiker Biotechnology Tianjin, 2019 nCoV IgG/IgM Rapid test	IgG	SENS	mild/asymptomatic	6/11	54.5 (28.0-78.7)		4; NL	na
Ab (LFIA, POC)	Dynamiker Biotechnology Tianjin, 2019 nCoV IgG/IgM Rapid test	IgG	SENS	hospitalised	23/23	100.0 (85.7-100.0)		242; BE	na
Ab (LFIA, POC)	Dynamiker Biotechnology Tianjin, 2019 nCoV IgG/IgM Rapid test	IgG	SPEC	na	114/116	98.3 (93.9-99.5)	54.3	4, 242; BE, NL	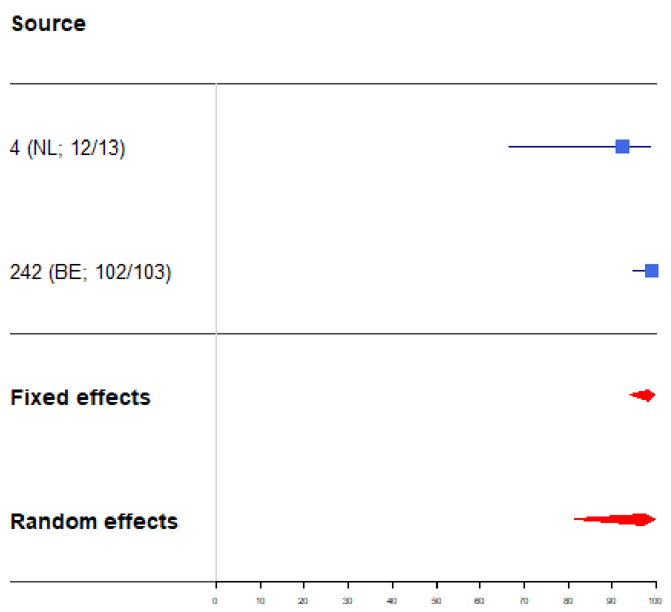
Ab (LFIA, POC)	Dynamiker Biotechnology Tianjin, 2019 nCoV IgG/IgM Rapid test	IgG or IgM	SENS	hospitalised	31/31	100.0 (89.0-100.0)	0.0	150, 242; BE, DK	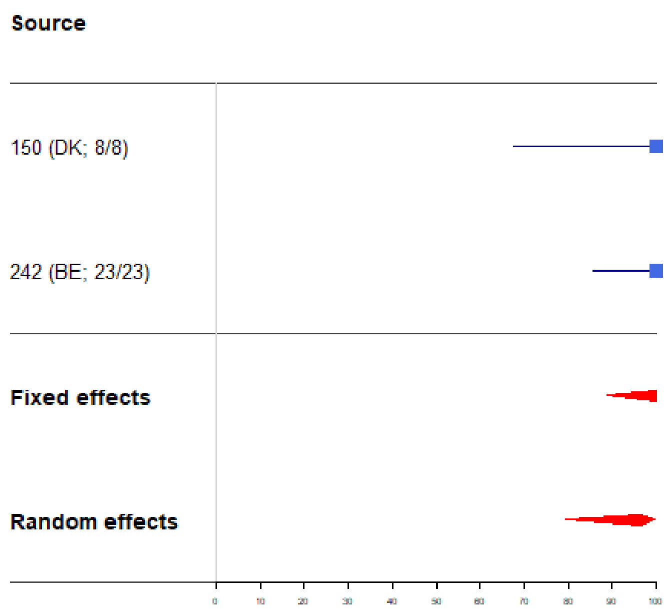
Ab (LFIA, POC)	Dynamiker Biotechnology Tianjin, 2019 nCoV IgG/IgM Rapid test	IgG or IgM	SENS	unk	65/73	89.0 (79.8-94.3)	78.9	65, 303; SE, TW	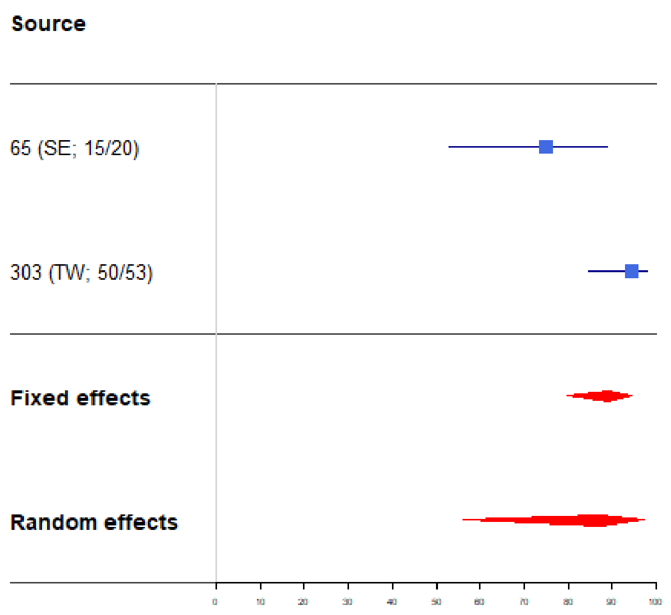
Ab (LFIA, POC)	Dynamiker Biotechnology Tianjin, 2019 nCoV IgG/IgM Rapid test	IgG or IgM	SPEC	na	242/248	97.6 (94.8-98.9)	37.8	65, 150, 242; BE, DK, SE	

									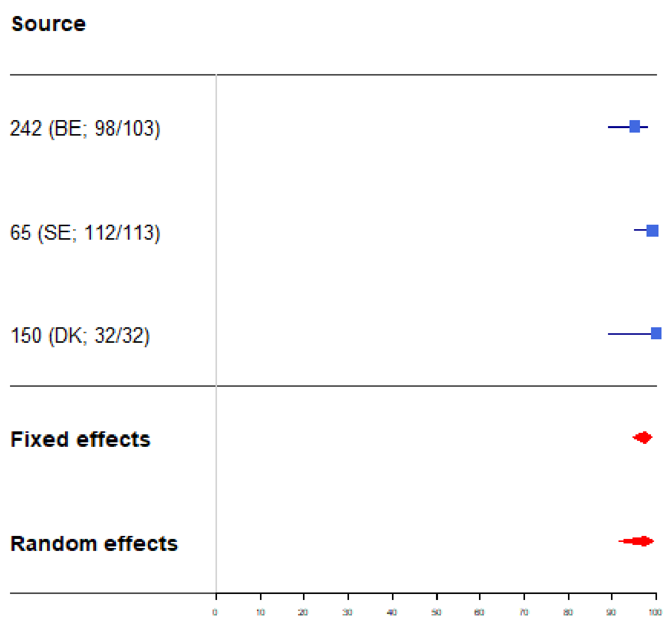
Ab (LFIA, POC)	Dynamiker Biotechnology Tianjin, 2019 nCoV IgG/IgM Rapid test	IgM	SENS	mild/asymptomatic	6/11	54.5 (28.0-78.7)		4; NL	na
Ab (LFIA, POC)	Dynamiker Biotechnology Tianjin, 2019 nCoV IgG/IgM Rapid test	IgM	SENS	hospitalised	23/23	100.0 (85.7-100.0)		242; BE	na
Ab (LFIA, POC)	Dynamiker Biotechnology Tianjin, 2019 nCoV IgG/IgM Rapid test	IgM	SPEC	na	98/103	95.1 (89.1-97.9)		242; BE	na
Ab (ELISA)	Epitope Diagnostics, EPI-KT-1032 Coronavirus COVID-19 IgG ELISA Kit	IgG	SENS	mild/asymptomatic	80/107	74.8 (65.8-82.0)	79.6	3, 277; NL, US	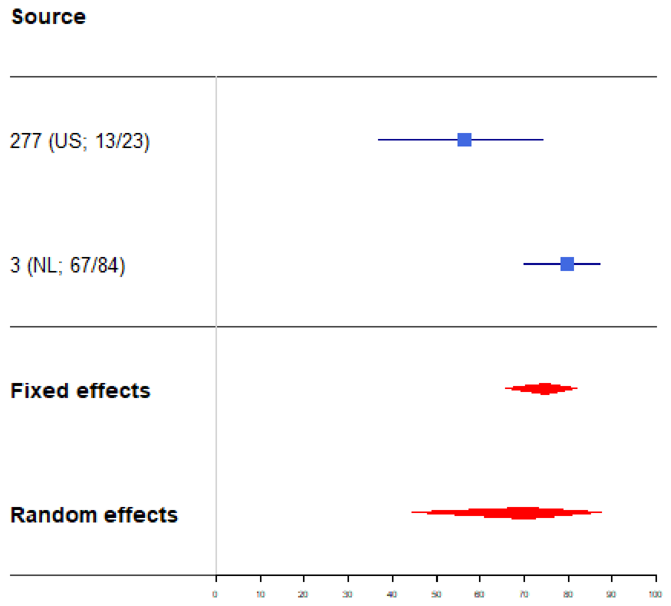
Ab (ELISA)	Epitope Diagnostics, EPI-KT-1032 Coronavirus COVID-19 IgG ELISA Kit	IgG	SENS	hospitalised	78/83	94.0 (86.7-97.4)	71.2	3, 44, 277; CA, NL, US	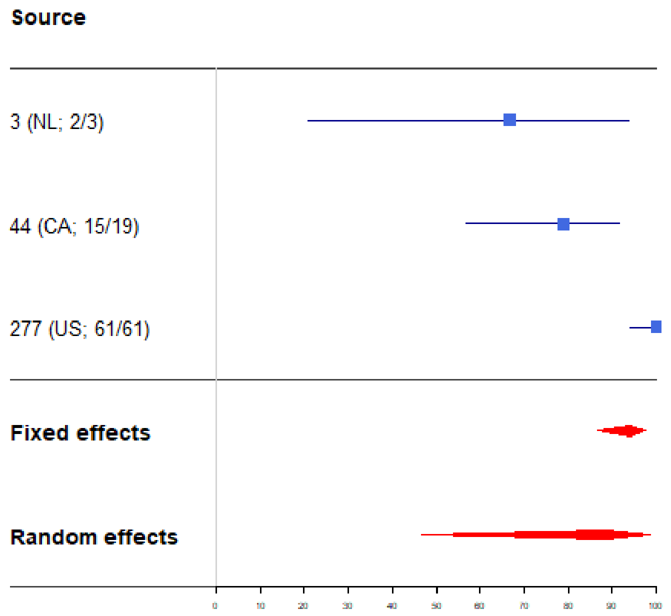
Ab (ELISA)	Epitope Diagnostics, EPI-KT-1032 Coronavirus COVID-19 IgG ELISA Kit	IgG	SENS	unk	95/99	96.0 (90.1-98.4)	51.9	37, 84, 297; AT, DE, US	

									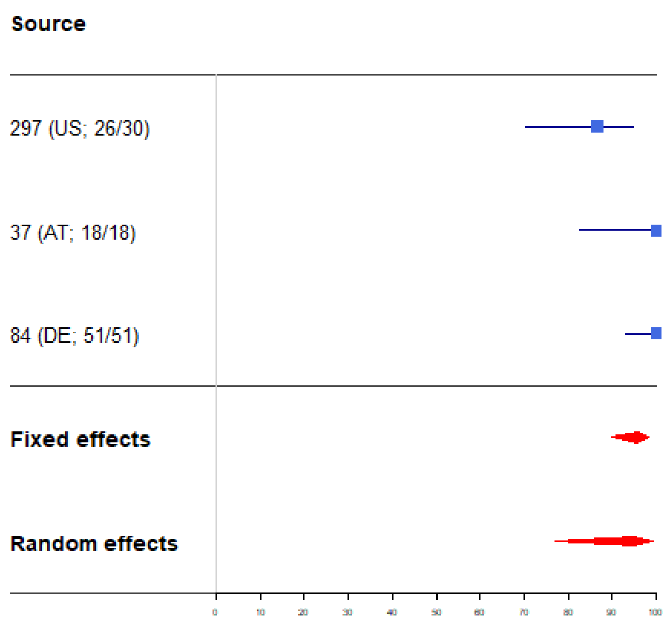
Ab (ELISA)	Epitope Diagnostics, EPI-KT-1032 Coronavirus COVID-19 IgG ELISA Kit	IgG	SPEC	na	1416/1451	97.6 (96.7-98.3)	73.1	3, 37, 44, 61, 84, 218, 269, 277, 297; AT, CA, DE(2), NL, UK, US(3)	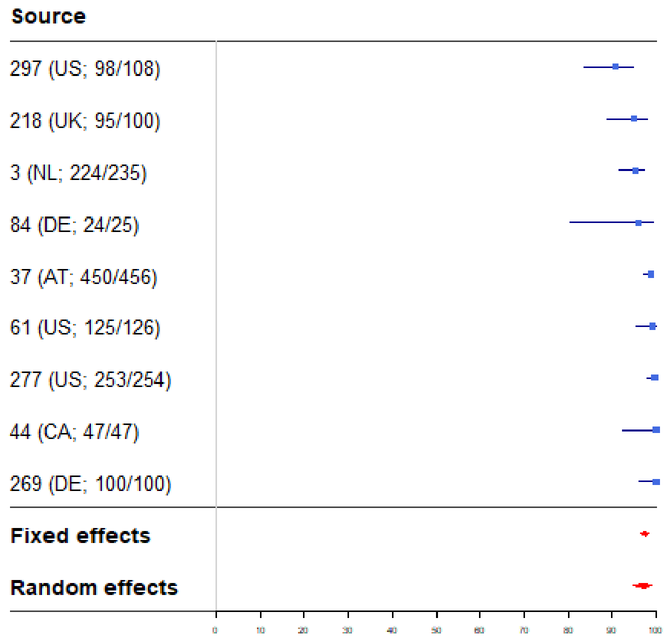
Ab (ELISA)	Epitope Diagnostics, EPI-KT-1033 Coronavirus COVID-19 IgM ELISA Kit	IgM	SENS	mild/asymptomatic	15/54	27.8 (17.6-40.9)		3; NL	na
Ab (ELISA)	Epitope Diagnostics, EPI-KT-1033 Coronavirus COVID-19 IgM ELISA Kit	IgM	SENS	hospitalised	21/22	95.5 (78.2-99.2)	59.8	3, 44; CA, NL	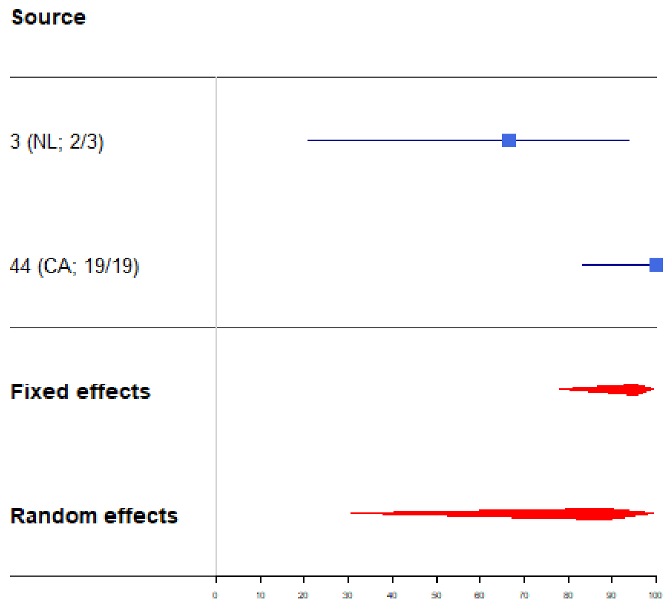
Ab (ELISA)	Epitope Diagnostics, EPI-KT-1033 Coronavirus COVID-19 IgM ELISA Kit	IgM	SENS	unk	40/48	83.3 (70.4-91.3)	53.9	37, 297; AT, US	

									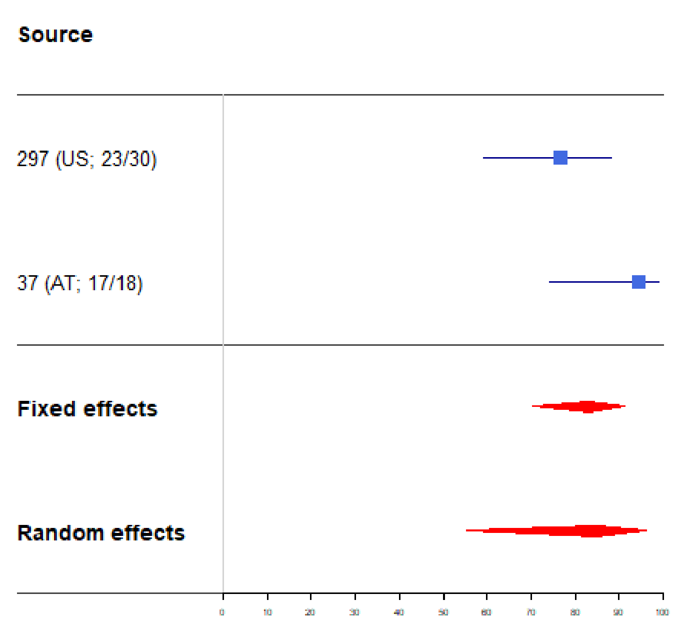
Ab (ELISA)	Epitope Diagnostics, EPI-KT-1033 Coronavirus COVID-19 IgM ELISA Kit	IgM	SPEC	na	795/810	98.1 (97.0-98.9)	0.0	3, 37, 44, 297; AT, CA, NL, US	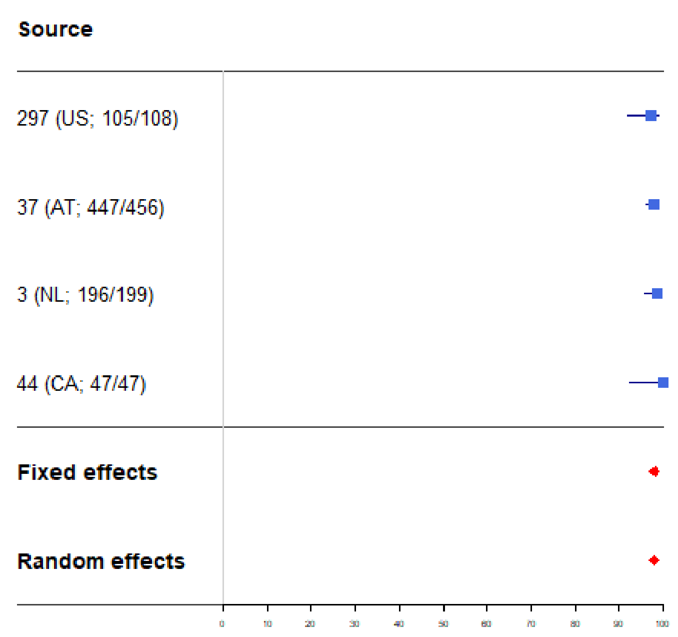
Ab (ELISA)	Euroimmun Medizinische Labordiagnostika, Anti-SARS-CoV-2 IgA S1 ELISA	IgA	SENS	mild/asymptomatic	43/64	67.2 (55.0-77.4)	0.0	3, 142; FI, NL	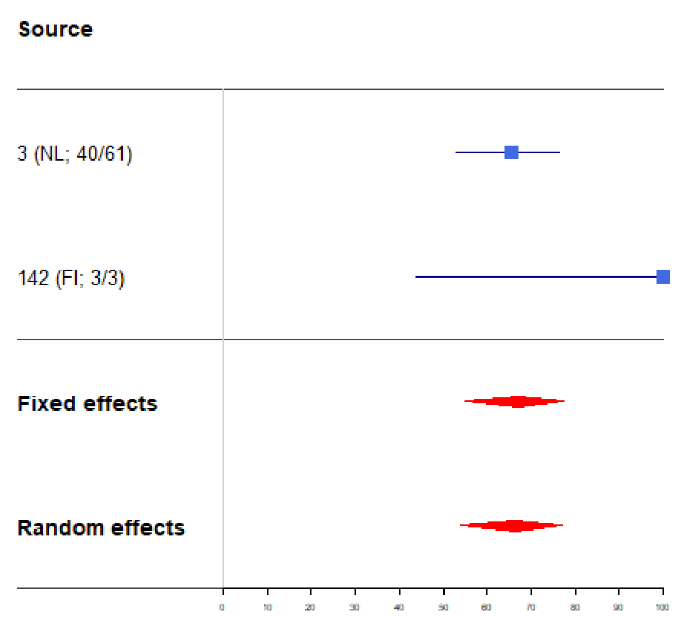
Ab (ELISA)	Euroimmun Medizinische Labordiagnostika, Anti-SARS-CoV-2 IgA S1 ELISA	IgA	SENS	hospitalised	215/224	96.0 (92.5-97.9)	0.0	3, 44, 142, 150, 177, 238, 241, 284; BE(2), CA, DK, FI, FR, GR, NL	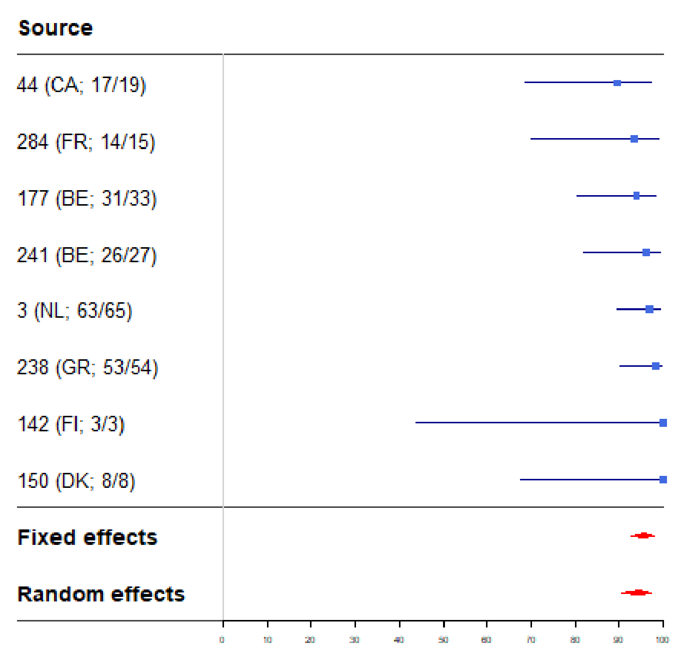
Ab (ELISA)	Euroimmun Medizinische	IgA	SENS	unk	201/212	94.8 (90.9-	0.0	7, 22, 208, 299;	

	Labordiagnostika, Anti-SARS-CoV-2 IgA S1 ELISA					97.1)		AU, BE, FR, US	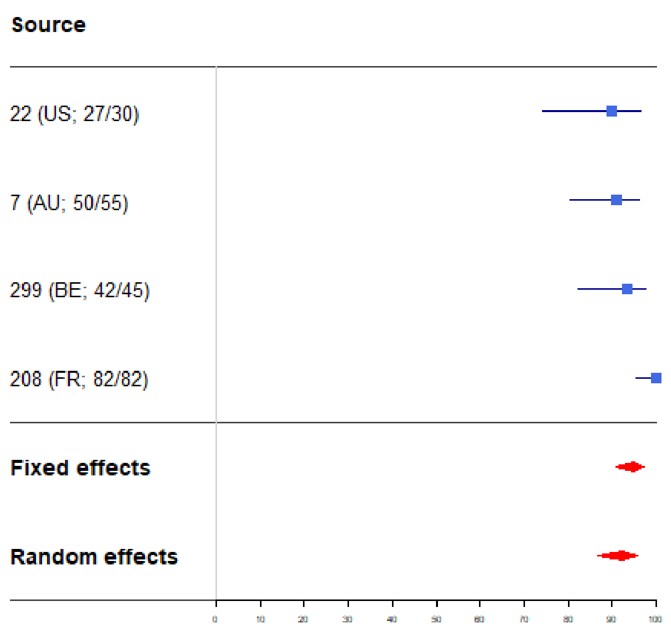
Ab (ELISA)	Euroimmun Medizinische Labordiagnostika, Anti-SARS-CoV-2 IgA S1 ELISA	IgA	SPEC	na	1265/1459	86.7 (84.9-88.3)	77.6	3, 7, 22, 44, 75, 141, 142, 150, 167, 177, 208, 238, 245, 284, 299; AU, BE(2), CA, DK, ES, FI(2), FR(2), GR, LU, NL(2), US	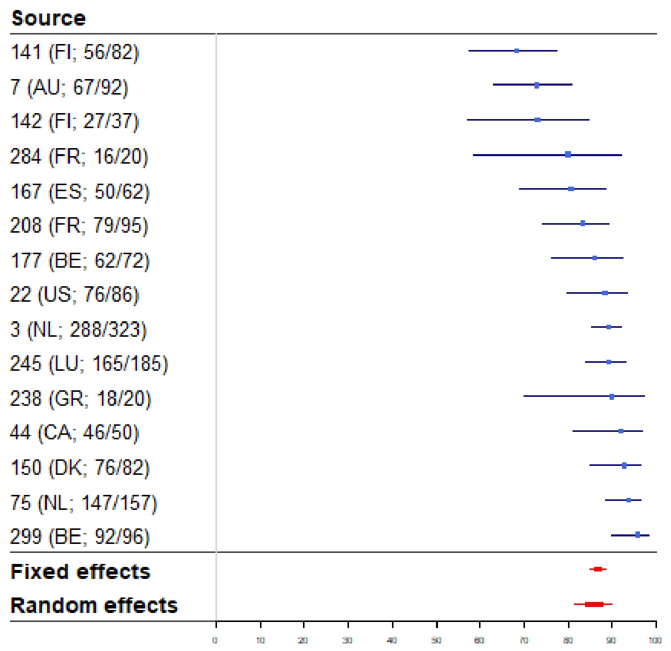
Ab (ELISA)	Euroimmun Medizinische Labordiagnostika, Anti-SARS-CoV-2 IgG NCP ELISA	IgG	SENS	unk	40/40	100.0 (91.2-100.0)		285; BE	na
Ab (ELISA)	Euroimmun Medizinische Labordiagnostika, Anti-SARS-CoV-2 IgG NCP ELISA	IgG	SPEC	na	107/113	94.7 (88.9-97.5)		285; BE	na
Ab (ELISA)	Euroimmun Medizinische Labordiagnostika, Anti-SARS-CoV-2 IgG S1 ELISA	IgG	SENS	mild/asymptomatic	105/132	79.5 (71.9-85.5)	83.0	3, 142, 174, 277; CH, FI, NL, US	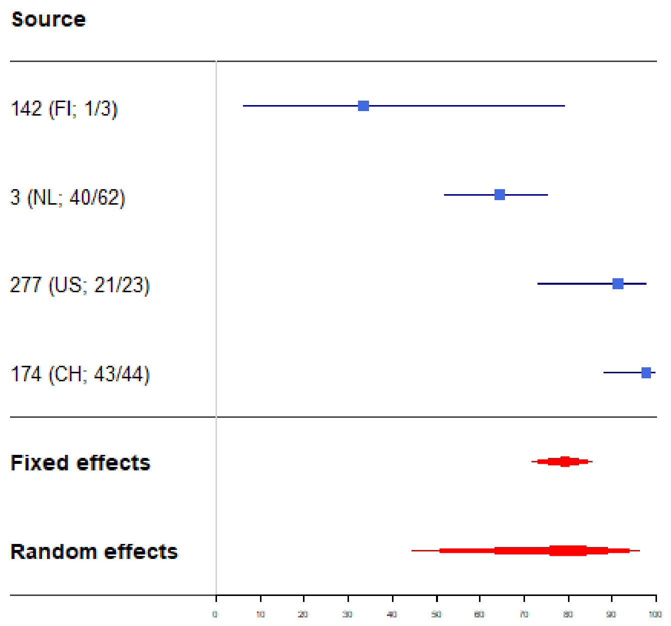
Ab (ELISA)	Euroimmun Medizinische Labordiagnostika, Anti-SARS-CoV-2 IgG S1 ELISA	IgG	SENS	hospitalised	399/431	92.6 (89.7-94.7)	0.0	3, 15, 44, 142, 150, 174, 177, 226, 238, 241, 242, 277, 284; BE(3), CA, CH(2), DE, DK, FI, FR, GR, NL, US	

									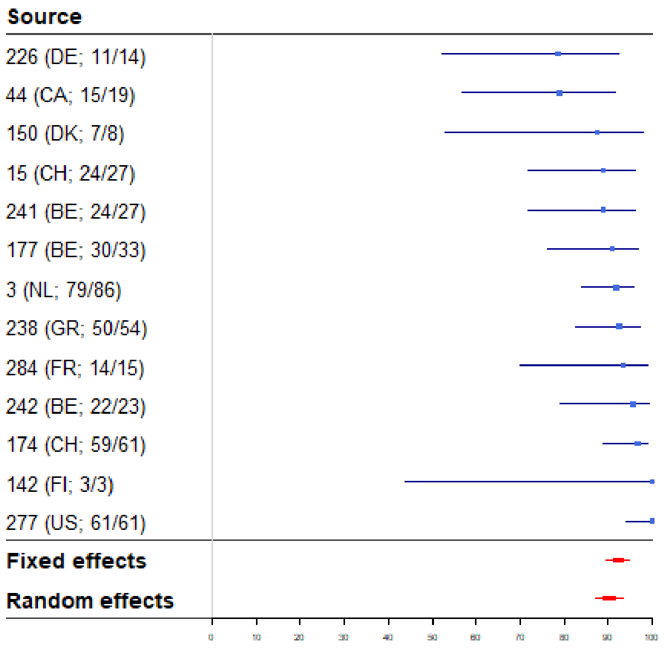
Ab (ELISA)	Euroimmun Medizinische Labordiagnostika, Anti-SARS-CoV-2 IgG S1 ELISA	IgG	SENS	unk	699/785	89.0 (86.7-91.0)	66.6	2, 5, 7, 22, 83, 84, 208, 255, 296, 299; AT, AU, BE, DE(2), DK, FR, UK, US(2)	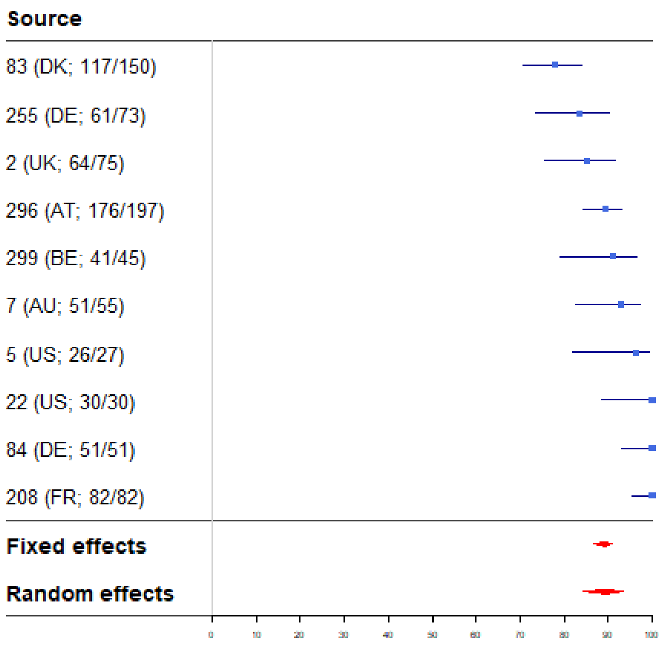
Ab (ELISA)	Euroimmun Medizinische Labordiagnostika, Anti-SARS-CoV-2 IgG S1 ELISA	IgG	SPEC	na	3871/3954	97.9 (97.4-98.3)	41.0	3, 5, 7, 15, 22, 30, 44, 61, 75, 83, 84, 93, 141, 142, 145, 146, 150, 167, 174, 177, 208, 226, 234, 242, 245, 269, 274, 277, 284, 299; AU, BE(3), CA, CH(2), DE(6), DK(2), ES, FI(2), FR(3), GR, LU, NL(2), US(5)	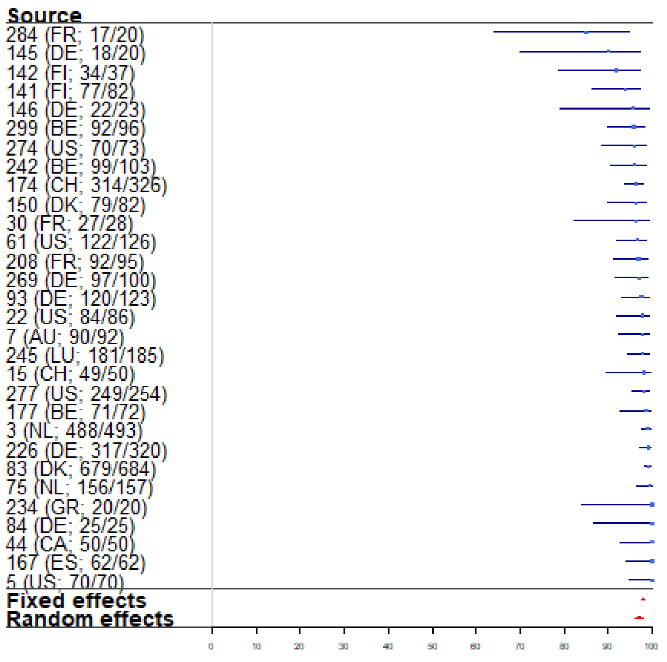
Ab (LFIA, POC)	GenBody, GenBody COVID-19 IgM/IgG	IgG	SENS	unk	16/29	55.2 (37.5-71.6)		5; US	na
Ab (LFIA, POC)	GenBody, GenBody COVID-19 IgM/IgG	IgG	SPEC	na	70/70	100.0 (94.8-100.0)		5; US	na
Ab (LFIA, POC)	GenBody, GenBody COVID-19 IgM/IgG	IgG or IgM	SENS	unk	17/29	58.6 (40.7-74.5)		5; US	na
Ab (LFIA, POC)	GenBody, GenBody COVID-19 IgM/IgG	IgG or IgM	SPEC	na	69/70	98.6 (92.3-99.7)		5; US	na
Ab (LFIA, POC)	GenBody, GenBody COVID-19 IgM/IgG	IgM	SENS	unk	11/29	37.9 (22.7-56.0)		5; US	na
Ab (LFIA, POC)	GenBody, GenBody COVID-19 IgM/IgG	IgM	SPEC	na	69/70	98.6 (92.3-99.7)		5; US	na
Ab (LFIA, POC)	Genobio Pharmaceutical, Virusee COVID-19 IgM/IgG Lateral Flow Assay	IgG	SENS	unk	18/29	62.1 (44.0-77.3)		5; US	na
Ab (LFIA, POC)	Genobio Pharmaceutical, Virusee COVID-19 IgM/IgG Lateral Flow Assay	IgG	SPEC	na	69/70	98.6 (92.3-99.7)		5; US	na
Ab (LFIA, POC)	Genobio Pharmaceutical, Virusee COVID-19 IgM/IgG Lateral Flow Assay	IgG or IgM	SENS	unk	23/29	79.3 (61.6-90.2)		5; US	na

Ab (LFIA, POC)	Genobio Pharmaceutical, Virusee COVID-19 IgM/IgG Lateral Flow Assay	IgG or IgM	SPEC	na	52/70	74.3 (63.0-83.1)		5; US	na
Ab (LFIA, POC)	Genobio Pharmaceutical, Virusee COVID-19 IgM/IgG Lateral Flow Assay	IgM	SENS	unk	16/29	55.2 (37.5-71.6)		5; US	na
Ab (LFIA, POC)	Genobio Pharmaceutical, Virusee COVID-19 IgM/IgG Lateral Flow Assay	IgM	SPEC	na	52/70	74.3 (63.0-83.1)		5; US	na
Ab (LFIA, POC)	Genrui Biotech, Novel Coronavirus (2019-nCoV) IgG/IgM Test Kit (Colloidal Gold)	IgG	SENS	hospitalised	16/20	80.0 (58.4-91.9)		44; CA	na
Ab (LFIA, POC)	Genrui Biotech, Novel Coronavirus (2019-nCoV) IgG/IgM Test Kit (Colloidal Gold)	IgG	SPEC	na	50/50	100.0 (92.9-100.0)		44; CA	na
Ab (LFIA, POC)	Genrui Biotech, Novel Coronavirus (2019-nCoV) IgG/IgM Test Kit (Colloidal Gold)	IgG or IgM	SENS	hospitalised	18/20	90.0 (69.9-97.2)		44; CA	na
Ab (LFIA, POC)	Genrui Biotech, Novel Coronavirus (2019-nCoV) IgG/IgM Test Kit (Colloidal Gold)	IgG or IgM	SPEC	na	50/50	100.0 (92.9-100.0)		44; CA	na
Ab (LFIA, POC)	Genrui Biotech, Novel Coronavirus (2019-nCoV) IgG/IgM Test Kit (Colloidal Gold)	IgM	SENS	hospitalised	18/20	90.0 (69.9-97.2)		44; CA	na
Ab (LFIA, POC)	Genrui Biotech, Novel Coronavirus (2019-nCoV) IgG/IgM Test Kit (Colloidal Gold)	IgM	SPEC	na	48/50	96.0 (86.5-98.9)		44; CA	na
Ab (ELISA)	Genscript, SARS-CoV-2 Spike S1-RBD IgG&IgM ELISA Detection Kit	IgG or IgM	SENS	unk	52/67	77.6 (66.3-85.9)		16; US	na
Ab (ELISA)	Genscript, SARS-CoV-2 Spike S1-RBD IgG&IgM ELISA Detection Kit	IgG or IgM	SPEC	na	422/468	90.2 (87.1-92.5)		16; US	na
Ab (LFIA, POC)	Getein Biotech, One Step Test for Novel Coronavirus (2019-nCoV) IgM/IgG Antibody (Colloidal Gold)	IgG	SENS	hospitalised	17/20	85.0 (64.0-94.8)		44; CA	na
Ab (LFIA, POC)	Getein Biotech, One Step Test for Novel Coronavirus (2019-nCoV) IgM/IgG Antibody (Colloidal Gold)	IgG	SENS	unk	19/27	70.4 (51.5-84.1)		5; US	na
Ab (LFIA, POC)	Getein Biotech, One Step Test for Novel Coronavirus (2019-nCoV) IgM/IgG Antibody (Colloidal Gold)	IgG	SPEC	na	120/120	100.0 (96.9-100.0)	0.0	5, 44; CA, US	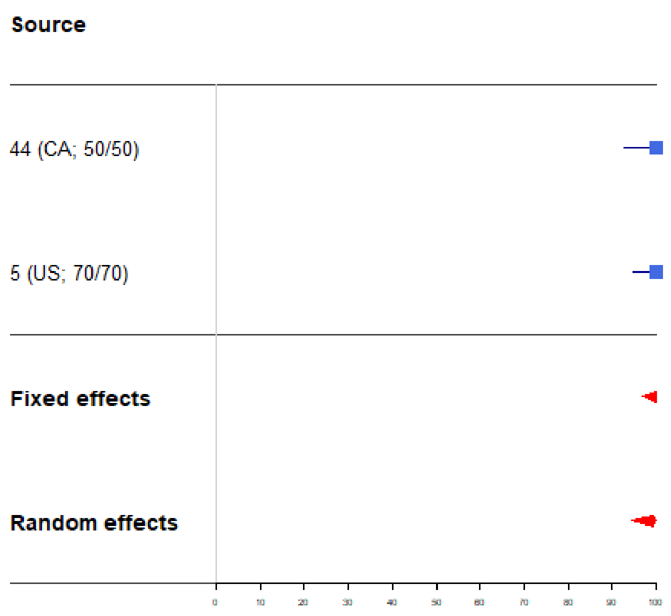
Ab (LFIA, POC)	Getein Biotech, One Step Test for Novel Coronavirus (2019-nCoV) IgM/IgG Antibody (Colloidal Gold)	IgG or IgM	SENS	hospitalised	17/20	85.0 (64.0-94.8)		44; CA	na
Ab (LFIA, POC)	Getein Biotech, One Step Test for Novel Coronavirus (2019-nCoV) IgM/IgG Antibody (Colloidal Gold)	IgG or IgM	SENS	unk	20/27	74.1 (55.3-86.8)		5; US	na
Ab (LFIA, POC)	Getein Biotech, One Step Test for Novel Coronavirus (2019-nCoV) IgM/IgG Antibody (Colloidal Gold)	IgG or IgM	SPEC	na	119/120	99.2 (95.4-99.9)	0.0	5, 44; CA, US	

									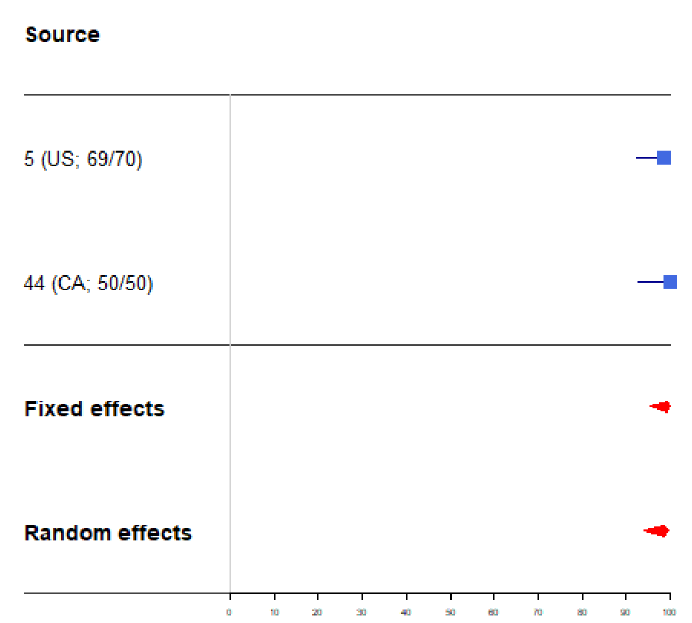
Ab (LFIA, POC)	Getein Biotech, One Step Test for Novel Coronavirus (2019-nCoV) IgM/IgG Antibody (Colloidal Gold)	IgM	SENS	hospitalised	1/20	5.0 (0.9-23.6)		44; CA	na
Ab (LFIA, POC)	Getein Biotech, One Step Test for Novel Coronavirus (2019-nCoV) IgM/IgG Antibody (Colloidal Gold)	IgM	SENS	unk	15/27	55.6 (37.3-72.4)		5; US	na
Ab (LFIA, POC)	Getein Biotech, One Step Test for Novel Coronavirus (2019-nCoV) IgM/IgG Antibody (Colloidal Gold)	IgM	SPEC	na	119/120	99.2 (95.4-99.9)	0.0	5, 44; CA, US	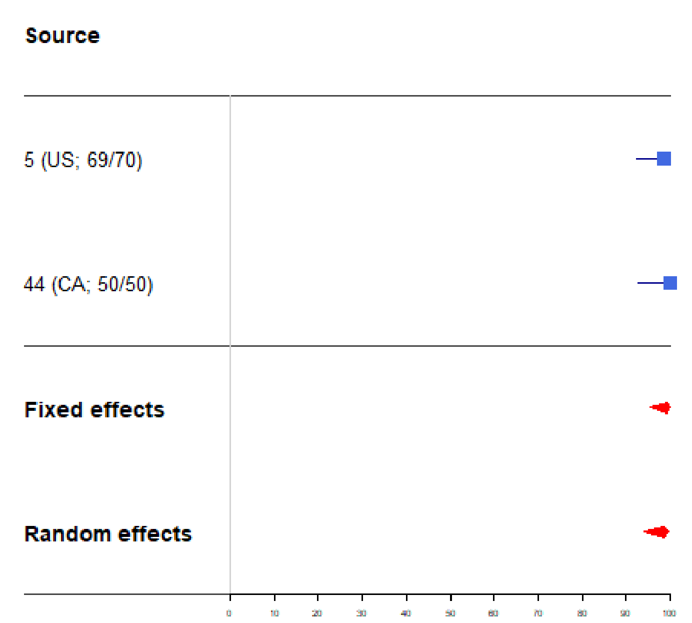
Ab (LFIA, POC)	GritOverseas, DiagnoSure COVID-19 IgG/IgM rapid test cassette	IgG	SENS	mild/asymptomatic	0/10	0.0 (0.0-27.8)		4; NL	na
Ab (LFIA, POC)	GritOverseas, DiagnoSure COVID-19 IgG/IgM rapid test cassette	IgG	SENS	hospitalised	NaN/NaN	NaN (NaN-NaN)		4;	na
Ab (LFIA, POC)	GritOverseas, DiagnoSure COVID-19 IgG/IgM rapid test cassette	IgG	SPEC	na	25/25	100.0 (86.7-100.0)		4; NL	na
Ab (LFIA, POC)	GritOverseas, DiagnoSure COVID-19 IgG/IgM rapid test cassette	IgM	SENS	mild/asymptomatic	2/10	20.0 (5.7-51.0)		4; NL	na
Ab (LFIA, POC)	GritOverseas, DiagnoSure COVID-19 IgG/IgM rapid test cassette	IgM	SENS	hospitalised	NaN/NaN	NaN (NaN-NaN)		4;	na
Ab (LFIA, POC)	Guangdong Hecin Biotech, 2019-nCoV IgM Antibody Test Kit	IgM	SENS	hospitalised	13/15	86.7 (62.1-96.3)		284; FR	na
Ab (LFIA, POC)	Guangdong Hecin Biotech, 2019-nCoV IgM Antibody Test Kit	IgM	SPEC	na	20/20	100.0 (83.9-100.0)		284; FR	na
Ab (LFIA, POC)	Guangzhou Fenghua Bioengineering, SARS-COV-2 IgM/IgG Combo Rapid Test Kit	IgG	SENS	unk	20/29	69.0 (50.8-82.7)		5; US	na
Ab (LFIA, POC)	Guangzhou Fenghua Bioengineering, SARS-COV-2 IgM/IgG Combo Rapid Test Kit	IgG	SPEC	na	66/70	94.3 (86.2-97.8)		5; US	na
Ab (LFIA, POC)	Guangzhou Fenghua Bioengineering, SARS-COV-2 IgM/IgG Combo Rapid Test Kit	IgG or IgM	SENS	unk	23/29	79.3 (61.6-90.2)		5; US	na
Ab (LFIA, POC)	Guangzhou Fenghua Bioengineering, SARS-COV-2 IgM/IgG Combo Rapid Test Kit	IgG or IgM	SPEC	na	43/70	61.4 (49.7-72.0)		5; US	na
Ab (LFIA, POC)	Guangzhou Fenghua Bioengineering, SARS-COV-2 IgM/IgG Combo Rapid Test Kit	IgM	SENS	unk	20/29	69.0 (50.8-82.7)		5; US	na

Ab (LFIA, POC)	Guangzhou Fenghua Bioengineering, SARS-COV-2 IgM/IgG Combo Rapid Test Kit	IgM	SPEC	na	45/70	64.3 (52.6-74.5)		5; US	na
Ab (LFIA, POC)	Guangzhou Wondfo Biotech, Wondfo SARS-CoV-2 Antibody Test	IgG or IgM	SENS	hospitalised	28/28	100.0 (87.9-100.0)		217; US	na
Ab (LFIA, POC)	Guangzhou Wondfo Biotech, Wondfo SARS-CoV-2 Antibody Test	IgG or IgM	SENS	unk	162/184	88.0 (82.6-92.0)	79.9	8, 78, 297, 303; AU, ES, TW, US	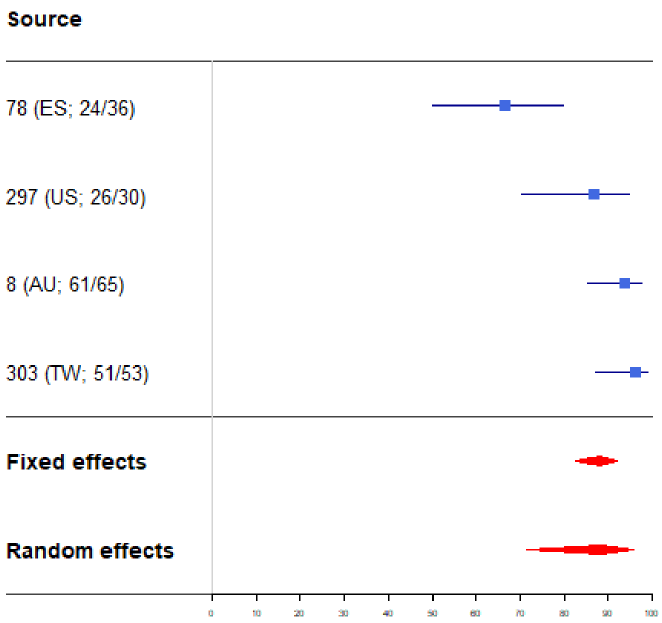
Ab (LFIA, POC)	Guangzhou Wondfo Biotech, Wondfo SARS-CoV-2 Antibody Test	IgG or IgM	SPEC	na	601/605	99.3 (98.3-99.7)	0.0	8, 51, 167, 217, 297; AU, BR, ES, US(2)	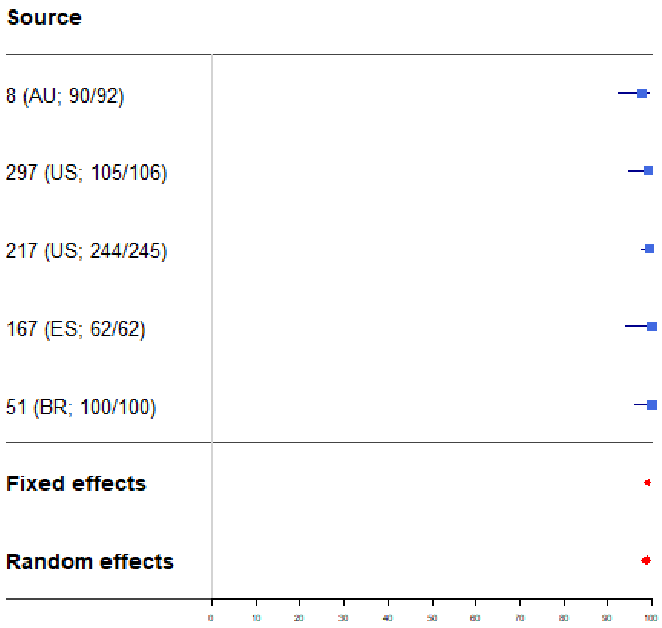
Ab (LFIA, POC)	H-Guard, Novel Coronavirus COVID-19 IgM/IgG Test Kit (colloidal gold)	IgG	SENS	unk	27/29	93.1 (78.0-98.1)		5; US	na
Ab (LFIA, POC)	H-Guard, Novel Coronavirus COVID-19 IgM/IgG Test Kit (colloidal gold)	IgG	SPEC	na	68/70	97.1 (90.2-99.2)		5; US	na
Ab (LFIA, POC)	H-Guard, Novel Coronavirus COVID-19 IgM/IgG Test Kit (colloidal gold)	IgG or IgM	SENS	unk	28/29	96.6 (82.8-99.4)		5; US	na
Ab (LFIA, POC)	H-Guard, Novel Coronavirus COVID-19 IgM/IgG Test Kit (colloidal gold)	IgG or IgM	SPEC	na	65/70	92.9 (84.3-96.9)		5; US	na
Ab (LFIA, POC)	H-Guard, Novel Coronavirus COVID-19 IgM/IgG Test Kit (colloidal gold)	IgM	SENS	unk	15/29	51.7 (34.4-68.6)		5; US	na
Ab (LFIA, POC)	H-Guard, Novel Coronavirus COVID-19 IgM/IgG Test Kit (colloidal gold)	IgM	SPEC	na	67/70	95.7 (88.1-98.5)		5; US	na
Ab (LFIA, POC)	Hangzhou Alltest Biotech, 2019-nCoV IgG/IgM Rapid Test Cassette	IgG	SENS	hospitalised	NaN/NaN	NaN (NaN-NaN)		71;	na
Ab (LFIA, POC)	Hangzhou Alltest Biotech, 2019-nCoV IgG/IgM Rapid Test Cassette	IgG	SENS	unk	102/115	88.7 (81.6-93.3)	0.0	8, 247; AU, ES	

										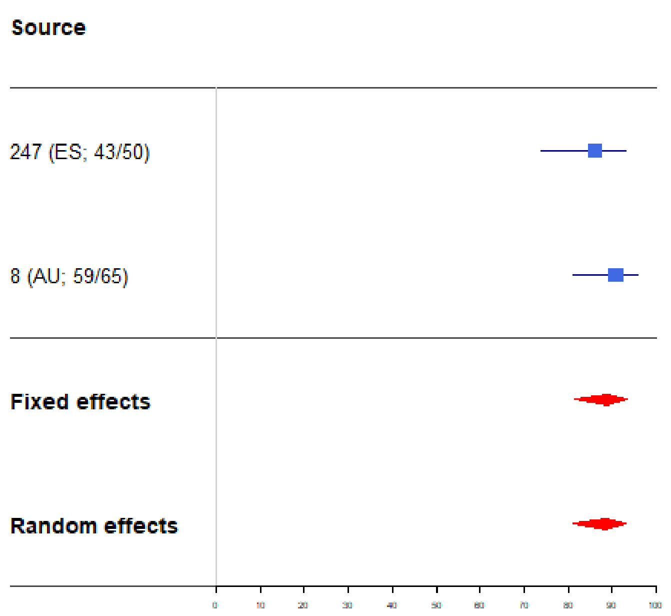
Ab (LFIA, POC)	Hangzhou Alltest Biotech, 2019-nCoV IgG/IgM Rapid Test Cassette	IgG	SPEC	na	254/254	100.0 (98.5-100.0)	0.0	8, 167, 247; AU, ES(2)		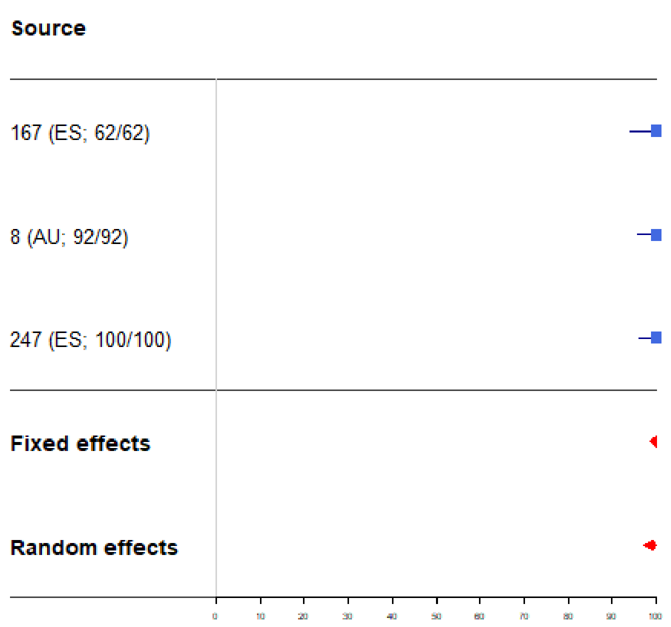
Ab (LFIA, POC)	Hangzhou Alltest Biotech, 2019-nCoV IgG/IgM Rapid Test Cassette	IgG or IgM	SENS	hospitalised	NaN/NaN	NaN (NaN-NaN)		150;		na
Ab (LFIA, POC)	Hangzhou Alltest Biotech, 2019-nCoV IgG/IgM Rapid Test Cassette	IgG or IgM	SENS	unk	155/168	92.3 (87.2-95.4)	0.0	8, 247, 303; AU, ES, TW		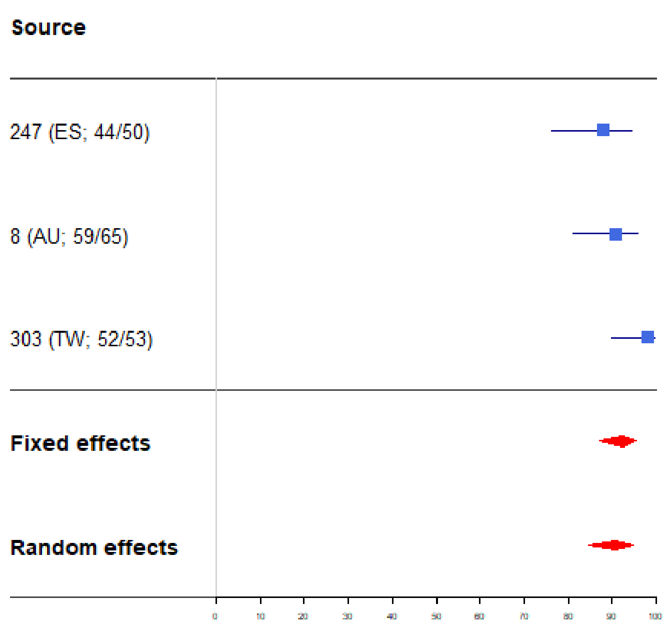
Ab (LFIA, POC)	Hangzhou Alltest Biotech, 2019-nCoV IgG/IgM Rapid Test Cassette	IgG or IgM	SPEC	na	260/269	96.7 (93.8-98.2)	29.4	8, 150, 167, 247; AU, DK, ES(2)		

									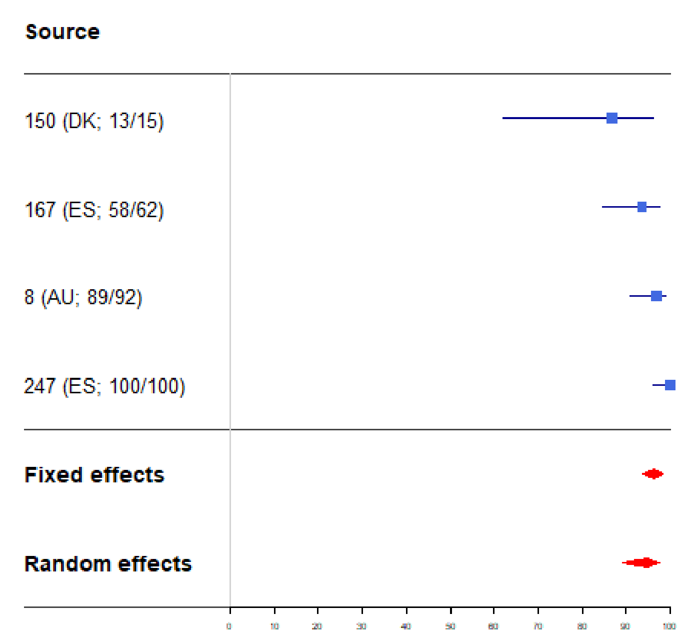
Ab (LFIA, POC)	Hangzhou Alltest Biotech, 2019-nCoV IgG/IgM Rapid Test Cassette	IgM	SENS	hospitalised	NaN/NaN	NaN (NaN-NaN)		71;	na
Ab (LFIA, POC)	Hangzhou Alltest Biotech, 2019-nCoV IgG/IgM Rapid Test Cassette	IgM	SENS	unk	25/115	21.7 (15.2-30.1)	89.6	8, 247; AU, ES	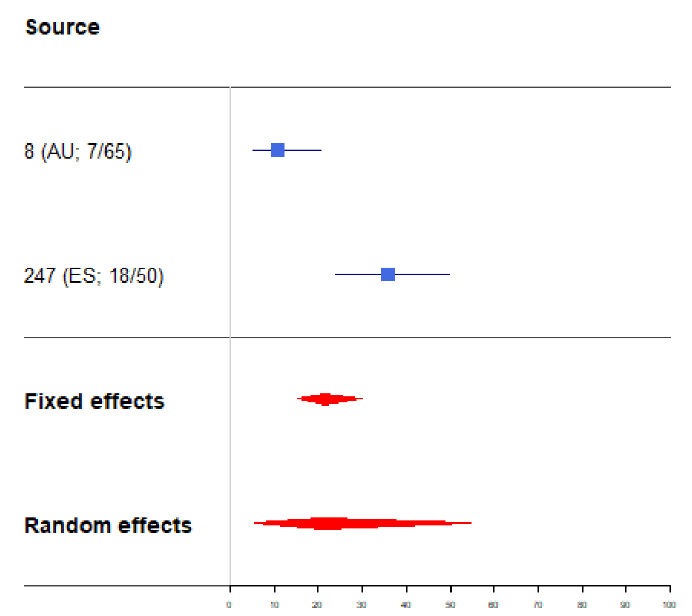
Ab (LFIA, POC)	Hangzhou Alltest Biotech, 2019-nCoV IgG/IgM Rapid Test Cassette	IgM	SPEC	na	247/254	97.2 (94.4-98.7)	20.3	8, 167, 247; AU, ES(2)	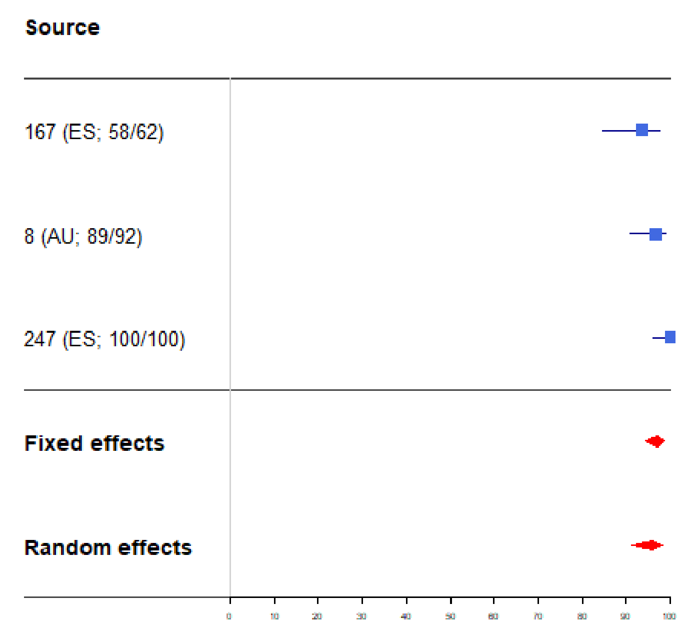
Ab (LFIA, POC)	Hangzhou Biotest Biotech, Lumiratek COVID-19 IgG/IgM Hurtigtest kassett	IgG	SENS	mild/asymptomatic	12/23	52.2 (33.0-70.8)		278; NO	na
Ab (LFIA, POC)	Hangzhou Biotest Biotech, Lumiratek COVID-19 IgG/IgM Hurtigtest kassett	IgG	SENS	unk	25/27	92.6 (76.6-97.9)		5; US	na
Ab (LFIA, POC)	Hangzhou Biotest Biotech, Lumiratek COVID-19 IgG/IgM Hurtigtest kassett	IgG	SPEC	na	70/70	100.0 (94.8-100.0)		5; US	na
Ab (LFIA, POC)	Hangzhou Biotest Biotech, Lumiratek COVID-19 IgG/IgM Hurtigtest kassett	IgG or IgM	SENS	unk	27/27	100.0 (87.5-100.0)		5; US	na
Ab (LFIA, POC)	Hangzhou Biotest Biotech, Lumiratek COVID-19 IgG/IgM Hurtigtest kassett	IgG or IgM	SPEC	na	70/70	100.0 (94.8-100.0)		5; US	na
Ab (LFIA, POC)	Hangzhou Biotest Biotech, Lumiratek	IgM	SENS	mild/asymptomatic	19/23	82.6 (62.9-93.0)		278; NO	na

	COVID-19 IgG/IgM Hurtigtest kassett								
Ab (LFIA, POC)	Hangzhou Biotest Biotech, Lumiratek COVID-19 IgG/IgM Hurtigtest kassett	IgM	SENS	unk	27/27	100.0 (87.5-100.0)		5; US	na
Ab (LFIA, POC)	Hangzhou Biotest Biotech, Lumiratek COVID-19 IgG/IgM Hurtigtest kassett	IgM	SPEC	na	70/70	100.0 (94.8-100.0)		5; US	na
Ab (LFIA, POC)	Hangzhou Clongene Biotech, 2019-nCoV IgG/IgM Rapid Test	IgG	SENS	hospitalised	23/23	100.0 (85.7-100.0)		242; BE	na
Ab (LFIA, POC)	Hangzhou Clongene Biotech, 2019-nCoV IgG/IgM Rapid Test	IgG	SPEC	na	101/103	98.1 (93.2-99.5)		242; BE	na
Ab (LFIA, POC)	Hangzhou Clongene Biotech, 2019-nCoV IgG/IgM Rapid Test	IgG or IgM	SENS	hospitalised	23/23	100.0 (85.7-100.0)		242; BE	na
Ab (LFIA, POC)	Hangzhou Clongene Biotech, 2019-nCoV IgG/IgM Rapid Test	IgG or IgM	SPEC	na	93/103	90.3 (83.0-94.6)		242; BE	na
Ab (LFIA, POC)	Hangzhou Clongene Biotech, 2019-nCoV IgG/IgM Rapid Test	IgM	SENS	hospitalised	13/23	56.5 (36.8-74.4)		242; BE	na
Ab (LFIA, POC)	Hangzhou Clongene Biotech, 2019-nCoV IgG/IgM Rapid Test	IgM	SPEC	na	94/103	91.3 (84.2-95.3)		242; BE	na
Ab (LFIA, POC)	Hangzhou Laihe Biotech, Novel coronavirus 2019nCov) IgM/IgG Antibody Combo Test Kit	IgG	SENS	mild/asymptomatic	9/23	39.1 (22.2-59.2)		278; NO	na
Ab (LFIA, POC)	Hangzhou Laihe Biotech, Novel coronavirus 2019nCov) IgM/IgG Antibody Combo Test Kit	IgG	SENS	unk	29/29	100.0 (88.3-100.0)		5; US	na
Ab (LFIA, POC)	Hangzhou Laihe Biotech, Novel coronavirus 2019nCov) IgM/IgG Antibody Combo Test Kit	IgG	SPEC	na	69/70	98.6 (92.3-99.7)		5; US	na
Ab (LFIA, POC)	Hangzhou Laihe Biotech, Novel coronavirus 2019nCov) IgM/IgG Antibody Combo Test Kit	IgG or IgM	SENS	unk	29/29	100.0 (88.3-100.0)		5; US	na
Ab (LFIA, POC)	Hangzhou Laihe Biotech, Novel coronavirus 2019nCov) IgM/IgG Antibody Combo Test Kit	IgG or IgM	SPEC	na	69/70	98.6 (92.3-99.7)		5; US	na
Ab (LFIA, POC)	Hangzhou Laihe Biotech, Novel coronavirus 2019nCov) IgM/IgG Antibody Combo Test Kit	IgM	SENS	mild/asymptomatic	21/23	91.3 (73.2-97.6)		278; NO	na
Ab (LFIA, POC)	Hangzhou Laihe Biotech, Novel coronavirus 2019nCov) IgM/IgG Antibody Combo Test Kit	IgM	SENS	unk	28/29	96.6 (82.8-99.4)		5; US	na
Ab (LFIA, POC)	Hangzhou Laihe Biotech, Novel coronavirus 2019nCov) IgM/IgG Antibody Combo Test Kit	IgM	SPEC	na	70/70	100.0 (94.8-100.0)		5; US	na
Ab (LFIA, POC)	Healgen, COVID-19 IgG/IgM Rapid Test Cassette	IgG	SENS	unk	26/27	96.3 (81.7-99.3)		5; US	na
Ab (LFIA, POC)	Healgen, COVID-19 IgG/IgM Rapid Test Cassette	IgG	SPEC	na	68/70	97.1 (90.2-99.2)		5; US	na
Ab (LFIA, POC)	Healgen, COVID-19 IgG/IgM Rapid Test Cassette	IgG or IgM	SENS	unk	27/27	100.0 (87.5-100.0)		5; US	na
Ab (LFIA, POC)	Healgen, COVID-19 IgG/IgM Rapid Test Cassette	IgG or IgM	SPEC	na	68/70	97.1 (90.2-99.2)		5; US	na
Ab (LFIA, POC)	Healgen, COVID-19 IgG/IgM Rapid Test Cassette	IgM	SENS	unk	27/27	100.0 (87.5-100.0)		5; US	na
Ab (LFIA, POC)	Healgen, COVID-19 IgG/IgM Rapid Test Cassette	IgM	SPEC	na	70/70	100.0 (94.8-100.0)		5; US	na
Ab (LFIA, POC)	Hefei Biohit Healthcare, SARS-CoV-2 IgM/IgG Antibody Test Kit	IgG	SENS	unk	28/29	96.6 (82.8-99.4)		5; US	na
Ab (LFIA, POC)	Hefei Biohit Healthcare, SARS-CoV-2 IgM/IgG Antibody Test Kit	IgG	SPEC	na	66/70	94.3 (86.2-97.8)		5; US	na
Ab (LFIA, POC)	Hefei Biohit Healthcare, SARS-CoV-2 IgM/IgG Antibody Test Kit	IgG or IgM	SENS	unk	28/29	96.6 (82.8-99.4)		5; US	na
Ab (LFIA, POC)	Hefei Biohit Healthcare, SARS-CoV-2 IgM/IgG Antibody Test Kit	IgG or IgM	SPEC	na	66/70	94.3 (86.2-97.8)		5; US	na
Ab (LFIA, POC)	Hefei Biohit Healthcare, SARS-CoV-2 IgM/IgG Antibody Test Kit	IgM	SENS	unk	28/29	96.6 (82.8-99.4)		5; US	na
Ab (LFIA, POC)	Hefei Biohit Healthcare, SARS-CoV-2 IgM/IgG Antibody Test Kit	IgM	SPEC	na	66/70	94.3 (86.2-97.8)		5; US	na
Ab (ELISA)	ID.Vet, ID screen SARS-CoV-2-N IgG Indirect	IgG	SENS	hospitalised	14/15	93.3 (70.2-98.8)		284; FR	na
Ab (ELISA)	ID.Vet, ID screen SARS-	IgG	SPEC	na	20/20	100.0		284; FR	na

	CoV-2-N IgG Indirect					(83.9-100.0)			
Ab (ELISA)	InBios International, SCoV-2 Detect IgG ELISA	IgG	SENS	unk	29/29	100.0 (88.3-100.0)		5; US	na
Ab (ELISA)	InBios International, SCoV-2 Detect IgG ELISA	IgG	SPEC	na	70/70	100.0 (94.8-100.0)		5; US	na
Ab (ELISA)	InBios International, SCoV-2 Detect IgM ELISA	IgM	SENS	unk	28/29	96.6 (82.8-99.4)		5; US	na
Ab (ELISA)	InBios International, SCoV-2 Detect IgM ELISA	IgM	SPEC	na	69/70	98.6 (92.3-99.7)		5; US	na
Ab (LFIA, POC)	InTec Products, Rapid SARS-CoV-2 antibody (IgM/IgG) Test	IgG	SENS	mild/asymptomatic	44/63	69.8 (57.6-79.8)		4; NL	na
Ab (LFIA, POC)	InTec Products, Rapid SARS-CoV-2 antibody (IgM/IgG) Test	IgG	SENS	hospitalised	31/31	100.0 (89.0-100.0)		4; NL	na
Ab (LFIA, POC)	InTec Products, Rapid SARS-CoV-2 antibody (IgM/IgG) Test	IgG	SPEC	na	107/112	95.5 (90.0-98.1)		4; NL	na
Ab (LFIA, POC)	InTec Products, Rapid SARS-CoV-2 antibody (IgM/IgG) Test	IgG or IgM	SPEC	na	83/98	84.7 (76.3-90.5)		75; NL	na
Ab (LFIA, POC)	InTec Products, Rapid SARS-CoV-2 antibody (IgM/IgG) Test	IgM	SENS	mild/asymptomatic	19/63	30.2 (20.2-42.4)		4; NL	na
Ab (LFIA, POC)	InTec Products, Rapid SARS-CoV-2 antibody (IgM/IgG) Test	IgM	SENS	hospitalised	NaN/NaN	NaN (NaN-NaN)		4;	na
Ab (LFIA, POC)	Innovita Biological Technology, 2019-nCoV Ab Test (Colloidal Gold)	IgG	SENS	hospitalised	53/61	86.9 (76.2-93.2)	16.1	44, 143; CA, JP	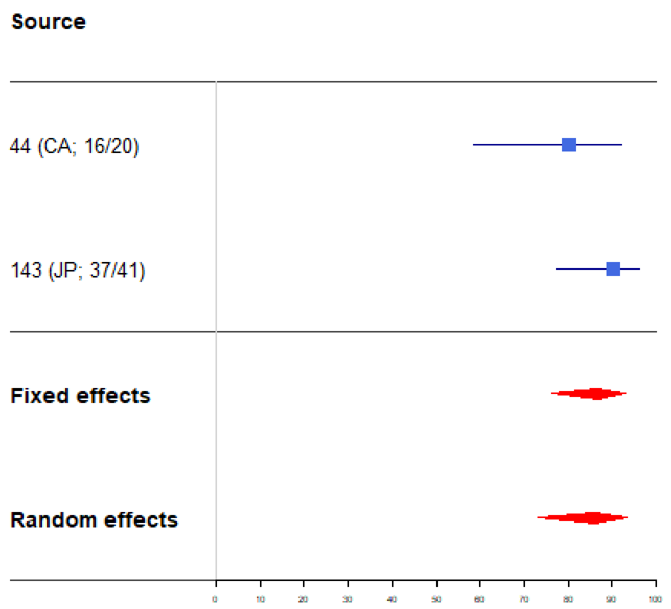
Ab (LFIA, POC)	Innovita Biological Technology, 2019-nCoV Ab Test (Colloidal Gold)	IgG	SENS	unk	13/19	68.4 (46.0-84.6)		297; US	na
Ab (LFIA, POC)	Innovita Biological Technology, 2019-nCoV Ab Test (Colloidal Gold)	IgG	SPEC	na	258/258	100.0 (98.5-100.0)	0.0	44, 143, 297; CA, JP, US	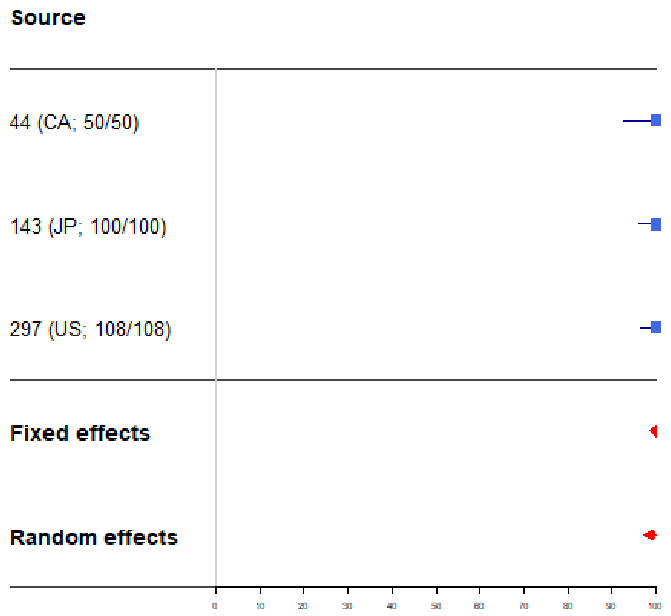
Ab (LFIA, POC)	Innovita Biological Technology, 2019-nCoV Ab Test (Colloidal Gold)	IgG or IgM	SENS	hospitalised	17/20	85.0 (64.0-94.8)		44; CA	na
Ab (LFIA, POC)	Innovita Biological Technology, 2019-nCoV Ab Test (Colloidal Gold)	IgG or IgM	SENS	unk	14/20	70.0 (48.1-85.5)		297; US	na
Ab (LFIA, POC)	Innovita Biological Technology, 2019-nCoV Ab Test (Colloidal Gold)	IgG or IgM	SPEC	na	154/158	97.5 (93.7-99.0)	0.0	44, 297; CA, US	

									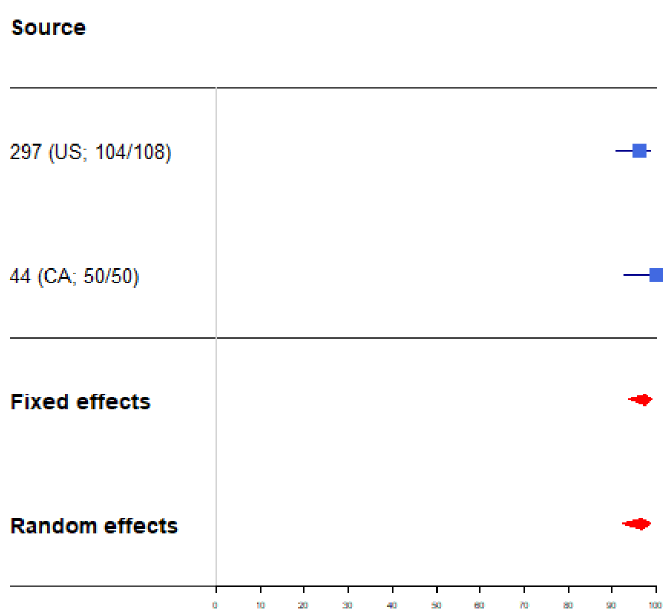
Ab (LFIA, POC)	Innovita Biological Technology, 2019-nCoV Ab Test (Colloidal Gold)	IgM	SENS	hospitalised	46/61	75.4 (63.3-84.5)	89.0	44, 143; CA, JP	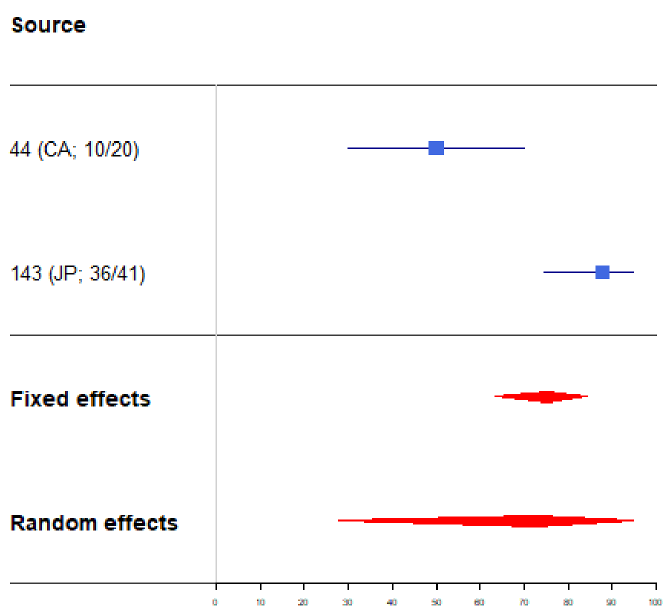
Ab (LFIA, POC)	Innovita Biological Technology, 2019-nCoV Ab Test (Colloidal Gold)	IgM	SENS	unk	5/20	25.0 (11.2-46.9)		297; US	na
Ab (LFIA, POC)	Innovita Biological Technology, 2019-nCoV Ab Test (Colloidal Gold)	IgM	SPEC	na	254/258	98.4 (96.1-99.4)	28.0	44, 143, 297; CA, JP, US	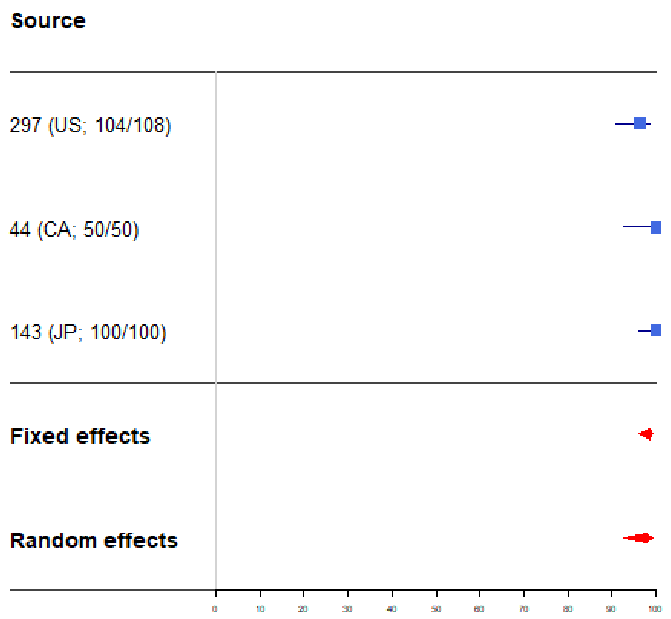
Ab (LFIA, POC)	Invenio Medical, COVID-19 IgG/IgM Ab Rapid Test	IgG	SENS	unk	21/27	77.8 (59.2-89.4)		5; US	na
Ab (LFIA, POC)	Invenio Medical, COVID-19 IgG/IgM Ab Rapid Test	IgG	SPEC	na	70/70	100.0 (94.8-100.0)		5; US	na
Ab (LFIA, POC)	Invenio Medical, COVID-19 IgG/IgM Ab Rapid Test	IgG or IgM	SENS	unk	21/27	77.8 (59.2-89.4)		5; US	na
Ab (LFIA, POC)	Invenio Medical, COVID-19 IgG/IgM Ab Rapid Test	IgG or IgM	SPEC	na	70/70	100.0 (94.8-100.0)		5; US	na
Ab (LFIA, POC)	Invenio Medical, COVID-19 IgG/IgM Ab Rapid Test	IgM	SENS	unk	20/27	74.1 (55.3-86.8)		5; US	na
Ab (LFIA, POC)	Invenio Medical, COVID-19 IgG/IgM Ab Rapid Test	IgM	SPEC	na	70/70	100.0 (94.8-100.0)		5; US	na
Ab (LFIA, POC)	Jiangsu Bioperfectus Technologies, PerfectPOC Novel Corona Virus (SARS-CoV-2) IgM/IgG Rapid Test Kit	IgG	SENS	unk	23/29	79.3 (61.6-90.2)		297; US	na

Ab (LFIA, POC)	Jiangsu Bioperfectus Technologies, PerfectPOC Novel Corona Virus (SARS-CoV-2) IgM/IgG Rapid Test Kit	IgG	SPEC	na	102/104	98.1 (93.3-99.5)		297; US	na
Ab (LFIA, POC)	Jiangsu Bioperfectus Technologies, PerfectPOC Novel Corona Virus (SARS-CoV-2) IgM/IgG Rapid Test Kit	IgG or IgM	SENS	unk	27/29	93.1 (78.0-98.1)		297; US	na
Ab (LFIA, POC)	Jiangsu Bioperfectus Technologies, PerfectPOC Novel Corona Virus (SARS-CoV-2) IgM/IgG Rapid Test Kit	IgG or IgM	SPEC	na	99/104	95.2 (89.2-97.9)		297; US	na
Ab (LFIA, POC)	Jiangsu Bioperfectus Technologies, PerfectPOC Novel Corona Virus (SARS-CoV-2) IgM/IgG Rapid Test Kit	IgM	SENS	unk	26/29	89.7 (73.6-96.4)		297; US	na
Ab (LFIA, POC)	Jiangsu Bioperfectus Technologies, PerfectPOC Novel Corona Virus (SARS-CoV-2) IgM/IgG Rapid Test Kit	IgM	SPEC	na	101/104	97.1 (91.9-99.0)		297; US	na
Ab (LFIA, POC)	Jiangsu Dablood Pharmaceutical, COVID-19 IgM/IgG One Step Rapid Test	IgG	SENS	unk	29/29	100.0 (88.3-100.0)		5; US	na
Ab (LFIA, POC)	Jiangsu Dablood Pharmaceutical, COVID-19 IgM/IgG One Step Rapid Test	IgG	SPEC	na	56/70	80.0 (69.2-87.7)		5; US	na
Ab (LFIA, POC)	Jiangsu Dablood Pharmaceutical, COVID-19 IgM/IgG One Step Rapid Test	IgG or IgM	SENS	unk	29/29	100.0 (88.3-100.0)		5; US	na
Ab (LFIA, POC)	Jiangsu Dablood Pharmaceutical, COVID-19 IgM/IgG One Step Rapid Test	IgG or IgM	SPEC	na	55/70	78.6 (67.6-86.6)		5; US	na
Ab (LFIA, POC)	Jiangsu Dablood Pharmaceutical, COVID-19 IgM/IgG One Step Rapid Test	IgM	SENS	unk	27/29	93.1 (78.0-98.1)		5; US	na
Ab (LFIA, POC)	Jiangsu Dablood Pharmaceutical, COVID-19 IgM/IgG One Step Rapid Test	IgM	SPEC	na	68/70	97.1 (90.2-99.2)		5; US	na
Ab (LFIA, POC)	Jiangsu Medomics medical technology, A Rapid IgM-IgG Combined Antibody Test Kit for SARSCoV-2	IgG	SENS	mild/asymptomatic	20/23	87.0 (67.9-95.5)		278; NO	na
Ab (LFIA, POC)	Jiangsu Medomics medical technology, A Rapid IgM-IgG Combined Antibody Test Kit for SARSCoV-2	IgM	SENS	mild/asymptomatic	12/20	60.0 (38.7-78.1)		278; NO	na
Ab (LFIA, POC)	LabOn Time, 2019-n-CoV IgG/IgM rapid test cassette	IgG	SPEC	na	72/72	100.0 (94.9-100.0)		177; BE	na
Ab (LFIA, POC)	LabOn Time, 2019-n-CoV IgG/IgM rapid test cassette	IgG or IgM	SENS	hospitalised	31/33	93.9 (80.4-98.3)		177; BE	na
Ab (LFIA, POC)	LabOn Time, 2019-n-CoV IgG/IgM rapid test cassette	IgG or IgM	SPEC	na	72/72	100.0 (94.9-100.0)		177; BE	na
Ab (LFIA, POC)	LabOn Time, 2019-n-CoV IgG/IgM rapid test cassette	IgM	SPEC	na	72/72	100.0 (94.9-100.0)		177; BE	na
Ab (LFIA, POC)	Liming Bio, StrongStep COVID-19 IgG/IgM Combo Test	IgG	SENS	hospitalised	23/23	100.0 (85.7-100.0)		242; BE	na
Ab (LFIA, POC)	Liming Bio, StrongStep COVID-19 IgG/IgM Combo Test	IgG	SPEC	na	102/103	99.0 (94.7-99.8)		242; BE	na
Ab (LFIA, POC)	Liming Bio, StrongStep COVID-19 IgG/IgM Combo Test	IgG or IgM	SENS	hospitalised	23/23	100.0 (85.7-100.0)		242; BE	na
Ab (LFIA, POC)	Liming Bio, StrongStep COVID-19 IgG/IgM Combo Test	IgG or IgM	SPEC	na	101/103	98.1 (93.2-99.5)		242; BE	na
Ab (LFIA, POC)	Liming Bio, StrongStep COVID-19 IgG/IgM Combo Test	IgM	SENS	hospitalised	13/23	56.5 (36.8-74.4)		242; BE	na
Ab (LFIA, POC)	Liming Bio, StrongStep COVID-19 IgG/IgM Combo Test	IgM	SPEC	na	102/103	99.0 (94.7-99.8)		242; BE	na
Ab (ELISA)	Lizhu, Recombinant nucleocapsid (rN) protein-based ELISA kit	IgG	SENS	hospitalised	89/100	89.0 (81.4-93.7)		158; CN	na
Ab (ELISA)	Lizhu, Recombinant nucleocapsid (rN) protein-based ELISA kit	IgG or IgM	SENS	hospitalised	95/100	95.0 (88.8-97.8)		158; CN	na
Ab (ELISA)	Lizhu, Recombinant nucleocapsid (rN) protein-based ELISA kit	IgM	SENS	hospitalised	80/100	80.0 (71.1-86.7)		158; CN	na
Ab (LFIA, POC)	LumiQuick Diagnostics, Quick Profile 2019-nCoV	IgG	SENS	unk	28/29	96.6 (82.8-99.4)		5; US	na

	IgG/IgM Test Card								
Ab (LFIA, POC)	LumiQuick Diagnostics, Quick Profile 2019-nCoV IgG/IgM Test Card	IgG	SPEC	na	67/70	95.7 (88.1-98.5)		5; US	na
Ab (LFIA, POC)	LumiQuick Diagnostics, Quick Profile 2019-nCoV IgG/IgM Test Card	IgG or IgM	SENS	unk	28/29	96.6 (82.8-99.4)		5; US	na
Ab (LFIA, POC)	LumiQuick Diagnostics, Quick Profile 2019-nCoV IgG/IgM Test Card	IgG or IgM	SPEC	na	63/70	90.0 (80.8-95.1)		5; US	na
Ab (LFIA, POC)	LumiQuick Diagnostics, Quick Profile 2019-nCoV IgG/IgM Test Card	IgM	SENS	unk	25/29	86.2 (69.4-94.5)		5; US	na
Ab (LFIA, POC)	LumiQuick Diagnostics, Quick Profile 2019-nCoV IgG/IgM Test Card	IgM	SPEC	na	66/70	94.3 (86.2-97.8)		5; US	na
Ab (LFIA, POC)	MEDsan, COVID-19 IgM/IgG Rapid Test	IgG	SENS	hospitalised	26/27	96.3 (81.7-99.3)		15; CH	na
Ab (LFIA, POC)	MEDsan, COVID-19 IgM/IgG Rapid Test	IgG	SENS	unk	26/29	89.7 (73.6-96.4)		5; US	na
Ab (LFIA, POC)	MEDsan, COVID-19 IgM/IgG Rapid Test	IgG	SPEC	na	114/120	95.0 (89.5-97.7)	29.8	5, 15; CH, US	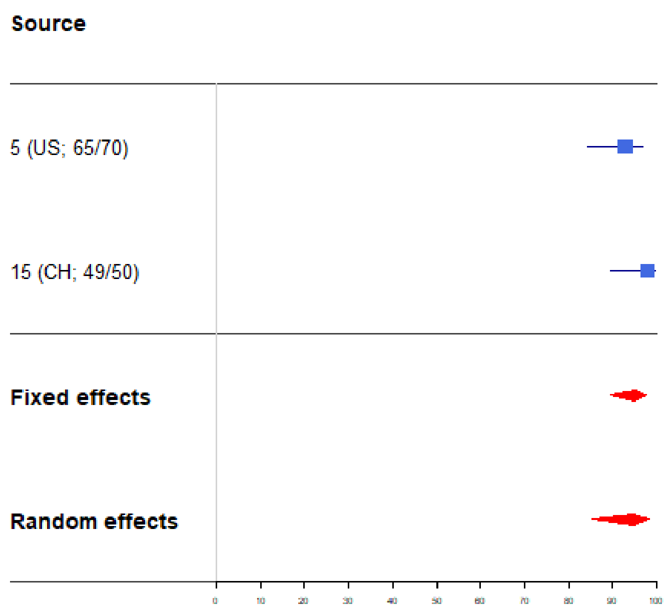
Ab (LFIA, POC)	MEDsan, COVID-19 IgM/IgG Rapid Test	IgG or IgM	SENS	unk	118/128	92.2 (86.2-95.7)	0.0	5, 296; AT, US	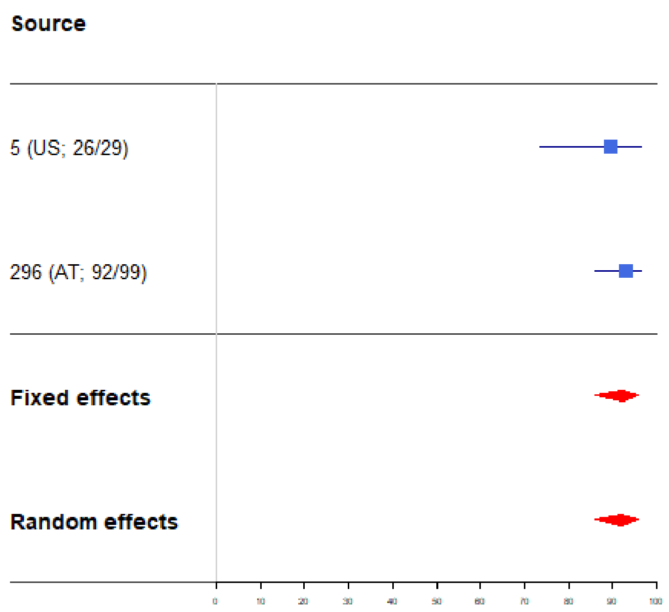
Ab (LFIA, POC)	MEDsan, COVID-19 IgM/IgG Rapid Test	IgG or IgM	SPEC	na	64/70	91.4 (82.5-96.0)		5; US	na
Ab (LFIA, POC)	MEDsan, COVID-19 IgM/IgG Rapid Test	IgM	SENS	unk	26/29	89.7 (73.6-96.4)		5; US	na
Ab (LFIA, POC)	MEDsan, COVID-19 IgM/IgG Rapid Test	IgM	SPEC	na	67/70	95.7 (88.1-98.5)		5; US	na
Ab (LFIA, POC)	Maccura Biotechnology, SARS-CoV2 LFIA IgG/IgM	IgG or IgM	SENS	unk	15/22	68.2 (47.3-83.6)		25; CH	na
Ab (LFIA, POC)	Maccura Biotechnology, SARS-CoV2 LFIA IgG/IgM	IgG or IgM	SPEC	na	90/100	90.0 (82.6-94.5)		25; CH	na
Ab (ELISA)	Mediagnost, Anti-SARS-CoV-2 ELISA	IgG	SENS	unk	47/73	64.4 (52.9-74.4)		255; DE	na
Ab (LFIA, POC)	Megna Health, Megna Rapid COVID-19 IgM/IgG Combo Test Kit	IgG	SENS	unk	29/29	100.0 (88.3-100.0)		5; US	na
Ab (LFIA, POC)	Megna Health, Megna Rapid COVID-19 IgM/IgG Combo Test Kit	IgG	SPEC	na	68/70	97.1 (90.2-99.2)		5; US	na
Ab (LFIA, POC)	Megna Health, Megna Rapid COVID-19 IgM/IgG Combo Test Kit	IgG or IgM	SENS	unk	29/29	100.0 (88.3-100.0)		5; US	na
Ab (LFIA, POC)	Megna Health, Megna Rapid COVID-19 IgM/IgG Combo Test Kit	IgG or IgM	SPEC	na	66/70	94.3 (86.2-97.8)		5; US	na
Ab (LFIA, POC)	Megna Health, Megna Rapid COVID-19 IgM/IgG Combo Test Kit	IgM	SENS	unk	24/29	82.8 (65.5-92.4)		5; US	na

Ab (LFIA, POC)	Megna Health, Megna Rapid COVID-19 IgM/IgG Combo Test Kit	IgM	SPEC	na	68/70	97.1 (90.2-99.2)		5; US	na
Ab (ELISA)	Mikrogen Diagnostik, recomWell SARS-CoV-2 IgG	IgG	SENS	mild/asymptomatic	43/55	78.2 (65.6-87.1)		3; NL	na
Ab (ELISA)	Mikrogen Diagnostik, recomWell SARS-CoV-2 IgG	IgG	SENS	hospitalised	1/1	100.0 (20.7-100.0)		3; NL	na
Ab (ELISA)	Mikrogen Diagnostik, recomWell SARS-CoV-2 IgG	IgG	SENS	unk	40/40	100.0 (91.2-100.0)		285; BE	na
Ab (ELISA)	Mikrogen Diagnostik, recomWell SARS-CoV-2 IgG	IgG	SPEC	na	429/445	96.4 (94.2-97.8)	0.0	3, 269, 285; BE, DE, NL	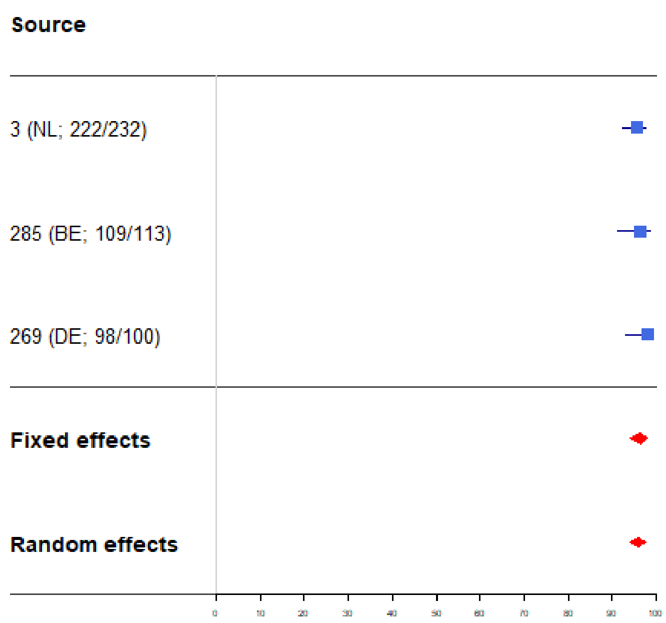
Ab (LFIA, POC)	Multi-G, COVID-19 IgG/IgM dual lane Ab Test Cassette	IgG	SENS	hospitalised	48/49	98.0 (89.3-99.6)		242; BE	na
Ab (LFIA, POC)	Multi-G, COVID-19 IgG/IgM dual lane Ab Test Cassette	IgG	SPEC	na	139/142	97.9 (94.0-99.3)		242; BE	na
Ab (LFIA, POC)	Multi-G, COVID-19 IgG/IgM dual lane Ab Test Cassette	IgG or IgM	SENS	hospitalised	48/49	98.0 (89.3-99.6)		242; BE	na
Ab (LFIA, POC)	Multi-G, COVID-19 IgG/IgM dual lane Ab Test Cassette	IgG or IgM	SPEC	na	139/142	97.9 (94.0-99.3)		242; BE	na
Ab (LFIA, POC)	Multi-G, COVID-19 IgG/IgM dual lane Ab Test Cassette	IgM	SENS	hospitalised	26/49	53.1 (39.4-66.3)		242; BE	na
Ab (LFIA, POC)	Multi-G, COVID-19 IgG/IgM dual lane Ab Test Cassette	IgM	SPEC	na	142/142	100.0 (97.4-100.0)		242; BE	na
Ab (LFIA, POC)	Multi-G, COVID-19 IgG/IgM single lane Ab Test Cassette	IgG	SENS	hospitalised	48/49	98.0 (89.3-99.6)		242; BE	na
Ab (LFIA, POC)	Multi-G, COVID-19 IgG/IgM single lane Ab Test Cassette	IgG	SPEC	na	139/142	97.9 (94.0-99.3)		242; BE	na
Ab (LFIA, POC)	Multi-G, COVID-19 IgG/IgM single lane Ab Test Cassette	IgG or IgM	SENS	hospitalised	48/49	98.0 (89.3-99.6)		242; BE	na
Ab (LFIA, POC)	Multi-G, COVID-19 IgG/IgM single lane Ab Test Cassette	IgG or IgM	SPEC	na	129/142	90.8 (85.0-94.6)		242; BE	na
Ab (LFIA, POC)	Multi-G, COVID-19 IgG/IgM single lane Ab Test Cassette	IgM	SENS	hospitalised	26/49	53.1 (39.4-66.3)		242; BE	na
Ab (LFIA, POC)	Multi-G, COVID-19 IgG/IgM single lane Ab Test Cassette	IgM	SPEC	na	132/142	93.0 (87.5-96.1)		242; BE	na
Ab (LFIA, POC)	NG Biotech, NG-Test IgG-IgM COVID-19	IgG	SENS	unk	82/82	100.0 (95.5-100.0)		208; FR	na
Ab (LFIA, POC)	NG Biotech, NG-Test IgG-IgM COVID-19	IgG	SPEC	na	93/95	97.9 (92.6-99.4)		208; FR	na
Ab (LFIA, POC)	NG Biotech, NG-Test IgG-IgM COVID-19	IgG or IgM	SENS	unk	82/82	100.0 (95.5-100.0)		208; FR	na
Ab (LFIA, POC)	NG Biotech, NG-Test IgG-IgM COVID-19	IgG or IgM	SPEC	na	91/95	95.8 (89.7-98.4)		208; FR	na
Ab (LFIA, POC)	NG Biotech, NG-Test IgG-IgM COVID-19	IgM	SENS	unk	82/82	100.0 (95.5-100.0)		208; FR	na
Ab (LFIA, POC)	NG Biotech, NG-Test IgG-IgM COVID-19	IgM	SPEC	na	91/95	95.8 (89.7-98.4)		208; FR	na
Ab (LFIA, POC)	NTBIO Diagnostics, COVID-19 IgG/IgM Antibody Test Cassette	IgG	SENS	hospitalised	25/27	92.6 (76.6-97.9)		15; CH	na
Ab (LFIA, POC)	NTBIO Diagnostics, COVID-19 IgG/IgM Antibody Test Cassette	IgG	SPEC	na	49/50	98.0 (89.5-99.6)		15; CH	na
Ab (LFIA, POC)	Nanjing Jiangsu Superbio Biomedical, SARS-CoV-2 (COVID-19) IgM/IgG Antibody Fast Detection Kit (Colloidal Gold)	IgG	SENS	unk	29/29	100.0 (88.3-100.0)		5; US	na

Ab (LFIA, POC)	Nanjing Jiangsu Superbio Biomedical, SARS-CoV-2 (COVID-19) IgM/IgG Antibody Fast Detection Kit (Colloidal Gold)	IgG	SPEC	na	59/70	84.3 (74.0-91.0)		5; US	na
Ab (LFIA, POC)	Nanjing Jiangsu Superbio Biomedical, SARS-CoV-2 (COVID-19) IgM/IgG Antibody Fast Detection Kit (Colloidal Gold)	IgG or IgM	SENS	unk	29/29	100.0 (88.3-100.0)		5; US	na
Ab (LFIA, POC)	Nanjing Jiangsu Superbio Biomedical, SARS-CoV-2 (COVID-19) IgM/IgG Antibody Fast Detection Kit (Colloidal Gold)	IgG or IgM	SPEC	na	58/70	82.9 (72.4-89.9)		5; US	na
Ab (LFIA, POC)	Nanjing Jiangsu Superbio Biomedical, SARS-CoV-2 (COVID-19) IgM/IgG Antibody Fast Detection Kit (Colloidal Gold)	IgM	SENS	unk	11/29	37.9 (22.7-56.0)		5; US	na
Ab (LFIA, POC)	Nanjing Jiangsu Superbio Biomedical, SARS-CoV-2 (COVID-19) IgM/IgG Antibody Fast Detection Kit (Colloidal Gold)	IgM	SPEC	na	69/70	98.6 (92.3-99.7)		5; US	na
Ab (LFIA, POC)	Nanjing Vazyme Medical Technology, 2019-nCoV IgG / IgM Detection Kit (Colloidal Gold-Based)	IgG	SENS	hospitalised	NaN/NaN	NaN (NaN-NaN)		71;	na
Ab (LFIA, POC)	Nanjing Vazyme Medical Technology, 2019-nCoV IgG / IgM Detection Kit (Colloidal Gold-Based)	IgG	SENS	unk	28/29	96.6 (82.8-99.4)		5; US	na
Ab (LFIA, POC)	Nanjing Vazyme Medical Technology, 2019-nCoV IgG / IgM Detection Kit (Colloidal Gold-Based)	IgG	SPEC	na	62/70	88.6 (79.0-94.1)		5; US	na
Ab (LFIA, POC)	Nanjing Vazyme Medical Technology, 2019-nCoV IgG / IgM Detection Kit (Colloidal Gold-Based)	IgG or IgM	SENS	unk	28/29	96.6 (82.8-99.4)		5; US	na
Ab (LFIA, POC)	Nanjing Vazyme Medical Technology, 2019-nCoV IgG / IgM Detection Kit (Colloidal Gold-Based)	IgG or IgM	SPEC	na	47/70	67.1 (55.5-77.0)		5; US	na
Ab (LFIA, POC)	Nanjing Vazyme Medical Technology, 2019-nCoV IgG / IgM Detection Kit (Colloidal Gold-Based)	IgM	SENS	hospitalised	NaN/NaN	NaN (NaN-NaN)		71;	na
Ab (LFIA, POC)	Nanjing Vazyme Medical Technology, 2019-nCoV IgG / IgM Detection Kit (Colloidal Gold-Based)	IgM	SENS	unk	19/29	65.5 (47.3-80.1)		5; US	na
Ab (LFIA, POC)	Nanjing Vazyme Medical Technology, 2019-nCoV IgG / IgM Detection Kit (Colloidal Gold-Based)	IgM	SPEC	na	54/70	77.1 (66.0-85.4)		5; US	na
Ab (ELISA)	NovaTec Immundiagnostica, NovaLisa SARS-CoV-2 IgA ELISA	IgA	SENS	mild/asymptomatic	NaN/NaN	NaN (NaN-NaN)		3;	na
Ab (ELISA)	NovaTec Immundiagnostica, NovaLisa SARS-CoV-2 IgA ELISA	IgA	SENS	hospitalised	55/62	88.7 (78.5-94.4)	35.4	242, 282; BE(2)	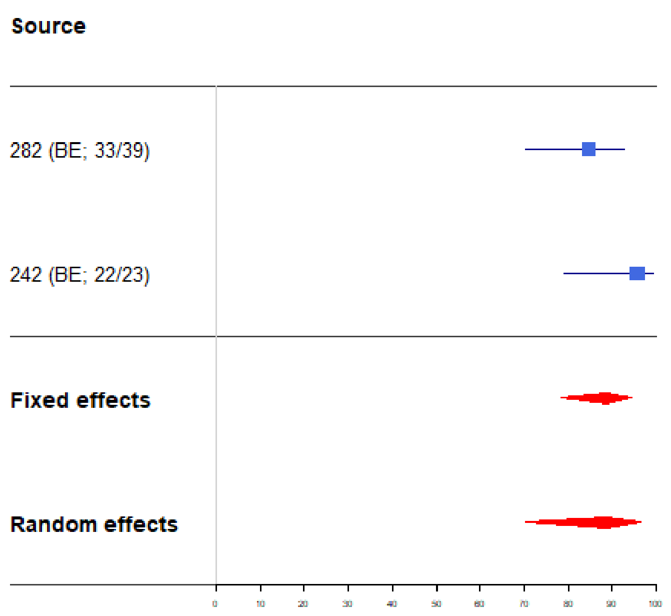
Ab (ELISA)	NovaTec Immundiagnostica, NovaLisa SARS-CoV-2 IgA ELISA	IgA	SENS	unk	NaN/NaN	NaN (NaN-NaN)		216;	na
Ab (ELISA)	NovaTec Immundiagnostica, NovaLisa SARS-CoV-2 IgA ELISA	IgA	SPEC	na	279/293	95.2 (92.1-97.1)	62.5	3, 216, 242, 282; BE(2), IT, NL	

									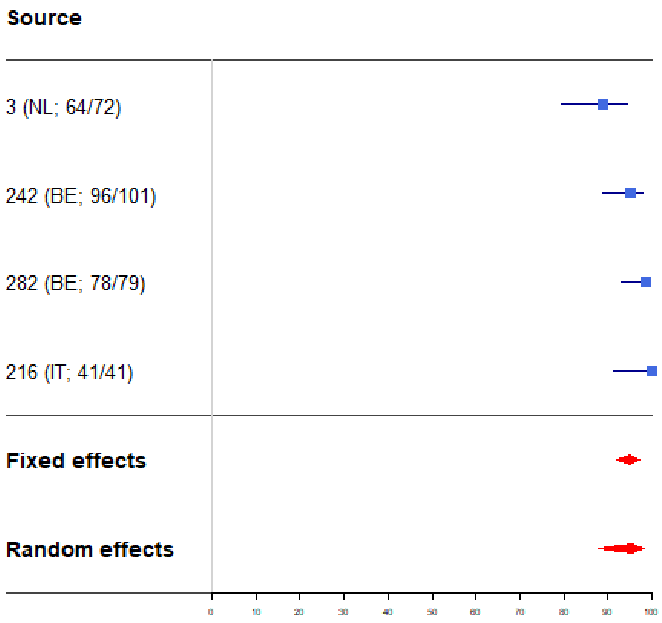
Ab (ELISA)	NovaTec Immundiagnostica, NovaLisa SARS-CoV-2 IgG ELISA	IgG	SENS	mild/asymptomatic	NaN/NaN	NaN (NaN-NaN)		3;	na
Ab (ELISA)	NovaTec Immundiagnostica, NovaLisa SARS-CoV-2 IgG ELISA	IgG	SENS	hospitalised	57/62	91.9 (82.5-96.5)	0.0	242, 282; BE(2)	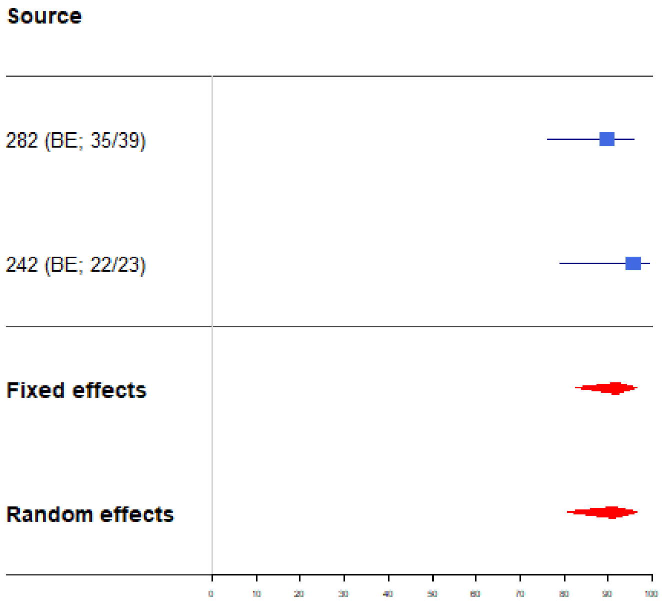
Ab (ELISA)	NovaTec Immundiagnostica, NovaLisa SARS-CoV-2 IgG ELISA	IgG	SENS	unk	50/73	68.5 (57.1-78.0)		255; DE	na
Ab (ELISA)	NovaTec Immundiagnostica, NovaLisa SARS-CoV-2 IgG ELISA	IgG	SPEC	na	285/293	97.3 (94.7-98.6)	0.0	3, 216, 242, 282; BE(2), IT, NL	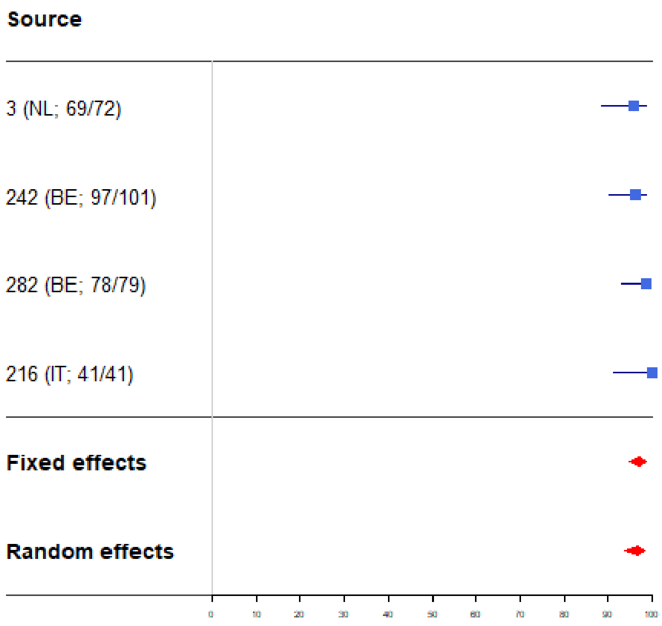
Ab (ELISA)	NovaTec Immundiagnostica, NovaLisa SARS-CoV-2 IgM ELISA	IgM	SENS	mild/asymptomatic	NaN/NaN	NaN (NaN-NaN)		3;	na
Ab (ELISA)	NovaTec Immundiagnostica, NovaLisa SARS-CoV-2 IgM ELISA	IgM	SENS	hospitalised	27/62	43.5 (31.9-55.9)	84.9	242, 282; BE(2)	

									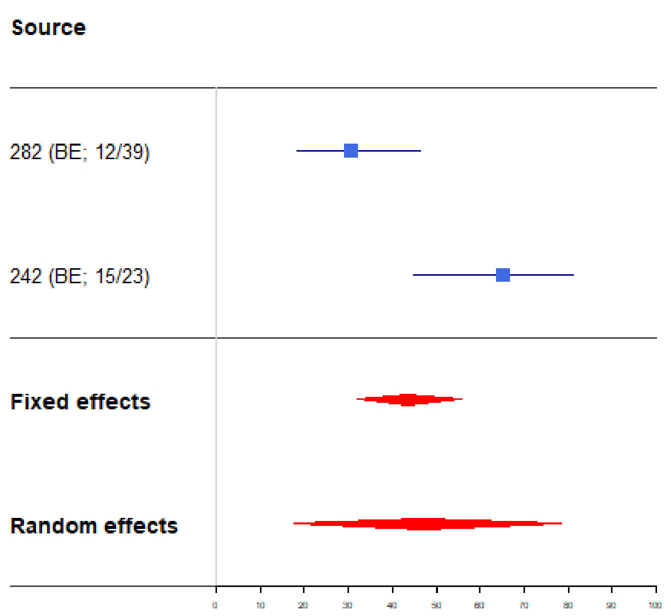
Ab (ELISA)	NovaTec Immundiagnostica, NovaLisa SARS-CoV-2 IgM ELISA	IgM	SENS	unk	NaN/NaN	NaN (NaN-NaN)		216;	na
Ab (ELISA)	NovaTec Immundiagnostica, NovaLisa SARS-CoV-2 IgM ELISA	IgM	SPEC	na	290/293	99.0 (97.0-99.7)	0.0	3, 216, 242, 282; BE(2), IT, NL	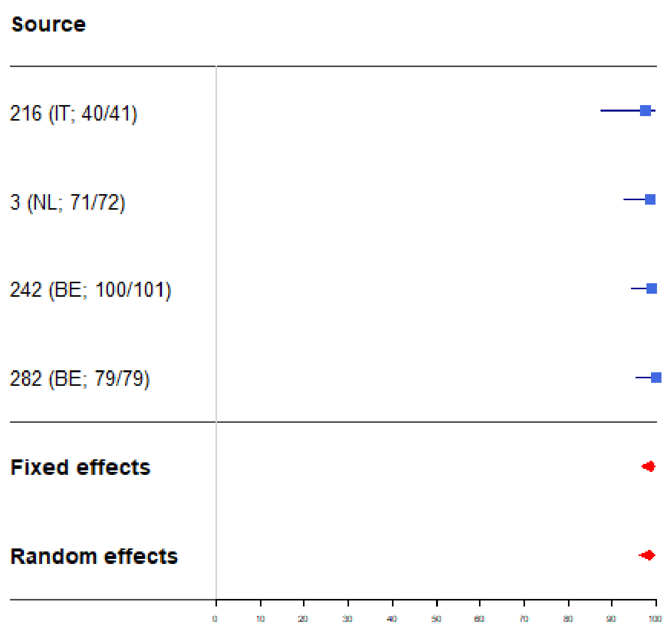
Ab (CLIA)	Ortho Clinical Diagnostics, VITROS Immunodiagnostic Products Anti-SARS-CoV-2 IgG	IgG	SENS	mild/asymptomatic	22/23	95.7 (79.0-99.2)		277; US	na
Ab (CLIA)	Ortho Clinical Diagnostics, VITROS Immunodiagnostic Products Anti-SARS-CoV-2 IgG	IgG	SENS	hospitalised	61/61	100.0 (94.1-100.0)		277; US	na
Ab (CLIA)	Ortho Clinical Diagnostics, VITROS Immunodiagnostic Products Anti-SARS-CoV-2 IgG	IgG	SENS	unk	212/227	93.4 (89.4-96.0)	0.0	9, 83; DK, UK	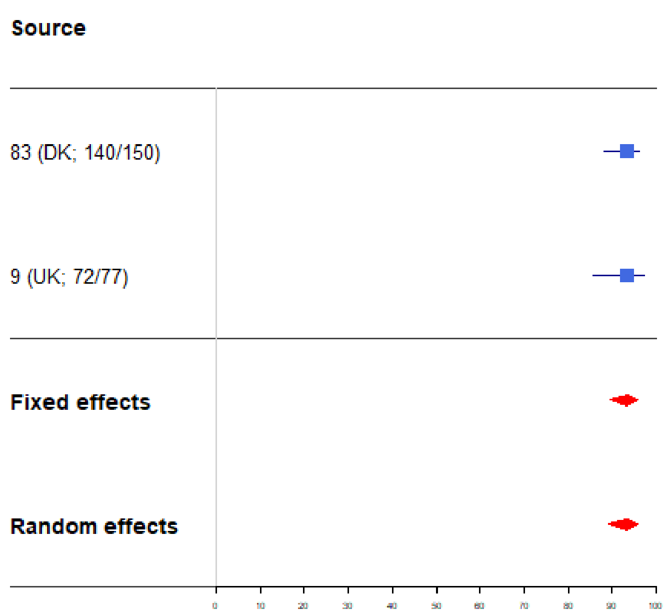
Ab (CLIA)	Ortho Clinical Diagnostics, VITROS Immunodiagnostic Products Anti-SARS-CoV-2 IgG	IgG	SPEC	na	1416/1420	99.7 (99.3-99.9)	0.0	9, 83, 277; DK, UK, US	

									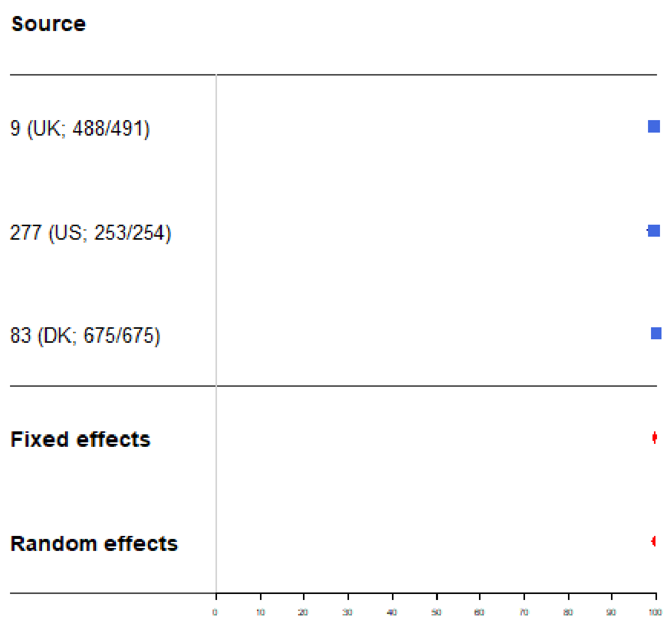
Ab (CLIA)	Ortho Clinical Diagnostics, VITROS Immunodiagnostic Products Anti-SARS-CoV-2 Total Ab	total Ab	SENS	unk	143/150	95.3 (90.7-97.7)		83; DK	na
Ab (CLIA)	Ortho Clinical Diagnostics, VITROS Immunodiagnostic Products Anti-SARS-CoV-2 Total Ab	total Ab	SPEC	na	732/732	100.0 (99.5-100.0)	33.8	74, 83; DK, US	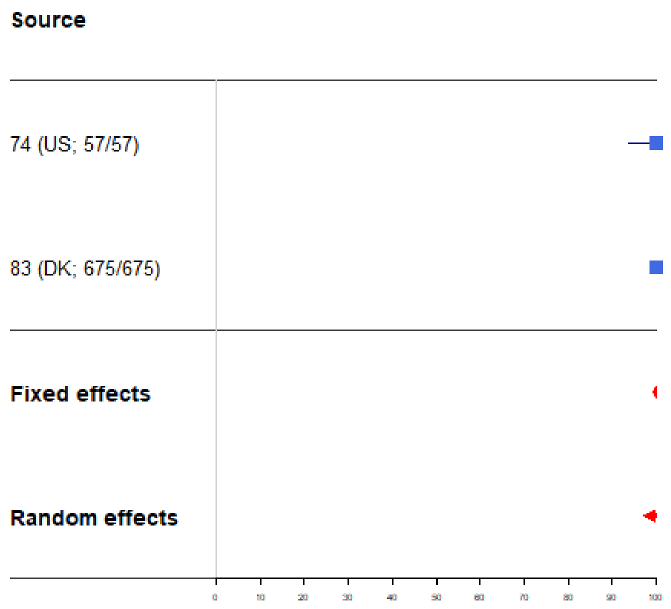
Ab (LFIA, POC)	PCL, PCL COVID19 IgG/IgM Rapid Gold	IgG	SENS	hospitalised	NaN/NaN	NaN (NaN-NaN)		47;	na
Ab (LFIA, POC)	PCL, PCL COVID19 IgG/IgM Rapid Gold	IgG	SENS	unk	23/29	79.3 (61.6-90.2)		5; US	na
Ab (LFIA, POC)	PCL, PCL COVID19 IgG/IgM Rapid Gold	IgG	SPEC	na	70/70	100.0 (94.8-100.0)		5; US	na
Ab (LFIA, POC)	PCL, PCL COVID19 IgG/IgM Rapid Gold	IgG or IgM	SENS	hospitalised	NaN/NaN	NaN (NaN-NaN)		47;	na
Ab (LFIA, POC)	PCL, PCL COVID19 IgG/IgM Rapid Gold	IgG or IgM	SENS	unk	28/29	96.6 (82.8-99.4)		5; US	na
Ab (LFIA, POC)	PCL, PCL COVID19 IgG/IgM Rapid Gold	IgG or IgM	SPEC	na	70/70	100.0 (94.8-100.0)		5; US	na
Ab (LFIA, POC)	PCL, PCL COVID19 IgG/IgM Rapid Gold	IgM	SENS	hospitalised	NaN/NaN	NaN (NaN-NaN)		47;	na
Ab (LFIA, POC)	PCL, PCL COVID19 IgG/IgM Rapid Gold	IgM	SENS	unk	27/29	93.1 (78.0-98.1)		5; US	na
Ab (LFIA, POC)	PCL, PCL COVID19 IgG/IgM Rapid Gold	IgM	SPEC	na	70/70	100.0 (94.8-100.0)		5; US	na
Ab (LFIA, POC)	PRIMA Lab, PRIMA COVID-19 IgG/IgM Rapid Test	IgG	SENS	hospitalised	23/23	100.0 (85.7-100.0)		242; BE	na
Ab (LFIA, POC)	PRIMA Lab, PRIMA COVID-19 IgG/IgM Rapid Test	IgG	SPEC	na	93/103	90.3 (83.0-94.6)		242; BE	na
Ab (LFIA, POC)	PRIMA Lab, PRIMA COVID-19 IgG/IgM Rapid Test	IgG or IgM	SENS	hospitalised	23/23	100.0 (85.7-100.0)		242; BE	na
Ab (LFIA, POC)	PRIMA Lab, PRIMA COVID-19 IgG/IgM Rapid Test	IgG or IgM	SPEC	na	88/103	85.4 (77.4-91.0)		242; BE	na
Ab (LFIA, POC)	PRIMA Lab, PRIMA COVID-19 IgG/IgM Rapid Test	IgM	SENS	hospitalised	16/23	69.6 (49.1-84.4)		242; BE	na
Ab (LFIA, POC)	PRIMA Lab, PRIMA COVID-19 IgG/IgM Rapid Test	IgM	SPEC	na	96/103	93.2 (86.6-96.7)		242; BE	na
Ab (LFIA, POC)	Phamatech, COVID19 RAPID TEST	IgG	SENS	unk	23/27	85.2 (67.5-94.1)		5; US	na
Ab (LFIA, POC)	Phamatech, COVID19 RAPID TEST	IgG	SPEC	na	67/70	95.7 (88.1-98.5)		5; US	na
Ab (LFIA, POC)	Phamatech, COVID19	IgG or	SENS	unk	23/27	85.2 (67.5-		5; US	na

	RAPID TEST	IgM				94.1)			
Ab (LFIA, POC)	Phamatech, COVID19 RAPID TEST	IgG or IgM	SPEC	na	66/70	94.3 (86.2-97.8)		5; US	na
Ab (LFIA, POC)	Phamatech, COVID19 RAPID TEST	IgM	SENS	unk	6/27	22.2 (10.6-40.8)		5; US	na
Ab (LFIA, POC)	Phamatech, COVID19 RAPID TEST	IgM	SPEC	na	69/70	98.6 (92.3-99.7)		5; US	na
Ab (LFIA, POC)	Premier, (unknown test name)	IgG	SENS	unk	23/30	76.7 (59.1-88.2)		297; US	na
Ab (LFIA, POC)	Premier, (unknown test name)	IgG	SPEC	na	107/108	99.1 (94.9-99.8)		297; US	na
Ab (LFIA, POC)	Premier, (unknown test name)	IgG or IgM	SENS	unk	27/30	90.0 (74.4-96.5)		297; US	na
Ab (LFIA, POC)	Premier, (unknown test name)	IgG or IgM	SPEC	na	105/108	97.2 (92.1-99.1)		297; US	na
Ab (LFIA, POC)	Premier, (unknown test name)	IgM	SENS	unk	26/30	86.7 (70.3-94.7)		297; US	na
Ab (LFIA, POC)	Premier, (unknown test name)	IgM	SPEC	na	106/108	98.1 (93.5-99.5)		297; US	na
Ab (LFIA, POC)	Prometheus Bio, 2019-nCoV IgG/IgM Test Cassette	IgG	SENS	unk	2/2	100.0 (34.2-100.0)		78; ES	na
Ab (LFIA, POC)	Prometheus Bio, 2019-nCoV IgG/IgM Test Cassette	IgG or IgM	SENS	unk	2/2	100.0 (34.2-100.0)		78; ES	na
Ab (LFIA, POC)	Prometheus Bio, 2019-nCoV IgG/IgM Test Cassette	IgM	SENS	unk	2/2	100.0 (34.2-100.0)		78; ES	na
Ab (LFIA, POC)	Qingdao Hightop Biotech, Hightop COVID-19 IgM/IgG Ab Rapid Test Kit	IgG	SENS	unk	60/65	92.3 (83.2-96.7)		8; AU	na
Ab (LFIA, POC)	Qingdao Hightop Biotech, Hightop COVID-19 IgM/IgG Ab Rapid Test Kit	IgG	SPEC	na	92/92	100.0 (96.0-100.0)		8; AU	na
Ab (LFIA, POC)	Qingdao Hightop Biotech, Hightop COVID-19 IgM/IgG Ab Rapid Test Kit	IgG or IgM	SENS	unk	60/65	92.3 (83.2-96.7)		8; AU	na
Ab (LFIA, POC)	Qingdao Hightop Biotech, Hightop COVID-19 IgM/IgG Ab Rapid Test Kit	IgG or IgM	SPEC	na	92/92	100.0 (96.0-100.0)		8; AU	na
Ab (LFIA, POC)	Qingdao Hightop Biotech, Hightop COVID-19 IgM/IgG Ab Rapid Test Kit	IgM	SENS	unk	38/64	59.4 (47.1-70.5)		8; AU	na
Ab (LFIA, POC)	Qingdao Hightop Biotech, Hightop COVID-19 IgM/IgG Ab Rapid Test Kit	IgM	SPEC	na	92/92	100.0 (96.0-100.0)		8; AU	na
Ab (MICROARRAY)	Quotient, MosaiQ COVID-19 Antibody Microarray	IgG or IgM	SENS	hospitalised	0/21	0.0 (0.0-15.5)		166; FR	na
Ab (MICROARRAY)	Quotient, MosaiQ COVID-19 Antibody Microarray	IgG or IgM	SENS	unk	9/9	100.0 (70.1-100.0)		166; FR	na
Ab (MICROARRAY)	Quotient, MosaiQ COVID-19 Antibody Microarray	IgG or IgM	SPEC	na	500/500	100.0 (99.2-100.0)		166; FR	na
Ab (LFIA, POC)	RayBiotech, Novel Coronavirus (SARS-CoV-2) IgM/IgG Antibody Detection Kit	IgG	SENS	mild/asymptomatic	11/23	47.8 (29.2-67.0)		278; NO	na
Ab (LFIA, POC)	RayBiotech, Novel Coronavirus (SARS-CoV-2) IgM/IgG Antibody Detection Kit	IgM	SENS	mild/asymptomatic	10/23	43.5 (25.6-63.2)		278; NO	na
Ab (CLIA)	Roche, Elecsys Anti-SARS-CoV-2	total Ab	SENS	mild/asymptomatic	88/96	91.7 (84.4-95.7)	74.5	3, 180; NL, UK	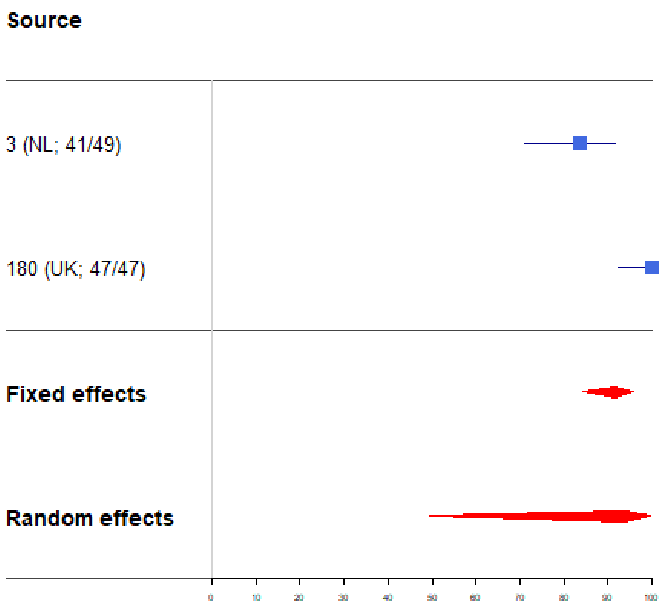
Ab (CLIA)	Roche, Elecsys Anti-SARS-CoV-2	total Ab	SENS	hospitalised	60/70	85.7 (75.7-92.1)	23.3	3, 44, 226; CA, DE, NL	

									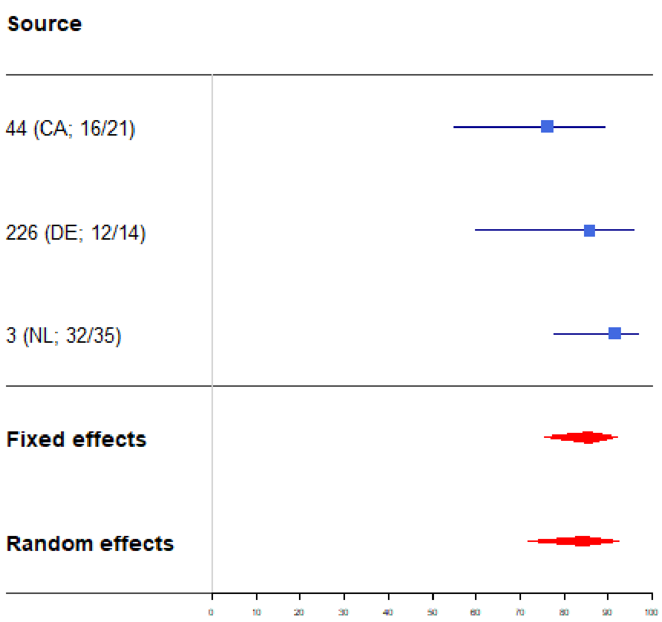
Ab (CLIA)	Roche, Elecsys Anti-SARS-CoV-2	total Ab	SENS	unk	1279/1351	94.7 (93.3-95.7)	50.3	6, 10, 65, 69, 81, 83, 84, 151, 224, 255, 271, 285, 296, 299; AT(2), BE(3), DE(2), DK, SE, SG, UK(2), US(2)	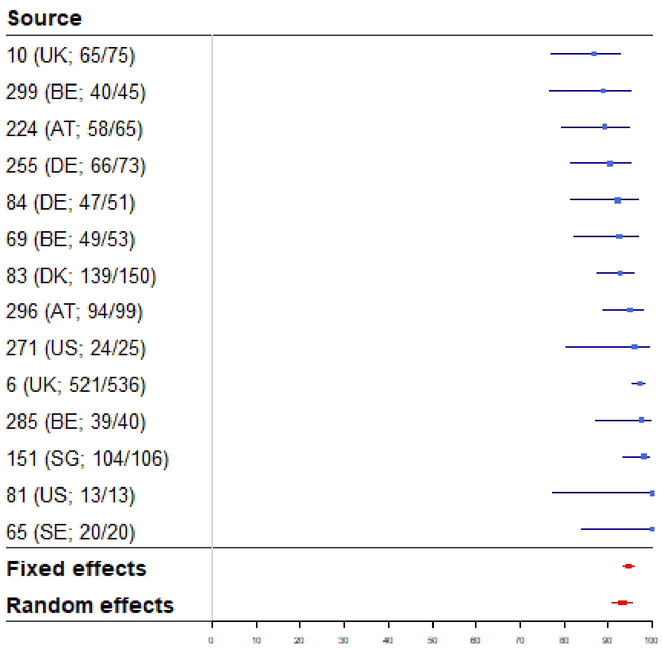
Ab (CLIA)	Roche, Elecsys Anti-SARS-CoV-2	total Ab	SPEC	na	7819/7833	99.8 (99.7-99.9)	16.5	3, 6, 10, 43, 44, 65, 69, 81, 83, 84, 93, 145, 151, 171, 224, 226, 245, 269, 271, 275, 285, 299; AT, BE(3), CA, DE(5), DK, LU, NL, SE, SG, UK(2), US(5)	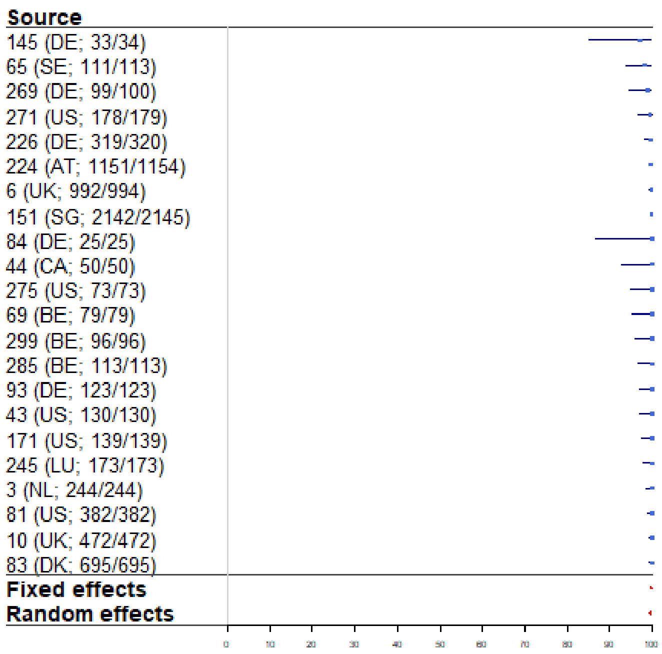
Ab (LFIA, POC)	SD BioSensor, Standard Q COVID-19 IgM/IgG Duo	IgG	SENS	hospitalised	28/28	100.0 (87.9-100.0)		217; US	na
Ab (LFIA, POC)	SD BioSensor, Standard Q COVID-19 IgM/IgG Duo	IgG	SENS	unk	19/27	70.4 (51.5-84.1)		5; US	na
Ab (LFIA, POC)	SD BioSensor, Standard Q COVID-19 IgM/IgG Duo	IgG	SPEC	na	1251/1254	99.8 (99.3-99.9)	67.1	5, 217; US(2)	

									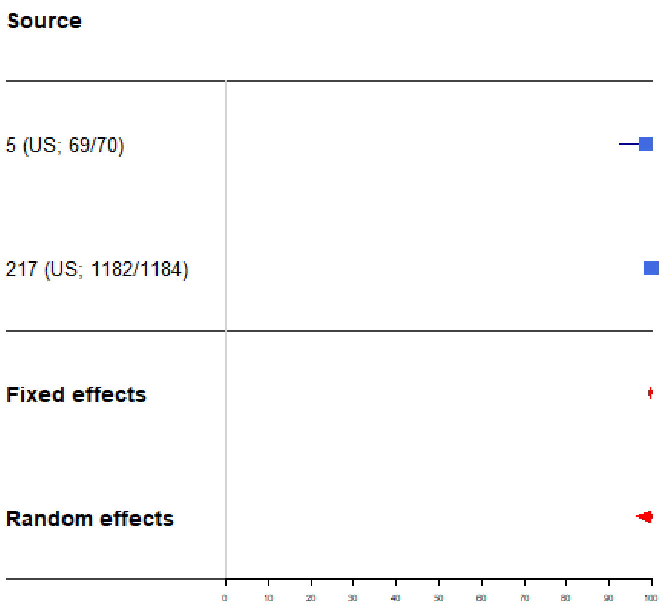
Ab (LFIA, POC)	SD BioSensor, Standard Q COVID-19 IgM/IgG Duo	IgG or IgM	SENS	unk	20/27	74.1 (55.3-86.8)		5; US	na
Ab (LFIA, POC)	SD BioSensor, Standard Q COVID-19 IgM/IgG Duo	IgG or IgM	SPEC	na	69/70	98.6 (92.3-99.7)		5; US	na
Ab (LFIA, POC)	SD BioSensor, Standard Q COVID-19 IgM/IgG Duo	IgM	SENS	hospitalised	24/28	85.7 (68.5-94.3)		217; US	na
Ab (LFIA, POC)	SD BioSensor, Standard Q COVID-19 IgM/IgG Duo	IgM	SENS	unk	13/27	48.1 (30.7-66.0)		5; US	na
Ab (LFIA, POC)	SD BioSensor, Standard Q COVID-19 IgM/IgG Duo	IgM	SPEC	na	1241/1256	98.8 (98.0-99.3)	0.0	5, 217; US(2)	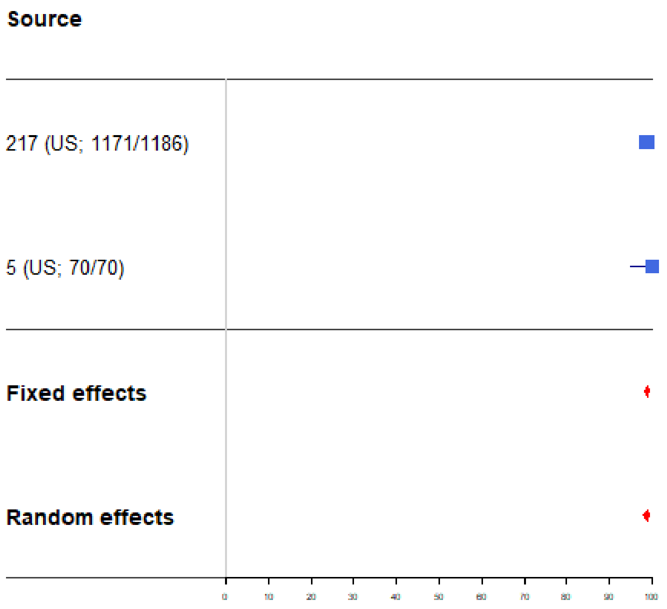
Ab (LFIA, POC)	Salofa, Sienna COVID-19 IgG/IgM Rapid Test Cassette (whole blood/serum/plasma)	IgG	SENS	unk	34/38	89.5 (75.9-95.8)	35.3	5, 78; ES, US	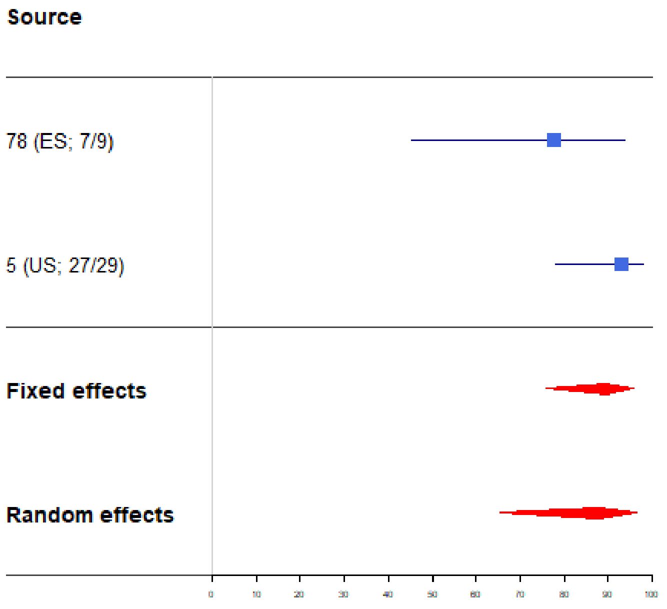
Ab (LFIA, POC)	Salofa, Sienna COVID-19 IgG/IgM Rapid Test Cassette (whole blood/serum/plasma)	IgG	SPEC	na	69/70	98.6 (92.3-99.7)		5; US	na
Ab (LFIA, POC)	Salofa, Sienna COVID-19 IgG/IgM Rapid Test Cassette (whole blood/serum/plasma)	IgG or IgM	SENS	unk	70/86	81.4 (71.9-88.2)	71.2	5, 78; ES, US	

									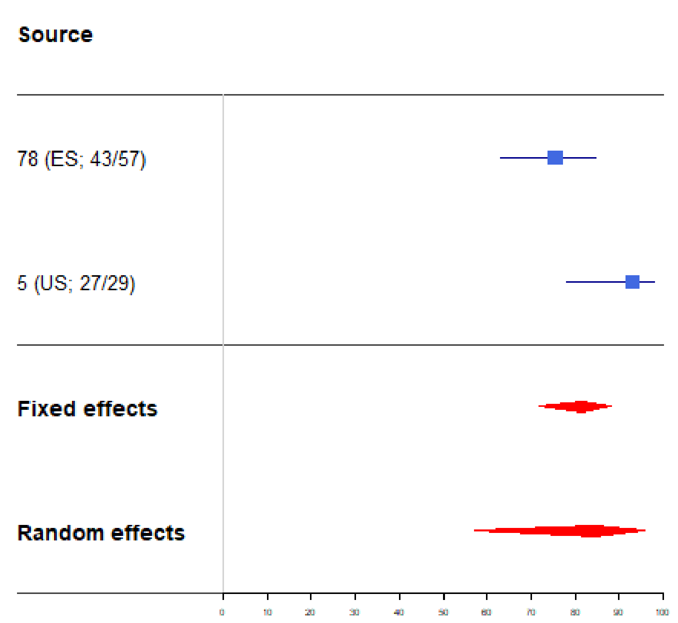
Ab (LFIA, POC)	Salofa, Sienna COVID-19 IgG/IgM Rapid Test Cassette (whole blood/serum/plasma)	IgG or IgM	SPEC	na	69/70	98.6 (92.3-99.7)		5; US	na
Ab (LFIA, POC)	Salofa, Sienna COVID-19 IgG/IgM Rapid Test Cassette (whole blood/serum/plasma)	IgM	SENS	unk	34/38	89.5 (75.9-95.8)	0.0	5, 78; ES, US	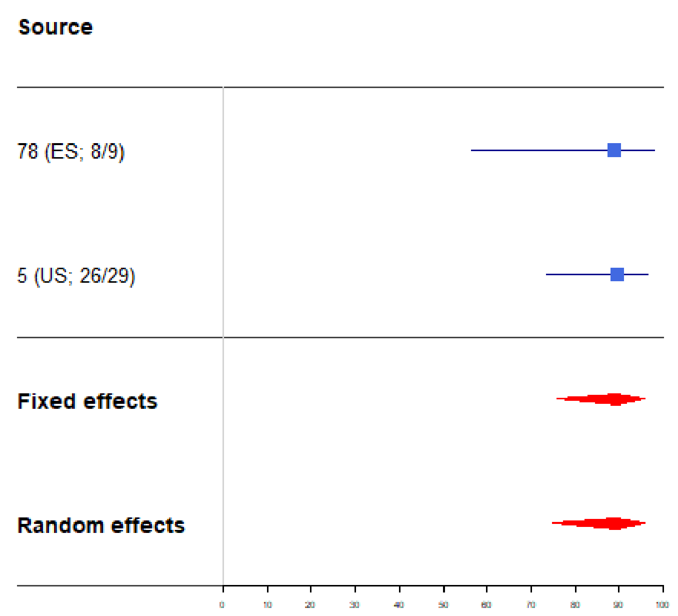
Ab (LFIA, POC)	Salofa, Sienna COVID-19 IgG/IgM Rapid Test Cassette (whole blood/serum/plasma)	IgM	SPEC	na	70/70	100.0 (94.8-100.0)		5; US	na
Ab (LFIA, POC)	Shanghai Fosun Long March Medical Science, Fosun COVID-19 IgG/IgM Rapid Antibody Detection Kit	IgG	SENS	unk	9/27	33.3 (18.6-52.2)		5; US	na
Ab (LFIA, POC)	Shanghai Fosun Long March Medical Science, Fosun COVID-19 IgG/IgM Rapid Antibody Detection Kit	IgG	SPEC	na	69/70	98.6 (92.3-99.7)		5; US	na
Ab (LFIA, POC)	Shanghai Fosun Long March Medical Science, Fosun COVID-19 IgG/IgM Rapid Antibody Detection Kit	IgG or IgM	SENS	unk	9/27	33.3 (18.6-52.2)		5; US	na
Ab (LFIA, POC)	Shanghai Fosun Long March Medical Science, Fosun COVID-19 IgG/IgM Rapid Antibody Detection Kit	IgG or IgM	SPEC	na	66/70	94.3 (86.2-97.8)		5; US	na
Ab (LFIA, POC)	Shanghai Fosun Long March Medical Science, Fosun COVID-19 IgG/IgM Rapid Antibody Detection Kit	IgM	SENS	unk	2/27	7.4 (2.1-23.4)		5; US	na
Ab (LFIA, POC)	Shanghai Fosun Long March Medical Science, Fosun COVID-19 IgG/IgM Rapid Antibody Detection Kit	IgM	SPEC	na	67/70	95.7 (88.1-98.5)		5; US	na
Ab (LFIA, POC)	Shanghai LiangRun Biomedicine Tech., Diagnostic kit IgM/IgG of Novel Coronavirus COVID-19	IgG	SENS	mild/asymptomatic	0/10	0.0 (0.0-27.8)		4; NL	na
Ab (LFIA, POC)	Shanghai LiangRun Biomedicine Tech., Diagnostic kit IgM/IgG of Novel Coronavirus COVID-19	IgG	SENS	hospitalised	NaN/NaN	NaN (NaN-NaN)		4;	na
Ab (LFIA, POC)	Shanghai LiangRun Biomedicine Tech., Diagnostic kit IgM/IgG of	IgG	SPEC	na	25/25	100.0 (86.7-100.0)		4; NL	na

	Novel Coronavirus COVID-19								
Ab (LFIA, POC)	Shanghai LiangRun Biomedicine Tech., Diagnostic kit IgM/IgG of Novel Coronavirus COVID-19	IgM	SENS	mild/asymptomatic	1/10	10.0 (1.8-40.4)		4; NL	na
Ab (LFIA, POC)	Shanghai LiangRun Biomedicine Tech., Diagnostic kit IgM/IgG of Novel Coronavirus COVID-19	IgM	SENS	hospitalised	NaN/NaN	NaN (NaN-NaN)		4;	na
Ab (LFIA, POC)	Shanghai Outdo Biotech, Novel Coronavirus (SARS-CoV-2) Antibody (IgM / IgG) Test (Colloidal Gold)	IgG or IgM	SENS	unk	23/24	95.8 (79.8-99.3)		258; CN	na
Ab (LFIA, POC)	Shanghai Outdo Biotech, Novel Coronavirus (SARS-CoV-2) Antibody (IgM / IgG) Test (Colloidal Gold)	IgG or IgM	SPEC	na	19/19	100.0 (83.2-100.0)		258; CN	na
Ab (CLIA)	Shenzhen New Industries Biomedical Engineering (SNIBE), Maglumi 2019-nCoV (SARS-CoV-2) IgG/IgM kit	IgG	SENS	hospitalised	71/76	93.4 (85.5-97.2)	60.3	177, 263; BE(2)	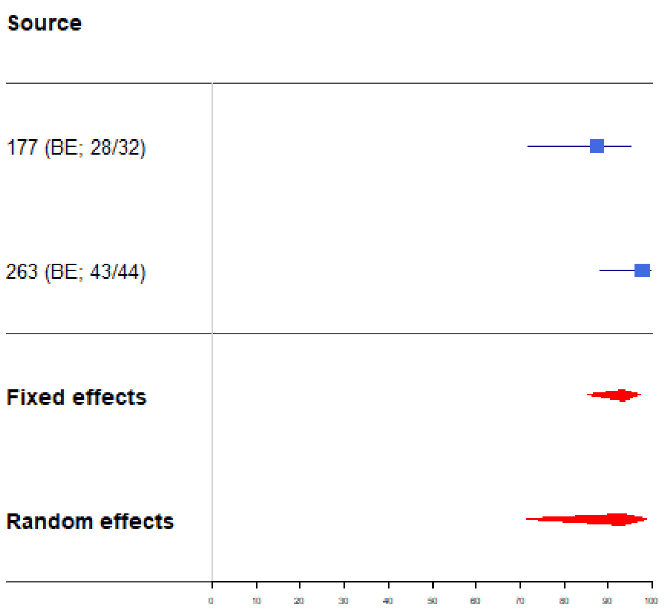
Ab (CLIA)	Shenzhen New Industries Biomedical Engineering (SNIBE), Maglumi 2019-nCoV (SARS-CoV-2) IgG/IgM kit	IgG	SENS	unk	987/1084	91.1 (89.2-92.6)	97.2	83, 213; CN, DK	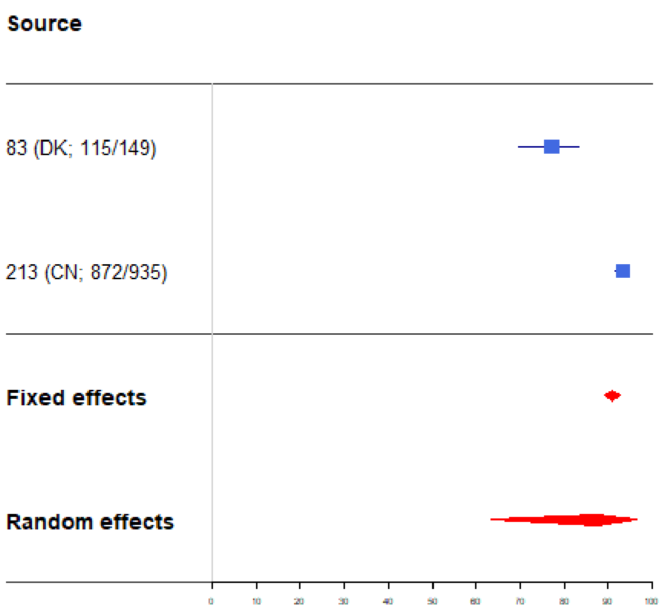
Ab (CLIA)	Shenzhen New Industries Biomedical Engineering (SNIBE), Maglumi 2019-nCoV (SARS-CoV-2) IgG/IgM kit	IgG	SPEC	na	1703/1744	97.6 (96.8-98.3)	93.0	83, 177, 213, 263, 291; BE(2), CN(2), DK	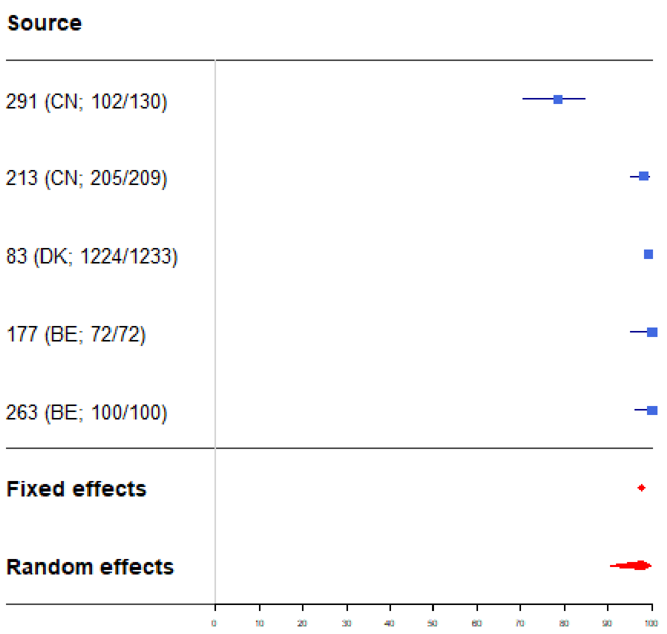
Ab (CLIA)	Shenzhen New Industries Biomedical Engineering (SNIBE), Maglumi 2019-	IgG or IgM	SENS	hospitalised	73/76	96.1 (89.0-98.6)	0.0	177, 263; BE(2)	

	nCoV (SARS-CoV-2) IgG/IgM kit								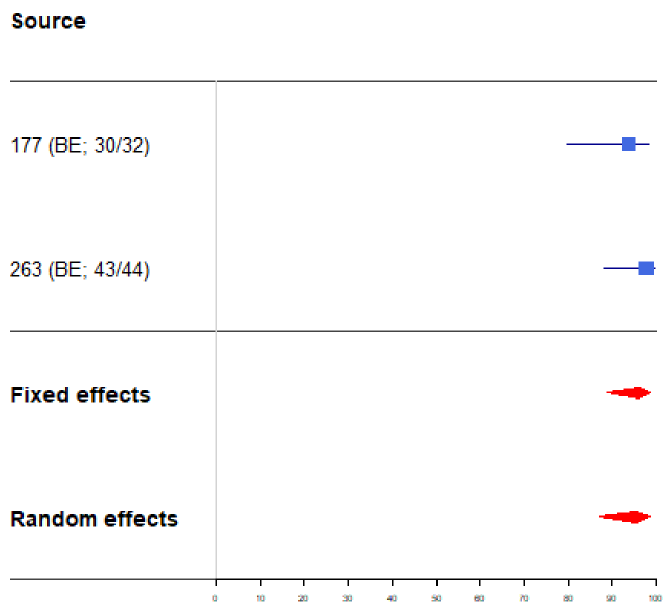
Ab (CLIA)	Shenzhen New Industries Biomedical Engineering (SNIBE), Maglumi 2019-nCoV (SARS-CoV-2) IgG/IgM kit	IgG or IgM	SENS	unk	40/40	100.0 (91.2-100.0)		285; BE	na
Ab (CLIA)	Shenzhen New Industries Biomedical Engineering (SNIBE), Maglumi 2019-nCoV (SARS-CoV-2) IgG/IgM kit	IgG or IgM	SPEC	na	281/285	98.6 (96.4-99.5)	34.0	177, 263, 285; BE(3)	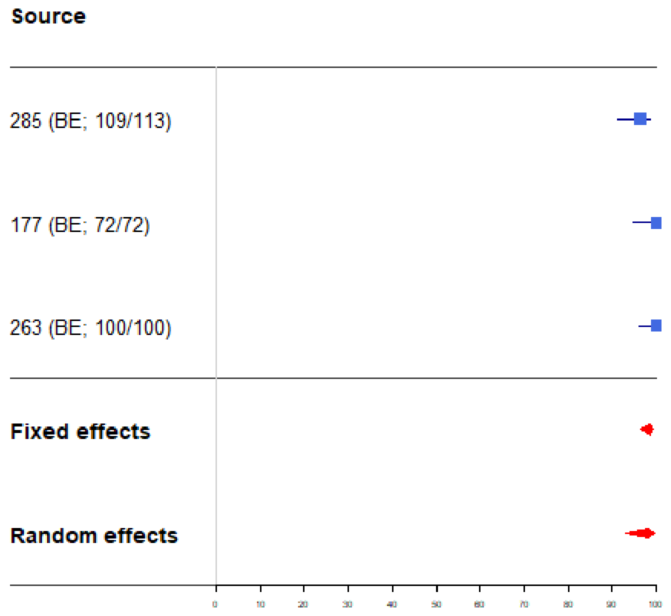
Ab (CLIA)	Shenzhen New Industries Biomedical Engineering (SNIBE), Maglumi 2019-nCoV (SARS-CoV-2) IgG/IgM kit	IgM	SENS	hospitalised	71/76	93.4 (85.5-97.2)	60.3	177, 263; BE(2)	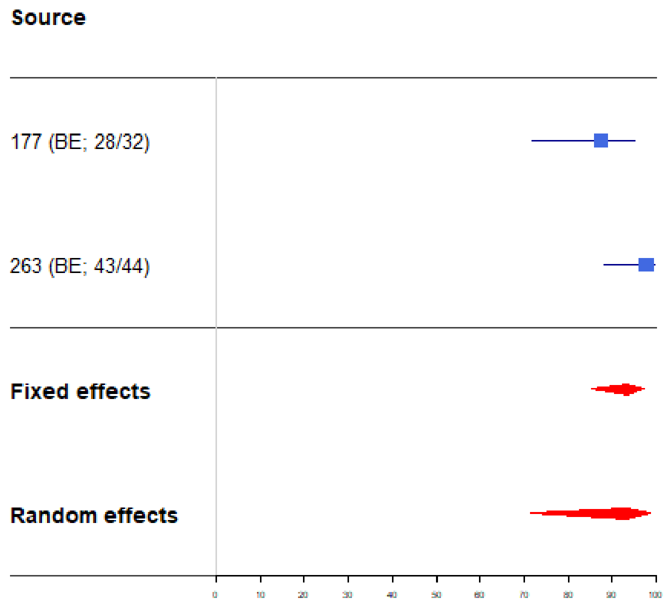
Ab (CLIA)	Shenzhen New Industries Biomedical Engineering (SNIBE), Maglumi 2019-nCoV (SARS-CoV-2) IgG/IgM kit	IgM	SENS	unk	735/1084	67.8 (65.0-70.5)	98.8	83, 213; CN, DK	

									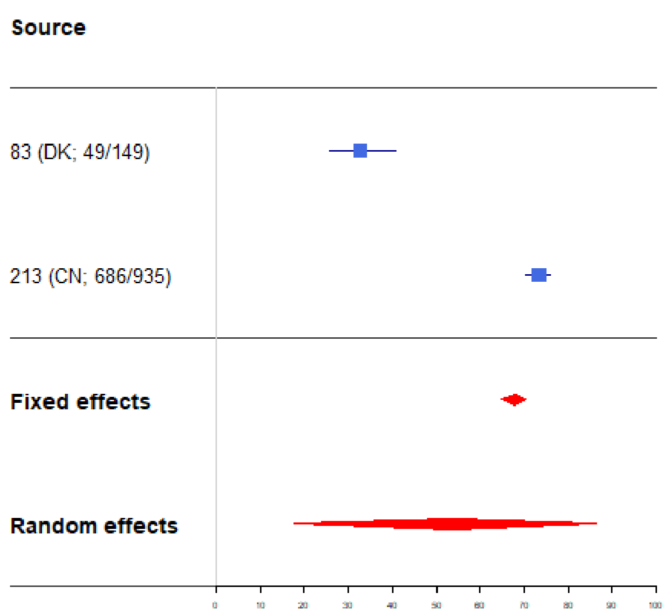
Ab (CLIA)	Shenzhen New Industries Biomedical Engineering (SNIBE), Maglumi 2019-nCoV (SARS-CoV-2) IgG/IgM kit	IgM	SPEC	na	1742/1756	99.2 (98.7-99.5)	84.3	83, 177, 213, 263, 291; BE(2), CN(2), DK	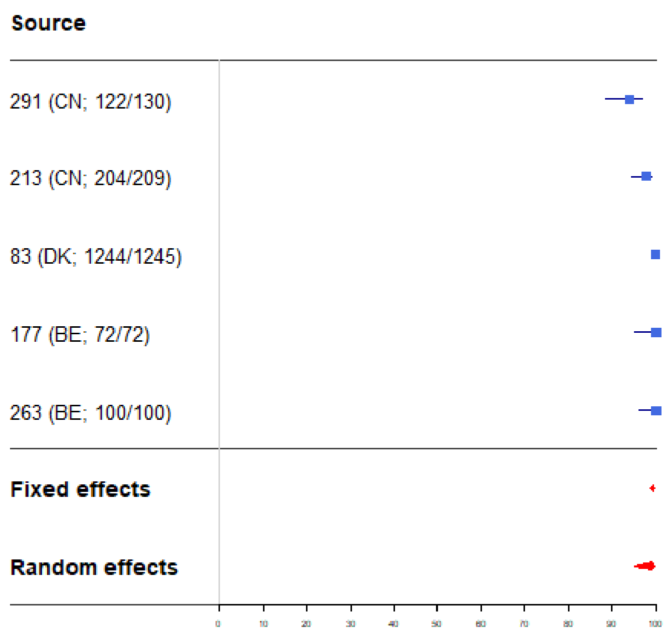
Ab (CLIA)	Shenzhen Yahuilong (YHLO) Biotech, SARS-CoV-2 IgG/IgM antibody detection kit	IgG	SENS	mild/asymptomatic	92/95	96.8 (91.1-98.9)		324; CN	na
Ab (CLIA)	Shenzhen Yahuilong (YHLO) Biotech, SARS-CoV-2 IgG/IgM antibody detection kit	IgG	SENS	hospitalised	NaN/NaN	NaN (NaN-NaN)		138;	na
Ab (CLIA)	Shenzhen Yahuilong (YHLO) Biotech, SARS-CoV-2 IgG/IgM antibody detection kit	IgG	SENS	unk	141/149	94.6 (89.8-97.3)		83; DK	na
Ab (CLIA)	Shenzhen Yahuilong (YHLO) Biotech, SARS-CoV-2 IgG/IgM antibody detection kit	IgG	SPEC	na	1300/1313	99.0 (98.3-99.4)	6.2	83, 138, 291, 322; CN(2), DK, IT	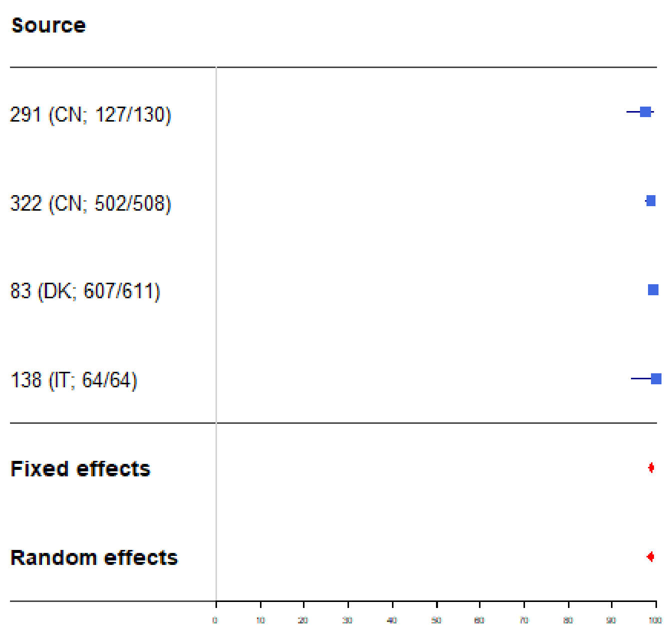
Ab (CLIA)	Shenzhen Yahuilong (YHLO) Biotech, SARS-CoV-2 IgG/IgM antibody detection kit	IgM	SENS	mild/asymptomatic	48/95	50.5 (40.6-60.4)		324; CN	na
Ab (CLIA)	Shenzhen Yahuilong (YHLO) Biotech, SARS-CoV-2 IgG/IgM antibody detection kit	IgM	SENS	hospitalised	NaN/NaN	NaN (NaN-NaN)		138;	na
Ab (CLIA)	Shenzhen Yahuilong (YHLO) Biotech, SARS-CoV-2 IgG/IgM antibody detection kit	IgM	SENS	unk	63/150	42.0 (34.4-50.0)		83; DK	na
Ab (CLIA)	Shenzhen Yahuilong (YHLO) Biotech, SARS-	IgM	SPEC	na	1297/1314	98.7 (97.9-99.2)	81.4	83, 138, 291, 322; CN(2), DK, IT	

	CoV-2 IgG/IgM antibody detection kit								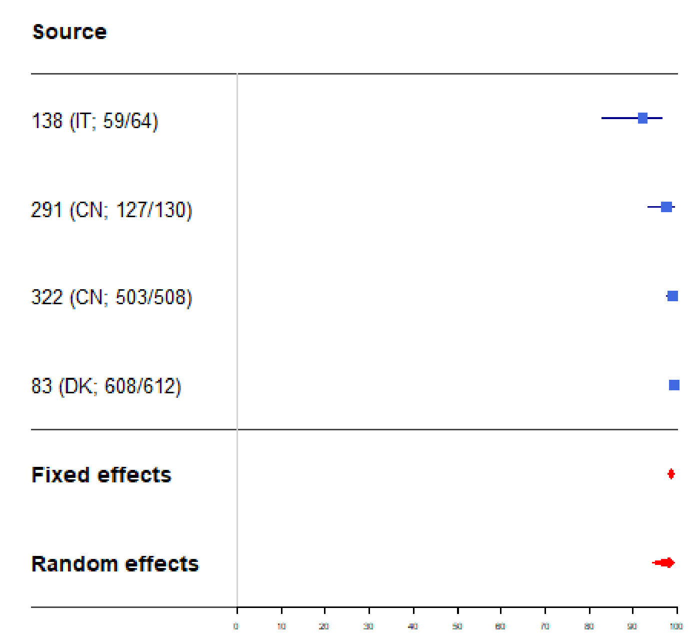
Ab (CLIA)	Siemens, Healthineers SARS-CoV-2 Total Assay on Atellica/ADVIA Centaur	total Ab	SENS	mild/asymptomatic	45/47	95.7 (85.8-98.8)		180; UK	na
Ab (CLIA)	Siemens, Healthineers SARS-CoV-2 Total Assay on Atellica/ADVIA Centaur	total Ab	SENS	hospitalised	12/14	85.7 (60.1-96.0)		226; DE	na
Ab (CLIA)	Siemens, Healthineers SARS-CoV-2 Total Assay on Atellica/ADVIA Centaur	total Ab	SENS	unk	732/757	96.7 (95.2-97.8)	79.2	6, 83, 255; DE, DK, UK	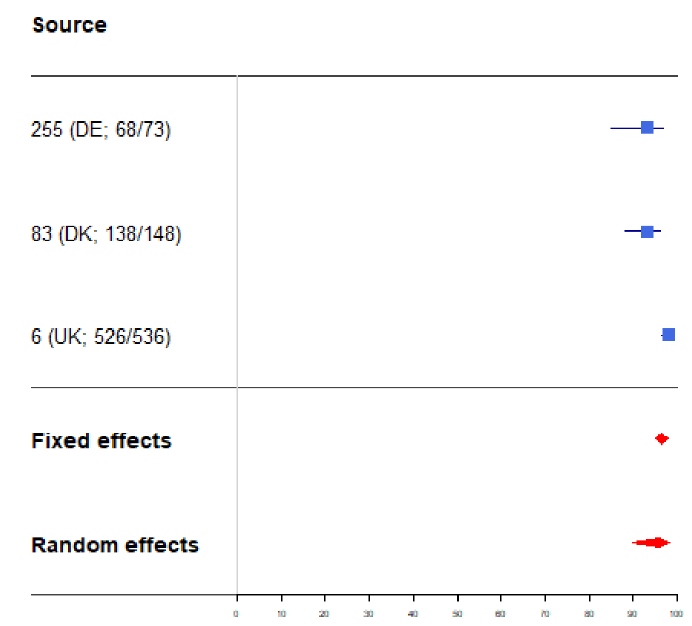
Ab (CLIA)	Siemens, Healthineers SARS-CoV-2 Total Assay on Atellica/ADVIA Centaur	total Ab	SPEC	na	2104/2108	99.8 (99.5-99.9)	0.0	6, 83, 93, 226; DE(2), DK, UK	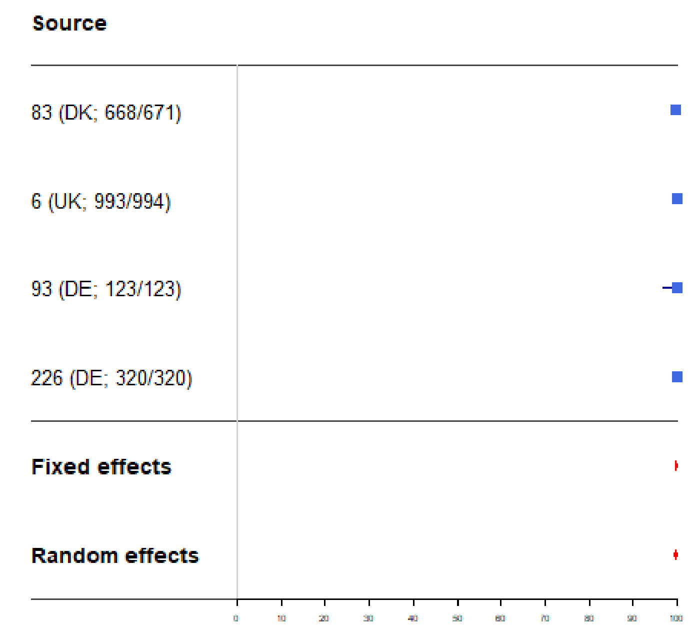
Ab (CLIA)	Siemens, Healthineers SARS-CoV-2 Total Assay on Vista	total Ab	SENS	unk	119/147	81.0 (73.8-86.5)		83; DK	na
Ab (CLIA)	Siemens, Healthineers SARS-CoV-2 Total Assay on Vista	total Ab	SPEC	na	631/631	100.0 (99.4-100.0)		83; DK	na
Ab (LFIA, POC)	Sugentech, SGTi-flex COVID-19 IgM/IgG	IgG	SENS	unk	27/29	93.1 (78.0-98.1)		5; US	na
Ab (LFIA, POC)	Sugentech, SGTi-flex COVID-19 IgM/IgG	IgG	SPEC	na	70/70	100.0 (94.8-100.0)		5; US	na
Ab (LFIA, POC)	Sugentech, SGTi-flex COVID-19 IgM/IgG	IgG or IgM	SENS	hospitalised	NaN/NaN	NaN (NaN-NaN)		40;	na
Ab (LFIA, POC)	Sugentech, SGTi-flex COVID-19 IgM/IgG	IgG or IgM	SENS	unk	29/29	100.0 (88.3-100.0)		5; US	na
Ab (LFIA, POC)	Sugentech, SGTi-flex	IgG or	SPEC	na	64/70	91.4 (82.5-		5; US	na

	COVID-19 IgM/IgG	IgM				96.0)			
Ab (LFIA, POC)	Sugentech, SGTi-flex COVID-19 IgM/IgG	IgM	SENS	unk	27/29	93.1 (78.0-98.1)		5; US	na
Ab (LFIA, POC)	Sugentech, SGTi-flex COVID-19 IgM/IgG	IgM	SPEC	na	64/70	91.4 (82.5-96.0)		5; US	na
Ab (LFIA, POC)	Sure Bio-Tech, SARS-CoV-2 IgM/IgG Ab Rapid Test	IgG	SENS	unk	24/30	80.0 (62.7-90.5)		297; US	na
Ab (LFIA, POC)	Sure Bio-Tech, SARS-CoV-2 IgM/IgG Ab Rapid Test	IgG	SPEC	na	108/108	100.0 (96.6-100.0)		297; US	na
Ab (LFIA, POC)	Sure Bio-Tech, SARS-CoV-2 IgM/IgG Ab Rapid Test	IgG or IgM	SENS	unk	25/30	83.3 (66.4-92.7)		297; US	na
Ab (LFIA, POC)	Sure Bio-Tech, SARS-CoV-2 IgM/IgG Ab Rapid Test	IgG or IgM	SPEC	na	108/108	100.0 (96.6-100.0)		297; US	na
Ab (LFIA, POC)	Sure Bio-Tech, SARS-CoV-2 IgM/IgG Ab Rapid Test	IgM	SENS	unk	22/30	73.3 (55.6-85.8)		297; US	na
Ab (LFIA, POC)	Sure Bio-Tech, SARS-CoV-2 IgM/IgG Ab Rapid Test	IgM	SPEC	na	108/108	100.0 (96.6-100.0)		297; US	na
Ab (LFIA, POC)	SureScreen Diagnostic, Covid-19 IgG/IgM Rapid Test Cassette	IgG	SENS	mild/asymptomatic	12/23	52.2 (33.0-70.8)		278; NO	na
Ab (LFIA, POC)	SureScreen Diagnostic, Covid-19 IgG/IgM Rapid Test Cassette	IgG	SENS	hospitalised	48/49	98.0 (89.3-99.6)		242; BE	na
Ab (LFIA, POC)	SureScreen Diagnostic, Covid-19 IgG/IgM Rapid Test Cassette	IgG	SPEC	na	196/198	99.0 (96.4-99.7)	0.0	4, 242; BE, NL	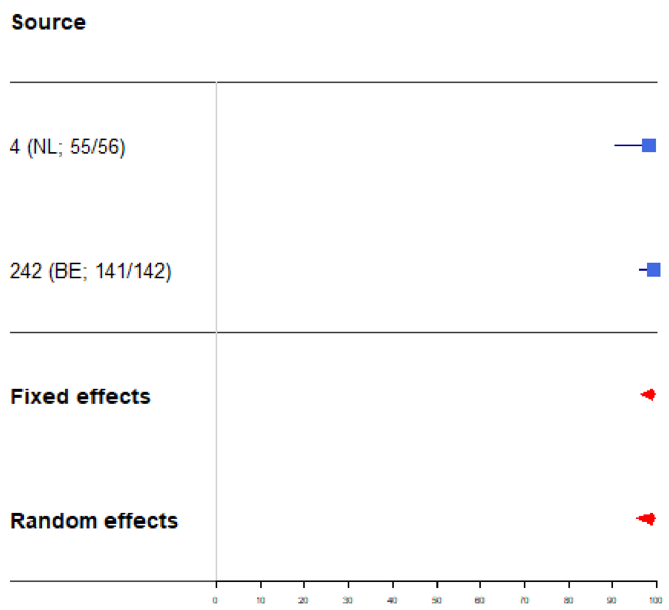
Ab (LFIA, POC)	SureScreen Diagnostic, Covid-19 IgG/IgM Rapid Test Cassette	IgG or IgM	SENS	mild/asymptomatic	15/37	40.5 (26.3-56.5)		4; NL	na
Ab (LFIA, POC)	SureScreen Diagnostic, Covid-19 IgG/IgM Rapid Test Cassette	IgG or IgM	SENS	hospitalised	48/49	98.0 (89.3-99.6)		242; BE	na
Ab (LFIA, POC)	SureScreen Diagnostic, Covid-19 IgG/IgM Rapid Test Cassette	IgG or IgM	SPEC	na	137/142	96.5 (92.0-98.5)		242; BE	na
Ab (LFIA, POC)	SureScreen Diagnostic, Covid-19 IgG/IgM Rapid Test Cassette	IgM	SENS	mild/asymptomatic	19/23	82.6 (62.9-93.0)		278; NO	na
Ab (LFIA, POC)	SureScreen Diagnostic, Covid-19 IgG/IgM Rapid Test Cassette	IgM	SENS	hospitalised	48/49	98.0 (89.3-99.6)		242; BE	na
Ab (LFIA, POC)	SureScreen Diagnostic, Covid-19 IgG/IgM Rapid Test Cassette	IgM	SPEC	na	138/142	97.2 (93.0-98.9)		242; BE	na
Ab (LFIA, POC)	Testsealabs, SARS-COV-2-IgG/IgM Test Cassette	IgG	SENS	unk	11/27	40.7 (24.5-59.3)		5; US	na
Ab (LFIA, POC)	Testsealabs, SARS-COV-2-IgG/IgM Test Cassette	IgG	SPEC	na	66/70	94.3 (86.2-97.8)		5; US	na
Ab (LFIA, POC)	Testsealabs, SARS-COV-2-IgG/IgM Test Cassette	IgG or IgM	SENS	unk	22/27	81.5 (63.3-91.8)		5; US	na
Ab (LFIA, POC)	Testsealabs, SARS-COV-2-IgG/IgM Test Cassette	IgG or IgM	SPEC	na	66/70	94.3 (86.2-97.8)		5; US	na
Ab (LFIA, POC)	Testsealabs, SARS-COV-2-IgG/IgM Test Cassette	IgM	SENS	unk	19/27	70.4 (51.5-84.1)		5; US	na
Ab (LFIA, POC)	Testsealabs, SARS-COV-2-IgG/IgM Test Cassette	IgM	SPEC	na	70/70	100.0 (94.8-100.0)		5; US	na
Ab (LFIA, POC)	Tianjin Beroni Biotechnology, SARS-COV-2 IgG/IgM Antibody Detection Kit	IgG	SENS	unk	9/27	33.3 (18.6-52.2)		5; US	na
Ab (LFIA, POC)	Tianjin Beroni Biotechnology, SARS-COV-2 IgG/IgM Antibody Detection Kit	IgG	SPEC	na	70/70	100.0 (94.8-100.0)		5; US	na
Ab (LFIA, POC)	Tianjin Beroni Biotechnology, SARS-COV-2 IgG/IgM Antibody Detection Kit	IgG or IgM	SENS	unk	24/27	88.9 (71.9-96.1)		5; US	na
Ab (LFIA, POC)	Tianjin Beroni	IgG or	SPEC	na	70/70	100.0		5; US	na

	Biotechnology, SARS-COV-2 IgG/IgM Antibody Detection Kit	IgM				(94.8-100.0)			
Ab (LFIA, POC)	Tianjin Beroni Biotechnology, SARS-COV-2 IgG/IgM Antibody Detection Kit	IgM	SENS	unk	22/27	81.5 (63.3-91.8)		5; US	na
Ab (LFIA, POC)	Tianjin Beroni Biotechnology, SARS-COV-2 IgG/IgM Antibody Detection Kit	IgM	SPEC	na	70/70	100.0 (94.8-100.0)		5; US	na
Ab (LFIA, POC)	Tianjin New Bay Bioresearch C. #1, Quik Pac II COVID-19 IgG & IgM Test	IgG	SENS	unk	22/29	75.9 (57.9-87.8)		5; US	na
Ab (LFIA, POC)	Tianjin New Bay Bioresearch C. #1, Quik Pac II COVID-19 IgG & IgM Test	IgG	SPEC	na	70/70	100.0 (94.8-100.0)		5; US	na
Ab (LFIA, POC)	Tianjin New Bay Bioresearch C. #1, Quik Pac II COVID-19 IgG & IgM Test	IgG or IgM	SENS	unk	28/29	96.6 (82.8-99.4)		5; US	na
Ab (LFIA, POC)	Tianjin New Bay Bioresearch C. #1, Quik Pac II COVID-19 IgG & IgM Test	IgG or IgM	SPEC	na	69/70	98.6 (92.3-99.7)		5; US	na
Ab (LFIA, POC)	Tianjin New Bay Bioresearch C. #1, Quik Pac II COVID-19 IgG & IgM Test	IgM	SENS	unk	27/29	93.1 (78.0-98.1)		5; US	na
Ab (LFIA, POC)	Tianjin New Bay Bioresearch C. #1, Quik Pac II COVID-19 IgG & IgM Test	IgM	SPEC	na	69/70	98.6 (92.3-99.7)		5; US	na
Ab (LFIA, POC)	UCP Biosciences, Coronavirus IgG/IgM Antibody (COVID-19) Test Cassette	IgG	SENS	unk	23/32	71.9 (54.6-84.4)		297; US	na
Ab (LFIA, POC)	UCP Biosciences, Coronavirus IgG/IgM Antibody (COVID-19) Test Cassette	IgG	SPEC	na	105/107	98.1 (93.4-99.5)		297; US	na
Ab (LFIA, POC)	UCP Biosciences, Coronavirus IgG/IgM Antibody (COVID-19) Test Cassette	IgG or IgM	SENS	unk	25/32	78.1 (61.2-89.0)		297; US	na
Ab (LFIA, POC)	UCP Biosciences, Coronavirus IgG/IgM Antibody (COVID-19) Test Cassette	IgG or IgM	SPEC	na	105/107	98.1 (93.4-99.5)		297; US	na
Ab (LFIA, POC)	UCP Biosciences, Coronavirus IgG/IgM Antibody (COVID-19) Test Cassette	IgM	SENS	unk	25/30	83.3 (66.4-92.7)		297; US	na
Ab (LFIA, POC)	UCP Biosciences, Coronavirus IgG/IgM Antibody (COVID-19) Test Cassette	IgM	SPEC	na	105/107	98.1 (93.4-99.5)		297; US	na
Ab (ELISA)	Vircell, COVID-19 ELISA IgG	IgG	SENS	mild/asymptomatic	NaN/NaN	NaN (NaN-NaN)		3;	na
Ab (ELISA)	Vircell, COVID-19 ELISA IgG	IgG	SENS	hospitalised	15/15	100.0 (79.6-100.0)		3; NL	na
Ab (ELISA)	Vircell, COVID-19 ELISA IgG	IgG	SPEC	na	246/264	93.2 (89.5-95.6)	0.0	3, 146, 245; DE, LU, NL	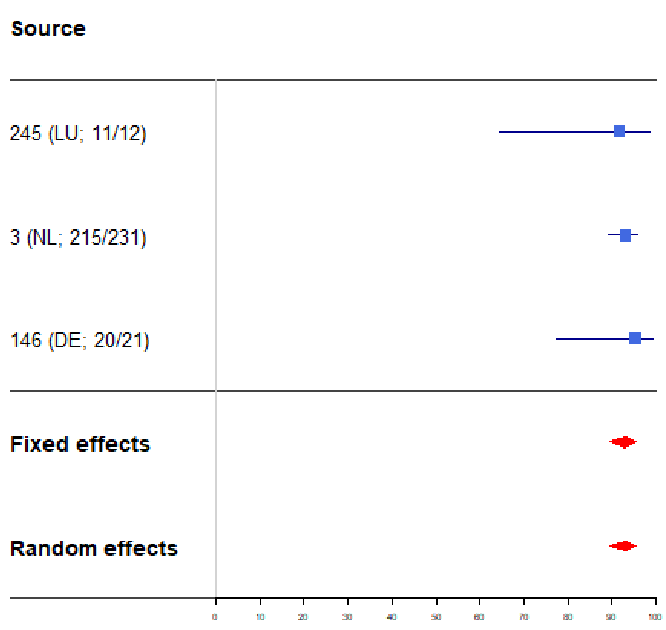
Ab (ELISA)	Vircell, COVID-19 ELISA IgM+IgA	IgM or IgA	SENS	mild/asymptomatic	NaN/NaN	NaN (NaN-NaN)		3;	na
Ab (ELISA)	Vircell, COVID-19 ELISA IgM+IgA	IgM or IgA	SENS	hospitalised	15/15	100.0 (79.6-100.0)		3; NL	na
Ab (ELISA)	Vircell, COVID-19 ELISA IgM+IgA	IgM or IgA	SPEC	na	122/156	78.2 (71.1-84.0)	91.2	3, 245; LU, NL	

									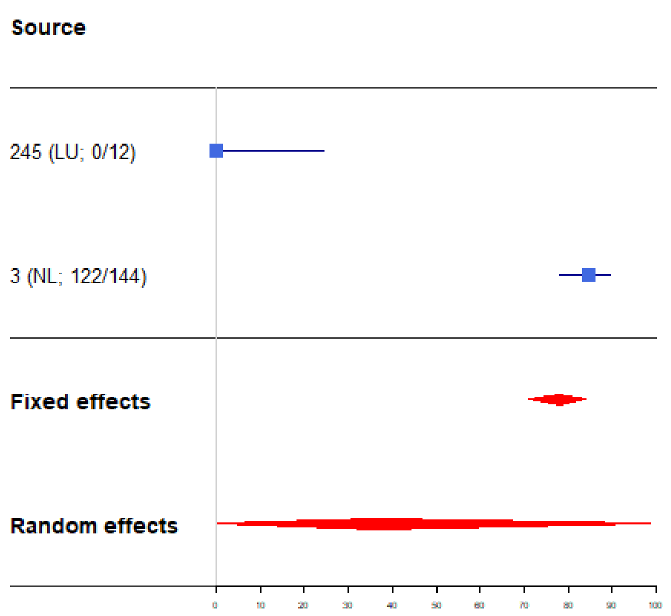
Ab (CLIA)	Vircell, COVID-19 VIRCLIA	IgG	SENS	mild/asymptomatic	NaN/NaN	NaN (NaN-NaN)		3;	na
Ab (CLIA)	Vircell, COVID-19 VIRCLIA	IgG	SENS	hospitalised	NaN/NaN	NaN (NaN-NaN)		3;	na
Ab (CLIA)	Vircell, COVID-19 VIRCLIA	IgG	SPEC	na	124/129	96.1 (91.2-98.3)		3; NL	na
Ab (CLIA)	Vircell, COVID-19 VIRCLIA	IgM or IgA	SENS	mild/asymptomatic	NaN/NaN	NaN (NaN-NaN)		3;	na
Ab (CLIA)	Vircell, COVID-19 VIRCLIA	IgM or IgA	SENS	hospitalised	NaN/NaN	NaN (NaN-NaN)		3;	na
Ab (CLIA)	Vircell, COVID-19 VIRCLIA	IgM or IgA	SENS	unk	NaN/NaN	NaN (NaN-NaN)		145;	na
Ab (CLIA)	Vircell, COVID-19 VIRCLIA	IgM or IgA	SPEC	na	95/100	95.0 (88.8-97.8)	11.5	3, 145; DE, NL	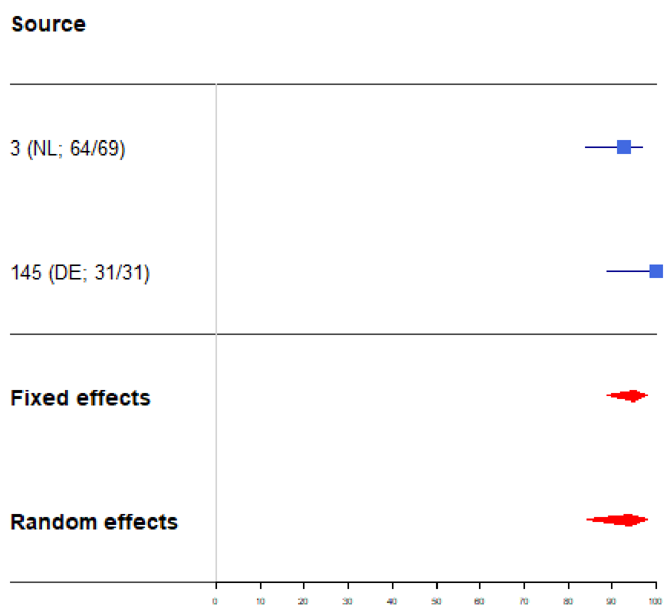
Ab (ELISA)	Virotech, SARS-CoV-2 (COVID-19) IgG ELISA	IgG	SENS	unk	48/73	65.8 (54.3-75.6)		255; DE	na
Ab (ELISA)	Virotech, SARS-CoV-2 (COVID-19) IgG ELISA	IgG	SPEC	na	35/35	100.0 (90.1-100.0)		145; DE	na
Ab (LFIA, POC)	VivaChek Biotech, VivaDiag COVID-19 IgM/IgG Rapid Test	IgG	SENS	hospitalised	23/23	100.0 (85.7-100.0)		242; BE	na
Ab (LFIA, POC)	VivaChek Biotech, VivaDiag COVID-19 IgM/IgG Rapid Test	IgG	SENS	unk	75/95	78.9 (69.7-85.9)	0.0	7, 297; AU, US	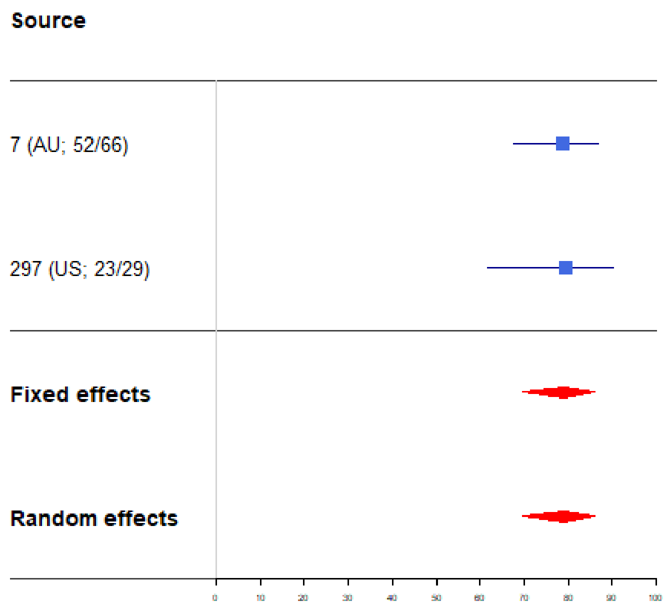
Ab (LFIA, POC)	VivaChek Biotech, VivaDiag COVID-19 IgM/IgG Rapid Test	IgG	SPEC	na	328/334	98.2 (96.1-99.2)	7.2	4, 7, 41, 242, 297; AU, BE, IT, NL, US	

										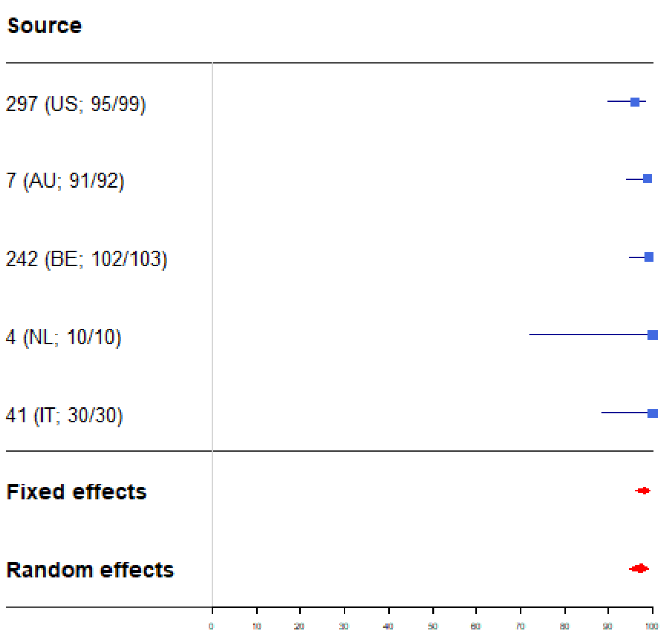
Ab (LFIA, POC)	VivaChek Biotech, VivaDiag COVID-19 IgM/IgG Rapid Test	IgG or IgM	SENS	mild/asymptomatic	1/1	100.0 (20.7-100.0)		4; NL	na	
Ab (LFIA, POC)	VivaChek Biotech, VivaDiag COVID-19 IgM/IgG Rapid Test	IgG or IgM	SENS	hospitalised	31/31	100.0 (89.0-100.0)	0.0	4, 242; BE, NL	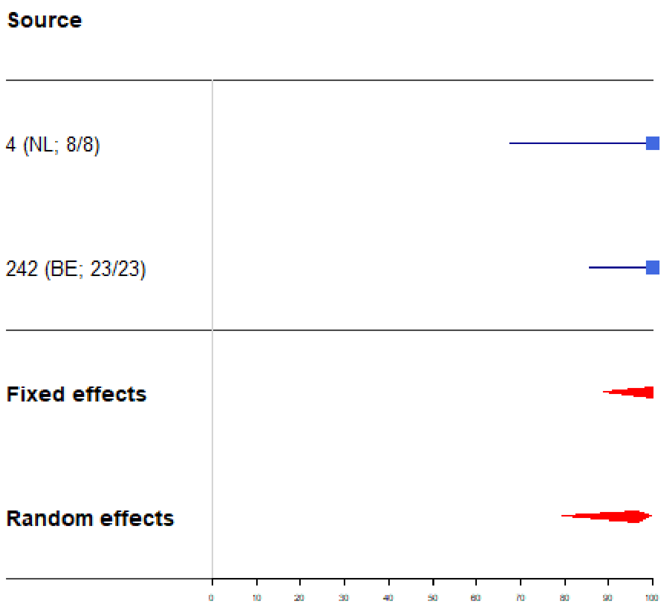	
Ab (LFIA, POC)	VivaChek Biotech, VivaDiag COVID-19 IgM/IgG Rapid Test	IgG or IgM	SENS	unk	76/95	80.0 (70.9-86.8)	0.0	7, 297; AU, US	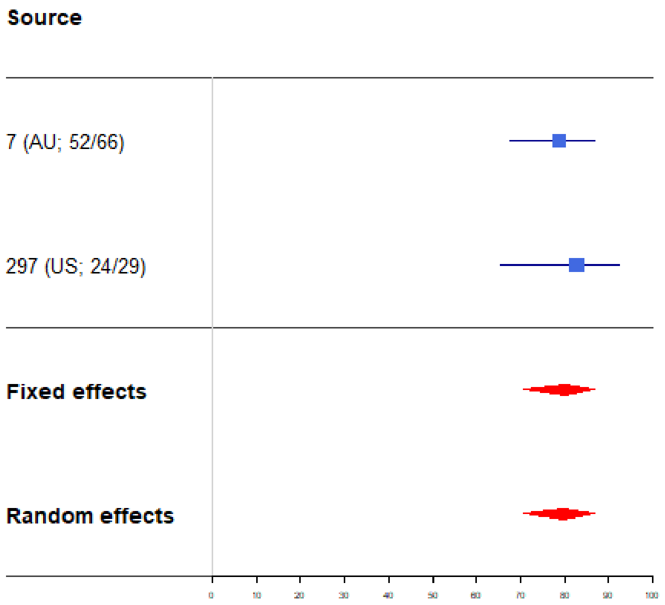	
Ab (LFIA, POC)	VivaChek Biotech, VivaDiag COVID-19 IgM/IgG Rapid Test	IgG or IgM	SPEC	na	316/324	97.5 (95.2-98.7)	21.9	7, 41, 242, 297; AU, BE, IT, US		

									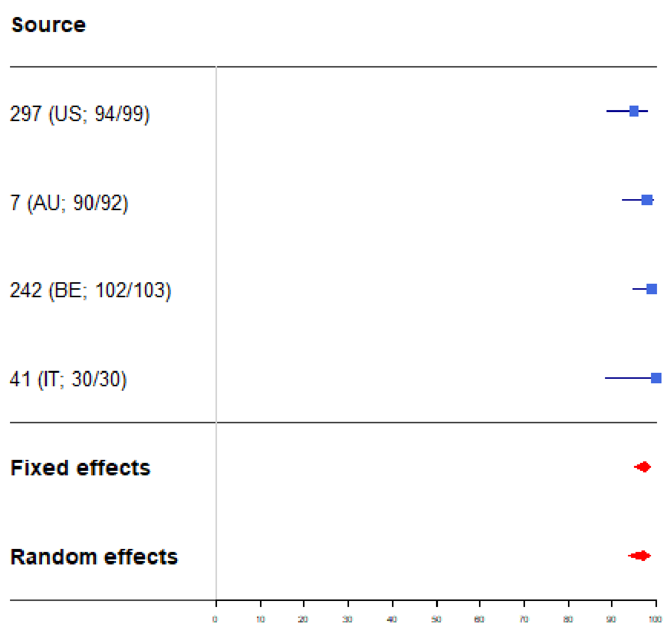
Ab (LFIA, POC)	VivaChek Biotech, VivaDiag COVID-19 IgM/IgG Rapid Test	IgM	SENS	hospitalised	23/23	100.0 (85.7-100.0)		242; BE	na
Ab (LFIA, POC)	VivaChek Biotech, VivaDiag COVID-19 IgM/IgG Rapid Test	IgM	SENS	unk	76/95	80.0 (70.9-86.8)	0.0	7, 297; AU, US	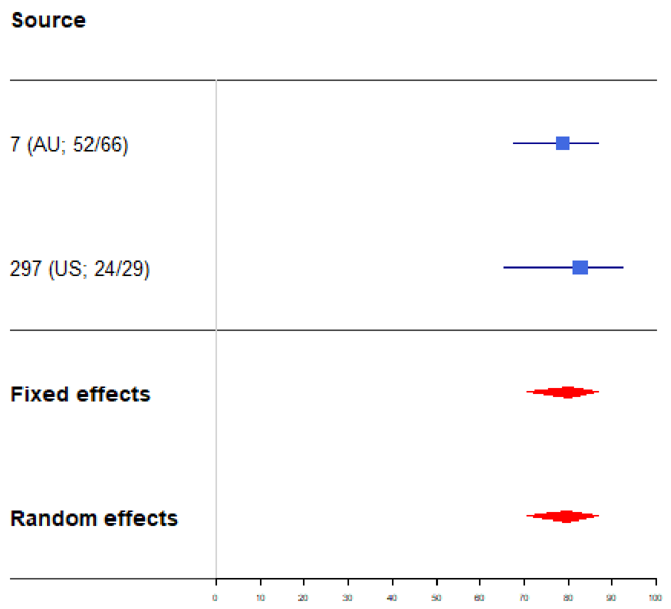
Ab (LFIA, POC)	VivaChek Biotech, VivaDiag COVID-19 IgM/IgG Rapid Test	IgM	SPEC	na	317/324	97.8 (95.6-98.9)	22.2	7, 41, 242, 297; AU, BE, IT, US	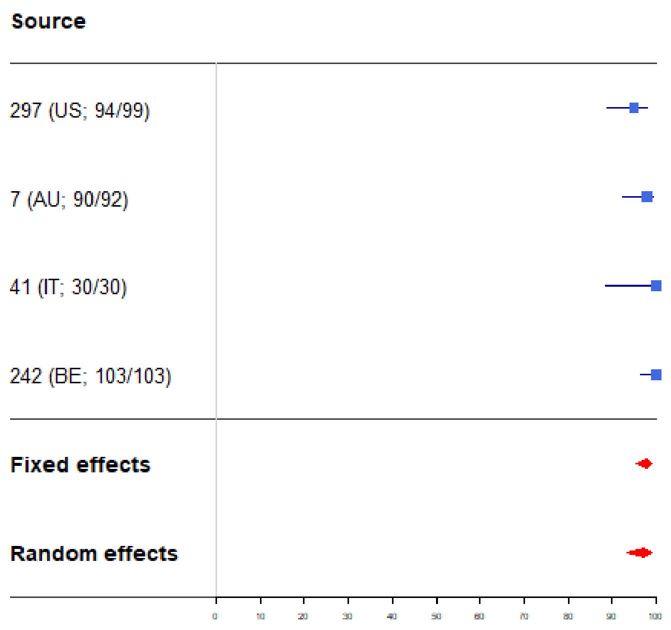
Ab (LFIA, POC)	Vomed, COVID-19 IgG/IgM Rapid Test Casette	IgG	SPEC	na	15/15	100.0 (79.6-100.0)		4; NL	na
Ab (LFIA, POC)	Vomed, COVID-19 IgG/IgM Rapid Test Casette	IgG or IgM	SENS	mild/asymptomatic	6/8	75.0 (40.9-92.9)		4; NL	na
Ab (LFIA, POC)	Vomed, COVID-19 IgG/IgM Rapid Test Casette	IgG or IgM	SENS	hospitalised	13/13	100.0 (77.2-100.0)		4; NL	na
Ab (LFIA, POC)	WHPM, Covisure Covid-19 IgM/IgG Rapid Test	IgG	SENS	unk	18/27	66.7 (47.8-81.4)		5; US	na
Ab (LFIA, POC)	WHPM, Covisure Covid-19 IgM/IgG Rapid Test	IgG	SPEC	na	68/70	97.1 (90.2-99.2)		5; US	na
Ab (LFIA, POC)	WHPM, Covisure Covid-19 IgM/IgG Rapid Test	IgG or IgM	SENS	unk	20/27	74.1 (55.3-86.8)		5; US	na
Ab (LFIA, POC)	WHPM, Covisure Covid-19 IgM/IgG Rapid Test	IgG or IgM	SPEC	na	68/70	97.1 (90.2-99.2)		5; US	na
Ab (LFIA, POC)	WHPM, Covisure Covid-19 IgM/IgG Rapid Test	IgM	SENS	unk	20/27	74.1 (55.3-86.8)		5; US	na
Ab (LFIA, POC)	WHPM, Covisure Covid-19 IgM/IgG Rapid Test	IgM	SPEC	na	68/70	97.1 (90.2-99.2)		5; US	na
Ab (LFIA, POC)	Wuhan Easy Diagnosis	IgG	SENS	unk	28/29	96.6 (82.8-		5; US	na

	Biomedicine, COVID-19 (SARS-CoV-2) IgM/IgG Antibody Test Kit					99.4)			
Ab (LFIA, POC)	Wuhan Easy Diagnosis Biomedicine, COVID-19 (SARS-CoV-2) IgM/IgG Antibody Test Kit	IgG	SPEC	na	69/70	98.6 (92.3-99.7)		5; US	na
Ab (LFIA, POC)	Wuhan Easy Diagnosis Biomedicine, COVID-19 (SARS-CoV-2) IgM/IgG Antibody Test Kit	IgG or IgM	SENS	unk	29/29	100.0 (88.3-100.0)		5; US	na
Ab (LFIA, POC)	Wuhan Easy Diagnosis Biomedicine, COVID-19 (SARS-CoV-2) IgM/IgG Antibody Test Kit	IgG or IgM	SPEC	na	62/70	88.6 (79.0-94.1)		5; US	na
Ab (LFIA, POC)	Wuhan Easy Diagnosis Biomedicine, COVID-19 (SARS-CoV-2) IgM/IgG Antibody Test Kit	IgM	SENS	unk	29/29	100.0 (88.3-100.0)		5; US	na
Ab (LFIA, POC)	Wuhan Easy Diagnosis Biomedicine, COVID-19 (SARS-CoV-2) IgM/IgG Antibody Test Kit	IgM	SPEC	na	63/70	90.0 (80.8-95.1)		5; US	na
Ab (LFIA, POC)	Wuhan UNscience Biotechnology, Covid-19 IgG/IgM Antibody Rapid Test Kit	IgG	SENS	mild/asymptomatic	17/20	85.0 (64.0-94.8)		278; NO	na
Ab (LFIA, POC)	Wuhan UNscience Biotechnology, Covid-19 IgG/IgM Antibody Rapid Test Kit	IgG	SENS	hospitalised	13/15	86.7 (62.1-96.3)		284; FR	na
Ab (LFIA, POC)	Wuhan UNscience Biotechnology, Covid-19 IgG/IgM Antibody Rapid Test Kit	IgG	SPEC	na	78/80	97.5 (91.3-99.3)	0.0	167, 284; ES, FR	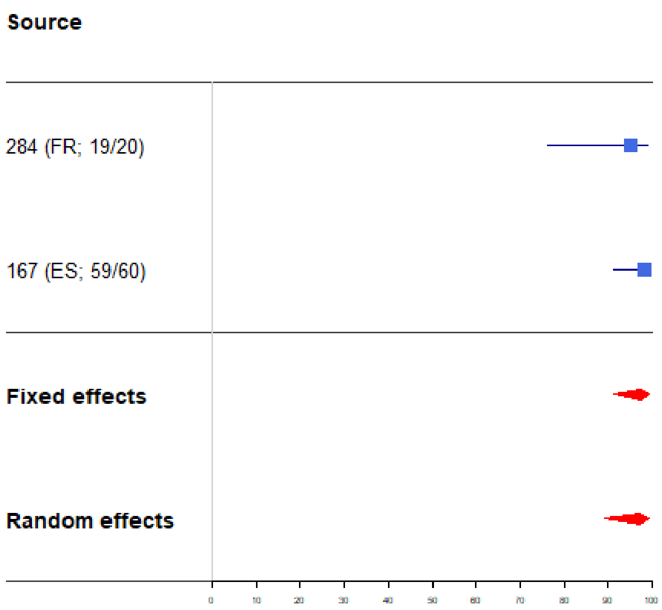
Ab (LFIA, POC)	Wuhan UNscience Biotechnology, Covid-19 IgG/IgM Antibody Rapid Test Kit	IgG or IgM	SPEC	na	53/60	88.3 (77.8-94.2)		167; ES	na
Ab (LFIA, POC)	Wuhan UNscience Biotechnology, Covid-19 IgG/IgM Antibody Rapid Test Kit	IgM	SENS	mild/asymptomatic	11/23	47.8 (29.2-67.0)		278; NO	na
Ab (LFIA, POC)	Wuhan UNscience Biotechnology, Covid-19 IgG/IgM Antibody Rapid Test Kit	IgM	SENS	hospitalised	12/15	80.0 (54.8-93.0)		284; FR	na
Ab (LFIA, POC)	Wuhan UNscience Biotechnology, Covid-19 IgG/IgM Antibody Rapid Test Kit	IgM	SPEC	na	67/80	83.8 (74.2-90.3)	83.7	167, 284; ES, FR	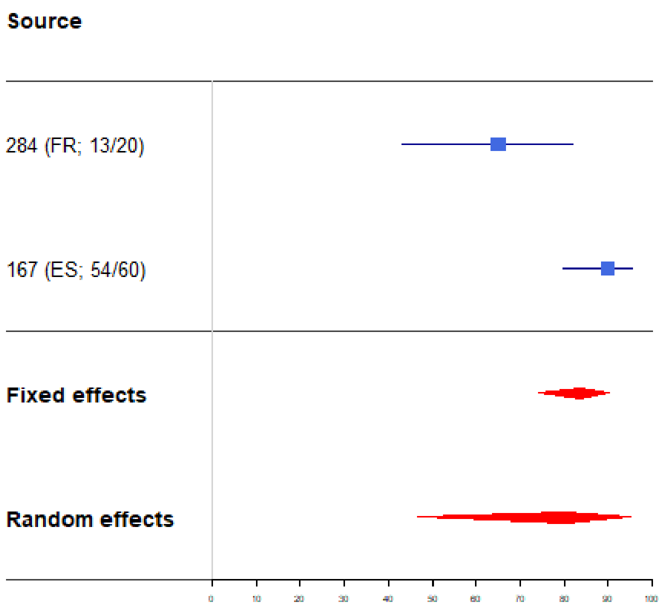
Ab (LFIA, POC)	Xiamen Biotime Biotechnology, SARS-CoV-2 IgG/IgM Rapid Qualitative Test Kit	IgG	SENS	unk	28/29	96.6 (82.8-99.4)		5; US	na
Ab (LFIA, POC)	Xiamen Biotime Biotechnology, SARS-CoV-2 IgG/IgM Rapid Qualitative Test Kit	IgG	SPEC	na	147/150	98.0 (94.3-99.3)	0.0	5, 141; FI, US	

									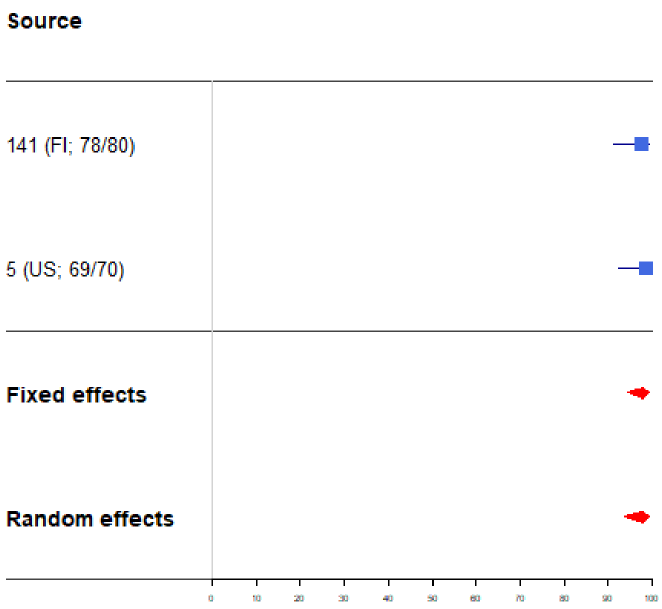
Ab (LFIA, POC)	Xiamen Biotime Biotechnology, SARS-CoV-2 IgG/IgM Rapid Qualitative Test Kit	IgG or IgM	SENS	unk	29/29	100.0 (88.3-100.0)		5; US	na
Ab (LFIA, POC)	Xiamen Biotime Biotechnology, SARS-CoV-2 IgG/IgM Rapid Qualitative Test Kit	IgG or IgM	SPEC	na	68/70	97.1 (90.2-99.2)		5; US	na
Ab (LFIA, POC)	Xiamen Biotime Biotechnology, SARS-CoV-2 IgG/IgM Rapid Qualitative Test Kit	IgM	SENS	unk	29/29	100.0 (88.3-100.0)		5; US	na
Ab (LFIA, POC)	Xiamen Biotime Biotechnology, SARS-CoV-2 IgG/IgM Rapid Qualitative Test Kit	IgM	SPEC	na	140/150	93.3 (88.2-96.3)	75.8	5, 141; FI, US	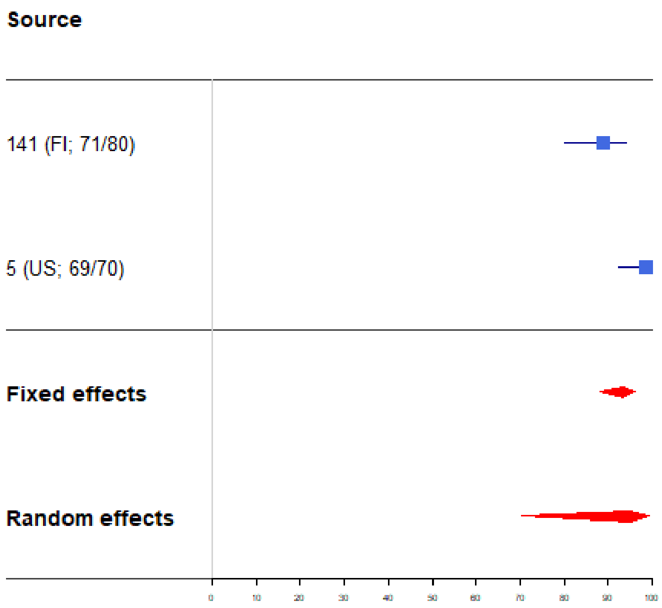
Ab (LFIA, POC)	Xiamen Boson Biotech, Rapid 2019-nCoV IgG/IgM Combo Test Card	IgG	SENS	mild/asymptomatic	10/15	66.7 (41.7-84.8)		4; NL	na
Ab (LFIA, POC)	Xiamen Boson Biotech, Rapid 2019-nCoV IgG/IgM Combo Test Card	IgG	SENS	hospitalised	39/40	97.5 (87.1-99.6)		4; NL	na
Ab (LFIA, POC)	Xiamen Boson Biotech, Rapid 2019-nCoV IgG/IgM Combo Test Card	IgG	SPEC	na	96/103	93.2 (86.6-96.7)		4; NL	na
Ab (LFIA, POC)	Xiamen Boson Biotech, Rapid 2019-nCoV IgG/IgM Combo Test Card	IgM	SENS	mild/asymptomatic	8/15	53.3 (30.1-75.2)		4; NL	na
Ab (LFIA, POC)	Xiamen Boson Biotech, Rapid 2019-nCoV IgG/IgM Combo Test Card	IgM	SENS	hospitalised	24/40	60.0 (44.6-73.7)		4; NL	na
Ab (CLIA)	Xiamen Innodx Biotech, Antibody test kit for 2019-nCoV	IgG or IgM	SENS	hospitalised	NaN/NaN	NaN (NaN-NaN)		164;	na
Ab (CLIA)	Xiamen Innodx Biotech, Antibody test kit for 2019-nCoV	IgG or IgM	SENS	unk	NaN/NaN	NaN (NaN-NaN)		291;	na
Ab (CLIA)	Xiamen Innodx Biotech, Antibody test kit for 2019-nCoV	IgG or IgM	SPEC	na	427/430	99.3 (98.0-99.8)	0.0	164, 291; CN(2)	

									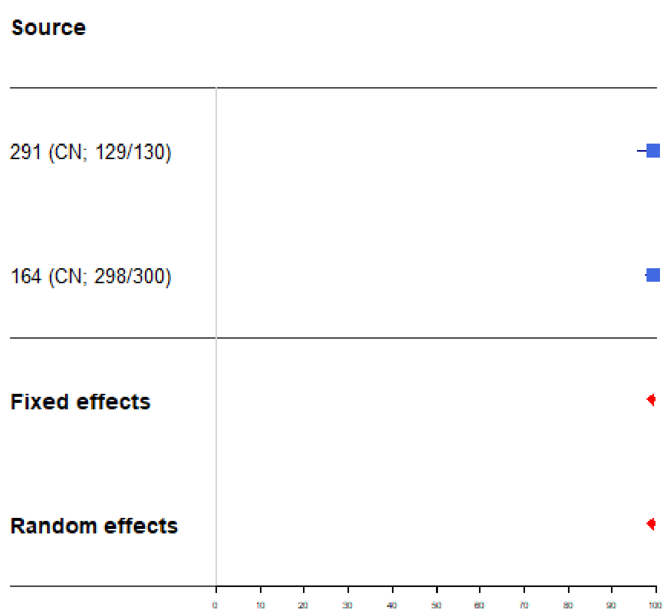
Ab (CLIA)	Xiamen Innodx Biotech, Antibody test kit for 2019-nCoV	IgM	SENS	hospitalised	NaN/NaN	NaN (NaN-NaN)		164;	na
Ab (CLIA)	Xiamen Innodx Biotech, Antibody test kit for 2019-nCoV	IgM	SPEC	na	298/300	99.3 (97.6-99.8)		164; CN	na
Ab (LFIA, POC)	Zentech, QuickZen COVID-19 IgM / IgG	IgG	SPEC	na	72/72	100.0 (94.9-100.0)		177; BE	na
Ab (LFIA, POC)	Zentech, QuickZen COVID-19 IgM / IgG	IgG or IgM	SENS	hospitalised	30/33	90.9 (76.4-96.9)		177; BE	na
Ab (LFIA, POC)	Zentech, QuickZen COVID-19 IgM / IgG	IgG or IgM	SPEC	na	72/72	100.0 (94.9-100.0)		177; BE	na
Ab (LFIA, POC)	Zentech, QuickZen COVID-19 IgM / IgG	IgM	SPEC	na	72/72	100.0 (94.9-100.0)		177; BE	na
Ab (LFIA, POC)	Zhejiang Orient Gene Biotech, COVID-19 IgG/IgM Rapid Test Cassette	IgG	SENS	mild/asymptomatic	69/74	93.2 (85.1-97.1)		4; NL	na
Ab (LFIA, POC)	Zhejiang Orient Gene Biotech, COVID-19 IgG/IgM Rapid Test Cassette	IgG	SENS	hospitalised	116/120	96.7 (91.7-98.7)	0.0	4, 15, 242; BE, CH, NL	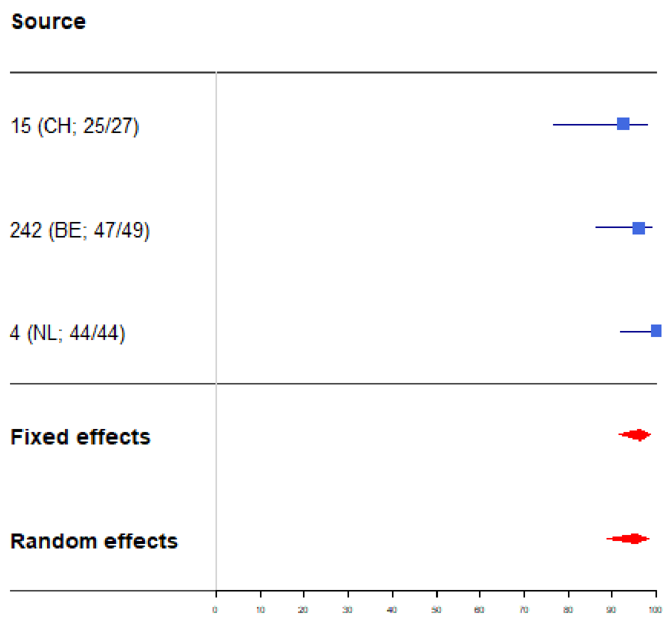
Ab (LFIA, POC)	Zhejiang Orient Gene Biotech, COVID-19 IgG/IgM Rapid Test Cassette	IgG	SENS	unk	85/92	92.4 (85.1-96.3)	0.0	57, 85; FR, SE	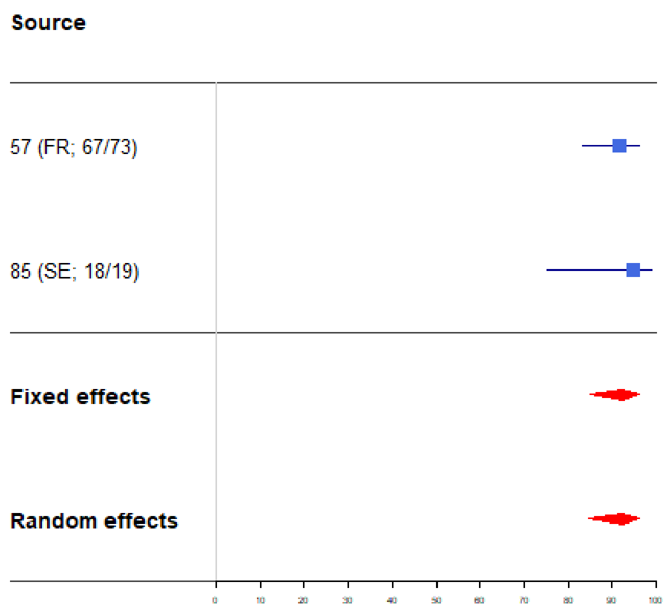
Ab (LFIA, POC)	Zhejiang Orient Gene Biotech, COVID-19 IgG/IgM Rapid Test Cassette	IgG	SPEC	na	555/568	97.7 (96.1-98.7)	47.0	4, 15, 57, 85, 242; BE, CH, FR, NL, SE	

									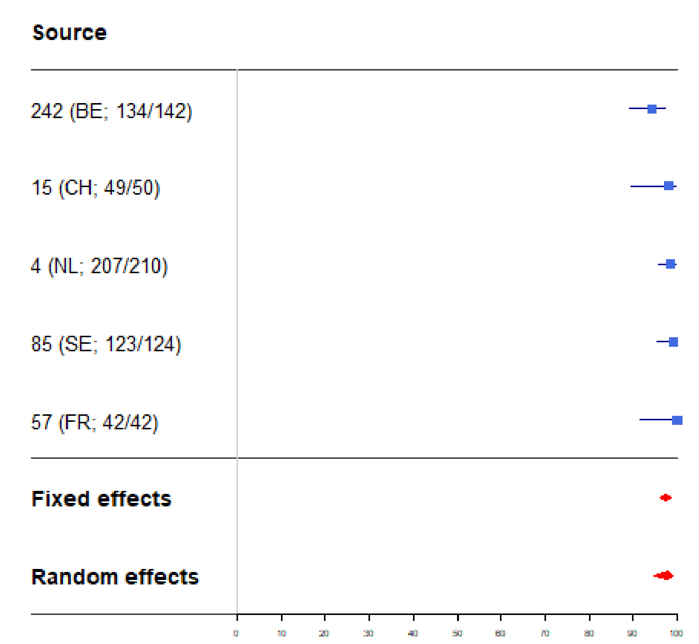
Ab (LFIA, POC)	Zhejiang Orient Gene Biotech, COVID-19 IgG/IgM Rapid Test Cassette	IgG or IgM	SENS	hospitalised	48/49	98.0 (89.3-99.6)		242; BE	na
Ab (LFIA, POC)	Zhejiang Orient Gene Biotech, COVID-19 IgG/IgM Rapid Test Cassette	IgG or IgM	SENS	unk	72/73	98.6 (92.6-99.8)		57; FR	na
Ab (LFIA, POC)	Zhejiang Orient Gene Biotech, COVID-19 IgG/IgM Rapid Test Cassette	IgG or IgM	SPEC	na	310/332	93.4 (90.2-95.6)	44.1	57, 75, 212, 242; BE, FR, NL(2)	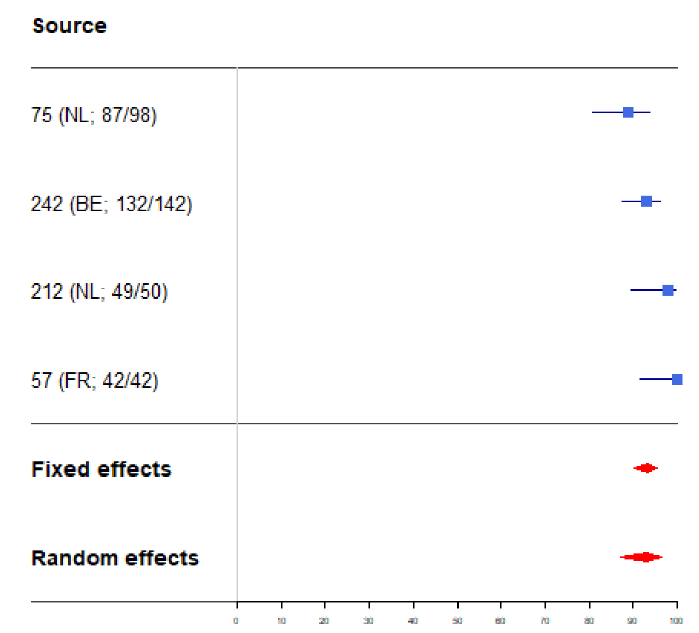
Ab (LFIA, POC)	Zhejiang Orient Gene Biotech, COVID-19 IgG/IgM Rapid Test Cassette	IgM	SENS	mild/asymptomatic	70/74	94.6 (86.9-97.9)		4; NL	na
Ab (LFIA, POC)	Zhejiang Orient Gene Biotech, COVID-19 IgG/IgM Rapid Test Cassette	IgM	SENS	hospitalised	80/93	86.0 (77.5-91.6)	0.0	4, 242; BE, NL	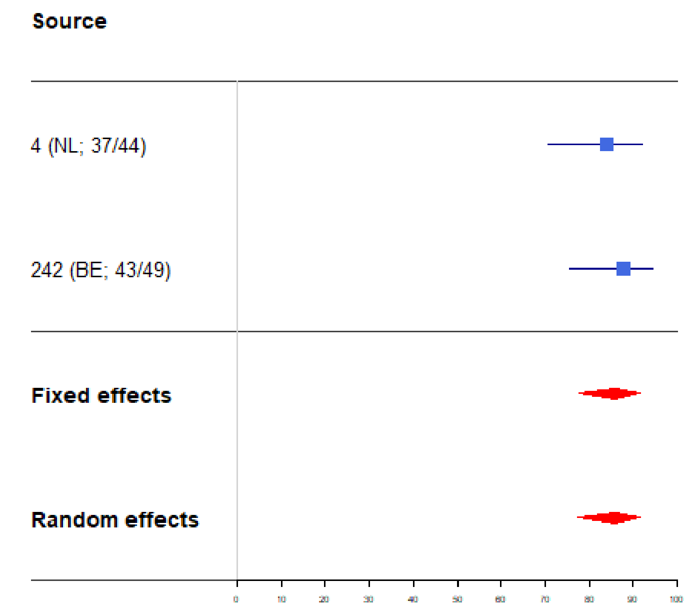
Ab (LFIA, POC)	Zhejiang Orient Gene Biotech, COVID-19 IgG/IgM Rapid Test Cassette	IgM	SENS	unk	76/92	82.6 (73.6-89.0)	68.4	57, 85; FR, SE	

									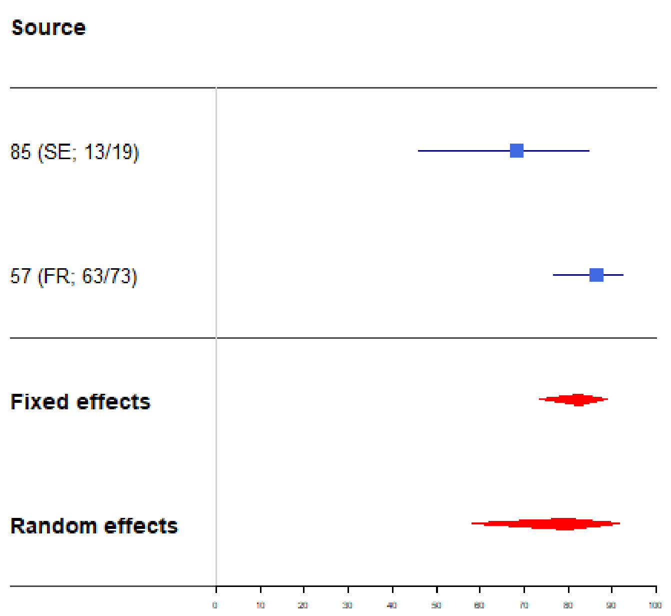
Ab (LFIA, POC)	Zhejiang Orient Gene Biotech, COVID-19 IgG/IgM Rapid Test Cassette	IgM	SPEC	na	303/308	98.4 (96.3-99.3)	30.9	57, 85, 242; BE, FR, SE	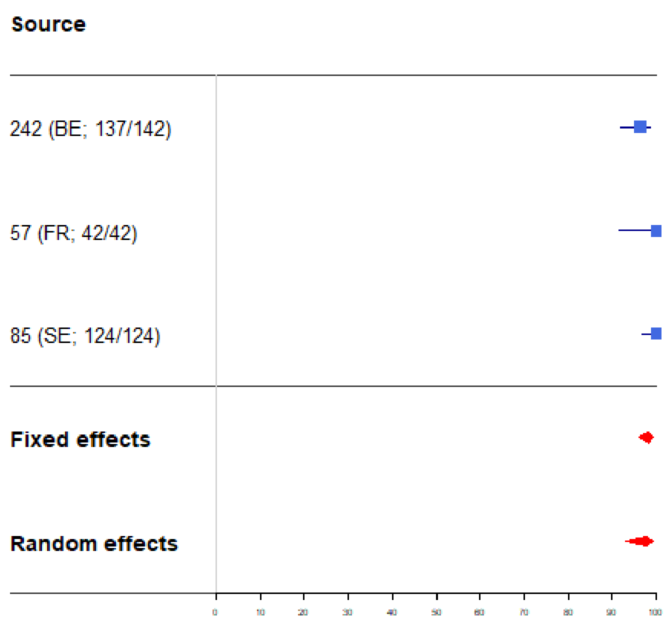
Ab (LFIA, POC)	Zhongshan Bio-Tech, SARS-CoV-2 IgM-IgG (GICA)	IgG	SENS	unk	28/29	96.6 (82.8-99.4)		5; US	na
Ab (LFIA, POC)	Zhongshan Bio-Tech, SARS-CoV-2 IgM-IgG (GICA)	IgG	SPEC	na	59/70	84.3 (74.0-91.0)		5; US	na
Ab (LFIA, POC)	Zhongshan Bio-Tech, SARS-CoV-2 IgM-IgG (GICA)	IgG or IgM	SENS	unk	28/29	96.6 (82.8-99.4)		5; US	na
Ab (LFIA, POC)	Zhongshan Bio-Tech, SARS-CoV-2 IgM-IgG (GICA)	IgG or IgM	SPEC	na	58/70	82.9 (72.4-89.9)		5; US	na
Ab (LFIA, POC)	Zhongshan Bio-Tech, SARS-CoV-2 IgM-IgG (GICA)	IgM	SENS	unk	14/29	48.3 (31.4-65.6)		5; US	na
Ab (LFIA, POC)	Zhongshan Bio-Tech, SARS-CoV-2 IgM-IgG (GICA)	IgM	SPEC	na	68/70	97.1 (90.2-99.2)		5; US	na
Ab (LFIA, POC)	Zhuhai Encode Medical Engineering, SARS-CoV-2 IgG/IgM Rapid Test	IgG	SENS	mild/asymptomatic	17/23	73.9 (53.5-87.5)		278; NO	na
Ab (LFIA, POC)	Zhuhai Encode Medical Engineering, SARS-CoV-2 IgG/IgM Rapid Test	IgG or IgM	SENS	unk	NaN/NaN	NaN (NaN-NaN)		218;	na
Ab (LFIA, POC)	Zhuhai Encode Medical Engineering, SARS-CoV-2 IgG/IgM Rapid Test	IgG or IgM	SPEC	na	100/100	100.0 (96.3-100.0)		218; UK	na
Ab (LFIA, POC)	Zhuhai Encode Medical Engineering, SARS-CoV-2 IgG/IgM Rapid Test	IgM	SENS	mild/asymptomatic	17/23	73.9 (53.5-87.5)		278; NO	na
Ab (LFIA, POC)	Zhuhai Livzon Diagnostics, Livzon IgM/IgG Diagnostic Kit for IgM/IgG Antibody to Coronovirus (SARS-Cov-2) Lateral Flow	IgG	SENS	unk	19/29	65.5 (47.3-80.1)		5; US	na
Ab (LFIA, POC)	Zhuhai Livzon Diagnostics, Livzon IgM/IgG Diagnostic Kit for IgM/IgG Antibody to Coronovirus (SARS-Cov-2) Lateral Flow	IgG	SPEC	na	70/70	100.0 (94.8-100.0)		5; US	na
Ab (LFIA, POC)	Zhuhai Livzon Diagnostics, Livzon IgM/IgG Diagnostic Kit for IgM/IgG Antibody to Coronovirus (SARS-Cov-2) Lateral Flow	IgG or IgM	SENS	unk	25/29	86.2 (69.4-94.5)		5; US	na
Ab (LFIA, POC)	Zhuhai Livzon Diagnostics, Livzon IgM/IgG Diagnostic Kit	IgG or IgM	SPEC	na	68/70	97.1 (90.2-99.2)		5; US	na

	for IgM/IgG Antibody to Coronavirus (SARS-Cov-2) Lateral Flow								
Ab (LFIA, POC)	Zhuhai Livzon Diagnostics, Livzon IgM/IgG Diagnostic Kit for IgM/IgG Antibody to Coronavirus (SARS-Cov-2) Lateral Flow	IgM	SENS	unk	25/29	86.2 (69.4-94.5)		5; US	na
Ab (LFIA, POC)	Zhuhai Livzon Diagnostics, Livzon IgM/IgG Diagnostic Kit for IgM/IgG Antibody to Coronavirus (SARS-Cov-2) Lateral Flow	IgM	SPEC	na	68/70	97.1 (90.2-99.2)		5; US	na
Ab (LFIA, POC)	Zhuhai Livzon Pharmaceutical Group, Diagnostic Kit for IgM / IgG Antibody to Coronavirus (SARS-CoV-2) (Lateral Flow)	IgG	SENS	hospitalised	140/162	86.4 (80.3-90.9)	21.0	219, 284, 304; CN(2), FR	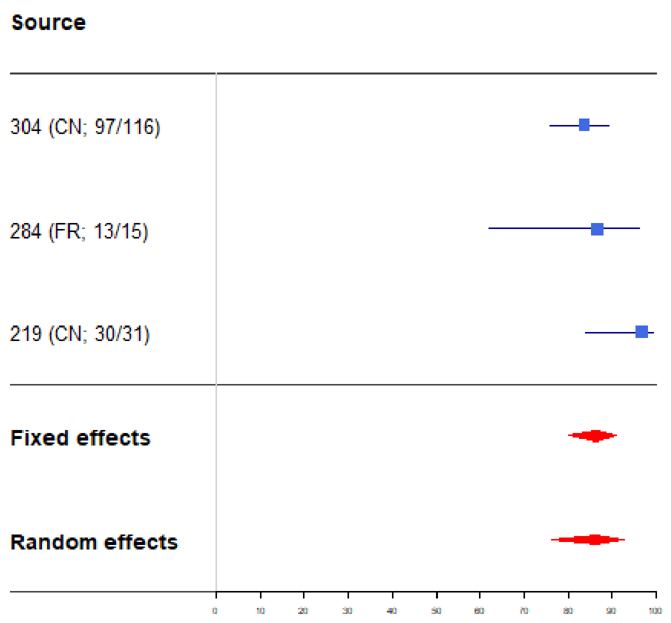
Ab (LFIA, POC)	Zhuhai Livzon Pharmaceutical Group, Diagnostic Kit for IgM / IgG Antibody to Coronavirus (SARS-CoV-2) (Lateral Flow)	IgG	SENS	unk	16/27	59.3 (40.7-75.5)		5; US	na
Ab (LFIA, POC)	Zhuhai Livzon Pharmaceutical Group, Diagnostic Kit for IgM / IgG Antibody to Coronavirus (SARS-CoV-2) (Lateral Flow)	IgG	SPEC	na	147/150	98.0 (94.3-99.3)	6.3	5, 284, 304; CN, FR, US	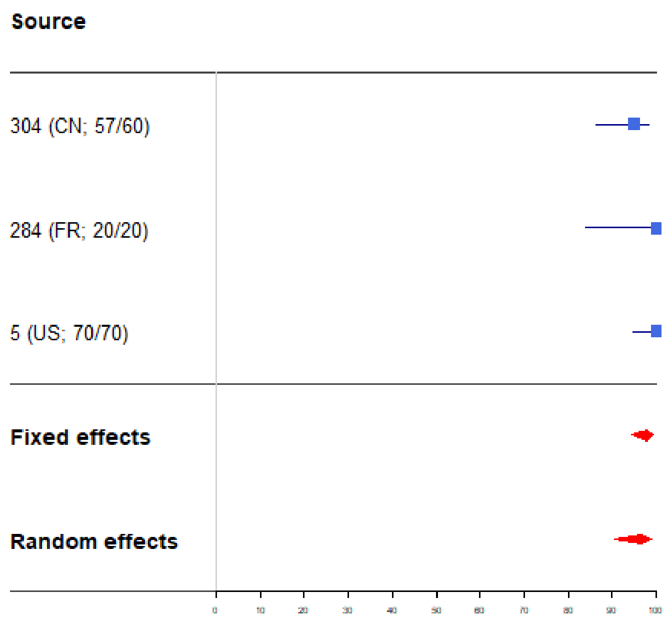
Ab (LFIA, POC)	Zhuhai Livzon Pharmaceutical Group, Diagnostic Kit for IgM / IgG Antibody to Coronavirus (SARS-CoV-2) (Lateral Flow)	IgG or IgM	SENS	hospitalised	30/31	96.8 (83.8-99.4)		219; CN	na
Ab (LFIA, POC)	Zhuhai Livzon Pharmaceutical Group, Diagnostic Kit for IgM / IgG Antibody to Coronavirus (SARS-CoV-2) (Lateral Flow)	IgG or IgM	SENS	unk	25/27	92.6 (76.6-97.9)		5; US	na
Ab (LFIA, POC)	Zhuhai Livzon Pharmaceutical Group, Diagnostic Kit for IgM / IgG Antibody to Coronavirus (SARS-CoV-2) (Lateral Flow)	IgG or IgM	SPEC	na	70/70	100.0 (94.8-100.0)		5; US	na
Ab (LFIA, POC)	Zhuhai Livzon Pharmaceutical Group, Diagnostic Kit for IgM / IgG Antibody to Coronavirus (SARS-CoV-2) (Lateral Flow)	IgM	SENS	hospitalised	123/162	75.9 (68.8-81.9)	0.0	219, 284, 304; CN(2), FR	

									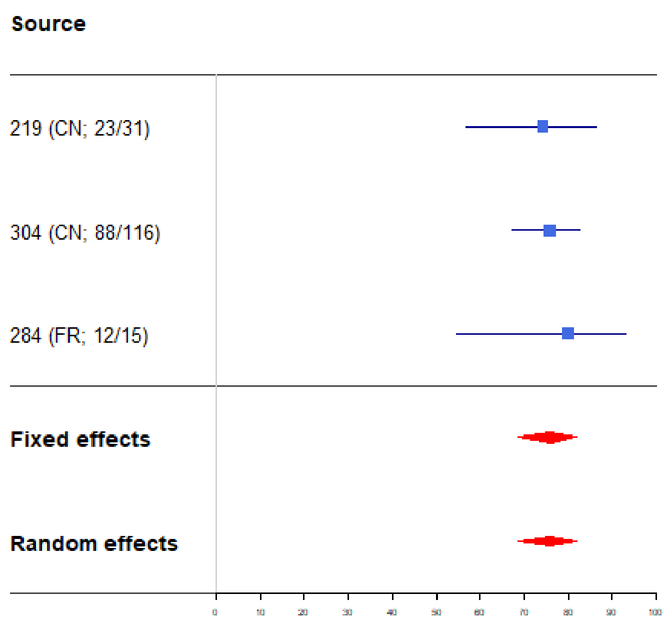
Ab (LFIA, POC)	Zhuhai Livzon Pharmaceutical Group, Diagnostic Kit for IgM / IgG Antibody to Coronavirus (SARS-CoV-2) (Lateral Flow)	IgM	SENS	unk	25/27	92.6 (76.6-97.9)		5; US	na
Ab (LFIA, POC)	Zhuhai Livzon Pharmaceutical Group, Diagnostic Kit for IgM / IgG Antibody to Coronavirus (SARS-CoV-2) (Lateral Flow)	IgM	SPEC	na	149/150	99.3 (96.3-99.9)	5.3	5, 284, 304; CN, FR, US	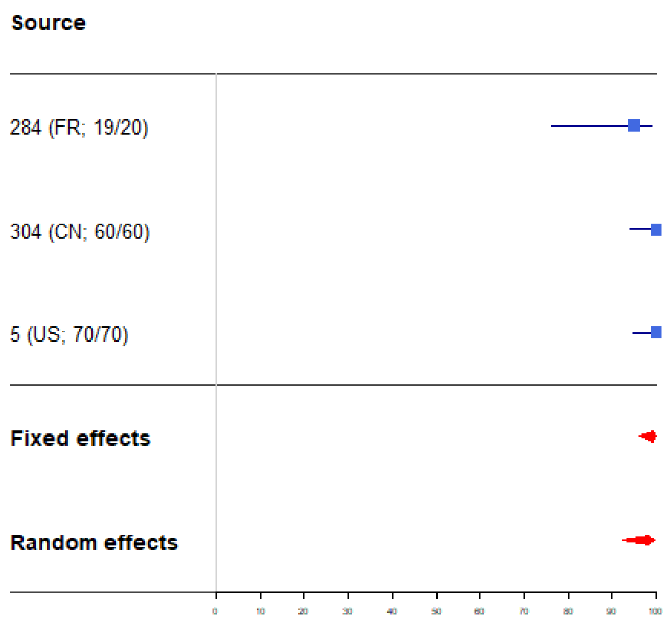
Ag (LFIA, POC)	Coris BioConcept, C-1023/TB Covid-19 Respi-Strip	Ag	SENS	unk	1/10	10.0 (1.8-40.4)		236; PT	na
Ag (LFIA, POC)	Shenzhen Bioeasy Biotechnology, Bioeasy 2019-nCoV Ag Fluorescence Rapid Test Kit	Ag	SENS	unk	4/12	33.3 (13.8-60.9)		236; PT	na
RNA (PCR)	1drop, 1copy COVID-19 qPCR Multi Kit	RdRP or E	SENS	unk	6/6	100.0 (61.0-100.0)		197; PL	na
RNA (PCR)	AB Analytica, RealQuality RQ-2019-nCoV	E	SENS	unk	33/33	100.0 (89.6-100.0)		198; HR	na
RNA (PCR)	AB Analytica, RealQuality RQ-2019-nCoV	RdRP	SENS	unk	32/33	97.0 (84.7-99.5)		198; HR	na
RNA (LAMP, POC)	Abbott, ID NOW COVID-19	RdRP	SENS	unk	385/483	79.7 (75.9-83.1)	82.8	21, 52, 82, 170, 176, 251, 328; US(7)	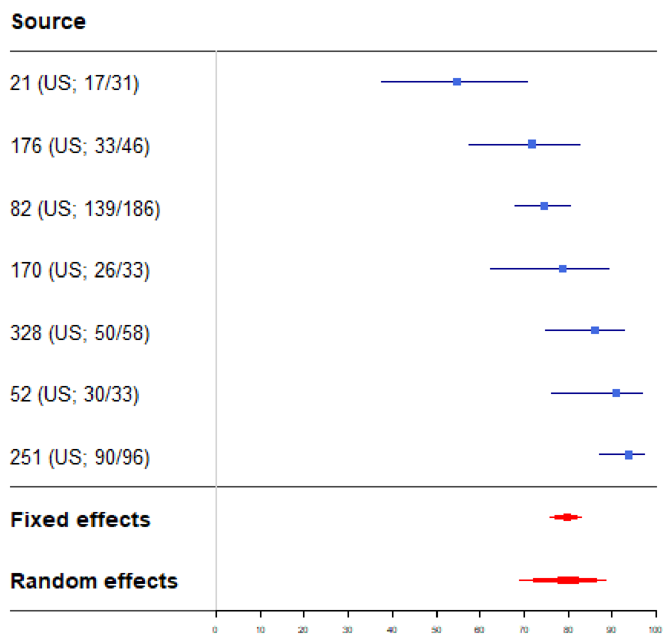
RNA (PCR)	Abbott, RealTime SARS-CoV-2	RdRP or N	SENS	unk	139/141	98.6 (95.0-99.6)		82; US	na
RNA (PCR)	Altona Diagnostics,	E	SENS	unk	89/101	88.1 (80.4-	0.0	36, 184, 195, 330;	

	RealStar SARS-CoV-2 RT-PCR Kit 1.0					93.1)		CH, FR, NL, US	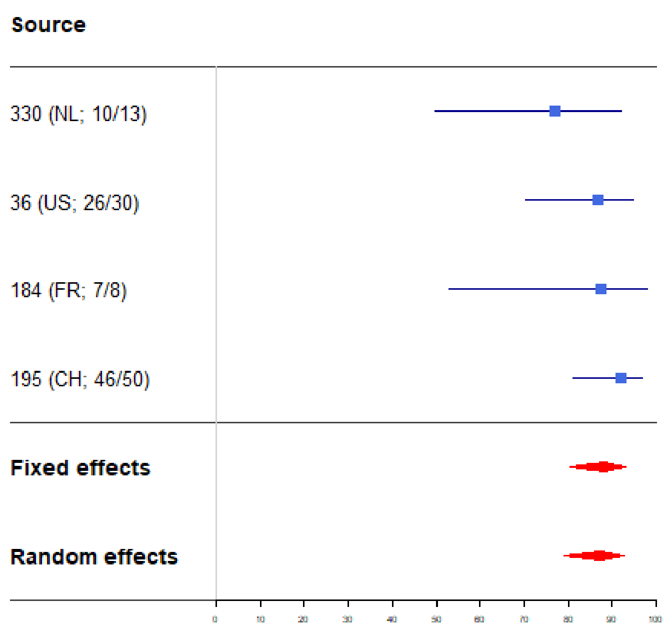
RNA (PCR)	Altona Diagnostics, RealStar SARS-CoV-2 RT-PCR Kit 1.0	E	SPEC	na	112/112	100.0 (96.7-100.0)	5.9	195, 330; CH, NL	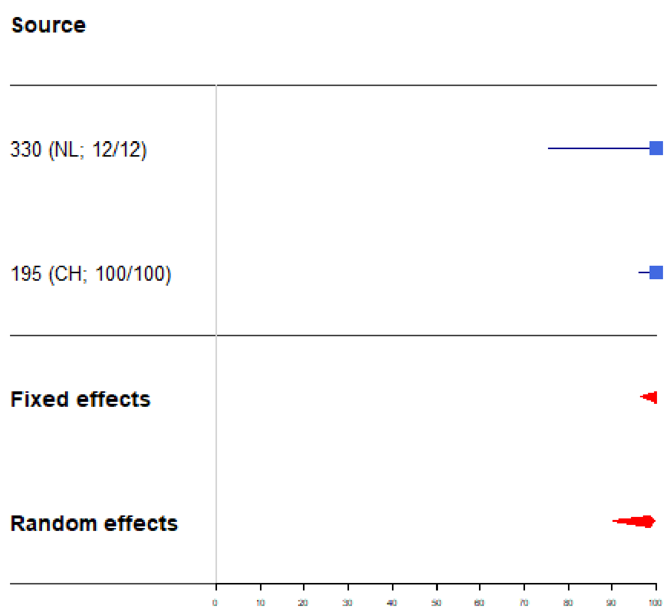
RNA (PCR)	Altona Diagnostics, RealStar SARS-CoV-2 RT-PCR Kit 1.0	S	SENS	unk	88/101	87.1 (79.2-92.3)	6.2	36, 184, 195, 330; CH, FR, NL, US	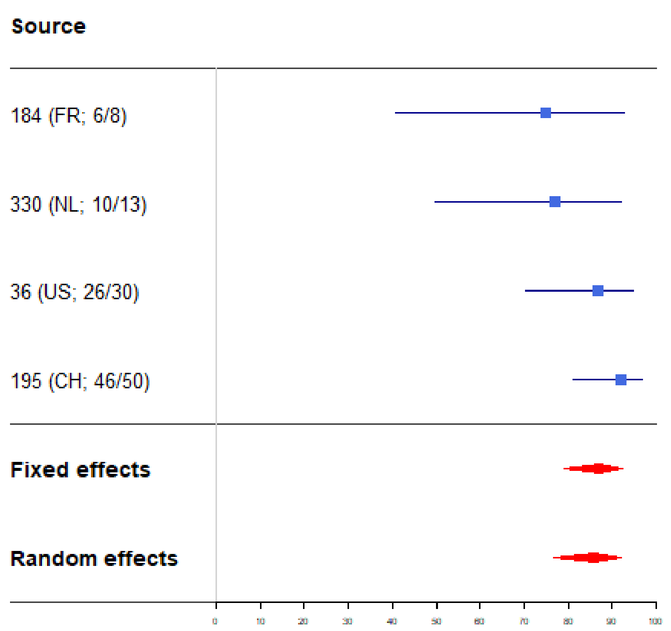
RNA (PCR)	Altona Diagnostics, RealStar SARS-CoV-2 RT-PCR Kit 1.0	S	SPEC	na	112/112	100.0 (96.7-100.0)	5.9	195, 330; CH, NL	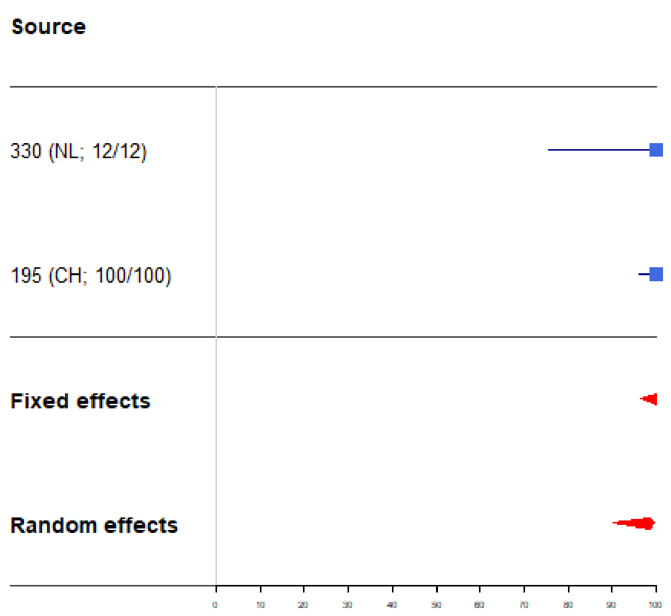
RNA (PCR)	Altona Diagnostics,	S or E	SENS	unk	169/207	81.6 (75.8-	61.2	184, 288, 298, 330;	

	RealStar SARS-CoV-2 RT-PCR Kit 1.0					86.3)		FR(3), NL	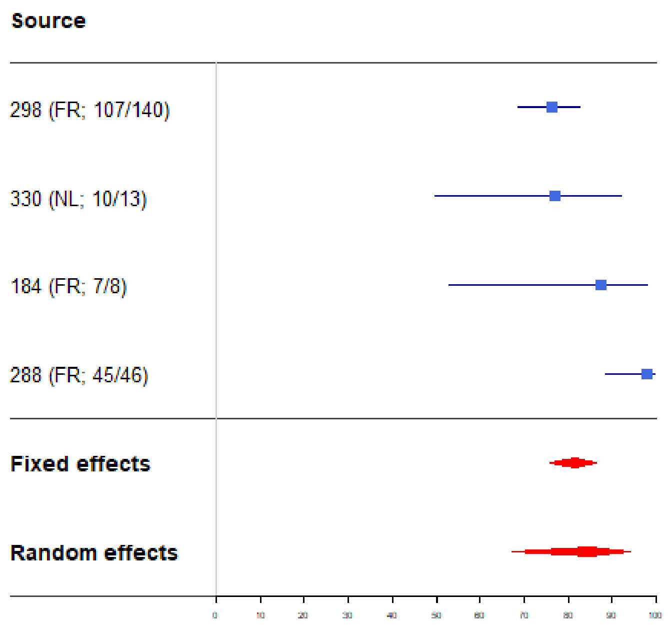
RNA (PCR)	Altona Diagnostics, RealStar SARS-CoV-2 RT-PCR Kit 1.0	S or E	SPEC	na	237/237	100.0 (98.4-100.0)	0.0	183, 298, 330; FR, NL, UK	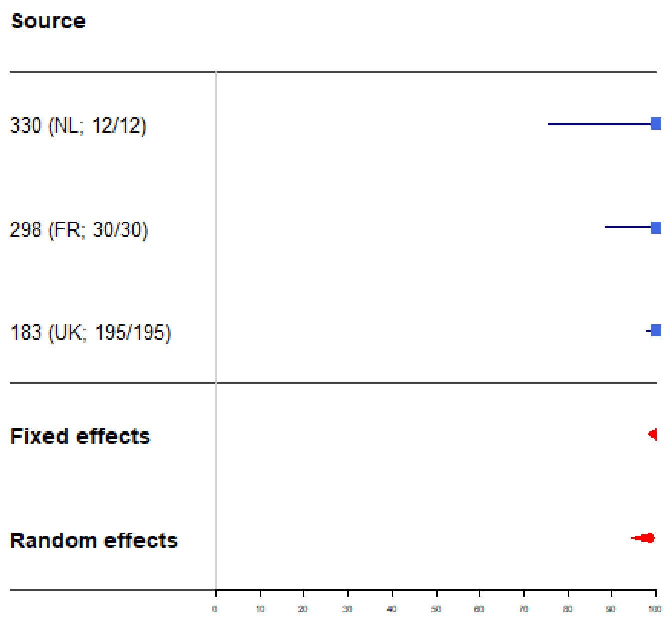
RNA (PCR)	Anatolia Geneworks, Bosphore Novel Coronavirus (2019-nCoV) Detection Kit V2	E	SENS	unk	8/21	38.1 (20.8-59.1)		199; PL	na
RNA (PCR)	Anatolia Geneworks, Bosphore Novel Coronavirus (2019-nCoV) Detection Kit V2	ORF1ab	SENS	unk	8/21	38.1 (20.8-59.1)		199; PL	na
RNA (PCR)	Anatolia Geneworks, Bosphore Novel Coronavirus (2019-nCoV) Detection Kit V2	ORF1ab or E	SENS	unk	8/21	38.1 (20.8-59.1)		199; PL	na
RNA (PCR)	Anatolia Geneworks, Bosphore Novel Coronavirus (2019-nCoV) Detection Kit V2	RdRP	SENS	unk	20/21	95.2 (77.3-99.2)		197; PL	na
RNA (OMEGA)	Atila BioSystems, Atila iAMP COVID-19 Detection (isothermal detection)	N	SENS	unk	50/50	100.0 (92.9-100.0)		195; CH	na
RNA (OMEGA)	Atila BioSystems, Atila iAMP COVID-19 Detection (isothermal detection)	N	SPEC	na	100/100	100.0 (96.3-100.0)		195; CH	na
RNA (OMEGA)	Atila BioSystems, Atila iAMP COVID-19 Detection (isothermal detection)	ORF1ab	SENS	unk	50/50	100.0 (92.9-100.0)		195; CH	na
RNA (OMEGA)	Atila BioSystems, Atila iAMP COVID-19 Detection (isothermal detection)	ORF1ab	SPEC	na	99/100	99.0 (94.6-99.8)		195; CH	na
RNA (OMEGA)	Atila BioSystems, Atila iAMP COVID-19 Detection (isothermal detection)	ORF1ab or N	SENS	unk	24/29	82.8 (65.5-92.4)		36; US	na
RNA (PCR)	AusDiagnostics, Coronavirus Typing Assay	ORF1ab	SENS	unk	125/125	100.0 (97.0-100.0)		19; AU	na
RNA (PCR)	AusDiagnostics, Coronavirus Typing Assay	ORF1ab	SPEC	na	254/254	100.0 (98.5-100.0)	0.0	19, 185; AU, UK	

									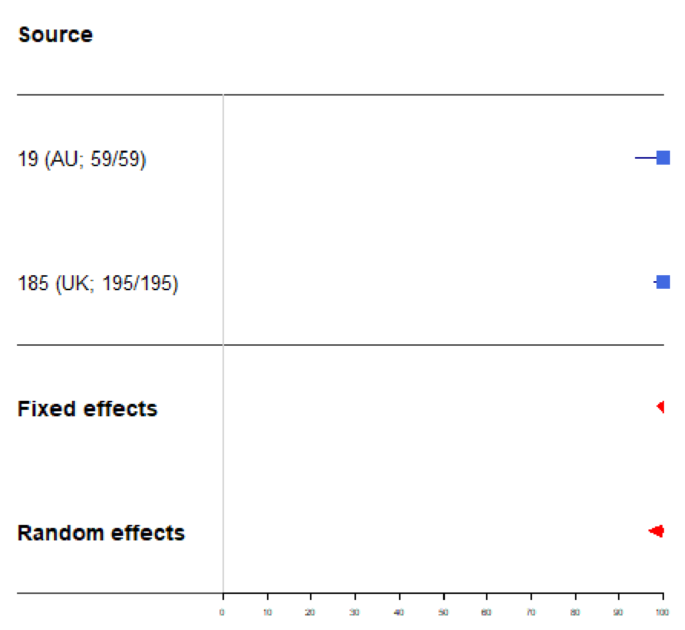
RNA (PCR)	BD LAXLUO, BioGX SARS-CoV-2 Open System Reagents for BD MAX	N	SENS	unk	7/7	100.0 (64.6-100.0)		186; FR	na
RNA (PCR)	BGI, Real-time fluorescent RT-PCR kit for detecting 2019 nCoV	ORF1ab	SENS	unk	137/146	93.8 (88.7-96.7)	27.8	168, 187, 195, 330; CH, JP, NL, PL	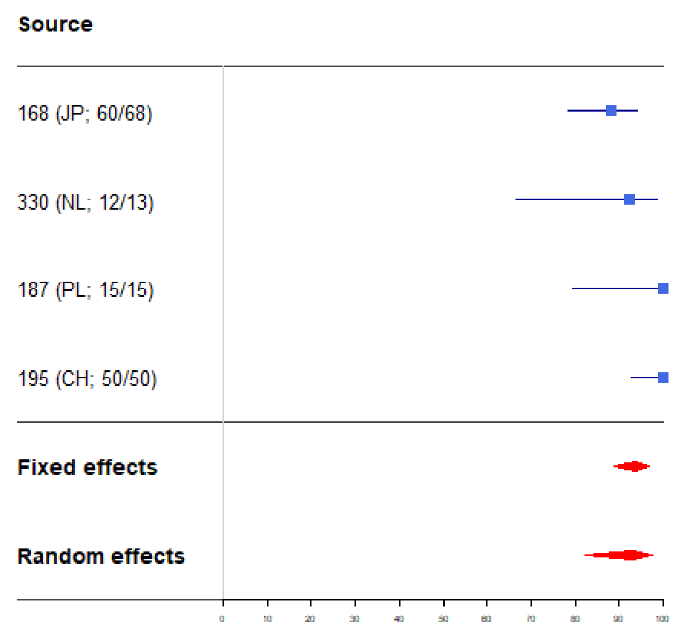
RNA (PCR)	BGI, Real-time fluorescent RT-PCR kit for detecting 2019 nCoV	ORF1ab	SPEC	na	111/112	99.1 (95.1-99.8)	0.0	195, 330; CH, NL	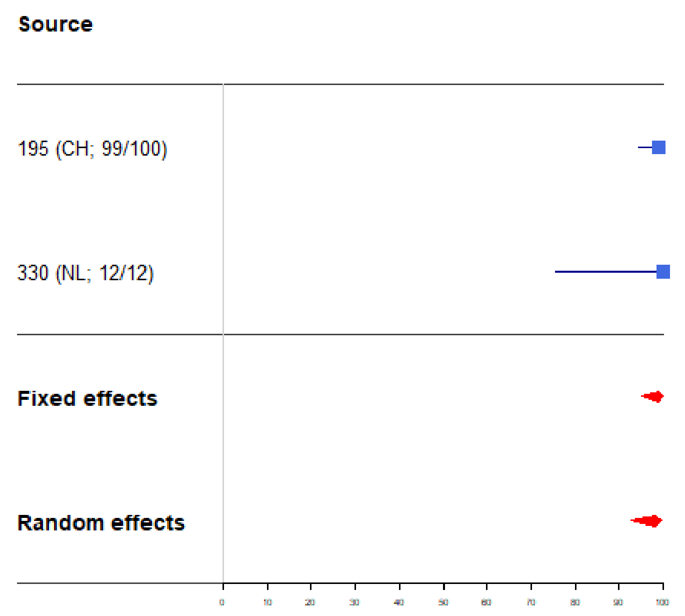
RNA (PCR)	BIOMAXIMA, SARS-CoV-2 Real Time PCR LAB-KIT	ORF1ab or N	SENS	unk	24/25	96.0 (80.5-99.3)		243; PL	na
RNA (PCR)	Beijing Wantai Biological Pharmacy Enterprise, Wantai SARS-CoV-2 RT-PCR Kit	N	SENS	unk	50/50	100.0 (92.9-100.0)		195; CH	na
RNA (PCR)	Beijing Wantai Biological Pharmacy Enterprise, Wantai SARS-CoV-2 RT-PCR Kit	N	SPEC	na	100/100	100.0 (96.3-100.0)		195; CH	na
RNA (PCR)	Beijing Wantai Biological Pharmacy Enterprise, Wantai SARS-CoV-2 RT-PCR Kit	ORF1ab	SENS	unk	50/50	100.0 (92.9-100.0)		195; CH	na
RNA (PCR)	Beijing Wantai Biological Pharmacy Enterprise, Wantai SARS-CoV-2 RT-PCR Kit	ORF1ab	SPEC	na	100/100	100.0 (96.3-100.0)		195; CH	na
RNA (PCR)	BioFire Defense, BioFire COVID-19 Test	ORF1ab	SENS	unk	74/75	98.7 (92.8-99.8)		260; US	na
RNA (PCR)	BioMerieux, ARGENE	E	SENS	unk	6/6	100.0		188; FR	na

	SARS-CoV-2 R-GENE					(61.0-100.0)			
RNA (PCR)	BioMerieux, ARGENE SARS-CoV-2 R-GENE	E or N	SENS	unk	6/6	100.0 (61.0-100.0)		188; FR	na
RNA (PCR)	BioMerieux, ARGENE SARS-CoV-2 R-GENE	N	SENS	unk	56/56	100.0 (93.6-100.0)	0.7	188, 195; CH, FR	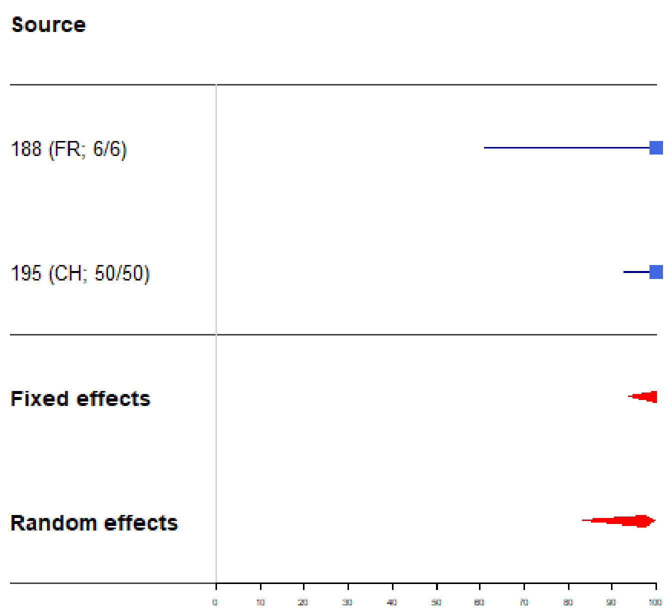
RNA (PCR)	BioMerieux, ARGENE SARS-CoV-2 R-GENE	N	SPEC	na	100/100	100.0 (96.3-100.0)		195; CH	na
RNA (PCR)	BioMerieux, ARGENE SARS-CoV-2 R-GENE	RdRP	SENS	unk	54/56	96.4 (87.9-99.0)	0.0	188, 195; CH, FR	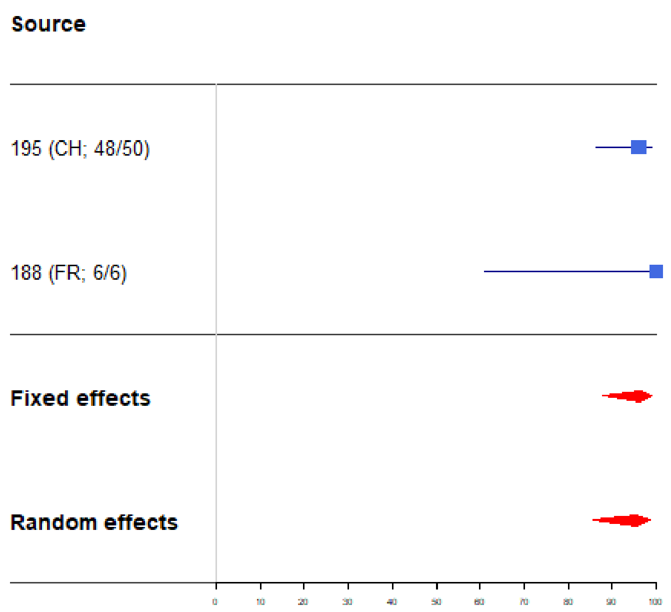
RNA (PCR)	BioMerieux, ARGENE SARS-CoV-2 R-GENE	RdRP	SPEC	na	100/100	100.0 (96.3-100.0)		195; CH	na
RNA (PCR)	BioMerieux, ARGENE SARS-CoV-2 R-GENE	RdRP or E	SENS	unk	6/6	100.0 (61.0-100.0)		188; FR	na
RNA (PCR)	BioMerieux, ARGENE SARS-CoV-2 R-GENE	RdRP or E or N	SENS	unk	6/6	100.0 (61.0-100.0)		188; FR	na
RNA (PCR)	BioMerieux, ARGENE SARS-CoV-2 R-GENE	RdRP or N	SENS	unk	6/6	100.0 (61.0-100.0)		188; FR	na
RNA (PCR)	Bioneer, AccuPower SARS-CoV-2 Real-Time RT-PCR Kit	E	SENS	unk	50/50	100.0 (92.9-100.0)		195; CH	na
RNA (PCR)	Bioneer, AccuPower SARS-CoV-2 Real-Time RT-PCR Kit	E	SPEC	na	100/100	100.0 (96.3-100.0)		195; CH	na
RNA (PCR)	Bioneer, AccuPower SARS-CoV-2 Real-Time RT-PCR Kit	RdRP	SENS	unk	50/50	100.0 (92.9-100.0)		195; CH	na
RNA (PCR)	Bioneer, AccuPower SARS-CoV-2 Real-Time RT-PCR Kit	RdRP	SPEC	na	100/100	100.0 (96.3-100.0)		195; CH	na
RNA (PCR)	Boditech, ExAmplar COVID-19 real-time PCR kit	E	SENS	unk	50/50	100.0 (92.9-100.0)		195; CH	na
RNA (PCR)	Boditech, ExAmplar COVID-19 real-time PCR kit	E	SPEC	na	100/100	100.0 (96.3-100.0)		195; CH	na
RNA (PCR)	Boditech, ExAmplar COVID-19 real-time PCR kit	RdRP	SENS	unk	45/50	90.0 (78.6-95.7)		195; CH	na
RNA (PCR)	Boditech, ExAmplar COVID-19 real-time PCR kit	RdRP	SPEC	na	100/100	100.0 (96.3-100.0)		195; CH	na
RNA (PCR, POC)	Cepheid, GeneXpert Xpert Xpress SARS-CoV-2	E or N	SENS	unk	422/427	98.8 (97.3-99.5)	0.0	21, 31, 76, 140, 178, 189, 190, 232, 239, 266, 276, 300, 328; BE, CH, CY, DE, FI, FR, NL, SE, US(5)	

									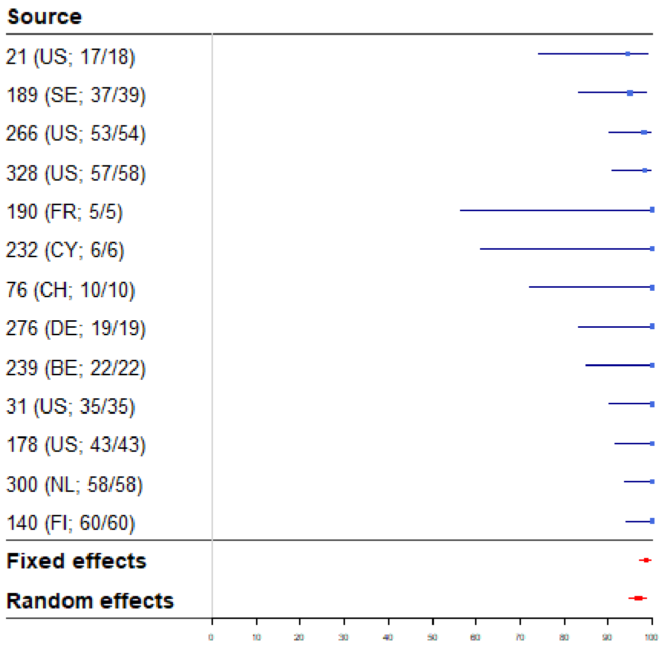
RNA (PCR, POC)	Cepheid, GeneXpert Xpert Xpress SARS-CoV-2	E or N	SPEC	na	18/18	100.0 (82.4-100.0)	0.0	76, 189, 239; BE, CH, SE	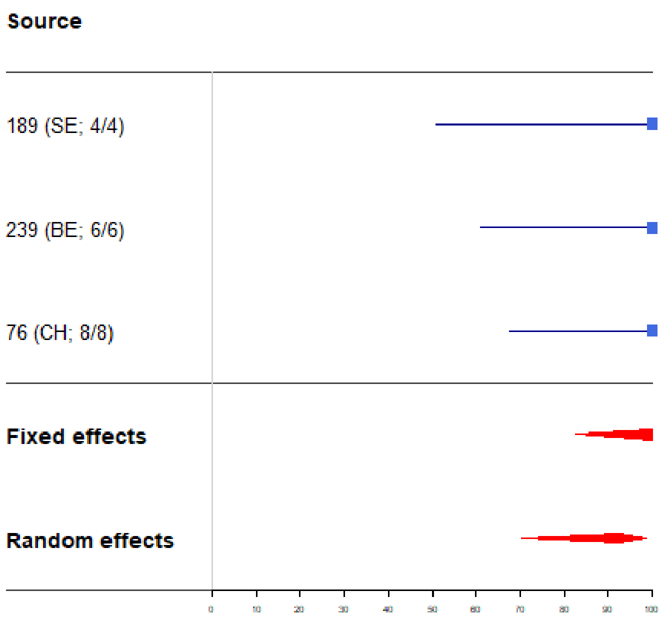
RNA (PCR)	CerTest Biotec, VIASURE SARS-CoV-2 Real Time PCR Detection Kit	N	SENS	unk	61/63	96.8 (89.1-99.1)	69.2	195, 330; CH, NL	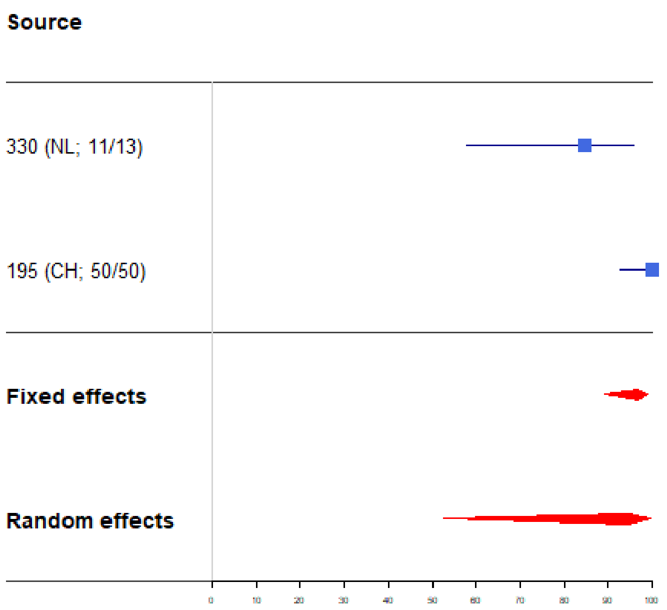
RNA (PCR)	CerTest Biotec, VIASURE SARS-CoV-2 Real Time PCR Detection Kit	N	SPEC	na	112/112	100.0 (96.7-100.0)	5.9	195, 330; CH, NL	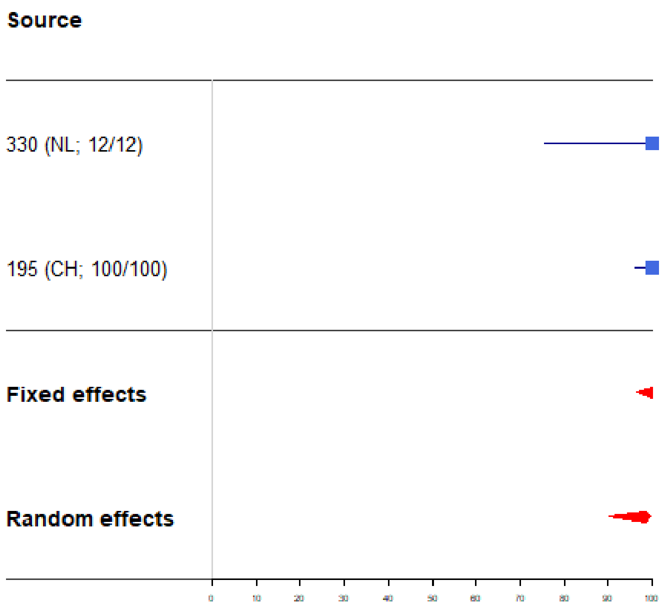
RNA (PCR)	CerTest Biotec,	ORF1ab	SENS	unk	59/63	93.7 (84.8-	79.9	195, 330; CH, NL	

	VIASURE SARS-CoV-2 Real Time PCR Detection Kit					97.5)			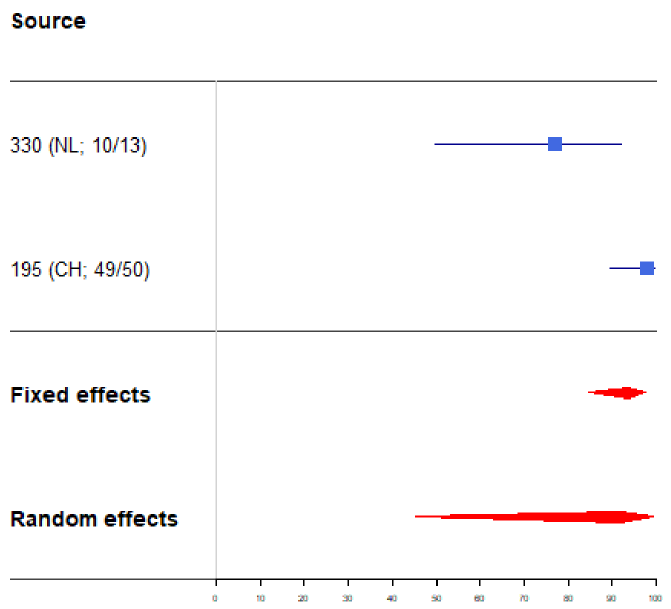
RNA (PCR)	CerTest Biotec, VIASURE SARS-CoV-2 Real Time PCR Detection Kit	ORF1ab	SPEC	na	112/112	100.0 (96.7-100.0)	5.9	195, 330; CH, NL	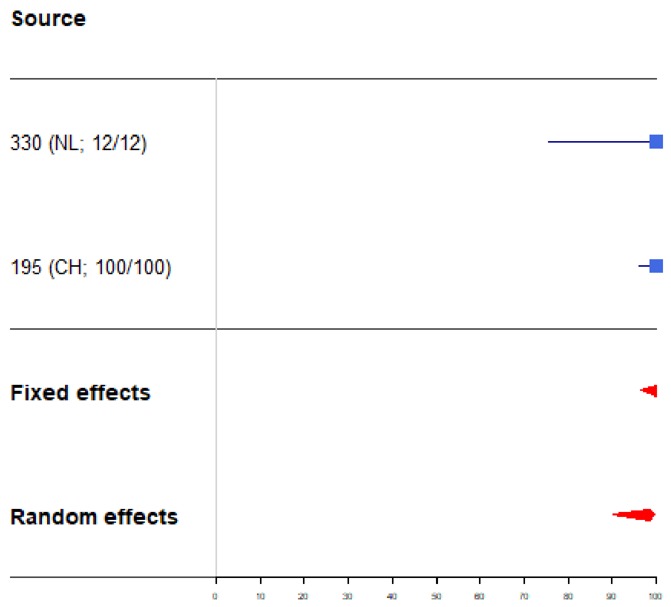
RNA (PCR)	CerTest Biotec, VIASURE SARS-CoV-2 Real Time PCR Detection Kit	ORF1ab or N	SENS	unk	11/13	84.6 (57.8-95.7)		330; NL	na
RNA (PCR)	CerTest Biotec, VIASURE SARS-CoV-2 Real Time PCR Detection Kit	ORF1ab or N	SPEC	na	207/207	100.0 (98.2-100.0)	46.0	192, 330; NL, UK	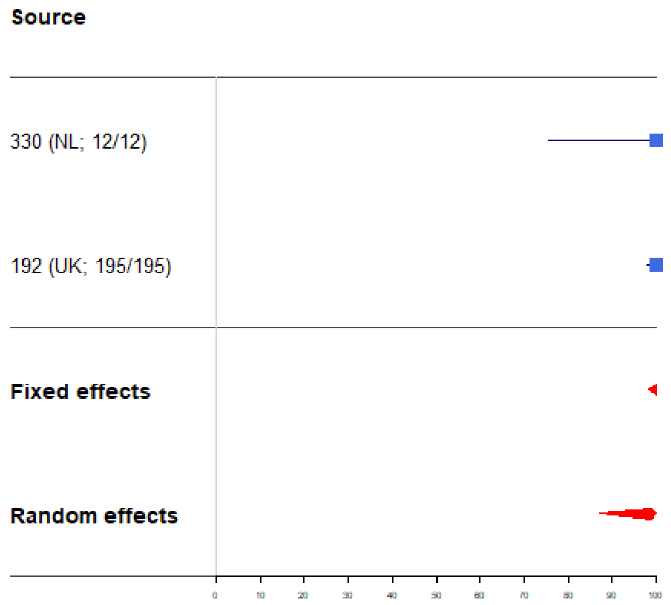
RNA (PCR)	CerTest Biotec, VIASURE SARS-CoV-2 Real Time PCR Detection Kit	S	SENS	unk	52/75	69.3 (58.2-78.6)	0.0	169, 191; AT, FR	

									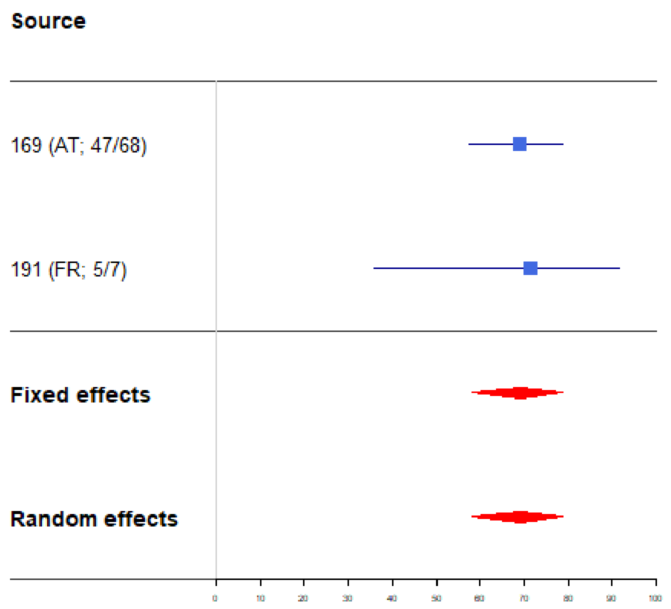
RNA (PCR)	Da An Gene, 2019-nCoV RT-PCR	N	SENS	unk	83/84	98.8 (93.6-99.8)	0.0	195, 198; CH, HR	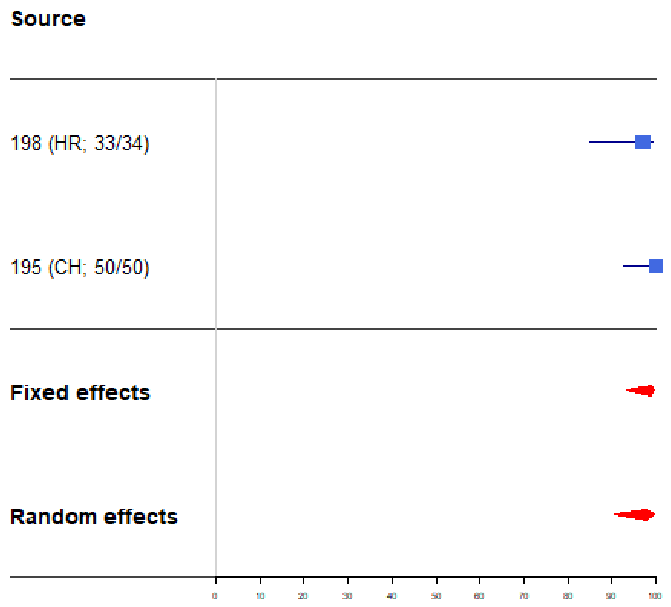
RNA (PCR)	Da An Gene, 2019-nCoV RT-PCR	N	SPEC	na	98/100	98.0 (93.0-99.4)		195; CH	na
RNA (PCR)	Da An Gene, 2019-nCoV RT-PCR	ORF1ab	SENS	unk	83/84	98.8 (93.6-99.8)	0.0	195, 198; CH, HR	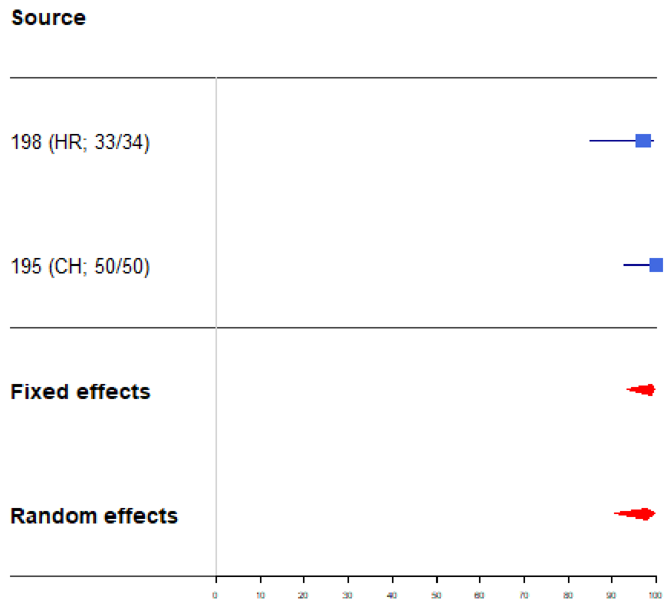
RNA (PCR)	Da An Gene, 2019-nCoV RT-PCR	ORF1ab	SPEC	na	96/100	96.0 (90.2-98.4)		195; CH	na
RNA (PCR)	DiaSorin, Simplexa COVID-19 Direct RT-PCR Kit	ORF1ab or S	SENS	unk	176/180	97.8 (94.4-99.1)	0.0	52, 251, 327; US(3)	

									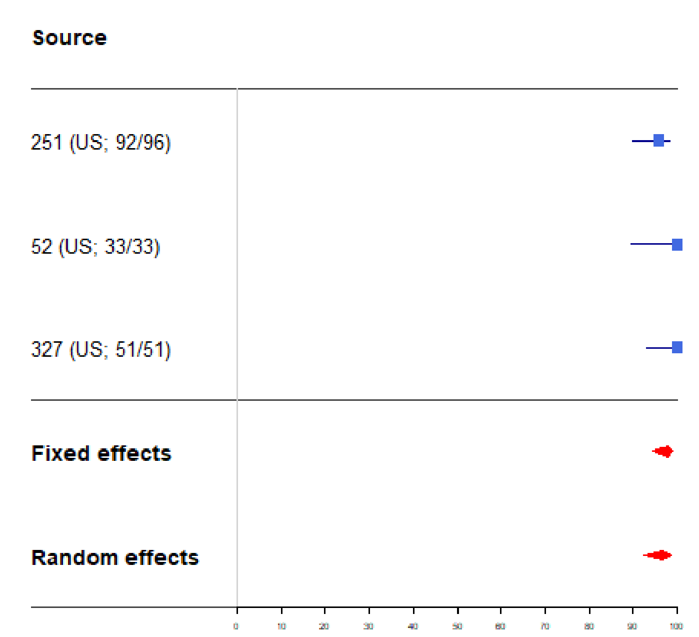
RNA (LAMP)	Eiken Chemical, Loopamp 2019-SARS-CoV-2 Detection Reagent Kit	na	SENS	hospitalised	30/30	100.0 (88.6-100.0)		144; JP	na
RNA (LAMP)	Eiken Chemical, Loopamp 2019-SARS-CoV-2 Detection Reagent Kit	na	SENS	unk	55/68	80.9 (70.0-88.5)		168; JP	na
RNA (LAMP)	Eiken Chemical, Loopamp 2019-SARS-CoV-2 Detection Reagent Kit	na	SPEC	na	24/24	100.0 (86.2-100.0)		144; JP	na
RNA (PCR)	Euroimmun Medizinische Labordiagnostika, EURORealTime SARS-CoV-2 PCR	ORF1ab or N	SENS	unk	50/50	100.0 (92.9-100.0)		195; CH	na
RNA (PCR)	Euroimmun Medizinische Labordiagnostika, EURORealTime SARS-CoV-2 PCR	ORF1ab or N	SPEC	na	98/100	98.0 (93.0-99.4)		195; CH	na
RNA (PCR)	GenMark Diagnostics, ePlex SARS-CoV-2 Test	N	SENS	unk	102/109	93.6 (87.3-96.9)	0.0	327, 328; US(2)	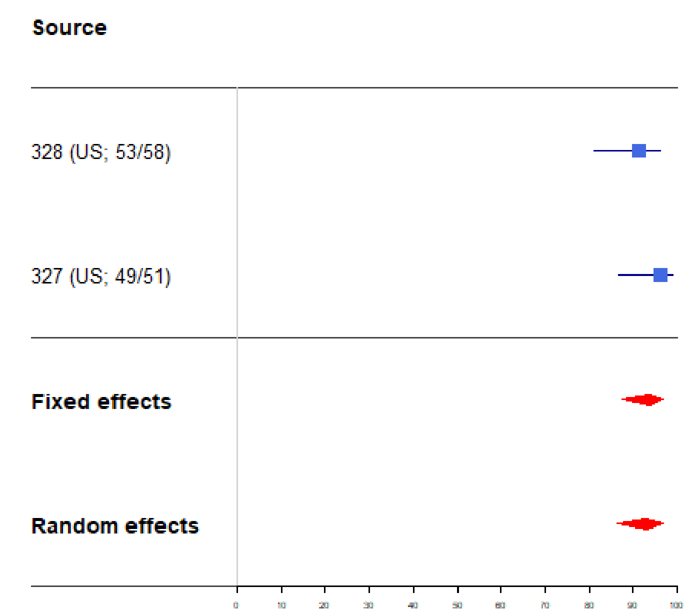
RNA (PCR)	GeneFirst, The Novel Coronavirus (2019-nCoV) Nucleic Acid Test Kit	N	SENS	unk	49/50	98.0 (89.5-99.6)		195; CH	na
RNA (PCR)	GeneFirst, The Novel Coronavirus (2019-nCoV) Nucleic Acid Test Kit	N	SPEC	na	100/100	100.0 (96.3-100.0)		195; CH	na
RNA (PCR)	GeneFirst, The Novel Coronavirus (2019-nCoV) Nucleic Acid Test Kit	ORF1ab	SENS	unk	50/50	100.0 (92.9-100.0)		195; CH	na
RNA (PCR)	GeneFirst, The Novel Coronavirus (2019-nCoV) Nucleic Acid Test Kit	ORF1ab	SPEC	na	99/100	99.0 (94.6-99.8)		195; CH	na
RNA (PCR)	GeneFirst, The Novel Coronavirus (2019-nCoV) Nucleic Acid Test Kit	ORF1ab or N	SPEC	na	195/195	100.0 (98.1-100.0)		193; UK	na
RNA (PCR)	Genetic Signatures, EasyScreen Pan-Coronavirus/SARS-CoV-2 Detection Kit	E or N	SPEC	na	195/195	100.0 (98.1-100.0)		194; UK	na
RNA (PCR)	Gerbion, virellaSARS-CoV-2 seqc Multiplex for detection of RNA of SARS-CoV-2 and other Betacoronaviruses	E	SENS	unk	1/5	20.0 (3.6-62.4)		199; PL	na
RNA (TMA-DKA)	Hologic, Aptima SARS-CoV-2 Assay (Panther System)	ORF1ab	SENS	unk	232/245	94.7 (91.1-96.9)	0.0	239, 260, 283; BE, FR, US	

									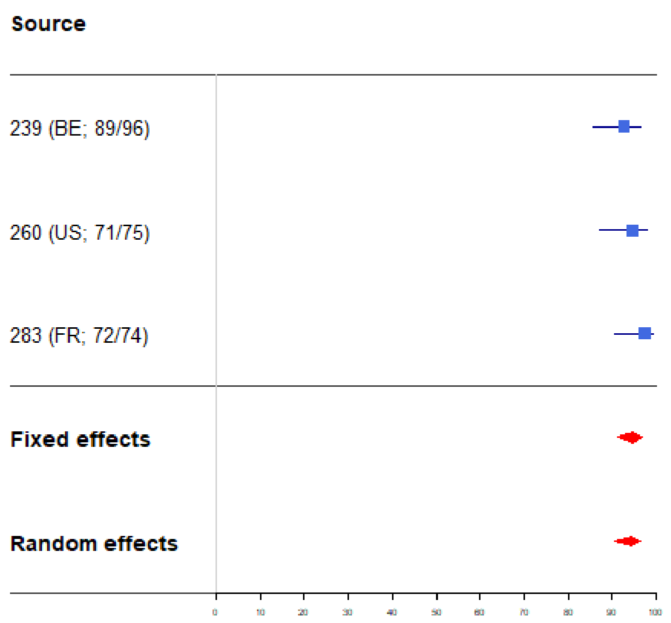
RNA (TMA-DKA)	Hologic, Aptima SARS-CoV-2 Assay (Panther System)	ORF1ab	SPEC	na	62/62	100.0 (94.2-100.0)		239; BE	na
RNA (PCR)	Hologic, SARS-CoV-2 Assay (Panther Fusion System)	ORF1ab	SENS	unk	516/525	98.3 (96.8-99.1)	29.8	53, 86, 260, 266, 283, 327, 328; FR, US(6)	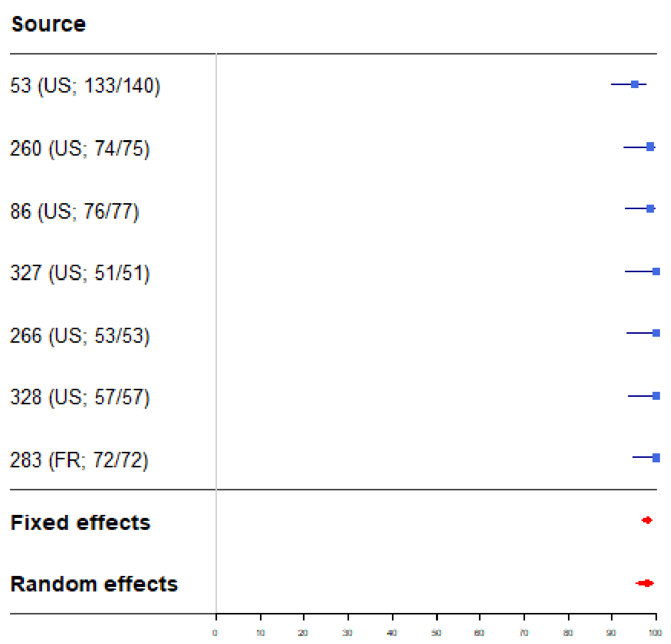
RNA (PCR)	KH Medical, RADI COVID-19 Detection Kit and RADI COVID-19 Triple Detection Kit	RdRP	SENS	unk	61/63	96.8 (89.1-99.1)	69.2	195, 330; CH, NL	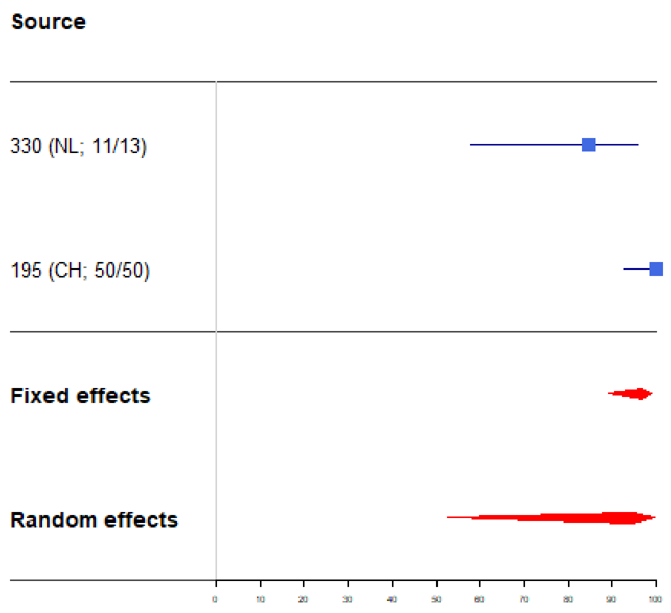
RNA (PCR)	KH Medical, RADI COVID-19 Detection Kit and RADI COVID-19 Triple Detection Kit	RdRP	SPEC	na	112/112	100.0 (96.7-100.0)	5.9	195, 330; CH, NL	

									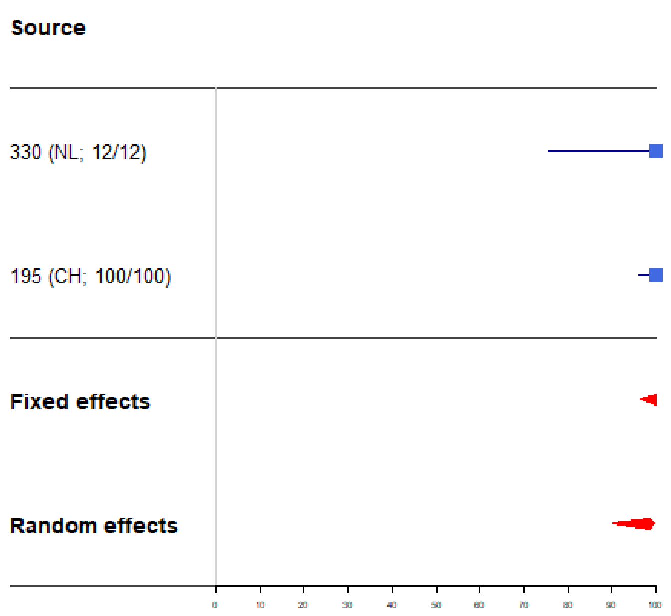
RNA (PCR)	KH Medical, RADI COVID-19 Detection Kit and RADI COVID-19 Triple Detection Kit	RdRP or S	SENS	unk	12/13	92.3 (66.7-98.6)		330; NL	na
RNA (PCR)	KH Medical, RADI COVID-19 Detection Kit and RADI COVID-19 Triple Detection Kit	RdRP or S	SPEC	na	12/12	100.0 (75.8-100.0)		330; NL	na
RNA (PCR)	KH Medical, RADI COVID-19 Detection Kit and RADI COVID-19 Triple Detection Kit	S	SENS	unk	62/63	98.4 (91.5-99.7)	31.6	195, 330; CH, NL	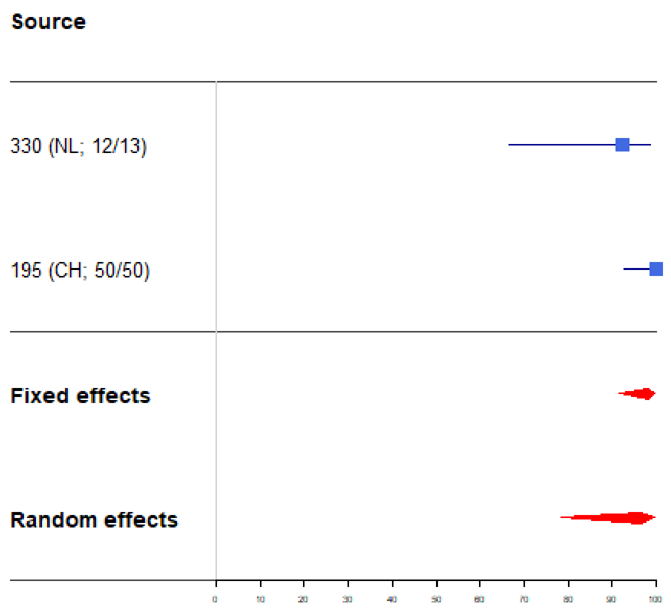
RNA (PCR)	KH Medical, RADI COVID-19 Detection Kit and RADI COVID-19 Triple Detection Kit	S	SPEC	na	112/112	100.0 (96.7-100.0)	5.9	195, 330; CH, NL	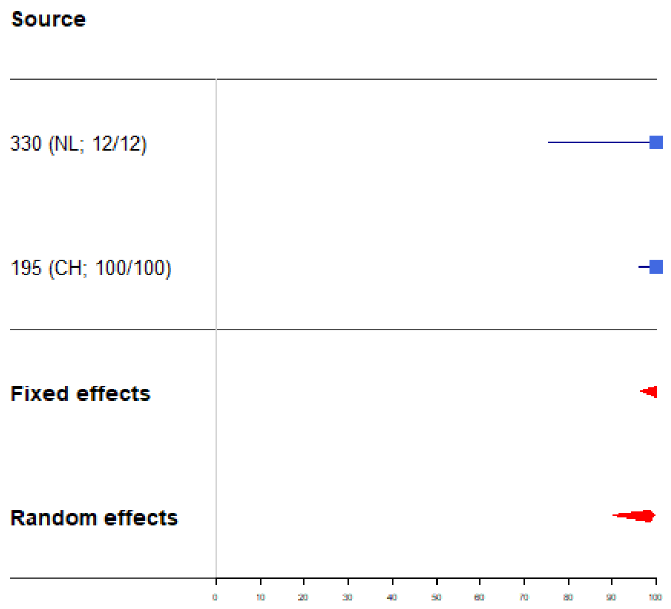
RNA (PCR)	Liferiver Bio-Tech, Novel Coronavirus (2019-nCoV) Real Time Multiplex RT-PCR kit	ORF1ab or E or N	SENS	unk	35/38	92.1 (79.2-97.3)		196; PL	na
RNA (PCR)	Luminex, ARIES SARS-CoV-2 Assay	ORF1ab or N	SENS	unk	19/30	63.3 (45.5-78.1)		276; DE	na
RNA (PCR)	Luminex, NxTAG CoV Extended Panel	E	SENS	unk	88/91	96.7 (90.8-98.9)		45; CN	na
RNA (PCR)	Luminex, NxTAG CoV Extended Panel	N	SENS	unk	78/91	85.7 (77.1-91.5)		45; CN	na
RNA (PCR)	Luminex, NxTAG CoV Extended Panel	ORF1ab	SENS	unk	89/91	97.8 (92.3-99.4)		45; CN	na
RNA (PCR)	Luminex, NxTAG CoV Extended Panel	ORF1ab or E or N	SENS	unk	89/91	97.8 (92.3-99.4)		45; CN	na
RNA (PCR, POC)	Mesa Biotech, Accula RSV Molecular POCT	N	SENS	unk	34/50	68.0 (54.2-79.2)		87; US	na

RNA (PCR, POC)	MiCo BioMed, Coronavirus disease 2019(COVID-19) Detection Kit	ORF3a or N	SENS	hospitalised	22/33	66.7 (49.6-80.2)		70; EC	na
RNA (PCR, POC)	Mobidiag, Novodiag COVID-19	ORF1ab or N	SENS	unk	57/61	93.4 (84.3-97.4)		140; FI	na
RNA (PCR)	Nanjing Vazyme Medical Technology, 2019-Novel Coronavirus (2019-nCoV) Triplex RT-qPCR Detection Kit	N	SENS	unk	15/17	88.2 (65.7-96.7)		199; PL	na
RNA (PCR)	Nanjing Vazyme Medical Technology, 2019-Novel Coronavirus (2019-nCoV) Triplex RT-qPCR Detection Kit	ORF1ab	SENS	unk	14/17	82.4 (59.0-93.8)		199; PL	na
RNA (PCR)	Nanjing Vazyme Medical Technology, 2019-Novel Coronavirus (2019-nCoV) Triplex RT-qPCR Detection Kit	ORF1ab or N	SENS	unk	15/17	88.2 (65.7-96.7)		199; PL	na
RNA (PCR)	NeuMoDx, SARS-CoV-2 Assay	N or Nsp2	SENS	unk	105/106	99.1 (94.8-99.8)		179; US	na
RNA (PCR)	OsangHealthcare, GeneFinder COVID-19 Plus RealAmp Kit	E or N	SENS	unk	17/20	85.0 (64.0-94.8)		68; FR	na
RNA (PCR)	PathoFinder, RealAccurate Quadruplex Corona-plus PCR Kit	RdRP or N	SENS	unk	32/34	94.1 (80.9-98.4)		198; HR	na
RNA (PCR)	PerkinElmer, SARS-CoV-2 Real-time RT-PCR Assay	N	SENS	unk	50/50	100.0 (92.9-100.0)		195; CH	na
RNA (PCR)	PerkinElmer, SARS-CoV-2 Real-time RT-PCR Assay	N	SPEC	na	99/100	99.0 (94.6-99.8)		195; CH	na
RNA (PCR)	PerkinElmer, SARS-CoV-2 Real-time RT-PCR Assay	ORF1ab	SENS	unk	50/50	100.0 (92.9-100.0)		195; CH	na
RNA (PCR)	PerkinElmer, SARS-CoV-2 Real-time RT-PCR Assay	ORF1ab	SPEC	na	100/100	100.0 (96.3-100.0)		195; CH	na
RNA (PCR)	Primerdesign, genesig Real-Time PCR CoVID-19 kit	RdRP	SENS	unk	101/106	95.3 (89.4-98.0)	70.0	195, 199, 330; CH, NL, PL	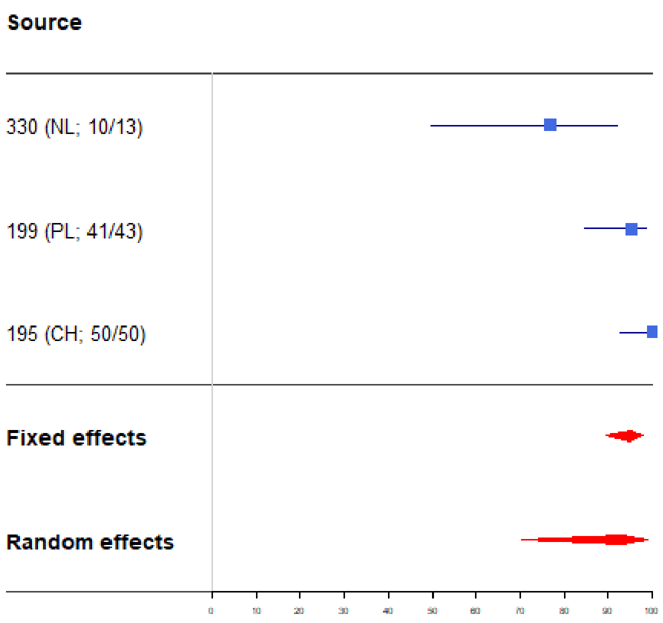
RNA (PCR)	Primerdesign, genesig Real-Time PCR CoVID-19 kit	RdRP	SPEC	na	307/307	100.0 (98.8-100.0)	0.0	195, 200, 330; CH, NL, UK	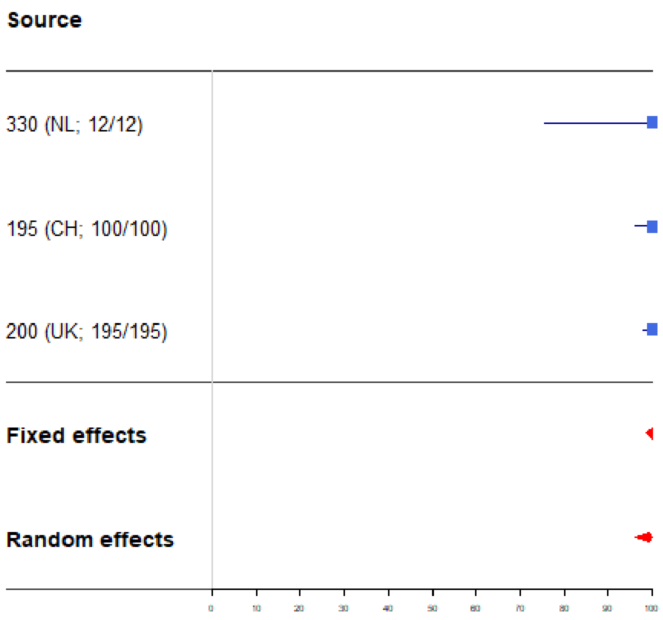
RNA (PCR)	Qiagen, QIAstat-Dx Respiratory SARS-CoV-2 Panel	ORF1ab or E	SENS	hospitalised	40/40	100.0 (91.2-100.0)		287; FR	na
RNA (PCR)	R-Biopharm, Ridagene SARS-CoV2	E	SENS	unk	63/63	100.0 (94.3-100.0)	0.0	195, 330; CH, NL	

									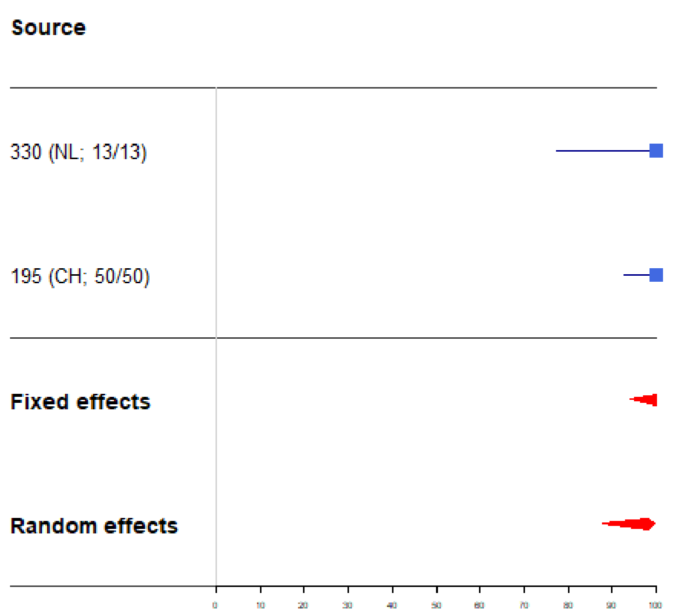
RNA (PCR)	R-Biopharm, Ridagene SARS-CoV2	E	SPEC	na	112/112	100.0 (96.7-100.0)	5.9	195, 330; CH, NL	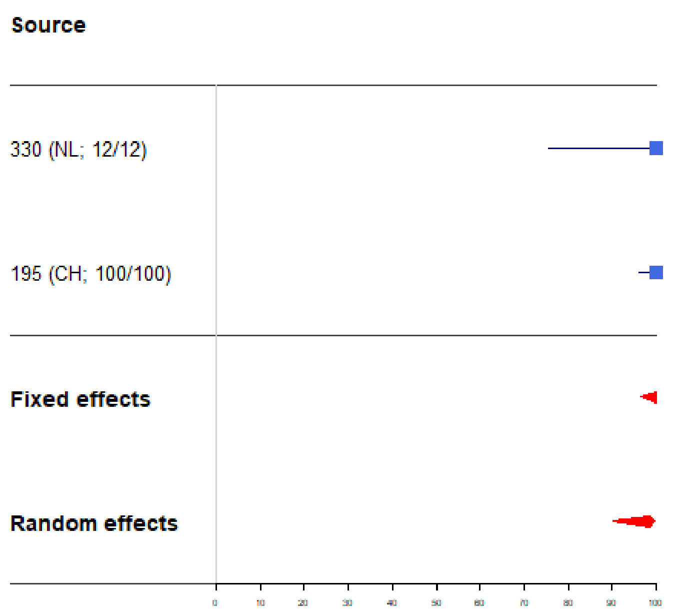
RNA (PCR)	Randox Laboratories, Extended Coronavirus Array	na	SPEC	na	195/195	100.0 (98.1-100.0)		201; UK	na
RNA (PCR)	Roche, COBAS SARS-CoV-2 test	E	SENS	unk	15/15	100.0 (79.6-100.0)		202; FR	na
RNA (PCR)	Roche, COBAS SARS-CoV-2 test	ORF1ab	SENS	unk	15/15	100.0 (79.6-100.0)		202; FR	na
RNA (PCR)	Roche, COBAS SARS-CoV-2 test	ORF1ab or E	SENS	unk	1111/1125	98.8 (97.9-99.3)	31.8	31, 52, 53, 76, 169, 178, 202, 210, 229, 246; AT, CH, DE, FR, SI, US(5)	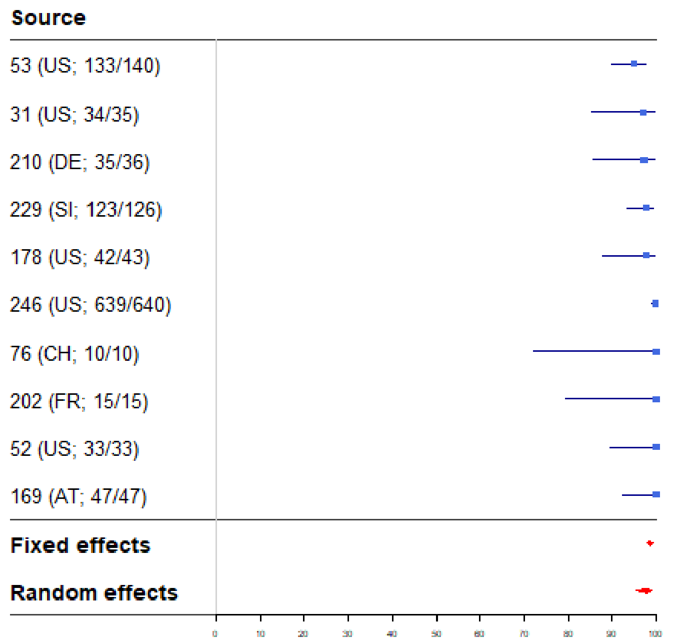
RNA (PCR)	Roche, COBAS SARS-CoV-2 test	ORF1ab or E	SPEC	na	38/38	100.0 (90.8-100.0)	0.0	76, 298; CH, FR	

									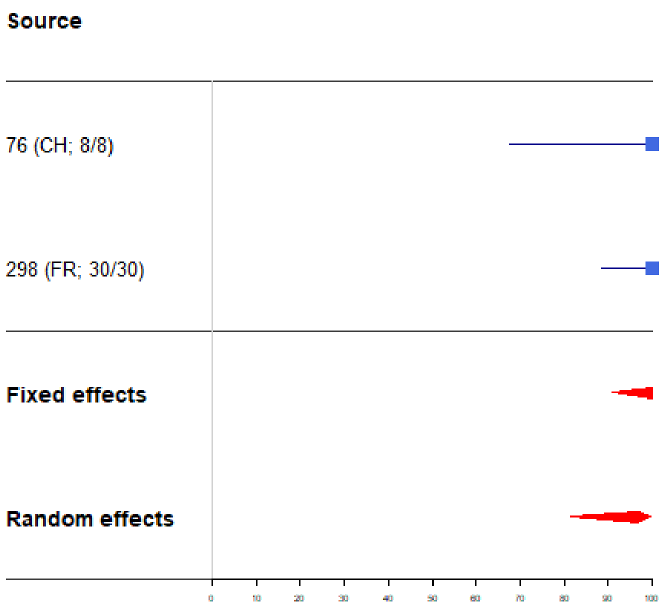
RNA (PCR)	Roche, LightMix Modular SARS-CoV E/N/RdRP	E	SENS	hospitalised	71/72	98.6 (92.5-99.8)		313; HK	na
RNA (PCR)	Roche, LightMix Modular SARS-CoV E/N/RdRP	E	SENS	unk	73/82	89.0 (80.4-94.1)	0.6	168, 240; JP, SI	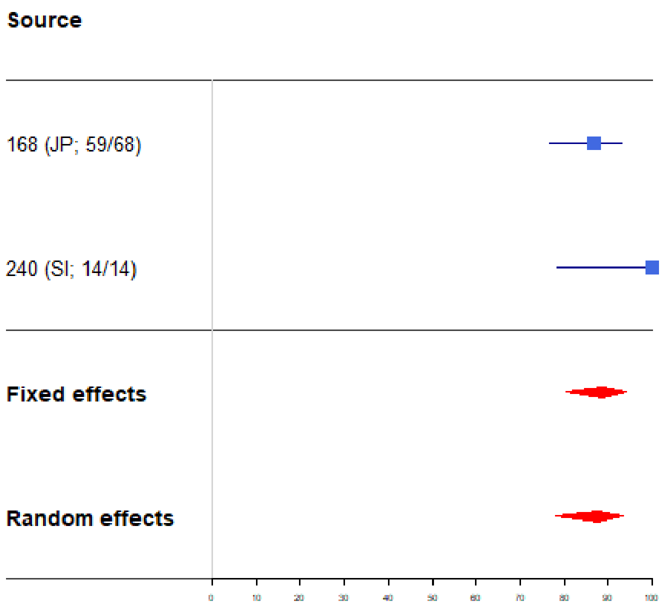
RNA (PCR)	Roche, LightMix Modular SARS-CoV E/N/RdRP	N	SENS	unk	56/82	68.3 (57.6-77.4)	0.0	168, 240; JP, SI	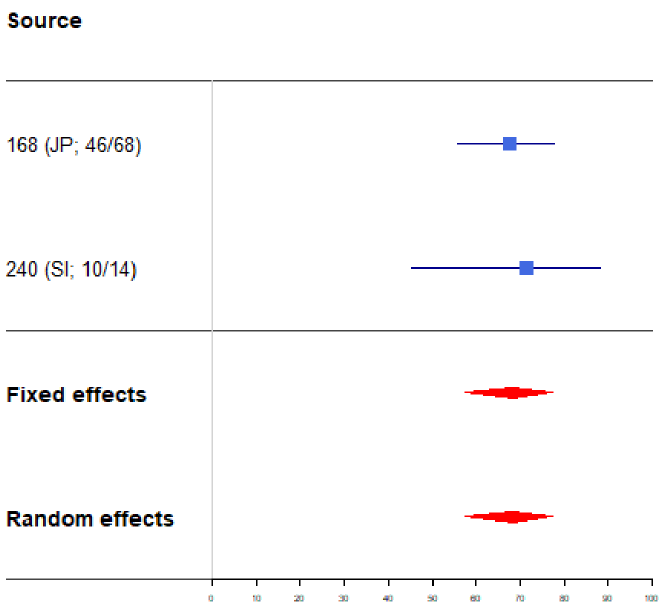
RNA (PCR)	Roche, LightMix Modular SARS-CoV E/N/RdRP	RdRP	SENS	unk	43/82	52.4 (41.8-62.9)	84.1	168, 240; JP, SI	

									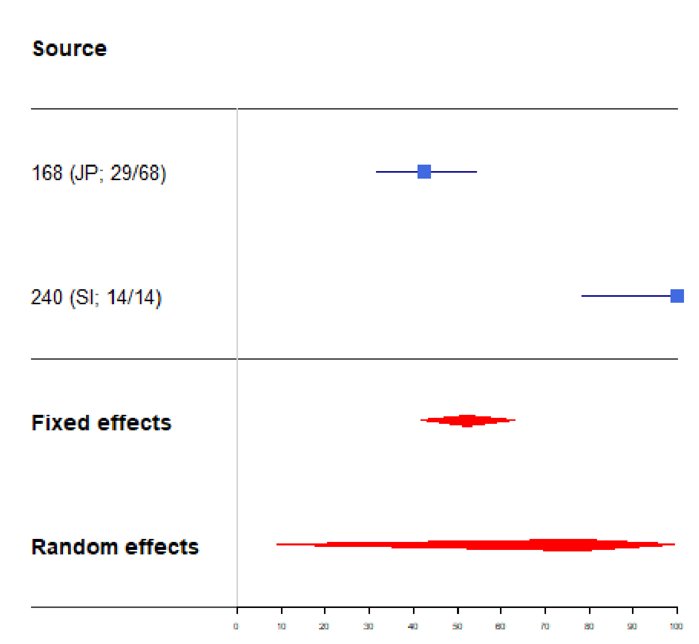
RNA (PCR)	SD BioSensor, Standard M nCoV Real-Time Detection Kit	E	SENS	unk	68/68	100.0 (94.7-100.0)	0.0	195, 233; CH, PL	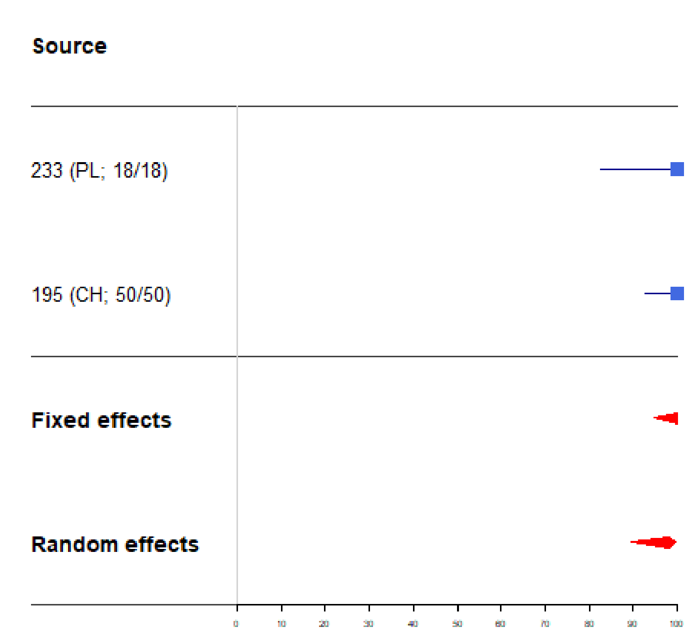
RNA (PCR)	SD BioSensor, Standard M nCoV Real-Time Detection Kit	E	SPEC	na	97/100	97.0 (91.5-99.0)		195; CH	na
RNA (PCR)	SD BioSensor, Standard M nCoV Real-Time Detection Kit	ORF1ab	SENS	unk	50/50	100.0 (92.9-100.0)		195; CH	na
RNA (PCR)	SD BioSensor, Standard M nCoV Real-Time Detection Kit	ORF1ab	SPEC	na	99/100	99.0 (94.6-99.8)		195; CH	na
RNA (PCR)	SD BioSensor, Standard M nCoV Real-Time Detection Kit	RdRP	SENS	unk	16/18	88.9 (67.2-96.9)		233; PL	na
RNA (PCR)	SD BioSensor, Standard M nCoV Real-Time Detection Kit	RdRP or E	SENS	unk	18/18	100.0 (82.4-100.0)		233; PL	na
RNA (PCR)	Sansure Biotech, 2019-nCoV nucleic acid diagnostic kit	N	SENS	unk	50/50	100.0 (92.9-100.0)		195; CH	na
RNA (PCR)	Sansure Biotech, 2019-nCoV nucleic acid diagnostic kit	N	SPEC	na	95/100	95.0 (88.8-97.8)		195; CH	na
RNA (PCR)	Sansure Biotech, 2019-nCoV nucleic acid diagnostic kit	ORF1ab	SENS	unk	50/50	100.0 (92.9-100.0)		195; CH	na
RNA (PCR)	Sansure Biotech, 2019-nCoV nucleic acid diagnostic kit	ORF1ab	SPEC	na	100/100	100.0 (96.3-100.0)		195; CH	na
RNA (PCR)	Seegene, Allplex 2019-nCoV assay	E	SENS	unk	68/80	85.0 (75.6-91.2)	87.1	68, 195, 330; CH, FR, NL	

									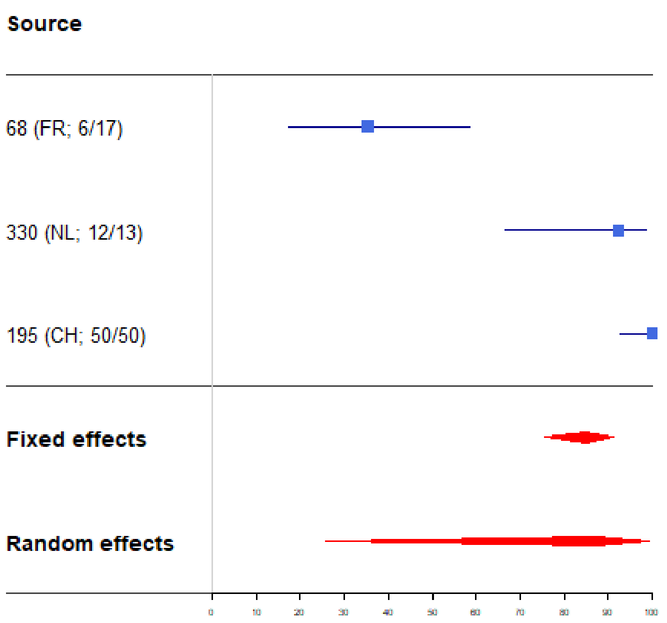
RNA (PCR)	Seegene, Allplex 2019-nCoV assay	E	SPEC	na	112/112	100.0 (96.7-100.0)	5.9	195, 330; CH, NL	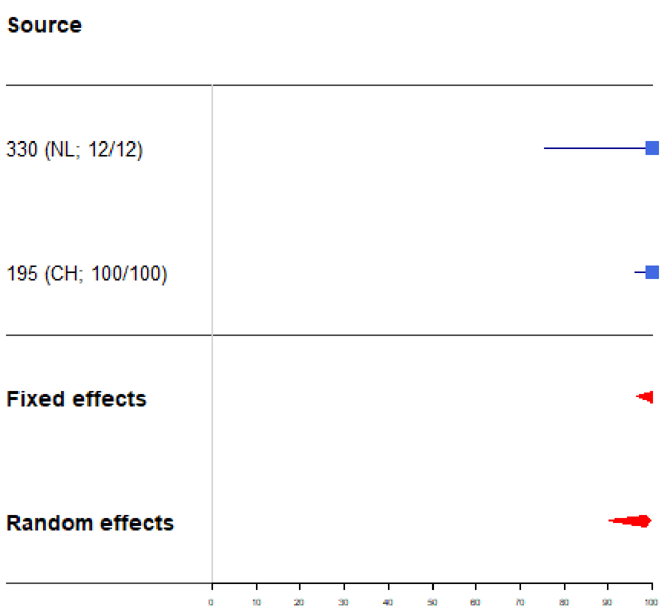
RNA (PCR)	Seegene, Allplex 2019-nCoV assay	E or N	SENS	unk	14/17	82.4 (59.0-93.8)		68; FR	na
RNA (PCR)	Seegene, Allplex 2019-nCoV assay	N	SENS	unk	60/67	89.6 (80.0-94.8)	87.5	68, 195; CH, FR	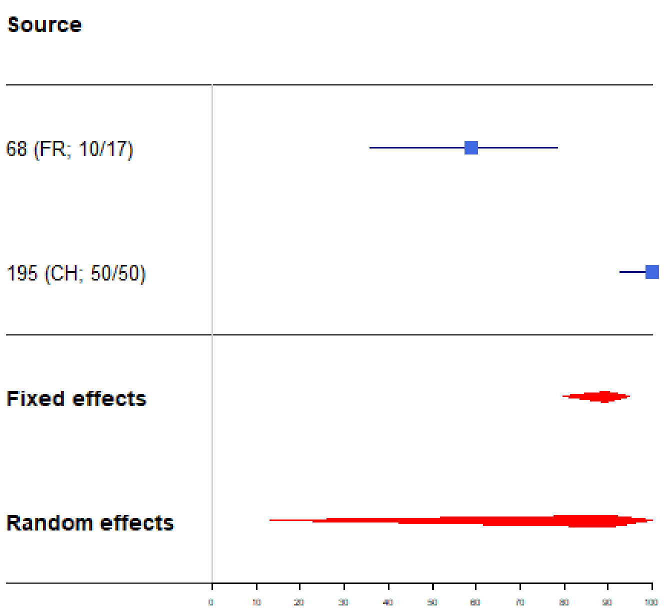
RNA (PCR)	Seegene, Allplex 2019-nCoV assay	N	SPEC	na	100/100	100.0 (96.3-100.0)		195; CH	na
RNA (PCR)	Seegene, Allplex 2019-nCoV assay	RdRP	SENS	unk	73/80	91.3 (83.0-95.7)	74.4	68, 195, 330; CH, FR, NL	

									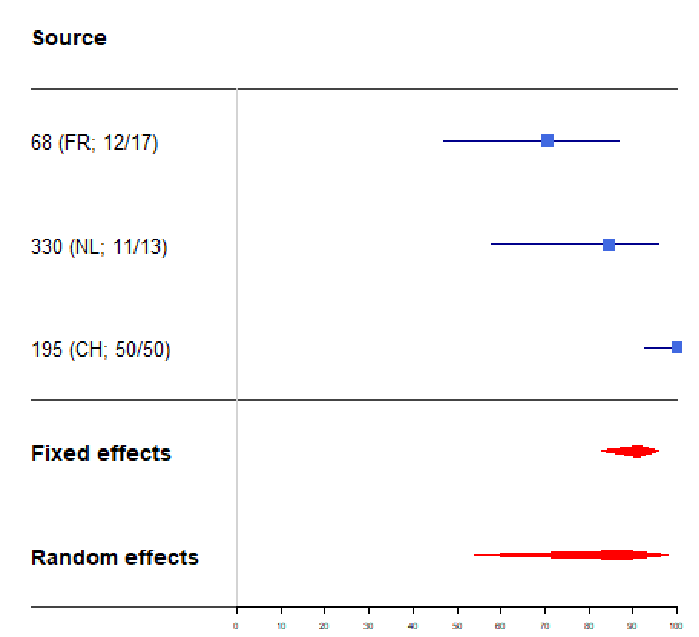
RNA (PCR)	Seegene, Allplex 2019-nCoV assay	RdRP	SPEC	na	112/112	100.0 (96.7-100.0)	5.9	195, 330; CH, NL	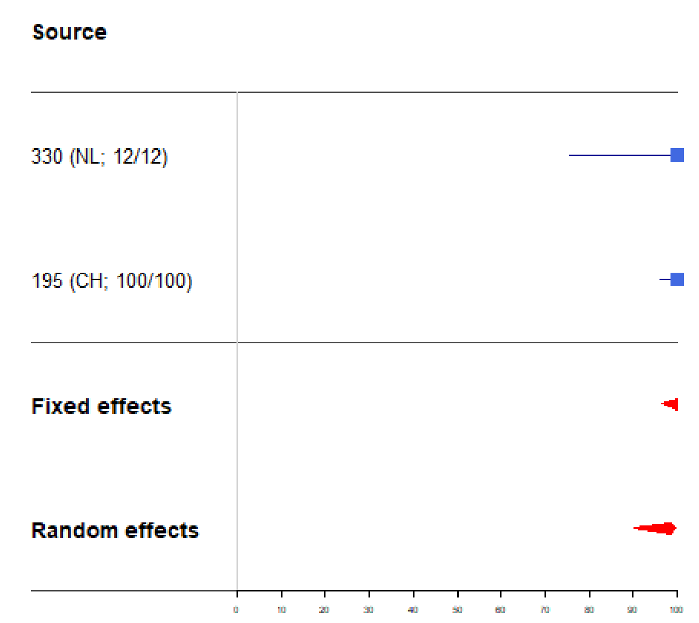
RNA (PCR)	Seegene, Allplex 2019-nCoV assay	RdRP or E	SENS	unk	27/30	90.0 (74.4-96.5)	19.6	68, 330; FR, NL	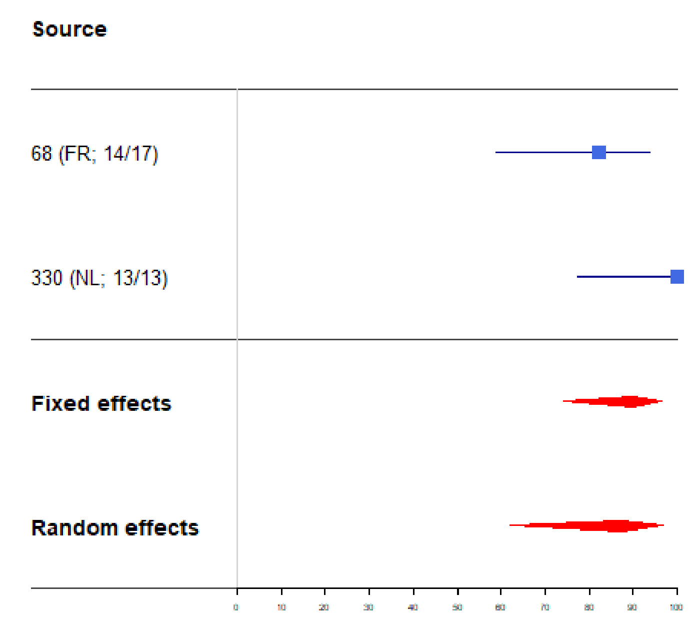
RNA (PCR)	Seegene, Allplex 2019-nCoV assay	RdRP or E	SPEC	na	12/12	100.0 (75.8-100.0)		330; NL	na
RNA (PCR)	Seegene, Allplex 2019-nCoV assay	RdRP or E or N	SENS	unk	17/17	100.0 (81.6-100.0)		68; FR	na
RNA (PCR)	Seegene, Allplex 2019-nCoV assay	RdRP or N	SENS	unk	17/17	100.0 (81.6-100.0)		68; FR	na
RNA (PCR)	Seegene, Allplex 2019-nCoV assay	RdRP or N	SPEC	na	195/195	100.0 (98.1-100.0)		203; UK	na
RNA (PCR)	Shanghai Kehua Bio-Engineering, KHB Diagnostic kit for SARS-CoV-2 Nucleic Acid (Real-time PCR)	E	SENS	unk	50/50	100.0 (92.9-100.0)		195; CH	na
RNA (PCR)	Shanghai Kehua Bio-Engineering, KHB Diagnostic kit for SARS-CoV-2 Nucleic Acid (Real-time PCR)	E	SPEC	na	100/100	100.0 (96.3-100.0)		195; CH	na
RNA (PCR)	Shanghai Kehua Bio-Engineering, KHB Diagnostic kit for SARS-	N	SENS	unk	50/50	100.0 (92.9-100.0)		195; CH	na

	CoV-2 Nucleic Acid (Real-time PCR)								
RNA (PCR)	Shanghai Kehua Bio-Engineering, KHB Diagnostic kit for SARS-CoV-2 Nucleic Acid (Real-time PCR)	N	SPEC	na	100/100	100.0 (96.3-100.0)		195; CH	na
RNA (PCR)	Shanghai Kehua Bio-Engineering, KHB Diagnostic kit for SARS-CoV-2 Nucleic Acid (Real-time PCR)	ORF1ab	SENS	unk	50/50	100.0 (92.9-100.0)		195; CH	na
RNA (PCR)	Shanghai Kehua Bio-Engineering, KHB Diagnostic kit for SARS-CoV-2 Nucleic Acid (Real-time PCR)	ORF1ab	SPEC	na	100/100	100.0 (96.3-100.0)		195; CH	na
RNA (PCR)	SolGent, DiaPlexQ Novel Coronavirus (2019-nCoV) Detection Kit	N	SENS	unk	19/21	90.5 (71.1-97.3)		199; PL	na
RNA (PCR)	SolGent, DiaPlexQ Novel Coronavirus (2019-nCoV) Detection Kit	ORF1ab	SENS	unk	19/21	90.5 (71.1-97.3)		199; PL	na
RNA (PCR)	SolGent, DiaPlexQ Novel Coronavirus (2019-nCoV) Detection Kit	ORF1ab or N	SENS	unk	21/27	77.8 (59.2-89.4)		199; PL	na
RNA (PCR)	ThermoFisher, TaqPath COVID-19 CE-IVD RT-PCR Kit V2	ORF1ab or S or N	SENS	unk	108/118	91.5 (85.1-95.3)	73.8	168, 195; CH, JP	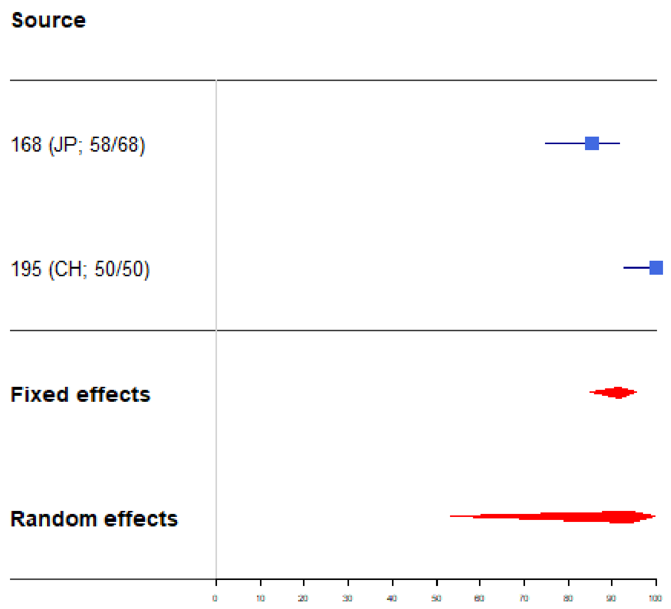
RNA (PCR)	ThermoFisher, TaqPath COVID-19 CE-IVD RT-PCR Kit V2	ORF1ab or S or N	SPEC	na	100/100	100.0 (96.3-100.0)		195; CH	na
RNA (PCR)	Tibmolbiol, SARS-CoV (COVID19) E-gene	E	SENS	unk	65/65	100.0 (94.4-100.0)	0.0	195, 204; CH, UK	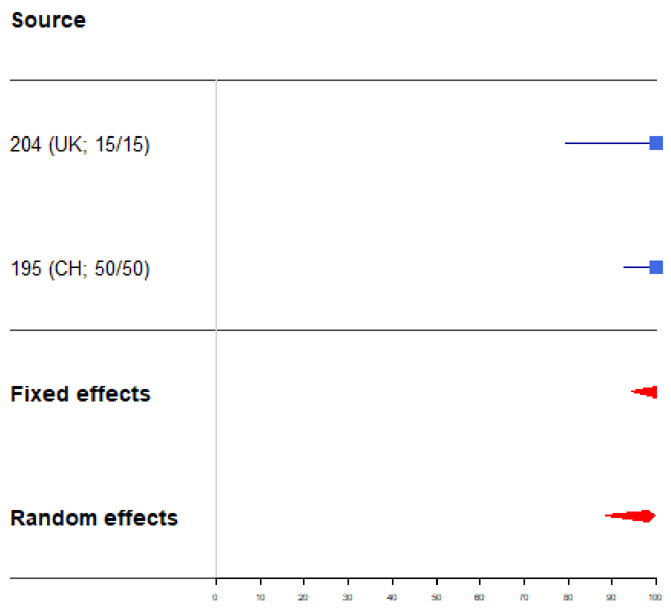
RNA (PCR)	Tibmolbiol, SARS-CoV (COVID19) E-gene	E	SPEC	na	250/250	100.0 (98.5-100.0)	0.0	195, 204; CH, UK	

										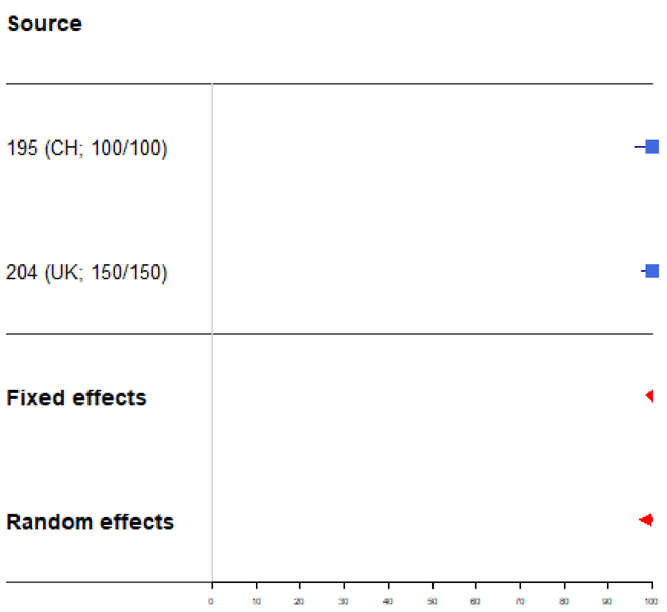
RNA (PCR)	Vela Diagnostics, ViroKey SARS-CoV-2 RT-PCR Test	ORF1ab	SENS	unk		50/50	100.0 (92.9-100.0)		195; CH	na
RNA (PCR)	Vela Diagnostics, ViroKey SARS-CoV-2 RT-PCR Test	ORF1ab	SPEC	na		100/100	100.0 (96.3-100.0)		195; CH	na
RNA (PCR)	Vela Diagnostics, ViroKey SARS-CoV-2 RT-PCR Test	RdRP	SENS	unk		47/50	94.0 (83.8-97.9)		195; CH	na
RNA (PCR)	Vela Diagnostics, ViroKey SARS-CoV-2 RT-PCR Test	RdRP	SPEC	na		100/100	100.0 (96.3-100.0)		195; CH	na
